# The accountability for reasonableness approach to guide priority setting in health systems within limited resources – findings from action research at district level in Kenya, Tanzania, and Zambia

**DOI:** 10.1186/1478-4505-12-49

**Published:** 2014-08-20

**Authors:** Jens Byskov, Bruno Marchal, Stephen Maluka, Joseph M Zulu, Salome A Bukachi, Anna-Karin Hurtig, Astrid Blystad, Peter Kamuzora, Charles Michelo, Lillian N Nyandieka, Benedict Ndawi, Paul Bloch, Øystein E Olsen

**Affiliations:** 1 DBL – Centre for Health Research and Development, Faculty of Health and Medical Sciences, University of Copenhagen, Thorvaldsensvej 57, DK 1871 Frederiksberg, Denmark; 2 Department of Public Health, Institute of Tropical Medicine, Nationalestraat 155, B 2000 Antwerpen, Belgium; 3 Institute of Development Studies, University of Dar Es Salaam, PO Box 35169, Dar Es Salaam, Tanzania; 4 Department of Public Health, School of Medicine, University of Zambia, PO Box 50110, Lusaka, Zambia; 5 Institute of Anthropology, Gender and African Studies University of Nairobi, PO Box 30197, Nairobi 00100, Kenya; 6 Umeå International School of Public Health, Umeå University, SE 90185 Umea, Sweden; 7 Department of Public Health and Primary Health Care, University of Bergen, PO Box 7804, 5020 Bergen, Norway; 8 Centre for Public Health Research, Kenya Medical Research Institute (KEMRI), PO Box 20752, Nairobi 00202, Kenya; 9 Primary Health Care Institute, PO Box 235, Iringa, Tanzania; 10 Steno Health Promotion Center, Steno Diabetes Center, Niels Steensens Vej 8, DK-2820 Gentofte, Denmark; 11 Affiliated to Centre for International Health, University of Bergen, Årstadveien 21 5th floor, N-5009 Bergen, Norway

**Keywords:** Accountability for reasonableness, Priority setting, Fairness, Decentralization, Decision making, Democratization, Health systems, Kenya, Tanzania, Zambia

## Abstract

**Background:**

Priority-setting decisions are based on an important, but not sufficient set of values and thus lead to disagreement on priorities. Accountability for Reasonableness (AFR) is an ethics-based approach to a legitimate and fair priority-setting process that builds upon four conditions: relevance, publicity, appeals, and enforcement, which facilitate agreement on priority-setting decisions and gain support for their implementation. This paper focuses on the assessment of AFR within the project REsponse to ACcountable priority setting for Trust in health systems (REACT).

**Methods:**

This intervention study applied an action research methodology to assess implementation of AFR in one district in Kenya, Tanzania, and Zambia, respectively. The assessments focused on selected disease, program, and managerial areas. An implementing action research team of core health team members and supporting researchers was formed to implement, and continually assess and improve the application of the four conditions. Researchers evaluated the intervention using qualitative and quantitative data collection and analysis methods.

**Results:**

The values underlying the AFR approach were in all three districts well-aligned with general values expressed by both service providers and community representatives. There was some variation in the interpretations and actual use of the AFR in the decision-making processes in the three districts, and its effect ranged from an increase in awareness of the importance of fairness to a broadened engagement of health team members and other stakeholders in priority setting and other decision-making processes.

**Conclusions:**

District stakeholders were able to take greater charge of closing the gap between nationally set planning and the local realities and demands of the served communities within the limited resources at hand. This study thus indicates that the operationalization of the four broadly defined and linked conditions is both possible and seems to be responding to an actual demand. This provides arguments for the continued application and further assessment of the potential of AFR in supporting priority-setting and other decision-making processes in health systems to achieve better agreed and more sustainable health improvements linked to a mutual democratic learning with potential wider implications.

## Background

Priority setting can be defined as the distribution of resources among competing interests, programs, or people, and is one of the most prominent health care policy questions [1], not least when resources are very scarce. Most efforts to strengthen district level priority setting in poorer countries have been using technical approaches based on burden of disease measures, cost effectiveness analysis, capacity considerations, and other measures that claim to be based on evidence. Such approaches are typically dominated by ‘experts’ [2,3]. However, central in priority-setting decisions are values, which in practice are rarely adequately recognized, made explicit, defined, discussed, and agreed upon. While technical approaches in priority setting may be useful and necessary, they have been questioned. First, they are based on health and economic data and do not take social, cultural, and other values into consideration. Moreover, they have not led to the intended sustainable improvements in addressing health needs and demands [4,5].

Making the values that underlie decisions in the priority-setting process explicit is important since actors tend to have diverging values; even the ways in which stakeholders relate to commonly applied values such as ‘efficiency’, ‘equity’, and ‘quality’ may conflict [6]. Such conflicts can only be managed meaningfully and productively through open discussion. Decision-making approaches that do not permit discussion and choice on the basis of relevant values tend to produce disagreement, a low sense of ownership, and controversy around both the desired outcomes and the allocation of resources. It is essential that values are made explicit because they do influence preferences among all concerned on health improvement and service options.

When agreement on desired priority outcomes is difficult to achieve, a mechanism of structured discussion and debate that contributes to legitimize the decisions made is necessary. Accountability for reasonableness (AFR) is an ethical framework for priority setting that aims at ensuring that the process towards setting priorities is fair, and that the actually decided-upon priorities are based on reasons that are communicated to all relevant parties involved. AFR thus provides decision makers with an approach to consider and jointly discuss competing values in the priority-setting process. According to AFR, a process for setting priorities is legitimate and fair if it meets four conditions. Relevance requires that decisions are founded in the values of all concerned and considered important. In practice, this means that all relevant stakeholders have the chance to participate in the process, that there is respect for differing views, and space to consider divergent opinions and preferences. The debates must be based on clear arguments, and all actors involved must be given the chance to have a voice. Publicity demands that priority-setting decisions and the reasons behind them are transparent and are made public. This can be done, for instance, through open meetings, diffusion of meeting agenda and minutes, and other communication processes. Appeals and revision require in that stakeholders are given an opportunity to appeal against decisions, propose revisions, and receive a reasoned response. This would mean that people affected by the decision have a voice and are effectively heard, and that a procedure for revision is ensured. Enforcement must ensure that the first three criteria are adhered to. This final condition is commonly referred to as leadership (of the AFR process), as arrangements must be made to ensure that there is one or more legitimate bodies able to ensure procedures for continuous application of all four conditions among the stakeholders including the public. Improving fairness and legitimacy also constitutes a democratic learning process within health systems [7-10].

AFR has attracted attention among decision makers, health care professionals, and scholars involved in empirical studies of priority setting. Applying the AFR principles is not an easy and straightforward process, but in recent years, this framework has nonetheless been tested in a number of studies in Canada, Norway, United Kingdom, and elsewhere. Results have shown that decision makers in health care organizations have found it a useful approach [11-14]. However, there has been little empirical research of its application at the district level in low- and middle-income countries. Few studies have documented the challenges that the approach faces in practice. The first studies that have reported on the application of AFR in low-income countries have focused on single organizations [15,16], or on assessments of priority-setting practices across countries [17,18].

There has been raised criticism of AFR conditions as not being adequate to ensure that the decision-making process will be fair, reasonable, and legitimate, as well as accepted by those of a different opinion or those adversely affected. Friedman has suggested, however, that profound popular involvement and establishing criteria for avoiding *a priori* exclusion of some values could imply an improvement in a priority-setting and decision-making context [19]. Resource managers may find that policy-related, generally-desired, non-health effects may be more important than disease or program-specific health effects [20]. Hence, there are divergent opinions as to whether general health-related or program specific and managerial arguments should be given more weight in the priority-setting process. A recent review illustrates the same dilemmas [21]. Others again have argued that AFR and a more technical priority-setting approach may be mutually supportive [22]. It has been pointed out that power differences can be seen as constraining the compliance with the four AFR conditions, and a fifth condition of empowerment [23] was proposed. However, this suggestion has not been fully incorporated in the AFR approach.

Based on existing evidence it seemed fruitful to assess the AFR approach in district health systems in African resource-poor settings with the aim to enhance existing knowledge about the relevance and usefulness of the AFR concept as well as about the implementation process and potential outcomes from diverse contexts. The study “*REsponse to ACcountable priority setting for Trust in health systems*” (REACT) commenced in 2006 through funding from the EU (under FP6 contact PL 517709). The overall objective of the project was to strengthen the legitimacy and fairness of priority-setting processes in Tanzania, Kenya, and Zambia.

We applied the AFR concept to decision-making processes at the district level in the three countries. More specifically, we sought to introduce the AFR approach in order to assess potential changes in terms of participation in priority-setting processes and potential influence of the approach on the district management, health workforce management, and, eventually, provision of services with a particular focus on the field of HIV/AIDS, malaria, emergency obstetric care, and general care.

**Figure 1:**
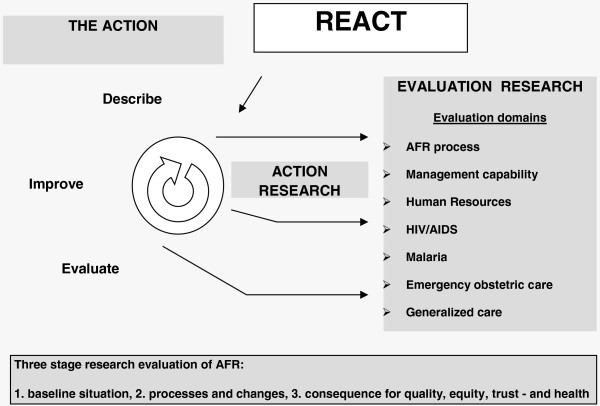
The REACT study design.

For a detailed presentation and discussion of the research objectives, design, and methods, we refer to a previous publication in this journal [24]. The research study design is shown in Figure 1.

Data and analyses for gaps in AFR conditions at baseline have been published for each of the three countries [25-27]. The methodology, detailed data description, and core results of the population-based surveys in the three countries have been published focusing on institutional childbirth that well illustrate also the chosen service assessment indicators for quality, equity, and trust in the three countries [28]. We further refer to a number of papers that present more thematic results from other baseline studies. They focus on HIV/AIDS and condom availability in all three counties [29], on malaria in Zambia [30], on emergency obstetric care in Kenya [31], on institutional childbirth in all three countries [28], on voluntary counseling and testing (VCT) for HIV infection in all three countries in Additional file 1 and [32], on perceptions on fair decision-making and limitations to fair decision-making processes in Tanzania [33].

The baseline studies found that involvement of communities and other relevant stakeholders featured prominently in the official policies, but that the existing mechanisms and processes for decision-making at district level did have substantial shortcomings in terms of participation in actual practice. In none of the three study sites were the conditions specified by the AFR approach for fair decision-making and priority-setting processes ensured [25-27]. These studies confirmed and detailed shortcomings in the respective services which seemed related to poor horizontal consensus building at service level, and thus indicated marked gaps in the practice of AFR conditions at all levels.

In this paper, we do not venture into a full discussion of the findings from the baseline studies, nor of the broader priority-setting debate, but we set out to identify strengths and weaknesses of the AFR application as further developed by the REACT project with the aim of adding to the debate on priority-setting processes within health systems and on provider and user accountability.

The paper, thus, consists of four main parts, being first the references for overall AFR-based methodology and the wealth of methodological and situational details in published baseline papers [25-33], second the further details of the applied REACT methods and the analysis and the discussion of data from the final evaluation survey, third a broadening of the discussions in relation to results from already published other thematically focused REACT process results papers, and fourth a discussion of implications for current health development policies and strategies. Conclusions finalize the paper. The subsequent appendix provides the latest version of a practical user guide of for application of AFR and the Additional file 2 for the REACT consortium, lists the researchers who have been actively participating in REACT as authors of one or more of the REACT publications referred to or as important contributors to project results.

## Methods

In the REACT project, we applied action research methodology to assess preconditions, processes, and outcomes of the application of the AFR approach in three study districts in Kenya, Tanzania, and Zambia, respectively.

### The cases

We chose the districts as cases and specifically focused on decision making at the district level and in units within which district health management teams play a central role. The study sites included Mbarali District in Tanzania, Malindi District in Kenya, and Kapiri Mposhi District in Zambia. These districts were purposively chosen because they have structures, processes, and actor configurations that emerge as typical for a district in their country. Furthermore, the districts were selected as comparable across the three countries. Being found ‘typical’ and ‘comparable’ nonetheless recognizes substantial diversity between the districts for example pertaining to the degree and type of decentralization in each country. The three countries do, however, have a similar multi-level structure (Table 1) and have comparable decision-making structures and procedures at district health teams. All three study districts are mainly rural and include one or more urban or semi-urban centers. They have a comparable burden of disease, while their populations are all larger than what the WHO recommends for a health district, ranging from 241,000 to 342,000 inhabitants. A summary comparison of the districts is shown in Table 1. Details for each country are further documented in the country-specific general AFR gap analysis publications [25-28].

**Table 1:** The three districts and their relations to country health system structures

**Levels**	**Mbarali, Tanzania**	**Malindi, Kenya**	**Kapiri Mposhi, Zambia**
**International**	Presence of many organizations and programs – both state and non-state actors		
**National**	Ministries for Health and Social Welfare and for local government	MOH (later divided into two)	MOH
**Subnational**	Cross-sectorial region, Health Zone (Health sector only)	Province – moving to a smaller unit County structure	Province
**District**	Council Health Team	District Health Team	District Health Team
	Council Health Services Board	District Health Board	District Health Board
	District Hospital Board	District Hospital Board	District Hospital Board
	Decentralization to local government, but professionally overseen by MOH (devolution)	Decentralization under MOH (deconcentration).	Decentralization under MOH (deconcentration)
**First line facility**	Health center/clinic and dispensary/health post; MOH, private for profit and not-private for profit.		
**Community**	Local structures and committees, (CSOs), NGOs, users and communities	Local structures and committees, CSOs, NGOs, users and communities	Local structures, neighborhood committees, CSOs, NGOs, users and communities

CSO, Civil Society Organization; MOH, Ministry of health.

### The baseline

The baseline studies have been published as referenced. They covered two components. The quantitative component community-level survey administered to a representative sample of 2,000 persons in each study district and the qualitative component that documented the existing decision-making processes and management practices concerning services, programs, and the health workforce, and secondly identified the informants’ perceptions and definitions of ‘fairness’ of decision-making processes. The qualitative components based on documentary reviews, in-depth interviews, focus group discussions, and observations as sources of data. Informants included representatives of the community, health workers of the first line health services, and the district hospital, health service managers, and district officials. These studies form the points of reference for the discussion and assessments of the processes and emerging outcomes from the application of AFR.

### The action research approach

We used a staged approach to the introduction of the AFR concept in each study district. The action research process started with informing the full District/Council Health Management Team (DHMT) and the formally involved stakeholders about the objectives of the project and about the content of the AFR principles and conditions. An ART was then formed in each district, including 3 to 4 senior members of the DHMT and 1 to 2 researchers from the national research partner institutions.

In a first step, initial ‘sensitization’ sessions solicited stakeholder and community views on AFR and the meanings and values these actors would associate with its four conditions. Finding in all three sites substantial concurrence between the AFR approach and the ‘local’ values, we proceeded to set up a context-adaptive continuous action research approach.

The ART met on a regular basis to facilitate, monitor, and guide the use of the AFR conditions in the district-level decision-making processes. To this end, the REACT project used a tested cyclical review process, called Describe–Evaluate–Improve (DEI) [11], aimed at stimulating the ARTs to continually evaluate the application of the AFR conditions and to address potential practical problems that emerged in the process. The DEI cycle was driven by the DHMT members (in Tanzania called council health management team (CHMT)) through processes where the researchers provided insights and advice. Guidelines for the DEI cycle were developed in a joint process by the ARTs of the three countries. The DEI guidelines were used in the teams’ annual priority-setting and planning exercises, but also in daily decision-making activities. Appendix 1 presents the latest version of the guideline. Whenever new stakeholder groups or individuals were included in the district decision-making process, they were introduced to AFR and the ongoing AFR-based process.

Beyond assessing potential change related to the AFR process, the research also aimed at generating knowledge about how AFR can be introduced. The learning process was thus also documented by the ARTs. They reported during specific AFR research meetings and during the annual REACT project meetings where discussions with all the involved researchers (and at times including senior DMHT members) from all the three countries led to the continuous adaptation of the DEI guidelines. Additionally, the regular coordination round trips made by the project coordinator, which aimed at monitoring and supporting country teams, provided opportunities to discuss the ART intervention and the DEI process. A focal person representing the researchers was agreed upon for each country in order to improve the data collection and AFR advisory support for the districts. The focal persons were selected based on being either university students or young researchers within or associated with one of the participating university departments in each study country. They started this work in the study districts in year 3 of the project. This allowed for more continuity of AFR support, but also for more in-depth observations of the ongoing process in the districts.

It should be noted that the project did not provide any monetary input to the district or to the governmental employees involved with the project at district level, except for provisions for meetings.

The project focused on health sector decision making at district, hospital, and first line facility level. We differentiated formal decision-making through planning meetings, for instance for developing the annual district plan, from the routine day-to-day decision making. We define the changes that were introduced to attitudes and practices of actors and to the organizational culture as the output of the actual AFR process. The study report is based on the analysis of the following data sources:

•Reports: the ART meeting minutes, DHMT meeting minutes, reports and minutes from meetings of joint district planning committees

•Observation reports

•Minutes of the annual round trips by the project coordinator.

•Minutes and reports of the joint meetings of the ARTs during the annual REACT project meetings and the general reports of the annual REACT project meetings

•Minutes of the monthly REACT Steering Committee telephone meetings and of its meetings during the annual project meetings

**Figure 2:**
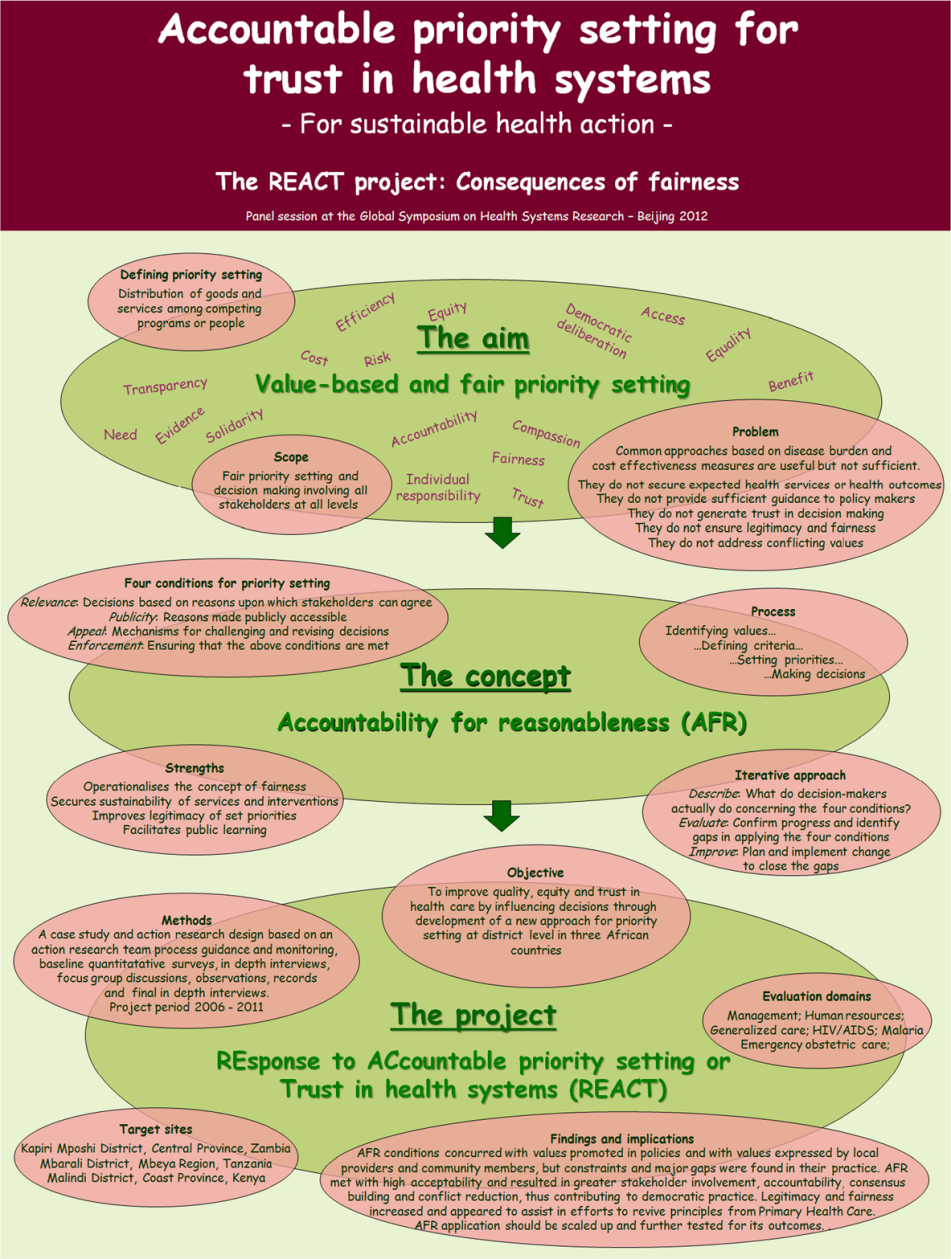
REACT project overview.

The second major data set consists of the in-depth interviews carried out at the end of the study. These focused on the actual application of the AFR conditions, on potential changes in views on and practice of decision making. A total of 54 interviews with 18 female and 36 male informants were carried out between July and August 2010. The individuals interviewed during the evaluation phase of the study were only in some cases identical with the informants interviewed during the baseline survey, but the overall characteristics of the study participants were very similar. The ones included in the evaluation study were 24 members of the DHMT, managers and program officers, 11 district and regional administrators, planning and human resource officers, 7 peripheral facilities’ staff (including one from a mission health center), 4 hospital senior staff (including one from a mission hospital), 4 NGO representatives, and 4 district health board members. Table 2 shows the respondent by district.A graphic overview of the REACT project elements, processes, and emerging output has been presented in several conferences and was recently displayed as part of a REACT project parallel session in the Global Symposium on Health Systems Research in Beijing in 2012, as shown in Figure 2.

**Table 2:** Respondent distribution by study district, organization, and gender

**Organization**	**DHMT**		**Higher authority, Manag, Admin**		**Facility**		**Hospital**		**NGO**		**Board**	
**Gender/District**	M	F	M	F	M	F	M	F	M	F	M	F
**Mbarali**	5	2	7	0	3	0	0	1	1	0	2	1
**Malindi**	4	2	1	0	1	1	0	2	0	2	0	1
**Kapiri Mposhi**	8	3	3	0	1	1	0	1	0	1	0	0
**All**	17	7	11	0	5	2	0	4	1	3	2	2

### Data analysis

All interviews were recorded, translated into English where necessary, and transcribed verbatim. The transcripts and interview notes were entered in NVIVO7, and rigorous thematic analysis was carried out by the country research teams.

### Ethical issues

Ethical clearance was obtained in the three countries. In Kenya, scientific and ethical approval was obtained from the Kenya Medical Research Institute and the National Ethical Review Committee (KEMRI SSC, number 1096). In Tanzania, research clearance was obtained from the Medical Research Coordinating Committee of the National Institute of Medical Research number (NIMR/HQ/R.8a/Vol.IX/416), and in Zambia research permission was obtained from the University of Zambia Research Ethics Committee (assurance No. FWA00000338, IRB00001131 of IOR0000774). Written informed consent was obtained from all informants. Confidentiality and anonymity of the study informants was emphasized and maintained throughout the study.

## Results

We present the results as follows: the introduction of AFR in decision-making processes, the application of the AFR conditions, and the effects of the introduction of AFR on decision making.

### The introduction of AFR in the decision-making processes

The AFR concept was introduced in all three study districts in 2006, but the intensity and timing differed between the sites.

In Mbarali (Tanzania), the preparations for application of AFR started in 2007. It became clear that repeated sensitization sessions with the stakeholders were needed. This delayed the start of the actual application of the framework. Furthermore, the ART decided to introduce the AFR process step-wise, and to gradually build up experience and expertise in handling each of the conditions. In practice, this meant that a start was made by the introduction of the relevance condition in the priority-setting process in 2008. The publicity and appeals conditions were introduced from 2010 onwards, followed by working on the full application of the leadership condition in 2011.

In Malindi (Kenya), the AFR concept was introduced to the DHMT in May 2008. All conditions of AFR were introduced in the decision-making process from 2009 onwards. A high staff turnover at senior level, including the District Medical Officer, led to irregular DHMT meetings during the first years of the project. Further turbulence resulted from the serious post-election social unrest in 2007 and 2008. The post-election split of the Ministry of Health meant that meeting routines were undermined in the Kenyan districts, which denied DHMT the opportunity for continuous application and follow-up of the AFR process except for one DHMT member and the Hospital Director, who both remained in post during the project and continued their efforts to align their own area of work to the conditions. The District Medical Officer who sanctioned the introduction of AFR continued to apply the conditions in the next posting in the regional office.

In Kapiri Mposhi (Zambia), the DHMT was introduced to AFR in 2007. The District Director of Health immediately saw the potential for district decision making. AFR application started in 2008, increasingly influencing the DHMT management. All AFR principles were acknowledged as relevant and accommodated into the existing set of organizational values. Our analysis shows that this was facilitated by the team’s shared values – values ultimately based in the Primary Health Care (PHC) strategy which had been implemented some 30 years earlier [34], and which was reflected in their motto “*provision of health services in partnership with the community*”. The AFR intervention provided the team with an additional stimulus to further apply notions of participation and transparency in their decision-making practice. Interviewees, indeed, said that the DHMT revived prior existing consultative mechanisms, such as health centre committees, as a consequence of the REACT intervention.

“*Following the dissolution of the Central Board of Health, some committees had stopped functioning and only started operating after the REACT program commenced*.” (DHMT member).

In all study districts we found that the greatest challenge was the initial difficulty to grasp and to apply the concept of AFR in daily practice. Inherent assumptions underlying AFR, e.g., the notion of no decisions being absolute right or wrong, or the potential of individuals’ taking a responsibility for choice, seemed to be new to most of those involved. Study participants noted that in this process the personal attributes of the DHMT members and especially of those in management positions had a direct impact on how AFR was adopted and implemented. A comparison of the districts processes is shown in Table 3.

**Table 3:** Comparison of the process of AFR introduction in the three districts

**Steps in establishment**	**Mbarali, Tanzania**	**Malindi, Kenya**	**Kapiri Mposhi, Zambia**
**Sensitization of stakeholders. Number of recorded sessions**	From 2007 and including community members; 6 sessions	From 2008, not going beyond the hospital team; 3 joint sessions	From 2007 including already coopted NGO and increasing to others including representatives of communities
**Effective start of application**	2008	2009	2008
**Number of ART meetings and of planning meetings referring to AFR**	Total 18 ART and 4 planning meetings. Report to CHMT. Other meetings.	Total 3 ART meetings. Report to DHMT. No record of other meetings.	Monthly meetings for AFR associated with DHMT meetings and thus also taken up in plans
**ART members regularly involved**	2 researchers, 5 CHMT members	1 researcher, 3 DHMT members	4 DHMT members. Researcher presence irregular
**Focal person/observer**	Junior scientist 2009-10	Scientist for irregular periods	Only as ad hoc visits by a researcher.

AFR, Accountability for Reasonableness; ART, Action Research Team; CHMT, Council Health Management Team; DHMT, District Health Management Team.

### Application of the AFR conditions

In this section, we make an attempt to present how each of the four conditions of AFR – relevance, publicity, appeal and revision, and leadership – were perceived and dealt with in each of the study sites.

### Putting the relevance condition into practice

The relevance condition consists of two main components: i) involvement of all relevant stakeholders and ii) identification of all values that play out in priority setting. The aim is to ensure that content of what is discussed in decision- and priority-making processes is perceived as relevant in the context where it is discussed. Our analysis shows that, within the general DEI guidelines, each ART developed its own approach.

In Mbarali, Tanzania, the ART introduced the relevance condition in 2008. Most members of the CHMT felt that the planning guidelines from the Prime Ministers’ Office, the Regional Administration and Local Government and from the Ministry of Health and Social Welfare did not allow for broad stakeholder involvement in decision-making processes. This was considered as limiting the legitimacy of the decision-making process made at district level. Drawing upon the AFR relevance condition, the CHMT made the initiative to identify women and youth representatives, as well as representatives of disadvantaged groups and people living with HIV/AIDS, and invited them to the district planning meetings. Six medical professionals from the hospital and from NGOs were also included in the process taking place in the planning meetings. It was said that this inclusion increased the legitimacy of the decisions made through better representation of diverse stakeholders, and broadened the set of values considered in the priority-setting process.

Interviewees noted that, with this inclusion, the CHMT chair did to a lesser extent dominate the decision-making process. To a greater extent the team members made the decisions jointly during the meetings. Discussing decisions made in the CHMT management meetings and communicating them onwards to relevant staff and other actors moreover led to greater transparency. One of the study participants indicated that some degree of change in the bureaucratic culture occurred during this process.

“*Contrary to the past, this time you will find the agenda in the meetings being discussed with a lot of openness.*” (CHMT member)

The analysis of meeting records, however, demonstrates that the groups representing the community were actually not invited to the planning meetings. The reason given by the CHMT members during the interviews were a perceived lack of authority to invite new stakeholders to participate in the planning meetings without the approval and funding from the District Executive Director, who is the overall local government in charge of all devoluted sectors including health. Another issue brought up during the interviews was related to the practice to pay for ‘voluntary work’; since the REACT project did not reimburse people for attending committee meetings, it was felt that people could not be invited since there was no provision for their reimbursement.

In an effort to identify views from the communities and to respond to the failure to involve more stakeholders in their meetings, in 2008, the CHMT members visited villages to collect information that would enable them to improve the priorities based on the views from the community.

In late 2010, the CHMT members revived the idea of involving specific groups from the communities in the planning meetings. The CHMT invited representatives from the communities; women, youth, elderly, disabled, and people living with HIV/AIDS were invited to participate in the preparation of the district annual budget and health plans. These community representatives were, moreover, trained in participatory planning, priority setting, and AFR, and did participate during the 2011/2012 planning session.

Concerning the second element of relevance – identification of core values – our analysis shows that the majority of the study participants from the CHMT indicate that, prior to the REACT project, CHMT decisions were largely based on priorities set by the national ‘essential health package’ with little consideration of local values. The interference from the central government and by vertical programs made local priority-setting processes difficult. The analysis shows that the involvement in the AFR process increased the awareness of CHMT members of such top down ‘interference’ in what is supposed to be locally driven processes.

“*Another challenge is that there is frequent interference from the Ministry by giving us directives and guidelines. This affects our performance and implementation of various health programs. And very often, the guidelines and directives from the Ministry come to the district level very late, and that affects the whole process of preparing district health plans. Not only that, but also the ministerial guidelines always change, and that leads to spoiling of the whole process of setting priorities*.” (Interview with a member of CHMT).

The visits of the CHMT to villages in 2008 demonstrated to the CHMT that people’s needs, preferences, and underlying values could be identified through meetings at community level. For instance, in the discussions during these visits, important issues related to service delivery at the district hospital were raised by the villagers. The knowledge generated during these visits was incorporated in the 2010–2011 Comprehensive Council Health Plan (CCHP).

“*The visit conducted in twelve villages enabled the district to solicit useful information. Members acknowledged that there were some important health needs identified by communities during the visit that did not receive enough consideration in the 2009/2010 CCHP*.” (Minutes of ART meeting)

Identified priorities included the need for construction of new health facilities, solving problems with procurement of drugs, supplies, and equipment, and shortage of health staff. Most importantly, the CHMT seemed to learn that through such a community oriented process important information may be obtained from the communities. Analysis of the interviews and documents moreover indicate that the CHMT subsequently started using local data more frequently for priority setting and in other decision-making contexts.

As mentioned above, in Malindi (Kenya), the initiation and implementation of AFR was accompanied by a number of difficulties. Against the background of many changes in the DHMT during the project period, the hospital director remained in post and became central in the introduction and application of AFR conditions within the hospital decision-making processes. A DHMT member who remained in the same post during the REACT period moreover reported to have adopted the AFR concept in decision-making processes. Both indicated that they had observed positive changes in terms of broader involvement as the result of their attempts at applying AFR conditions, however limited these would be in the larger picture. Although the use of the AFR conditions was not involving all DHMT members in Malindi, the concept was broadly perceived as acceptable and useful.

In Kapiri Mposhi (Zambia), increased stakeholder involvement was actively pursued after the initiation of the AFR intervention. More mid-level managers were involved in the decision making and management of the health services, including the management team of the Rural Health Centres and the district hospital management team. Existing cross cutting teams also including managers and staff outside the District Health Team covering district health planning, malaria control, clinical services, human resources, and mother and child health were to a greater extent involved in coordinated priority-setting processes.

“*The number has grown, … there are people from DHMT, also the hospital staff attend, the nursing sisters from health centers, representatives from neighborhood health committees, we are quite many this time. For a long time, what used to happen was that just a few officers would attend and then they would come to tell us whatever was discussed.*” (Staff, DHMT).

The AFR process was moreover extended to the health centres through the establishment of their own AFR action teams.

The study informants indicated that, as a result, decisions were now to a greater extent made collectively and not merely by one person, and that the new arrangements allowed problems in the community to be dealt with at the health center level. Interviewees also expressed the view that the sense of community ownership of the health programs increased.

“*I think the greatest thing that we have improved is community involvement, because now, we don’t just sit and agree with the district commissioner. We don’t do anything without being convinced that, actually, the community is happy about it and they have made an input in it.*” (DHMT member)

### Publicity

The second AFR condition is publicity, which implies that decision makers are to publicize their priorities and the reasons for their decisions, so that stakeholders, including the public, can understand the values involved in the choices made, and assess whether the processes decided upon are actually implemented.

The CHMT in Mbarali, Tanzania, initiated efforts to disseminate district health priorities in 2009 by starting a communication of the CCHP priorities to all its members and to the district health program managers and district hospital workers. Later, the CHMT translated the priorities from English to Kiswahili (the national language in Tanzania) and disseminated them further through the notice boards of the district hospital, the District Council Offices, the Village Council Offices, the Ward Executive Offices, and the health centers and dispensaries. The analysis of interviews and documents indicates that publicizing district health priorities saw a notable change in the management culture of the CHMT:

“*Although the existing mechanisms for publicity are ad hoc, people were impressed with the decision to make the priorities known to them. For example, some women from Isitu village indicated this when they met one of the ART members. The district hospital workers were also surprised by the decision to publicize the priorities, which was not the culture in the previous years*.” (CHMT member)

However, the CHMT members mentioned that unpredictable and late disbursement of funds from the national level was often a barrier to fully implementing the publicity condition, not primarily because this decreased operational funding for dissemination, but because of the uncertainty it induced.

“*It is very difficult to publicize priorities in advance, because we are not sure beforehand how much funds will come from the Ministry. Since we are not sure of the amount we will get, we are afraid of announcing publicly our priorities to avoid problems and complaints from the citizens after failing to implement what we announced*.” (CHMT member).

Additionally, the CHMT members recognized that the publicity mechanism did not take into account those who can neither read nor write.

In Malindi, Kenya, there were also increased efforts to publicize decisions during the project period. The District Medical Officer of Health (DMOH) launched a health service newsletter at the start of the project, an initiative later taken up by the provincial health team when the DMOH was promoted to direct its public health activities. The hospital management team reinforced its posting of decisions and priorities at notice boards.

In Kapiri Mposhi, Zambia, the DHMT increased its use of existing ways to make decisions and reasons public to the community. This included the use of drama groups, neighborhood health committees, traditional birth attendants, community meetings, information sessions at the clinics, posters, suggestion boxes, and the development committees. Increased use was also made of memos, meetings, and reports to communicate with members of staff. Our analysis shows that these efforts contributed to higher inclusion in meetings of representatives from the churches, traditional leaders, healers, community-based organizations, NGOs, and of ordinary community members.

“*As I said, there’s been a lot of publicity and, you know, program officers are actually coming up and everyone will bring their issues and you look at them now fairly.*” (Health center staff member).

In summary, we found that the strengthening of publicity efforts took place at all three study sites, and that the increased dissemination of the priority-setting activity was found to be both an acceptable and feasible practice. Our analysis indicates that in all three sites, the AFR approach made people aware that the existing channels of information sharing were inadequate, and that it for various reasons was beneficial to become more interactive and to ensure enhanced exchange between providers and users.

However, despite the efforts to improve the communication channels, the analysis showed that none of the districts systematically communicated the reasons and criteria that were used to arrive at their decisions. Actual discussion of such criteria occurred only when knowledgeable staff happened to be available at the site of posting and thus able to inform staff or other actors.

### Appeal and revision

The CHMT in Mbarali, Tanzania, took initiative to develop the appeal and revision mechanism for the district. This began with the creation of the appeal and revision procedures for the district hospital workers in early 2010, informing them through a staff meeting.

“*The CHMT developed an appeal mechanism at the district hospital level through which hospital staff would be able to voice their concerns, views, and opinions concerning not only health priorities set during planning processes but also concerning day-to-day management of the hospital.*” (Minutes of ART meeting)

However, the response to the request for comments and appeals relating to district health priorities was low. Some members reported that out of ten health facilities that publicized health priorities, only one received appeals from the community.

In Malindi, Kenya, the suggestion box was found to be a prominent feature in health facilities, but its use remained very limited. However, other improvements to communication occurred after repeated introductions of AFR.

“*I find people have kind of become free. People have been interacting freely. They have been exchanging their ideas rather freely, starting with the DHMT members themselves, even to the people in the rural health facilities. The interaction to me has been good. People have really taken into the account what others are saying and there is some consideration of what people are airing*.” (DHMT member)

However, we found that in Malindi, no further efforts were undertaken to specifically improve the modalities for appeal during the project period.

Informants in Kapiri Mposhi described how appeal channels existed before the REACT project was initiated, but they qualified that these were neither well defined nor functional. In practice, contested issues, including those that could be sorted out at local level, were usually referred to the Ministry. Analysis of documents and observations indicates that over the period of three years of the initiation of AFR, there was an increase in appeal mechanisms at all levels and in their actual use by health center staff and community representatives.

“*In the old days, once management made a decision, it was final, unlike now: if you make a decision and the decision it’s not favored by the people, they come and they try to appeal, then you revisit or probably change it. This has been a result of REACT*.” (DHMT member)

In summary, the analysis showed that the informants considered the appeal and revision mechanism to be very important. In all three districts it was felt that to allow the members of the planning team to establish a fair process for change to contested priority-setting decisions, would enable them to also defend such change to the higher authorities as well reasoned commitments made to the locally involved other stakeholders and to the communities.

### Leadership and enforcement

The AFR approach to priority setting requires that public or voluntary regulation of the decision process is put in place to ensure that the relevance, publicity, and appeal and revision conditions are met.

In Mbarali, Tanzania, the CHMT ensured enforcement. The District Medical Officer played a key role in this process. He was present during the whole project. When he was transferred towards the end of the project, his successor was introduced to the ongoing AFR process during the transition period. The incorporation of new members in the decision-making team, primarily program managers and hospital staff, was said to increase the legitimacy of the CHMT as the leader for AFR.

In Malindi, the leadership condition was hardly fulfilled. To a large degree, this was due to frequent changes in personnel in the DHMT. During the implementation period, there were three transfers of DMOH as well as of two other officers in the ART. Each transfer resulted in two to three months of waiting for a replacement, and next waiting for the new entrants to settle into required office routines before their involvement with the AFR process.

In Kapiri Mposhi, the District Director of Health was well established in his function from the onset and remained in post throughout the project period. The analysis shows that there was no formal decision taken to vest the leadership function in the DHMT, but most stakeholders appreciated the role played by the leaders at the district level in promoting AFR. The informants stated that the leaders were willing to consider or seek advice from other members of staff. Informants were generally said to have confidence in the leadership of the DHMT, because it consisted of people who were willing to involve all stakeholders, provide guidance, and generally take responsibility in situations of disagreement, while maintaining inclusiveness in decision-making processes. The management and personal leadership style of the district director was thus noted as important.

“*Apart from REACT, I think that good leadership skills and attributes in the District have contributed to improved fair priority-setting processes. REACT has just supplemented the efforts*”. (Member of provincial health team)

In several instances where decisions of the DHMT were misunderstood, the appeal procedure clarified the issue, confirmed it, or led to revision. This change was mainly attributed to applying the AFR conditions, but also to the management approach of the District Medical Officer of Health in Kapiri Mposhi. It was felt that his managerial abilities and competencies played a major role in opening up channels of appeal in the district.

In summary, effective leadership was considered by the interviewees in all three study districts as a pre-requisite for enforcement of all rules and regulations, as well as the practices required by AFR. The DHMT assumed the enforcement function by default in all cases. There was, however, little evidence of concrete mechanisms or aims for public ‘take over’ as guarantor for AFR.

### Outcomes of the introduction of AFR-guided decision-making processes

In this section, we present the findings regarding changes that seem to be related to the implementation of AFR on decision making where we focus on the type of decisions, levels addressed, and changes it seems to have triggered (see Methodology). Although we are strongly aware of the shortcomings of the intervention, and we recognize that through this research endeavor we cannot attribute changes in service output or outcome directly to AFR, we argue that certain changes in attitudes and approaches to decision-making processes seem to be strongly associated with the REACT project’s introduction of the AFR conditions. In Mbarali, Tanzania, as presented above, AFR conditions were introduced sequentially. This process was mostly applied in the formal planning cycle meetings, and was reported to have introduced a more inclusive and transparent routine for making decisions, including stimulating the CHMT to actively identify priorities at the village level. Another practical example was the issue of more speedy processes of decision making due to AFR mentioned by several health staff:

“*Another change which I have noted is washing machine. We had no washing machine at this hospital. Medical attendants used to wash clothes. We repeatedly asked for a washing machine. This issue was included in the last year’s comprehensive council health plan.*” (Senior hospital staff).

In Malindi, the AFR intervention was introduced in far more difficult circumstances, and had less chance to take off and, not surprisingly, the document review and analysis of the interviews indicate that the effects of the AFR intervention were very limited. When applied at all, it was during the formal planning meetings, but the continuity among key staff was limited, thus reducing its potential. On the personal initiative of the hospital director, the AFR principles were introduced in decision-making processes at the hospital, but its implications did not seem to trickle down to the first-line services. The interventions seemed to be too unsystematic and lack the necessary follow-up to have substantial impact upon attitudes and organizational culture, even if, as we have seen, the approach resulted in change of decision making practice by individual members in the DHMT and the hospital.

The AFR approach was most comprehensively implemented in Kapiri Mposhi. Here, the interviewees explicitly describe that priority-setting and other decision-making processes had changed as a result of the introduction of the AFR process. The AFR approach thus seems to have assisted the DHMT in better exploiting their actual decision spaces.

“*What has changed, as I said, is* [that] *we are able now to sit down as a team and make a decision together at every level, which was not the case before.* […] *It has been gradual, actually, in the last three years. It’s been gradual and even ourselves, we have just found that we can’t do certain things without involving as many people as possible*.” (District ART member)

Such noted changes are not attributable to particular AFR conditions, but are examples of improved management that, according to the DHMT itself, came about because of the practice of AFR. One might see those examples as a first outcome of the broader leadership concept inherent in the AFR process. The following may thus be seen as examples of managerial outcomes from the practice of AFR:

“*With the REACT program, you have to analyze: ‘Surely, if we are to do this, what benefit does it have to the community or how is it going to help improve maybe the, the health status of the community?’* […] *AFR talks of priority setting. Even when the resources are limited, we are able to see what could be done with the same limited resources that we have, and as I mentioned that where sometimes we have completely failed to see what we can do. That is how we came to the stakeholders and see if they can come to our aid.*” (District Health Officer)

A number of examples showed that AFR can usefully support districts in dealing with divergent interpretations and can prevent crises by resolving conflicts.

The analysis identified several critical incidents that show how, in Kapiri Mposhi, the DHMT was stimulated on the basis of AFR principles to engage with other stakeholders, and facilitated the identification and discussion of local priorities. For instance, when a major NGO, which had scaled up an anti-retroviral treatment program without formal coordination with the DHMT plans suddenly stopped its activities in the district, the DHMT acted upon it, realizing that the sudden stop would lead to a major gap in the service delivery. The DHMT sought contact with other NGOs, which jointly with the DHMT maintained the service delivery through a broader than usual redistribution of tasks, responsibilities and, thus, resource use between government and Civil Society Organization/NGO services.

Another example was presented describing how the DHMT found that several members of teams doing insecticide spraying to control malaria had not performed well. The DHMT identified unclear selection criteria and procedures as the cause, and in response developed transparent selection criteria and engaged other stakeholders in overseeing the recruitment process of the sprayers. This process ensured that all applicants (and their communities) accepted the employment decision. As a result, the teams became motivated and team performance improved.

“*We decided to be open to external stakeholders to help in the recruitment of spray providers. This helped getting the right people and, subsequently, in providing quality services to a lot of people*.” (District ART member)

A summary assessment of the AFR implementation process, focusing on the uptake and use of the AFR concept, is presented in Table 4.

**Table 4:** Summary assessment of the AFR implementation

**What worked well**	**Challenges**
AFR was regarded as a concrete and workable approach to strengthen the influence of values and context on decision making	AFR principles of legitimacy and fairness as supported by the conditionschange ways of thinking and acting which is only consolidated after a relatively long joint practice
The AFR conditions were accepted as process guidance for use of criteria for priority setting	Stakeholders, including communities, were used to be included in decision-making processes on an *ad hoc* basis, and had some trouble seeing AFR as a change from ‘business as usual’
AFR increased the stakeholder and public understanding of their opportunities to influence local health action	Action research methods were not well recognized by all involved researchers and their institutions to be as valid as other research
The AFR process guidance facilitated the coordination between current decision makers and expanded their inclusion of others in support of the implementation of national policies in local contexts	
AFR conditions influenced priority setting and other decisions in some of the sites	
**Elements facilitating the application of AFR**	**Elements constraining the application of AFR**
Fairness and other AFR-related values of transparency, accountability, and equity were already recognized as desirable aims by respondents	Concerns for managerial consequences and risks to existing agendas and power relations were likely to be the reason for a limited national and donor interest in the approach
AFR principles of inclusiveness and accountability corresponded well with existing policy guidelines and planning aims	The lack of focus on predetermined outcomes may not have been seen as a procedural support, but rather as a challenge to the strong international and national priority setting and programming
Formal structures in place for boards and committees	Limited organizational, leadership, communication, and advocacy skills may have been among reasons for poor stakeholder and public awareness of options for health action
The action research approach with continuous researcher support bridged the research into practice gap for AFR from the onset	

### Limitations

This study had a number of limitations. First, as already mentioned, the actual project duration was too short to demonstrate effects in terms of changes in the ultimate outcomes of AFR for quality, equity, and trust and for health outcomes. The late start of the application of AFR was due to the need to establish a shared interpretation of the AFR approach, and of the consequences of its introduction in the study sites as well as for the qualitative research to complete the baseline study.

Second, there is a risk that interviewees might have expressed what they believed the interviewers wanted to hear, rather than their experience with the intervention (social desirability). We attempted to reduce this risk by carefully formulating open ended questions in interview guides, and through checking concurrence of statements with process progress, records, observations, and concrete examples. Because the project did not introduce any new resources, the risk of social desirability bias seemed to have been reduced.

Third, research results from studies in several countries, which differ in their organizational and managerial set-up at district level, must be interpreted with caution. However, our purposive selection of countries and districts allowed us to have three broadly similar settings. The three study countries have differently organized health systems, but priority setting, planning, and management values and approaches were all heavily influenced by decades of support to the health sector and to governments from roughly the same group of aid agencies. This similarity is reflected in the results of the baseline studies from all three countries, which do not differ substantially in the identified gaps and expressed concerns.

Fourth, the private ‘for profit’ sector was not specifically targeted, although if private actors are stakeholders, they would be ‘captured’ by the AFR approach.

A final limitation arises from the focus on the district health team as the main unit of analysis for change of decision making processes. We chose the DHMT because we consider it as an important hub at the level of local health systems, where constructive engagement of users and their communities as well as organizational management of health services occurs.

## Discussion

This section further summarizes and reflects on the assessment of AFR in the REACT project and on opportunities for continued practice. In general, the principles of AFR were deemed relevant and useful by the district level decision makers in all three study settings. While it took time to reach a shared understanding of the terms, it was also clear that fair decision making is considered to be important by district level stakeholders.

Strengthening specific capacities including leadership and communication skills of managers, as well as knowledge and involvement of representatives on the demand side emerge as important for the implementation and consolidation of AFR. These observations concur with those made in a realist evaluation of AFR in Tanzania [35].

We also learned that it is important to fully involve user and community representatives as participants in the action research teams rather than mainly as informants. A more standardized and controlled approach, for instance coordinated by a project manager, was considered, but was also found to possibly restrict the ‘open ended’ nature of the action research. Instead the addition of a focal person in each district did strengthen the advice on AFR and improved on observation reports. The arrangement was most stable in the Tanzanian district. In the other two study districts the focal person was changed once or twice or was not as regularly available to the district team.

This study seems to add to earlier findings suggesting that the AFR concept provides a governance tool that contributes to balancing major opposed objectives such as improvement of population health and well-being on the one hand, and providing the highest possible quality response to the illness of any individual on the other. This can be said with reference to the concept that AFR contributes to identifying priorities, and attempts to enhance the fairness of the priority-setting process, not only by addressing technical, managerial, and contextual factors, but by allowing the involvement of all relevant actors and by structuring their meaningful participation in the process. It is the latter principle that gives AFR the potential to enhance perceived fairness and legitimacy of the priority-setting process, in our case, at the district level in the chosen study countries. This study showed that AFR can be applied as an ethical and democratic value-based approach to enhance mutual responsibility for health for all.

However, as we have stated, a number of challenges emerged. It took time to reach a shared understanding of the core concepts, perhaps less because of fundamental philosophical differences than operational constraints that made it difficult to see how such a new approach could be implemented. However, sustained support and the use of the DEI cycle were instrumental to meet these challenges. Initially, concerns about the feasibility of involving non-professionals in priority setting were expressed by a few DHMT members. Discussions with the ARTs addressed this concern, but when practical experiences demonstrated the feasibility and acceptability of the approach, this initial concern was overcome. We also found that while the existing priority-setting, planning, and budgeting mechanisms and policies seemed well suited for formal planning and allocation of funds, they emerged as ineffective in dealing with unanticipated changes during implementation, such as decreased or delayed resource allocation and inclusion of new programs and policy changes. The elaborate district plans thus became increasingly less realistic for guidance as the financial year progressed. This was also documented by the REACT baseline publications [25-28]. In such cases, the AFR conditions were found to provide an alternative procedure for coping with unexpected, but in reality expected change.

Involving the stakeholders – both health and non-health actors and increasingly those beyond the district health management teams in decision making influenced decisions, shared responsibility for them, and better supported their implementation. Publicizing and sharing information contributes to improved transparency, and thus to increased perceptions of fairness which seemed to emerge in a number of the examples seen in the study. The universalistic application of specific principles – as opposed to managerial decisions based on personal relations – has been shown to lead to higher levels of trust and commitment of staff [36,37]. The importance of procedural justice for trust in decision making has also been shown [38]. The involvement of stakeholders for increased mutual trust is located at the core of AFR.

The capacity and stability of the district health leadership was found to be particularly important for the enforcement function that facilitates the AFR conditions. However, the AFR leadership function may be better consolidated if delegated to reasonably permanent and committed staff and possibly other local actors. Optimally, the leadership function is to be adopted and enforced by local actors and communities who demand fair decisions and accountability to themselves. However, to transgress actual power relations (authoritative or technical insight-based) would need acceptance that communities and individuals are the experts on their own values, and that technical values only lead to sustainable progress if weighed against the social and individual values that facilitate engagement. This is relevant whether the concerned communities and the individuals involved are managers, health workers, service users, or community leaders. In an AFR process, many values and other contectual factors may remain implicit, but attempts to tease out such context in each setting may consume all the energy of change, lose sight of potential mutual benefits across groups and knowledge differences, and may prevent the AFR process from strengthening the legitimacy and thus relevance of decisions across power divides. Likewise the term empowerment has connotation of a battlefield, and may in a longer term democratic development process be better termed as development of capacity for responsive leadership, workforce communication skills, community accountability, and joint monitoring.

The health sector of a country is one of the most complex sectors and the one that most directly and regularly affects all individuals as well as being of high awareness, opinion, and concern of the whole population. Long term planning as well as daily decisions within actual rather than expected resource limits are necessary at all levels of the health system. AFR may assist in managing this complexity, and emerges as highly relevant as it is currently targeted to the health system.

The challenges of implementing AFR, as seen in the present study, indicate that fairness in priority setting may be an ideal that can be approached though probably never be fully achieved. However, unfairness can be mitigated through a continuous effort. The argument is thus related to a similar one for good governance and democratic practice, which are never fully achieved, but where efforts counter emerging undemocratic practices. State level representative democracy in various forms is recognized as indispensable for stabile development, but more debate is needed as to why or why not to more strongly apply democratic approaches at sub-national levels and in the management of fields such as health.

The implementation and adaptation of AFR in all three countries could be further facilitated by stronger involvement and support of the provincial or regional offices as well as the relevant ministries. It could be part of the process through which district administrations and political bodies, health service boards, and facility governing committees hold district health managers accountable for doing what they have agreed to do. Additionally, stakeholders should be more continually informed, and their opportunities for appeal and for influencing decisions should be improved. This would make the leadership condition less dependent on individual managerial leaders and closer to the broader enforcement condition of the AFR. Two later publications elaborate how the relevance and the application of AFR was ensured and led to better understanding of community participation in Tanzania [39], and on the challenges of involving decision makers and researchers in action research in Tanzania [40].

At the end of the project and its funding, researchers in Tanzania and Zambia made commitments to continue their involvement and support to study district AFR processes. In Tanzania, AFR was absorbed into guidance for national health research priority setting from 2006 [41] see additional file 3. Moreover, since 2009 AFR has been applied on a pilot basis in four other districts in Tanzania within the area served by the participating Zonal Resource Centre in Iringa. Training packages for AFR and for supportive capacity-building in this setting have been developed and implemented [42].

The expansion of a country knowledge base can support a continued assessment of AFR. In Kenya, a health systems research unit was formed in 2011 by REACT researchers in one institution. In all study countries, AFR-associated research is continuing within PhD studies. While capacity development and influence of both the supply and demand sides are important and should be promoted, the most important at any level would be the continuous promotion of fairness principles using an AFR approach linked to decentralization policies.

### Implications

The strategies and methods that the REACT project has employed in its application of the AFR conditions add to a vast number of other efforts to support good governance and democratic learning. AFR constitutes a framework of conditions that in principle are easy to overview and use in capacity development for democratic practice shaped by specific contexts.

The implementation of AFR in the three districts demonstrates that stakeholders are concerned with commonly shared, seemingly universal values pertaining to fairness. A broad range of globally shared values, strategic approaches, broader provider, user, and community involvement, were integrated in the PHC strategy. They were much valued by developing countries which gained increased influence on their own health systems. Both PHC and newer systems approaches have received recent attention by the WHO [43,44]. These meet new efforts at community monitoring and accountability evident through networks such as Equinet (Additional file 4) and the Community of Practitioners on Accountability and Social Action in Health (COPASAH; Additional file 5). However, many of these initiatives have difficulties in being brought to scale within national health systems. The importance of complementary roles of all stakeholders to achieve scale up of relevant, acceptable, and operationally feasible interventions has been emphasized in other studies [45]. The procedural guidance through AFR could be a new way for the national health sector to become more inclusive of such civil society initiatives. AFR might also assist in the necessary coordination with a generally fragmented ‘private for profit’ health sector.

Current global attempts of introducing equitable, Universal Health Coverage (UHC) [46] could in this context benefit from harvesting knowledge from this experience. PHC, as a defined strategy, has been misunderstood as a focus on the first contact level of services for too long to again become a sufficiently unifying term. UHC may be an answer to this if defined not as a providers’ view to health care, but as a comprehensive systems approach balancing provision by all providers with use and action by all user communities. Introducing AFR-based fair and legitimate priority-setting processes has the potential to guide UHC towards a sustainable, long-term impact for all. To this end, a concurrent health systems research and capacity development can best be achieved through lessons from various forms of action research [47]. Inspiration for action based on a focus on people and their preferences exists in several recent debates and in publications such as *When people come first*[48].

AFR is expected to contribute to good governance and democratic learning by guiding priority setting in the health sector, as this is probably the service sector which is most directly involving all individuals and is of high importance for their trust in government, private, voluntary health systems, and their relation to other sectors and to national governance. The AFR conditions are not formulated to be specific for the health sector only and could, if agreed, be modeled and tested as a practical approach to strengthen other sectors and multilevel in country democratization.

## Conclusions

The AFR concept was accepted as a tool for improved fairness and legitimacy of priority setting in health care in the three study districts. Although differences between the study districts were observed, implementing stakeholders took with the implementation of AFR greater charge of closing the gap between nationally set planning and the local realities and demands of the served communities within the resources actually received or possible to mobilize locally. Participation and contextual relevance of decisions and transparency of the priority-setting process contributed to the improvements of decision making beyond priority setting.

Supportive policies, leadership capacity, and commitment are key determinants for effective uptake of AFR. Capacity development for using the AFR conditions in decision making, including the associated communication and leadership skills, can accelerate their application. The study results imply that AFR can be applied to health systems and possibly also to other service-providing organizations and social systems. Further research should, however, be of at least 5 years duration, be based on additional adaptation of the guidance, facilitation in specific settings, on support from higher levels, and on integration of the monitoring of AFR with routine service monitoring.

## Appendix

### Application of accountability for reasonableness (AFR)

#### Guidance for the Action Research Team (ART)

1. **Relevance**

In the relevance condition, the goal is to make reasonable decisions that are inclusive, transparent, and fair. The following steps should be followed in applying this condition.

Step 5. Review the stakeholder’s involvement

Step 4. Consensus building and decision making

■ Develop list of ranked priorities;

■ Rating and scoring mechanism may assist to guide the criteria to reach the consensus;

■ Provide reasons for each identified priority.

Step 3. Start the dialogue

Stakeholders now can start the discussion focusing on identification of priorities.

During the dialogue, the following issues should be considered:

■ Provide a statement of rationale for each decision;

■ The discussion should focus on values of the stakeholders involved in the decision-making process;

■ To ensure the reliability, validity, and completeness of the data made available and presented in priority setting;

■ To capture values of each stakeholder;

■ Criteria should be developed for each identified priority.

Step 2. Clarification of decision-making procedures

■ Decision-makers and stakeholders need to know and understand

a) how decisions will be made;

b) on what basis will these decisions be made.

Step 1. Stakeholder identification

Identify your stakeholders and include them in decision-making in order to ensure that decision-making includes a broad range of ideas and stakeholder perspectives.

This can increase gradually from a start of the conditions within your own team to increasingly involve others aiming at good representation of communities and their representatives.

2. **Publicizing the priorities**

It is important to disseminate the decisions on the priorities identified and their reasons in order to legitimate them to the public. Publicizing of the priorities can be conducted through the following steps:

■ Decide who is the target group;

■ Identify the ways/methods by which the decided priorities will be disseminated (e.g., letters, notice boards, meetings, newsletters, etc.);

■ Disseminate the priorities and their reasons.

3. **Appeals/revision**

Some things to consider:

■ Depending on the subject in question, appeals may come from different groups including:

■ Appeals can be directed towards health planning priorities or daily management decisions;

■ Stakeholders should be given enough time to appeal and feedback should be given promptly.

1. health workers;

2. community, etc.

Actions to be taken

■ Set up a committee responsible for receiving and compiling appeals to be submitted to the District Health Management Team (DHMT);

■ Set up ways for appeals in different situations for stakeholders, e.g., through meetings, letters, face-to-face conversation, etc.;

■ DHMT to process and discuss appeals for decisions on whether to reject, accept, or revise;

■ DHMT to give reasons for accepting or rejecting appeals and revising decisions;

■ Set up mechanisms for providing feedback to stakeholders. Feedback can be provided through meetings, letters, face-to-face conversation, etc.;

■ DHMT to ensure that the process of feedback provision does not exceed one month from receiving appeals.

4. **Leadership**

To spearhead and drive the process, a team comprised of the District Medical Officer (DMO) and another three members from the Health Management Team should be formed to lead the application of AFR in the district.

(i) The team leader (DMO) should ensure the following:

■ Meetings are transparent and fair;

■ The members have equal rights;

■ Roles and responsibility of the members are known;

■ There is a mechanism for reaching consensus during decision-making process.

(ii) The criteria for selecting Action Research Team (ART) members from the district:

■ They must understand the application of four conditions of AFR and the Describe–Evaluate–Improve (DEI) process;

■ They must have interest in improving health systems through AFR implementation;

■ They must have a mandate to bring change in the district;

■ Committed members;

■ Consider issue of gender.

(iii) Terms of reference for the ART

■ Create awareness by conducting capacity building on AFR concepts to health staff, community, and other stakeholders;

■ Facilitate the application, implementation, and sustainability of AFR intervention in the district;

■ Monitor progress of the process in the implementation of AFR conditions.

(iv) Other issues to be considered

■ The ART should meet every second month with an agenda using a discussion guide, i.e., DEI approach;

■ The ART should report to all other health management team members on a quarterly basis;

■ The ART should promote AFR in other meetings as well, e.g., DHMT and others.

(v) Agenda for conducting ART meetings

The mandate for the meeting is on the implementation of the four conditions of AFR

3. Improving

Way forward

New action points

••••AOB

••••Closing of the meeting

2. Evaluating

Lessons learnt

Opportunities for improvement

1. Describing

Good practices

Challenges

■ Opening of the meeting;

■ Approval of the agenda;

■ Confirmation of the previous meeting;

■ Matters arising using DEI approach.

### The DEI process was applied for AFR conditions in ART and in some other meetings

1. Describe

Ensure a focus on values as the starting point for dialogue. It is the value base that determines the criteria used, the information needed, the priorities set, and, finally, the decisions made

■ What decision makers and priority-setting members actually do concerning the four conditions (relevance, publicity, appeals/revision and leadership);

■ Review/revise current priorities in relation to values and criteria;

■ Align priorities with the values and criteria and make them public;

■ Management to invite and respond to comments and appeals to priorities

2. Evaluate

How successfully the decision-making process met the conditions of ‘Accountability for Reasonableness’. The gaps between ‘what you do’ and ‘what you should be doing’ must be identified. To close this gap, you need to be able to evaluate your success.

■ Confirm progress in applying AFR conditions for priority setting;

■ Identify gaps in application of AFR conditions;

■ Opportunities for improvement;

■ Other lessons learnt.

3. Improve

The decision-making process to make it more ethical, the gaps you identify are areas of improvement for subsequent iterations of decision-making.

■ Plan and implement change to close the gaps in AFR during priority setting;

■ Develop new action points in relation to AFR concept.

## Abbreviations

AFR: Accountability for Reasonableness; ART: Action Research Team; CHMT: Council Health Management Team; CCHP: Comprehensive Council Health Plan; DEI: Describe–Evaluate–Improve; DHMT: District Health Management Team; DMOH: District Medical Officers of Health; NGO: Non-Governmental Organization; PHC: Primary Health Care; REACT: REsponse to ACccountable priority setting for Trust in health systems; UHC: Universal health coverage.

## Competing interests

The authors declare that they have no competing interests.

## Authors’ contributions

JB conceived the study and developed its main methods with ØO, coordinated the study implementation, participated in the intervention, data collection and analysis, and drafted the manuscript. BM contributed to methods development, monitored data use, participated in data analysis, and provided major contributions to the manuscript. SM participated in data collection and analysis in Tanzania and contributed to the manuscript. JZ participated in data analysis in Zambia and contributed to the manuscript. SB participated in data collection and analysis in Kenya and contributed to the manuscript. AKH contributed to methods development, managed data, and contributed to the manuscript. AB contributed to methods development, data analysis, and the manuscript. PK facilitated the intervention, led Tanzanian teams, participated in data analysis, and contributed to the manuscript. CM facilitated the intervention, led Zambian field teams, participated in data analysis, and contributed to the manuscript. LN facilitated the intervention, collected data, participated in their analysis, and contributed to the manuscript. BN facilitated the intervention, collected process data, assisted in their analysis, and contributed to the manuscript. PB assisted in methods development, managed data, and contributed to the manuscript. ØO conceived the study and developed methods, participated in data analysis, and contributed to the manuscript. All authors read and approved the final manuscript.

## Supplementary Material

Additional file 1 **Njeru MK.** HIV testing services in Kenya, Tanzania and Zambia: Determinants, experiences and responsiveness. PhD Dissertation. Click here for file

Additional file 2 The REACT Consortium. Click here for file

Additional file 3 Tanzania National Health Research Priorities, 2006-2010. Click here for file

Additional file 4 Equinet. Click here for file

Additional file 5 Community of Practitioners on Accountability and Social Action for Health - COPASAH. Click here for file

## References

[B1] Martin D Singer PA A strategy to improve priority setting in health care institutions Health Care Anal 2000 11 1 59 68 14510309 10.1023/A:1025338013629

[B2] Murray CJL Lopez A Murray CJL, Lopez AD Quantifying the burden of disease and injury attributable to ten major risk factors The Global Burden of Disease: A Comprehensive Assessment of Mortality and Disability from Diseases, Injuries, and Risk Factors in 1990 and Projected to 2020 1996 Cambridge, MA: Harvard University Press

[B3] Hoedemaekkers R Dekkers W Justice and solidarity in priority setting in health care Health Care Anal 2003 11 4 325 343 14769014 10.1023/B:HCAN.0000010061.71961.87

[B4] Maundy E Kapiriri L Norheim OF Combining evidence and values in priority setting: testing the balance sheet method in a low-income country BMC Health Serv Res 2007 7 152 10.1186/1472-6963-7-152 17892561 PMC2096625

[B5] Mshana S Shemilu H Ndawi B Momburi R Olsen OE Byskov J Martin DK What do district health planners in Tanzania think about improving priority setting using ‘accountability for Reasonableness’? BMC Health Serv Res 2007 7 180 10.1186/1472-6963-7-180 17997824 PMC2151948

[B6] Olsen ØE Ndeki S Norheim OF Human resources for emergency obstetric care in northern Tanzania: distribution of quantity or quality? Hum Resour Health 2005 3 5 10.1186/1478-4491-3-5 16053519 PMC1199615

[B7] Daniels N Sabin J Limits to health care: fair procedures, democratic deliberation, and the legitimacy problem for ensurers Philosophy Public Affairs 1997 26 4 303 350 10.1111/j.1088-4963.1997.tb00082.x 11660435

[B8] Daniels N Sabin J The ethics of accountability in managed care reform Health Affairs (Millwood) 1998 17 50 64 10.1377/hlthaff.17.5.50 9769571

[B9] Daniels N Sabin J Setting Limits Fairly: Can we Learn to Share Medical Resources? 2002 New York: Oxford University Press

[B10] Daniels N Just Health: Meeting Health Needs Fairly 2008 Cambridge: Cambridge University Press

[B11] Martin DK Giacomini M Singer PA Fairness, accountability for reasonableness, and the views of priority setting decision-makers Health Policy 2002 61 279 290 10.1016/S0168-8510(01)00237-8 12098521

[B12] Martin DK Reeleder D Keresztes C Singer PA What do hospital decision makers in Ontario, Canada, have to say about their fairness of priority setting in their institutions? BMC Health Serv Res 2005 5 8 10.1186/1472-6963-5-8 15663792 PMC548272

[B13] Walton NA Martin DK Peter EH Pingle DM Singer PA Priority setting and cardiac surgery: a qualitative case study Health Policy 2007 80 3 444 458 10.1016/j.healthpol.2006.05.004 16757057

[B14] Jansson S Implementing accountability for reasonableness-the case of pharmaceutical reimbursement in Sweden Health Economics Policy Law 2007 2 153 171 10.1017/S1744133107004082 18634660

[B15] Kapiriri L Martin DK Priority setting in developing countries health care institutions: the case of a Ugandan hospital BMC Health Serv Res 2006 6 127 10.1186/1472-6963-6-127 17026761 PMC1609114

[B16] Kapiriri L Martin DK Bedsides rationing by health practitioners in a context of extreme resource constraints: the case of Uganda Med Decis Mak 2007 27 44 52 10.1177/0272989X06297397 17237452

[B17] Johansson KA Jerene D Norheim OF National HIV treatment guidelines in Tanzania and Ethiopia: are they legitimate rationing tools? J Med Ethics 2008 34 478 483 10.1136/jme.2007.021329 18511624

[B18] Kapiriri L Norheim F Martin D Priority setting at the micro-, meso- and macro-levels in Canada, Norway and Uganda Health Policy 2007 82 1 78 94 10.1016/j.healthpol.2006.09.001 17034898

[B19] Friedman A Beyond accountability for reasonableness Bioethics 2008 22 101 112 10.1111/j.1467-8519.2007.00605.x 18251770

[B20] Lippert-Rasmussen K Lauridsen S Justice and the allocation of healthcare resources: should indirect, non-health effects count? Med Healthcare Philosophy 2010 13 3 237 246 10.1007/s11019-010-9240-9 20306307

[B21] Hipgrave DB Alderman KB Anderson I Soto EJ Health sector priority setting at meso-level in lower and middle income countries: lessons learned, available options and suggested steps Soc Sci Med 2014 102 190e200 24565157 10.1016/j.socscimed.2013.11.056

[B22] Baltussen R Mikkelsen E Tromp N Hurtig A-K Byskov J Olsen OE Bærøe K Hontelez JA Singh J Norheim OF Balancing efficiency, equity and feasibility of HIV treatment in South Africa – development of programmatic guidance Cost Effect Res Allocation 2013 11 26 10.1186/1478-7547-11-26 PMC3851565 24107435

[B23] Gibson JL Martin DK Singer PA Priority setting in hospitals: fairness, inclusiveness, and the problem of institutional power differences Soc Sci Med 2005 61 2355 2362 10.1016/j.socscimed.2005.04.037 15950347

[B24] Byskov J Bloch P Blystad A Hurtig A-K Fylkesnes K Kamuzora P Kombe Y Kvåle G Marchal B Martin DK Michelo C Ndawi B Ngulube TJ Nyamongo I Olsen ØE Onyango-Ouma W Sandøy IF Shayo EH Silwamba G Songstad NG Tuba M Accountable priority setting for trust in health systems – the need for research into a new approach for strengthening sustainable health action in developing countries Health Res Policy Systems 2009 7 7 10.1186/1478-4505-7-7 PMC2777144 19852834

[B25] Bukachi SA Onyango-Ouma W Siso JM Nyamongo IK Mutai JK Hurtig A-K Olsen ØE Byskov J Healthcare priority setting in Kenya: a gap analysis applying the accountability for reasonableness framework Int J Health Plann Manag 2013 In press 10.1002/hpm.2197 23775594

[B26] Zulu JM Michelo C Msoni C Hurtig A-K Byskov J Blystad A Increased fairness in priority setting processes within the health sector: the case of Kapiri-Mposhi District, Zambia BMC Health Serv Res 2014 14 75 10.1186/1472-6963-14-75 24548767 PMC3932790

[B27] Maluka S Kamuzora P San Sebastiån M Byskov J Olsen ØE Shayo E Ndawi B Hurtig AK Decentralized health care priority-setting in Tanzania: evaluating against the accountability for reasonableness framework Soc Sci Med 2010 71 4 751 759 10.1016/j.socscimed.2010.04.035 20554365

[B28] Ng’anjo Phiri S Kiserud T Kvåle G Byskov J Evjen-Olsen B Michelo C Echoka E Fylkesnes K Factors associated with health facility childbirth in districts of Kenya, Tanzania and Zambia: a population based survey BMC Pregnancy Childbirth 2014 14 219 10.1186/1471-2393-14-219 24996456 PMC4094404

[B29] Sandøy IF Blystad A Shayo EH Maundy E Michelo C Zulu J Byskov J Condom availability in high risk places and condom use: a study at district level in Kenya, Tanzania and Zambia BMC Public Health 2012 12 1030 10.1186/1471-2458-12-1030 23181969 PMC3533956

[B30] Tuba M Sandoy IF Bloch P Byskov J Fairness and legitimacy of decisions during delivery of malaria services and ITN interventions in Zambia Malar J 2010 9 309 10.1186/1475-2875-9-309 21040552 PMC2988042

[B31] Echoka E Kombe Y Dubourg D Makokha A Evjen-Olsen B Mwangi M Byskov J Olsen OE Mutisya R Existence and functionality of emergency obstetric care services at district level in Kenya: theoretical coverage versus reality BMC Health Serv Res 2013 13 113 10.1186/1472-6963-13-113 23522087 PMC3616893

[B32] Njeru MK Blystad A Shayo EH Nyamongo IK Fylkesnes K Practicing provider-initiated HIV testing in high prevalence settings: Consent concerns and missed preventive opportunities BMC Health Services Research 2011 11 87 doi:10.1186/1472-6963-11-87. Also included and supplemented in: Njeru MK: Determinants, Experiences and Responsiveness of HIV Testing Services in Kenya, Tanzania and Zambia. PhD Thesis. University in Bergen; 2011 [https://bora.uib.no/bitstream/handle/1956/4837/Dr.thesis_Mercy%20K.%1666%2020Njeru.pdf;jsessionid=F5CC2289DA52585E7CB06E31CC161E5B.bora-uib_worker?sequence=1] 10.1186/1472-6963-11-87 21507273 PMC3105945

[B33] Shayo EH Norheim OF Mboera LEG Byskov J Maluka S Kamuzora P Blystad A Challenges to fair decision-making processes in the context of health careservices: a qualitative assessment from Tanzania Int J Equity Health 2012 11 30 10.1186/1475-9276-11-30 22676204 PMC3476442

[B34] WHO Declaration of Alma-Ata International Conference on Primary Health Care 1978 USSR: Alma-Ata

[B35] Maluka S Kamuzora P San Sebastian M Byskov J Ndawi B Olsen ØE Hurtig A-K Implementing accountability for reasonableness framework at district level in Tanzania: a realist evaluation Implement Sci 2011 6 11 10.1186/1748-5908-6-11 21310021 PMC3041695

[B36] Pearce J Branyiczki I Bigley G Insufficient bureaucracy: trust and commitment in particularistic organisations Organ Sci 2000 11 148 162 10.1287/orsc.11.2.148.12508

[B37] Gould-Williams J The importance of HR practices and workplace trust in achieving superior performance: a study of public sector institutions Int J Human Res Manag 2003 1 28 54

[B38] Gilson L Palmer N Schneider H Trust and health worker performance: exploring a conceptual framework using South African evidence Soc Sci Med 2005 61 7 1418 1429 10.1016/j.socscimed.2004.11.062 16005777

[B39] Kamuzora P Maluka S Ndawi B Byskov J Hurtig A-K Promoting community participation in priority setting in district health systems: experiences from Mbarali district, Tanzania Glob Health Action 2013 6 22669 24280341 10.3402/gha.v6i0.22669 PMC3841300

[B40] Maluka S Kamuzora P Ndawi B Hurtig A-K Involving decision-makers in the research process: challenges of implementing the accountability for reasonableness approach to priority setting at the district level in Tanzania Glob Public Health 2014 9 7 760 772 10.1080/17441692.2014.922208 24921238

[B41] National Institute for Medical Research Tanzania Health Research Priorities, 2006-2010 2006 Tanzania: NIMR [http://www.nimr.or.tz/wp-content/uploads/2013/07/TANZANIA-HEALTH-RESEARCH-PRIORITIES-2006.pdf]

[B42] Primary Health Care Institute AFR Training Packages 2010 Iringa, Tanzania: PHCI

[B43] WHO The World Health Report 2008: Primary Health Care (Now More Than Ever) 2008 Geneva: WHO 2008

[B44] WHO Systems Thinking for Health Systems Strengthening 2009 WHO: Geneva

[B45] Milat AJ King L Newson R Wolfenden L Rissel C Bauman A Redman S Increasing the scale and adoption of population health interventions: experiences and perspectives of policy makers, practitioners, and researchers Health Res Policy Systems 2014 12 18 10.1186/1478-4505-12-18 PMC3996855 24735455

[B46] WHO The World Health Report: Health Systems Financing: The Path to Universal Coverage 2010 Geneva: WHO 10.2471/BLT.10.078741 PMC2878164 20539847

[B47] Loewenson R Flores W Shukla A Kagis M Baba A Ryklief A Mbwili-Muleya C Kakde D Raising the profile of participatory action research at the 2010 global symposium on health systems research MEDICC Rev 2011 13 3 35 38 10.1590/S1555-79602011000300008 21778957

[B48] Biehl S Petryna A When People Come First, Critical Studies in Global Health 2013 Princeton, NJ: Princeton University Press

