# Supplementary material for: The accountability for reasonableness approach to guide priority setting in health systems within limited resources – findings from action research at district level in Kenya, Tanzania, and Zambia
Source: Health Res Policy Syst. 2014 Aug 20;12:49. doi: 10.1186/1478-4505-12-49 (PMC4237792; doi:10.1186/1478-4505-12-49)
Supplement: Additional file 1 — Njeru MK. HIV testing services in Kenya, Tanzania and Zambia: Determinants, experiences and responsiveness. PhD Dissertation. [file 1478-4505-12-49-S1.pdf]

HIV testing services in Kenya, Tanzania and Zambia: Determinants, experiences and responsiveness

Mercy Karimi Njeru

Dissertation for the degree of Philosophiae Doctor (PhD)
at the University of Bergen, Norway
2011

DEDICATED TO

My beloved: Parents, Sisters, Brothers and Teachers.

TABLE OF CONTENTS

ACKNOWLEDGEMENTS **4**

LIST OF ABBREVIATIONS **7**

LIST OF PUBLICATIONS **8**

ABSTRACT **9**

1 INTRODUCTION **11**

1.1 Global overview of the HIV/AIDS situation 11
1.2 A brief history on HIV/AIDS 11
1.3 HIV/AIDS in Kenya, Tanzania and Zambia 17
1.4 Health system values in the context of HIV testing 18
1.5 Problem statement 20

2 STUDY OBJECTIVES **21**

2.1 Overall objective 21
2.2 Specific objectives 21

3 MATERIAL AND METHODS **22**

3.1 REACT Project 22
3.2 Study area 22
3.3 Study design and rationale 24
3.4 Data collection methods applied 27
3.5 Quantitative Methods 28
3.6 Statistical analysis and the applied behavioural Model 29
3.7 Qualitative Methods 30
3.8 Qualitative data analysis 38
3.9 Ethical Clearance 42

4 RESULTS **44**

4.1 PAPER I: 44
4.2 PAPER II: 45
4.3 PAPER III: 47

5 DISCUSSION **48**

5.1 Discussion of Methods 48
5.2 Discussion of Main findings 57

6 CONCLUSIONS **65**

7 IMPLICATIONS FOR POLICY AND RESEARCH **66**

7.1 Policy considerations 66
7.2 Future research recommendations 67

8 REFERENCES **68**

9 ORIGINAL PAPERS PAPER I – III **83**

10 APPENDICES **148**

Acknowledgements

First and foremost, I thank God for blessing me with this opportunity and for the strength, motivation, endurance, commitment and drive to pursue and complete my doctoral degree. Many people have been involved in special ways in contributing to the completion of this work. I would like to express my heartfelt thanks to you all. It is not possible to give an exhaustive list of all those who have been involved. Those who may not be personally acknowledged please accept my sincere apologies.

I wish to thank our study respondents, participants and informants in the three countries (Kenya, Tanzania and Zambia) who willingly provided information that was used in this study. I thank them for teaching me through their responses and for the many discussions engaged in during the field work. Without you, this study would not have been possible.

My deepest gratitude goes to my wonderful supervisors: Professors Knut Fylkesnes (main supervisor), Astrid Blystad (co-supervisor) and Isaac Nyamongo (co-supervisor) for their continuous support, contribution towards my scientific orientation, constructive comments, inspiration, insightful feedback and advice that has given me confidence to write and present the findings of this study. I wish to express my utmost appreciation to Knut Fylkesnes. As my main supervisor your advice, encouragement and optimism kept me inspired to work harder. Astrid Blystad I am grateful for your tireless support, enthusiasm and the attention to detail that you gave to the write-ups. I wish to acknowledge the support and motivation provided by Isaac Nyamongo all through the study process.

I take this opportunity to thank the Norwegian government through the Quota scholarship program for financial support. I extend my sincere gratitude to the University of Bergen and the Centre for International Health (CIH) for providing the opportunity to study. I am highly indebted to the CIH directors of my course: Professors Bernt Lindtjørn and Rune Nilsen for facilitating a working environment that enabled me to complete this work. What is more, the rare opportunity at the Centre for International Health provided a platform for me to interact on an academic and social basis with students from many different nationalities and academic backgrounds. It is indeed an opportunity of a lifetime that has exposed my mind to different cultures and ways of reasoning whilst pursuing my studies. Thank you all!

I wish to thank the EU funded REACT project which provided an avenue for me to undertake this study, and for providing part of the data used. I wish to thank Dr. Jens Byskov (REACT coordinator) and the entire REACT family for the constant encouragement and follow-up on the study progress. Special thanks go to Professor Gunnar Kvåle for facilitating my studies in Bergen as the REACT country coordinator and to Dr. Bjørg Evjen-Olsen for encouraging me to take up this study and the friendship you offered me.

I thank the Kenyan government through the Kenya Medical Research Institute (KEMRI) - Centre for Public Health Research (CPHR) for making it possible for me to engage in this study while still employed. In this regard, I wish to extend my sincere appreciation to the director of KEMRI, Dr. Solomon Mpoke, Dr Yeri Kombe (director CPHR), and Mr Harun Owuor (head administrator CPHR) for the support and motivation to take up this study. To my colleagues in KEMRI (Lillian Nyandieka, Moses Mwangi, James Muttunga, Joseph Mutai, Titus Kitaka, Getrude Waiharo, Anasiah Mbaga and Jane Mukami) I am grateful for your support; some of you also assisted in data collection, data entry, transcriptions and translations.

I extend my heartfelt appreciation to the administrative team at CIH and student services for promptly attending to all the administrative needs during my study period: Borgny Lavik, Solfrid Vikøren, Ingvild Hope, Linda Forshaw, Unni Kvernhusvik and Ana Veronica Cordova.

I am deeply grateful to my parents, brothers, sisters, nephew and niece for their tireless support, advice, encouragement and prayers. My loving father (Dominic Njeru) and mother (Anna Njeru), nothing can repay the great love, care and education you have given me. You have both struggled to provide our needs and education. I am truly indebted to you. To my sisters, Kaari and Makena and brothers Kimathi and Mwenda, you are wonderful. Thank you for your prayers and encouragement during the entire study process. I am lucky to have you in my life. My sister Kaari thanks for reading my thesis. I also wish to thank my brother-in-law, Professor Magambo, for motivating and encouraging me to engage in this study and to pursue a career in research. To Fabrice, the care, support, advice and friendship you have offered me all through this process is priceless; thank you. To my very good friend Beatrice Wassuna thank you for the motivation and for staying in touch all through the study process.

I would also like to thank my colleagues and friends who supported me academically and offered much needed friendship. Alemnesh Mirkuzie for the inspiring conversations in the office we shared, for reading my draft thesis and for constructive comments. Charles Michelo for your professional advice and the push to complete my studies; I needed it! Lumbwe Chola, Marte Jürgensen and Elizabeth Shayo for the fruitful discussions, the great team work and inspiring chats. I extend my utmost appreciation to members of the Global Health Research Group and the HIV Research Group for the opportunity to present my study findings and for your constructive feedback which strengthened my papers.

My extended thanks go to my friends in Norway who made my life interesting and exciting. I am grateful for the great times we have spent together. You have been an inspiration to me. My special thanks go to Angelina Sijaona, Catherine Kahabuka, Ingvild Sandoy, Amal Elamin, Karen Marie Moland, Nils Gunnar Songstad, Nkomba Kayeyi, Mpundu Makasa, Rahel Manumbu, Mari Skar Manger, Maria Mathisen, Ferda Gülcan, Jørund Nygård, Robert Byamugisha, Lars Thore Fadnes, Sally Tayeb, and Jonas Bengtsen.

To my Kenyan friends in Norway, thank you for bringing home close to me. I shall always cherish the good times we have shared together. My extended appreciation goes to Loren Munyuthe, Vincent Mrimba, Judy Ologi, Matanda Dennis, and Gabriel Oguda.

List of Abbreviations

AIDS	Acquired immunodeficiency syndrome
AFR	Accountability for reasonableness
ANC	Antenatal clinic
ART	Antiretroviral therapy
ARV	Antiretroviral
CBO	Community based organisation
CDC	Centre for disease control
CI	Confidence interval
FBS	Facility based study
FGD	Focus group discussion
GOK	Government of Kenya
HCT	HIV counselling and testing
HIV	Human immunodeficiency virus
IDI	In-depth interview
IEC	Information education and communication
KEMRI	Kenya Medical Research Institute
NGO	Non-governmental Organizations
PITC	provider initiated testing and counselling
PBS	Population based survey
PMTCT	Prevention of mother to child transmission
REACT	REsponse to ACcountable priority-setting for trust in health systems
SEA	Standard enumeration area
UNAIDS	Joint United Nations Programme on HIV/AIDS
VCT	Voluntary counselling and testing
WHO	World Health Organization

List of publications

- *Paper I*: Njeru MK, Jurgensen M, Chola L, Michelo C, Byskov J, Muttunga J, Kesheni S, Fylkesnes K: **Inequalities in voluntary counselling and testing exposure: results from population based surveys in Kenya, Tanzania and Zambia.** Submitted January 2011.

- *Paper II:* Njeru MK, Blystad A, Shayo EH, Nyamongo IK, Fylkesnes K: **Practicing provider initiated HIV testing in high prevalence settings: Consent concerns and missed preventive opportunities.** *BMC Health Service Research* 2011*,* 11(87).

- *Paper III:* Njeru MK, Blystad A, Nyamongo IK, Fylkesnes K: **A critical assessment of the WHO responsiveness tool: lessons from voluntary HIV testing and counselling services in Kenya**. *BMC Health Service Research* 2009, **9**(243).

Abstract

Introduction

HIV/AIDS has been one of the most challenging pandemics in health and development. Sub-Saharan Africa remains the most affected region and it handles over two-thirds of the individuals infected world wide. A large number of interventions have been implemented to control the infection. HIV testing is one of these interventions, and is a key entry point for both prevention and treatment. HIV testing has mainly been offered through the client initiated, voluntary counselling and testing (VCT) services. However, low use of VCT has been reported in several studies despite substantial scale-up during the past 10-15 years. Provider-initiated testing and counselling (PITC) models have been introduced to increase the test rates in the context of the growing availability of treatment. Nevertheless, little has been documented on experiences with the PITC model. This study sought to assess exposure to HIV testing through VCT and the prevention of mother to child transmission (PMTCT) based testing services that practice PITC at antenatal clinics, investigate determinants of VCT use, explore experiences and perceptions with the PITC model within PMTCT program and assess the applicability of the responsiveness concept in the evaluation of VCT.

Methods

The thesis comprises of: 1) a cross-sectional study of 5689 respondents in three African districts; 2) a concurrent triangulation mixed-methods study that utilised data from: a population-based survey in three study districts, 34 focus group discussions and 18 in-depth interviews; and 3) a concurrent nested mixed-methods study applied in one of the study districts among 328 VCT users and 36 VCT counsellors.

Results

The findings indicate that education attainment and stigma were significantly associated with VCT use across the three districts. Women were much more likely to test for HIV than men in the two districts with seemingly higher use of PMTCT. Only minor gender differences appeared for VCT use. PMTCT-based HIV testing was not always accompanied by pre-test counselling and limited post-test counselling was experienced. In settings where the PITC model had been scaled up extensively through the PMTCT program, informants expressed frustration related to their experienced inability to ‘opt-out’ or decline from the provider-initiated HIV testing services. There was an experienced additional burden on women testing

through the PMTCT program as they were encouraged to recruit their spouses to go for HIV testing. The elements proposed by WHO to measure responsiveness were highly valued in a VCT context. However, qualitative findings revealed pertinent aspects of the elements (e.g. confidentiality and autonomy) that were experienced as crucial in the local setting, but had not been captured by the tool.

Conclusion

Variations in HIV testing exposure was largely related to the extent to which the PITC model had been scaled up through the PMTCT program in the three districts. Determinants of VCT use differed less by district. Education attainment and HIV stigma were dominant factors related to HIV testing across the three districts. This underscores the need to improve efforts to promote education and effective anti-stigma programs to reduce inequalities in HIV testing. Variation in exposure to overall HIV testing in the study populations can mainly be explained by the extent to which the PITC model had been scaled up through the PMTCT programme. However, scaling up of HIV testing at PMTCT settings seem to reach mainly pregnant women. Whereas this strategy seems beneficial in increasing the test levels, the manner in which the PITC strategy is currently implemented raises serious ethical concerns; the removal of the pre-test component of counselling leaves no room for obtaining informed consent; and the approach implies missed preventive opportunities that are inherent in the post-test counselling concept. Responsiveness provides a fruitful concept to evaluate HIV testing services; however, the WHO responsiveness tool needs substantial revision in order to capture elements of experiences relevance for the local context in which it is employed.

1 Introduction

1.1 Global overview of the HIV/AIDS situation

Human Immunodeficiency Virus (HIV) attacks the immune system that is vital in protecting the body from life-threatening opportunistic infections. HIV/AIDS is a global challenge that poses a threat to the health of individuals, public health and development [1, 2]. Since the discovery of the virus, ~60 million people have been infected of whom 25 million are reported dead. Currently 33.3 million people are living with the virus [2, 3]. In 2009, an estimated 1.8 million people worldwide died of AIDS, and 2.6 million new infections were reported globally [2].

Sub-Saharan Africa remains the most affected region by this pandemic and it harbours over two-thirds of those living with the virus (22.5 million). This region recorded 1.3 million deaths in 2009, accounting for almost three-quarters of all HIV related deaths globally. In the same year, 1.9 million new infections occurred in the region, a figure that is more than half the number of new infections worldwide. However, HIV incidence has been reported to be declining or stabilising in 22 countries in sub-Saharan Africa. This decline has particularly been associated with reductions in risk behaviour [2].

1.2 A brief history on HIV/AIDS

In the early 1980's AIDS was classified as a rare type of pneumonia until 1982 when Centre for Disease Control (CDC) established the term Acquired Immune Deficiency Syndrome (AIDS) [4, 5]. The causes to AIDS were not known at that time. It was hypothesised that the virus only affected gay men since it was first presented by men within that population [4, 6]. It was then also referred to as the "gay disease". This hypothesis was short-lived as reports on AIDS cases emerged from different populations throughout the 1980s [7, 8]. Although there are many ways that HIV can be transmitted (e.g. parenteral exposure, peri-natal from mother to child), heterosexual transmission is the most predominant route.

Over the years, stigma and fear were some of the dominating negative factors that surrounded those infected, possibly due to the manner in which the disease is acquired / transmitted, the absence of a cure and social exclusion aspects. To quite some extent this stigma related

scenario has remained to date. In addition to these negative challenges, death rates made the epidemic the leading cause of death worldwide in the ages between 15 and 59 [6]. This led to the emergence of enormous interventions to control the disease. HIV preventive measures that have been implemented include: e.g. prevention and treatment of sexually transmitted diseases, prevention of mother to child transmission, safe blood transfusion, condom promotion, and HIV testing and counselling to mention a few. Inevitably, HIV testing became crucial in the control of this disease, and has been referred to as the actual entry point to prevention, treatment and care [9].

HIV testing

HIV testing dates back to 1985 when the first antibody test was approved and made available in Western countries [10-12]. For a long time, treatment was not available, and the test was a dangerous move to some, since it made apparent avenues of additional harm to already vulnerable groups (for example, gay people). Other than a change in behaviour, it offered little to those already infected [11]. Furthermore, the fear and stigma attached to this disease made it different from the other ailments. The discovery that oneself might be infected was accompanied by much psychological harm, creating a recognition that counselling was critical [11].

The complexity of the challenges implied in the global HIV scenario made HIV testing exceptional hence ethical and human rights concerns became abundant. The CDC guidelines released in March 1986 advocated routine voluntary counselling and HIV testing for high-risk groups. The risk groups included homosexual and bisexual men, intra venous drug users, individuals with signs of AIDS, prostitutes, newborn infants and haemophiliacs [13]. To protect the privacy of those testing HIV positive, confidentiality became a key factor in the public health practice in many health facilities in the United States of America. In other states, anonymous testing was practiced, i.e. identification of clients solely by numbers. It was hypothesised that ensuring anonymity during HIV testing would increase test rates compared to mere observing of confidentiality. A trial on anonymous versus confidentiality in HIV testing was conducted and evidently HIV testing increased by 50% when anonymity was ensured [14]. The increase was mainly among the gay population where the observed increase in testing amounted to 125% [14]. The explanation given was that HIV sero-positive individuals suffered high discrimination and any breach of confidentiality came with serious repercussions to the individuals such as refusal of medical insurance, loss of employment, and

eviction from housing among others factors [14, 15]. Despite the advantages in increasing test rates among the at risk groups, some disadvantages were experienced in practicing anonymous testing. These included the difficulty for the counsellors in tracing clients who did not return for the test results or for a retest in cases of errors [14].

Despite generating high test exposures anonymity was not favoured by the general public. It was argued that preventing the spread of AIDS took precedent over personal privacy (that is the practice of anonymity) [16]. In the extreme some wanted quarantine to be put in place for those infected with the virus [17]. The perspectives from the at-risk groups were weighed up against the public health views that favoured routine types of HIV testing. A balance of the two perspectives brought about an HIV testing policy where voluntarism was considered vital and where the critical role of counselling was fronted [18]. Emphasis on HIV counselling was particularly inspired by studies that reported no behavioural changes in homosexual men after knowledge of their HIV antibody status and that HIV-positive individuals suffered emotional distress upon knowing their status [19]. Counselling was seen as an important element, both in dealing with the challenging emotional dimensions as well as providing an opportunity to motivate behavioural changes. This was essential in preventing further transmission of the infection. Counselling was perceived as the way to “expedite the adoption of a safer sex norm”, said to be key in containing the disease [20]p.709].

HIV counselling

HIV counselling has been defined as a dialogue between a client and a counsellor. The objective of this dialogue is to enable a person to make informed and personal decisions about testing, and to learn how to cope with the stress brought about by the test results [21]. Counselling has traditionally been offered on two occasions during HIV testing; before the test (pre-test counselling) and after the test (post-test counselling). Pre-test counselling has been described as a prerequisite for obtaining consent [20], and according to UNAIDS, it is necessary for preparing the client psychologically for the HIV test and its results. At this stage, the counsellor discusses with the client ways to cope with knowing ones HIV status, and attempts to correct myths and misinformation surrounding the disease [21].

Pre-test counselling can be seen to fuel *autonomy*, a virtue held high within the rights based discourse and is classified as one of the elements that make up the responsiveness concept. Responsiveness is one of the health systems goals that look at the non-health aspects

considered important in enhancing trust in healthcare systems. From a responsiveness viewpoint, autonomy is described as the involvement of the health service user/client or the patient in making decisions that pertain to their health. It does assume that positive involvement can be achieved when the users are provided with relevant information, consulted on preferences, and when patients' consent is sought before any proceedings are taken. In relation to HIV testing, autonomy emerges as an important aspect. It entails that respect is observed regarding the rights of the patient to accept or refuse testing [22, 23].

The nature of the post-test counselling depends on the test result. It presents an opportunity for the counsellor to present alternative prevention methods. For instance, the counsellor can present ways to prevent HIV infection, discuss ways to reduce the risk of infection or transmission, provide emotional support on how to cope with an HIV-positive status, and make referrals to treatment, care and support services [21]. The counselling element in HIV testing services has by most participants in the field been perceived as vital with its potentially comprehensive role in HIV prevention, treatment and support measures, both at individual and societal levels [24]. A randomised trial conducted in Kenya, Tanzania and Trinidad found that individuals who received counselling were more likely to change behaviour than the ones who received merely health information [25]. Along with the opportunity to promote behavioural change and health improvement at an individual level, HIV counselling is seen to encourage openness that can in turn reduce fear and stigma of the disease at a societal level [24].

Types of HIV testing and counselling

With the approval of the finger-prick rapid test in 2002, HIV testing became possible outside the laboratory, which assisted in improving knowledge of HIV status in the population [6]. The rapid finger-prick test is still in use for HIV testing. Two main modes of testing are discussed in the following pages; voluntary counselling and testing and Provider initiated opt-out models for testing.

Voluntary counselling and testing (VCT)

VCT is a process by which individuals undergo counselling and testing for HIV [9]. These services have commonly been client or user initiated, motivated by a person's own interest and right to know his/her HIV status. VCT emphasizes pre- and post-test counselling, and has

either been integrated in established health facilities or provided independently in the community [26].

VCT has been deemed beneficial in many ways: studies have shown that it significantly helps people change their risky behaviour through, e.g. increase in condom use, and decrease in unprotected sex among the ones with a positive test results [25, 27-29]. Others studies noted changes also among the ones testing HIV-negative [29, 30]. VCT has also been shown to motivate behavioural change among couples who were tested together [28].

Despite such documented benefits VCT has faced many challenges. The most prominent has been the low uptake also in locations where the services are readily available. Several studies indicate that substantial proportions of a population express high willingness to be HIV tested, which could be an indication of poor acceptability of the manner in which VCT is offered [31-34]. Stated willingness to be tested is not the same as getting tested. New strategies to ensure increased uptake in testing have thus been sought.

The difficulties encountered with regard to mother-to-child transmission of HIV turned out to be severe, and the prevention of this form of transmission of HIV has been hampered particularly by low uptake of VCT. In this context VCT was not seen as strong enough a measure to ensure that new mothers received guidance and medication. With the demonstrated efficacy of drugs (Zidovudine) to mothers and infants in 1994, the eagerness to reach the women in question became substantial [35]. This enhanced the interest in increasing the uptake in HIV testing.

During the early years of the new millennium, a consultation was convened by WHO with a focus on alternative ways to ensure increased access to HIV testing services. The consultation team reported that new approaches to HIV testing were necessary. During the consultation meetings it was argued that the pre-test counselling that was deemed a prerequisite to HIV testing could also be a potential hindrance. Within a fairly short time, the pre-test counselling that WHO had established was considered counterproductive. From a subsequent report, it was argued that the pre-test counselling procedure in VCT could particularly detract individuals from seeking HIV testing [11, 36].

In June 2003, WHO disseminated a brief summary on HIV testing in light of the discussions that proceeded from the consultations [37]. ‘Voluntarism’, ‘informed consent’ and ‘confidentiality’ that were included in the earlier version of the CDC guidelines were retained, although a problematization of ‘HIV testing exeptionalism’ was sensed. Routine HIV testing in antenatal clinics and elsewhere in the health system where it was perceived to benefit the patient was now recommended. This change was accompanied with other transitions. The ‘pre-test counselling’ concept, previously seen as the vehicle to attain informed consent, was now replaced with ‘pre-test information’ [37]. This period moreover, saw a highly welcome increase in funding in relation to HIV and treatment e.g. through initiatives like the ‘3 by 5’ that was launched in 2003 by WHO. This initiative aimed to reach three million HIV-positive patients with treatment by 2005. Globally oriented organizations mobilised rapidly to increase the availability of anti-retroviral (ARV) drugs. Unfortunately by itself this initiative did not yield much result, and the anticipated targets were not reached by 2005 [6]. This focus on treatment has partly distracted the focus from HIV prevention campaigns.

Provider initiated opt-out HIV testing and counselling

As declared by a senior WHO representative, changes in the testing strategies became inevitable to give better access to ARV drugs [38]. In addition, new views related to making HIV testing a ‘routine examination’ in healthcare settings supported by studies from Botswana, made a significant impact as WHO underwent a change in its approach to HIV testing [39, 40]. After almost a year of scrutiny, new guidelines on ‘provider initiated testing and counselling’ (PITC) were released in May 2007 [26].

The PITC guidelines differed substantially from the ‘traditional’ VCT concept. The differences emerged in relation to the manner of initiating testing, the objective of the testing, and the emphasis on HIV counselling. PITC gained momentum to increase testing levels in high prevalence populations with the assumption that it would imply simultaneous benefits for HIV prevention and treatment [26]. The ‘provider initiated opt-out approach’ has most commonly been implemented in specialised programs, such as the prevention of mother-to-child transmission (PMTCT) program. The provider initiated opt-out approach entails that HIV testing is initiated by the health provider, who proceeds to test the patients unless the patient actively declines testing after receiving the ‘pre-test information’ [26]. Thus in principle the provider initiated testing approach retains the option for clients to decline testing. Pre-test counselling is, however, eliminated and substituted with pre-test information. Post-

test counselling is still recommended within the provider initiated opt-out approach [26]. Plans are underway to scale up this model of testing in many nations.

Success of the provider initiated opt-out testing strategies has been reported in some studies, primarily in terms of increasing the test levels and in terms of the proportion of women receiving PMTCT [41-45]. Critical voices have however been raised. A study from Botswana found that although routine testing was widely supported, a majority felt that they could not refuse the test, making issues on the applicability of informed consent and the autonomy of patients questionable during implementation of the new strategy [46]. The roll-out of this testing model has over time been faced by criticism both from the ethicists and human rights activist. The model is criticized for paving the way for neglect of rights-based dimensions retained in the WHO 2003 guidelines on new approaches to HIV testing with its emphasis on 'voluntarism', 'informed consent' and 'confidentiality' [47-49].

1.3 HIV/AIDS in Kenya, Tanzania and Zambia

The data for this thesis were drawn from three African countries; Kenya, Tanzania and Zambia. The estimated HIV prevalence among young people aged 15-24 is 2.8% in Kenya [50], 6.5% in Zambia [51], and 2.4% in Tanzania [52]. HIV/AIDS has affected all sectors (e.g. education sector, agricultural sector among others) in these countries, hence preventive, treatment and care strategies are deemed necessary and are in place to manage the disease

HIV testing is one of the most important interventions in HIV/AIDS in all the three countries. In Kenya VCT was first launched in 2000 with only three sites available. This was scaled up, and there were about 1000 sites countrywide by 2007 [53]. Tanzania, on the other hand, initiated VCT earlier in 1989. However, coverage was remarkably low, and only about 92 public VCT sites were available in the country in 2001. The preceding years have experienced a scale-up of the services and across the country. In Zambia there were about 1563 VCT sites in public and private health facilities by 2009, [54]. With the introduction of the PITC model of testing these governments have scaled-up the testing strategy, primarily through the prevention of mother-to-child transmission (PMTCT) programs. However, plans are underway to make HIV testing a routine test so as to reach all that utilise health facilities. An example is Kenya where the current guidelines propose HIV tests for all patients using health facilities [53].

1.4 Health system values in the context of HIV testing

Health systems are defined as all activities whose ultimate goal is to improve the health of the population [55]. Not only do these systems aim at improving health, but they respond to the needs and expectations of the people being served. In this regard, 'responsiveness' has become an important concept directed at the non-health related dimension increasingly being considered as one of the main goals of a health system in order to respond to the needs of the people [55]. WHO indicated that some of the outputs of a well-functioning health system are responsiveness and equitable access to people-centred care [56]. HIV counselling and testing (HCT) services are central part of the health system in combating the pandemic as they provide an entry point to prevention of infection or treatment of those already infected. Like any other services within the health system, HCT services must respond to people's needs and expectation.

Responsiveness

The concept 'responsiveness' is as stated above, one of the goals of a health system making it an 'output' that should be targeted [55]. Responsiveness entails the non-health aspects that are closely related to the way in which people are treated and handled by the health system. Responsiveness in this context emerges as a crucial factor that influences trust and use of health services. According to the literature responsiveness can be viewed from two angles. Firstly, when users of a health system are perceived as 'consumers', emphasis on responsiveness is looked upon as a means of attracting consumers. Secondly, responsiveness can be related to the safeguarding of the rights of patients to adequate and timely care [57]. It is assumed that a responsive health system is conducive for individuals to seek care earlier, and makes them more open in their interactions with the health providers. In turn this implies that the health-care users are better able to assimilate vital information which ultimately contributes to the enhancement of health in a population [22]. A responsive health system thus contributes to increased utilization [22].

WHO defines 2 major components to measure responsiveness, namely, 'respect for persons' and 'client orientation'. Respect for persons focuses on aspects of the individual interaction with the health system [23, 57-59]. It involves elements such as 'dignity', 'autonomy', 'confidentiality' and 'communication', whereas 'prompt attention', 'quality of basic amenities', 'choice of provider' and 'social support' make up the client orientation component. The outlined elements are central in measuring responsiveness of health systems. These

elements have been used to measure responsiveness levels of health systems in several studies [22, 23].

Health system responsiveness emerges as particularly important in a context of HIV testing services due to the heavy stigma attached to the disease. In an HIV context, a responsive system that offers people centred approaches to testing emerges as vital for trust and acceptability of the related services. Hence, responsiveness emerges as a highly relevant concept in evaluating such services. In the research presented in this thesis, I attempt to address how the responsiveness concept can be drawn upon in assessing HIV testing services.

Equity in health care

Equity is indicated as one of the basic principles in healthcare provision by most nations [55, 56]. The concept 'equity' has been widely debated over years, being given a number of different definitions, all of which revolve around common points of 'fair distribution', for example of health services, among different individuals and groups in a society [59-62]. "Equity is concerned with creating equal opportunities for health and bringing health differentials down to the lowest level possible" [61]. Whitehead [60] proposed a definition of equity in healthcare that consists of three different dimensions: *equal access to available care for equal need* - which entails even distribution of resources and facilities. When available resources are spent on high-technology health services that provide for only a small portion of the population, it is judged as inequitable. *Equal utilization for equal need* occurs when the use of services is not restricted by social or economic disadvantage. However, if people choose not to use services for ethical, cultural or religious reasons - the concept does not apply. *Equal quality for all occurs* when professionals put the same effort into their work with all social groups.

Going beyond the definitions, equity is seen as both a normative and an empirical concept. The normative dimension addresses the question of values, that is, the ethics and desirability of interventions, whereas the empirical dimension deals with assessment of health status and determinants [61]. The major goal of equity in health is to provide and enhance opportunities for all individuals to achieve optimal health within their potential [61]. In this study, I attempt to determine whether VCT services are equally utilised by the population they serve drawing upon the Andersen's behavioural model for health service use as discussed in the methods section.

1.5 Problem statement

In the past 15 years there has been a substantial scale-up of HIV testing through VCT services, and most recently through the PITC model of testing. Studies have reported low uptake of client-initiated VCT even in places where the services are readily available [63]. Explanations have been sought for the low use of VCT. The available literature indicates that the lack of treatment, low trust in the local health care services, stigma, confidentiality concerns, and adverse consequences related to disclosure are key barriers to VCT uptake [12, 32, 34, 63-65]. Other studies have shown inequities in access indicated by higher utilization of VCT among individuals with higher levels of education compared with groups with low education [32, 66]. Equity in access has been declared a central strategic goal by most national HIV authorities.

The PITC models sharply contribute to an increase in the number of people testing for HIV. The PITC model has mainly been adapted by specific programs targeting particular categories of individuals within the population primarily pregnant women through the prevention of mother-to-child transmission programme (PMTCT) [41, 67, 68]. The PITC model has been seen that it could violate ethical principles brought about by the lack of counselling and for subjecting mainly women to testing [47, 49, 69-71]. Knowledge related to people's own experiences with the new testing model is however, very scarce, and the demand for studies to explore perceptions and experiences with the new strategy has been raised [47, 72]. This study attempts to address this void by generating knowledge on experiences with both VCT and the PITC models of testing.

In a context where HIV testing through the highly scaled-up VCT model has faced poor utilization, the responsiveness concept can be seen to be of vital importance in evaluating these services. Previous studies have mainly assessed responsiveness of entire health systems [58, 73]. However, a few studies have assessed responsiveness of specific health services (mental health) [74-76]. The responsiveness concept had not previously been applied on HIV services.

2 Study Objectives

2.1 Overall objective

To investigate HIV testing services from health providers, clients and community perspectives.

2.2 Specific objectives

1) To investigate factors that determine the use of VCT based HIV testing services in three African districts.

2) To investigate exposure of HIV testing in the adult population, and to explore perceptions and experiences with VCT and PMTCT based provider initiated opt-out testing approach at ANC clinics in three African districts.

3) To assess the applicability of the responsiveness concept in evaluating HIV testing services with a focus on VCT.

3 Material and Methods

3.1 REACT Project

The study was conducted as part of an ongoing EU-funded research project, REACT (REsponse to ACcountable priority setting for Trust in healthcare) implemented in three African districts; Malindi in Kenya; Mbarali in Tanzania; and Kapiri Mposhi in Zambia; (Figure 1 provides a map showing the three districts). REACT is an action research based health systems project that draws upon the ethical framework 'Accountability for Reasonableness' (AFR) to promote fair priority setting in healthcare. The AFR framework is applied as a 'tool' by decision makers for guidance towards fair decision-making processes. AFR aims to broaden stakeholder involvement in the identification and consideration of relevant values in priority setting for healthcare based on continuous reference and adaptation to the four conditions of i) relevance, ii) publicity, iii) appeals and iv) leadership [77]. The influence of AFR in this project is assessed in the recorded change in the priority setting processes and in indicators for 'trust', 'equity' and 'quality'. A recent publication based on health systems and REACT data in Tanzania indicated the need for more participatory and mutually acceptable decision-making processes in improving healthcare [78].

HIV control programmes are one of the focused 'evaluation domains' within REACT. The knowledge generated within the frames of REACT are drawn upon in papers I and II. The papers highlight some of the gaps in the implementation of HIV testing policies as experienced by our study participants. The knowledge generated creates a basis for addressing fairness of decision-making process for HIV testing in the above mentioned districts.

3.2 Study area

The main study area for the present study was Malindi district. However, relevant data from the other REACT project districts (Mbarali and Kapiri Mposhi) were analyzed and presented in papers I and II. These districts were chosen by REACT on the assumption that they were similar in disease burden, health systems and population [79]. A closer look at the available epidemiological information, however, revealed some distinct differences, particularly with reference to HIV prevalence [52, 80-82]

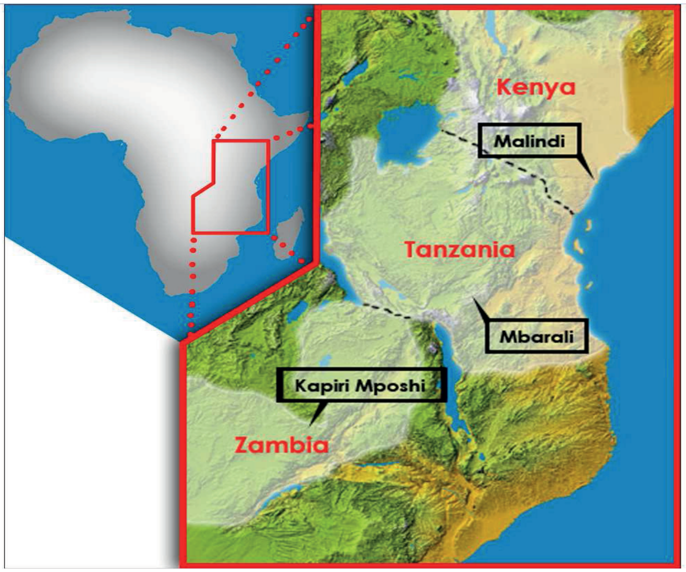

Figure 1: Map of the study areas in the selected countries.

Malindi district

Malindi district is one of the seven districts in the coastal region of Kenya. The district occupies a geographical area of 7605 square kilometres and is largely semi-arid [83, 84] Malindi district is divided into three administrative divisions, 16 locations and 56 sub-locations. It has an estimated population of 350,000 [84]. The district has a rising population with a growth of about 3.9% (1989-1999). The district has three hospitals (one government and two private) and 24 dispensaries (17 GOK- and 7 NGO-based). HIV/AIDS is one of the most prevalent diseases in the district with levels ranging from 15 to 17%, a range that is much higher than the national prevalence (6-7%) [50, 80, 84].

Mbarali district

Mbarali district is located in the Mbeya region of Tanzania. It has two divisions with 11 wards and 98 registered villages. The district has a population of ~ 235,000, with a growth rate of 2.8% [85]. Residents of Mbarali district depend on health services that are provided primarily by the government, although services offered by the private sector inclusive of NGOs are increasing. There are two hospitals, two health centres and 39 dispensaries in this district [86]. The HIV prevalence in this district is estimated at 7.9%[52].

.

Kapiri Mposhi district

Kapiri Mposhi is a district located in the central province of Zambia; it has a population of almost 275,000 of which ~20% live in urban areas. The annual population growth rate is 5.8%. Health services are primarily provided by the government with very few private and mission facilities. HIV is one of the most prevalent diseases in the area, with an estimated level of 13% in the rural part and 32.2% in the urban part of the district [81, 82].

3.3 Study design and rationale

A cross sectional design was applied in paper I, and a mixed-methods study design was applied in the other two papers (Papers II and III). The mixed methods approach entails mixing both quantitative and qualitative methods of data collection in a single study [87, 88]. The challenges of HIV testing are highly complex, and a combination of methods was deemed appropriate to generate knowledge that could address the research questions in a broad and comprehensive manner. It was anticipated that qualitative interviews would add content and meaning to the knowledge generated by the survey data, thus increasing our understanding of the patterns of utilization of HIV testing (Papers II and III) and people's experiences with VCT and the PITC HIV testing model practiced at ANC settings. Mixed-methods are considered important in providing an opportunity to present divergent views, producing more nuanced knowledge important in informing theory and practice, and adding insight and understanding to what may have been missed if only a single method of research investigation was used [87].

Creswell et al.[88] outlines three main issues that are used to define the type of mixed-method approach used. First, the nature of data collection detailing whether it happened concurrently or sequentially is considered. Second, it needs to indicate whether the data sources are equal by explaining if one dominates over the other hence, indicating the priority of the data. Third, the place in the research process where the mixing of the data occurs is taken into account [88]. This background has been used to develop six mixed-method designs that can be employed in research [88]. In our study, two types of mixed-method design were used, namely a concurrent triangulation design (Paper II) and a concurrent nested design (Paper III). These are presented in detail in the next sections where the designs of the specific papers is described. Details related to study populations and study settings are described in the specific papers attached to this thesis.

Paper I, Study design

A cross-sectional survey design was used to investigate the determinants of use of HIV testing strategies (VCT). Data were drawn from a population-based survey carried out as part of the REACT project in 2007, conducted in the three selected districts as shown in Figure 2.

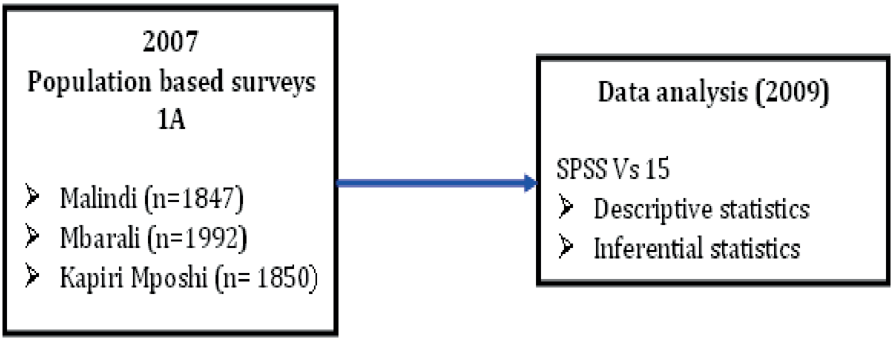

Figure 2: Cross-sectional study design

Paper II, Study design

Paper II investigated exposure of HIV testing, and explored people's experiences and perceptions in relation to the provider initiated (opt-out) HIV testing model. A concurrent triangulation mixed-method design was applied that entailed the use of both quantitative and qualitative methods. Data collection took place 'concurrently' or during the same phase of the research in this design [88]. The quantitative section included questions that were part of the population-based survey conducted among adults in the three districts (as in paper I), while the qualitative data-set was obtained from a total of 34 focus-group discussions (FGDs) and 18 in-depth interviews (IDIs). 24 of the FGDs originated from REACT, eight FGDs being conducted in each district. The remaining 10 FGDs and 18 IDIs originated from a study focusing on HIV testing, counselling perceptions and experiences conducted in Malindi district of Kenya (Figure 3). The data sets are described in the next sections.

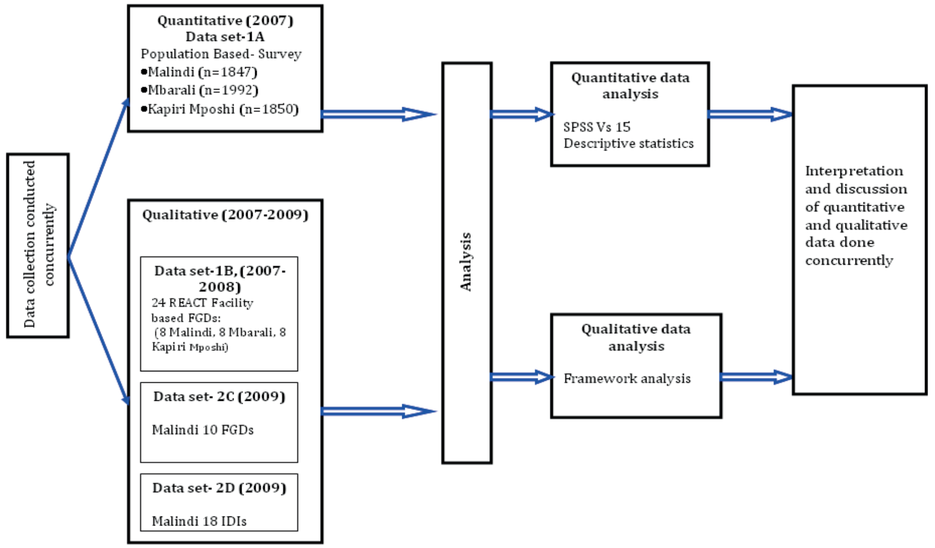

Figure 3: Concurrent triangulation mixed-methods design

Paper III, study design

With the very clear VCT related challenges on low use that have emerged from previous studies as presented in the introduction, the concept of 'responsiveness' emerged as potentially important. Thus assessment of the tool used to measure responsiveness was deemed necessary as a first step. To assess the responsiveness tool in evaluating VCT services, a concurrent nested mixed-method design was employed (as in Figure 2). An explicit aim was to explore "responsiveness" in the context of HIV testing in an African district. Quantitative and qualitative data were collected during the same phase of data collection [88], using a standardized questionnaire that had closed-ended questions proposed by WHO to measure responsiveness. The tool was adjusted to include one open-ended question at the end of the questionnaire. The same study participants thus replied to both the quantitative questions as well as to the open-ended question. The tool was administered to 328 VCT clients and 36 VCT counsellors (health providers); a total of 364 participants. 300 participants (both users (264) and health providers (36)) agreed to reply to the open-ended question. Observational field-notes were also written during the study.

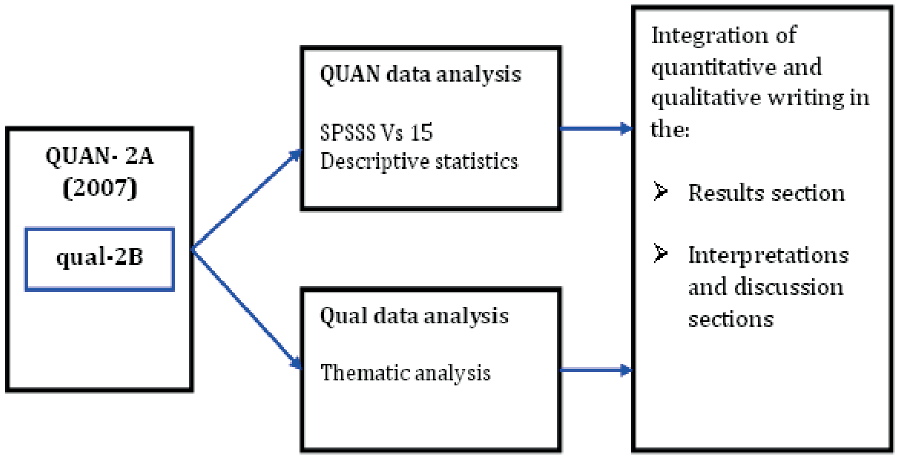

Figure 4: Concurrent nested mixed-methods study design

3.4 Data collection methods applied

All together, the study utilised seven data-sets generated through quantitative and qualitative techniques. These data-sets were conducted within the REACT project or through separate studies conducted in Malindi district by the author of this thesis. The data-sets within REACT were obtained from the three study districts. I was involved in collecting the data from Malindi but not the data collected within the REACT project as indicated in table 1 below. These data collection methods are discussed in detail in the next sections.

Table 1: Description of the data collection involvement of the different data set

Data collection method	Assigned number	Source of data	Was the author involved in the data collection?	Who were involved
Population based survey (PBS)	1A	REACT	NO	REACT members and co- authors
Focus group discussions (FGD) (First set)	1B	REACT	NO	REACT members (social scientists), co-authors
Facility based survey (FBS)	2A	MALINDI	YES	Field assistants
Open-ended question	2B	MALINDI	YES	Field assistants
Focus group discussions (FGDs) (Second set)	2C	MALINDI	YES	Social scientist
Individual interviews (IDIs)	2D	MALINDI	YES	Social scientist
Observations	2E	MALINDI	YES	None

3.5 Quantitative Methods

Two main types of surveys were used, namely a population-based survey - 1A (PBS) and a facility-based survey- 2A (FBS). The PBS was conducted within the REACT project in the three selected districts, whereas the FBS was conducted only in the Malindi district.

Population-based surveys - 1A (Papers I & II)

A multi-stage stratified random cluster sampling strategy was used to draw the samples. In each district, the sampling frame was stratified into rural and urban areas. A cluster of households referred to as a 'standard enumeration area' (SEA) considered as the primary sampling unit was selected using probability proportional to size of the sampling unit. The required sample size was calculated to be 2000 individuals (aged 15 and older) in each district. The study was handled as a baseline survey for the REACT project, and the sample size calculation was computed with the assumptions of a 0.5 probability of outcome, a precision of 0.05, a minimum measurable change of 0.1, a power of 0.80, and the design effect at 2. A total of 49 clusters (10 in Malindi, 19 in Mbarali, 20 in Kapiri Mposhi) were selected from the urban stratum and 67 clusters (16 in Malindi, 26 in Mbarali, 25 in Kapiri Mposhi) were selected from the rural stratum. A random selection of a fixed number of households in the selected SEAs was done. At the household level, one male and one female aged 15 and above were randomly selected to participate in the survey. The author of this thesis was involved in data analysis but not developing the design, developing the tools or collecting the data of this study.

Facility-based exit survey – 2A (Paper III)

Exit interviews were conducted in the 15 VCT facilities available in Malindi district at the time of the study. To avoid recall and recognition biases, exit interviews were considered the best option in capturing responsiveness of VCT services. The exit questionnaire was administered to users of VCT, and to healthcare workers at the VCT centres. A sample of 325 people was calculated to be required (assumptions being a 90% confidence interval and a probability of 50% of the measured phenomenon). At the study district, we aimed to interview all who attended the VCT within the span of one month (mid October to mid November 2007). Since VCT offers client-initiated services for HIV testing, we faced the challenge of reaching our sample target size if we randomised. Therefore, we chose to interview all individuals over 18 years of age who underwent VCT and who consented to take part in the research. Facility

records of the previous four months were used to calculate proportionate samples for the respective VCT centres.

The questionnaire employed that had been developed by WHO to measure responsiveness was, slightly adjusted to fit the study setting (VCT) and to include one open-ended question. The open-ended question was added as a result of the pilot study as the researchers sensed that there were aspects that were not properly covered by the questionnaire. Four field workers were recruited to assist in data collection. Selection was based on their experience with similar studies and good knowledge of the area. Two days were spent on training the four field workers. At the VCT centres we interviewed both the healthcare providers (mainly counsellors) and the users. We depended on the healthcare providers to appraise the VCT users about the survey. At the health facilities we were either offered a separate room to conduct the interviews or we sat outside under a tree in places where no extra room was available.

3.6 Statistical analysis and the applied behavioural Model

Both data-sets (the population-based survey - 1A and the facility based exit interviews – 2A) were analysed using SPSS version 15 for MS windows (SPSS inc. Chicago, IL, USA). Descriptive analysis was performed for both studies. To guide the analysis in paper I, the behaviour model for health services use by Andersen was employed [89]. The model has been used extensively particularly to investigate questions related to equity in the use of healthcare services after adjustment for need factors, i.e. equal use for equal need. Need attributes for VCT is less apparent compared with general healthcare seeking where illness and perception of one’s own health status are the most powerful determinants. Therefore, the original model suggested by Andersen [84] demanded modifications for our study, which was based on a careful literature review of the determinants of HIV testing. This has been discussed in detail in paper I attached with this thesis. Logistic regression analysis was used to test the performance of the modified model, and to estimate associations between factors and VCT exposure. All analyses were performed by first stratifying data by district, residence and sex. Potential interactions were sought and tested. Only the significant factors were considered before performing a pooled analysis for all three districts.

Figure 5: The modified behavioural model for health services use [89, 90]

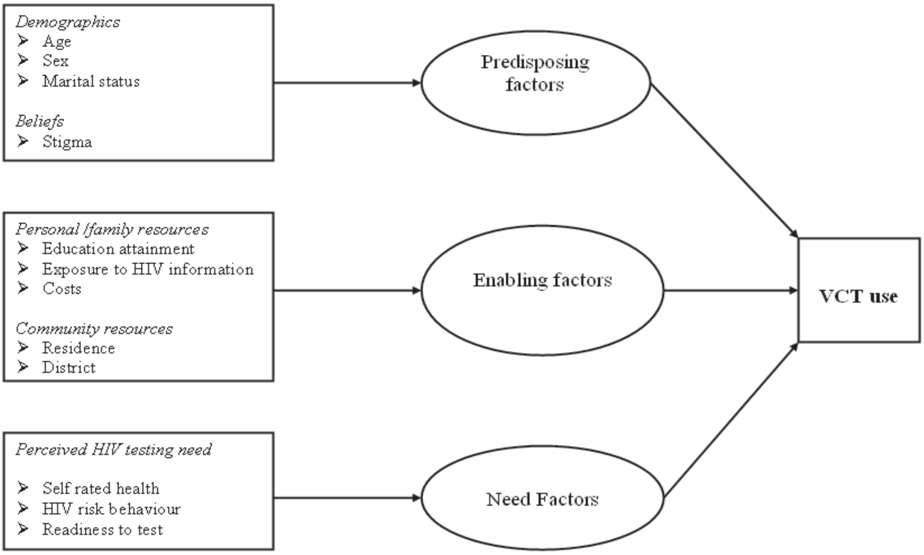

3.7 Qualitative Methods

Qualitative research entails methods of inquiry aimed at gaining a depth of understanding of the issue(s) at stake, and the reasons behind their occurrence [91]. Qualitative research methods involve "systematic collection, organization and interpretation of material derived from talk and or observation" [91]. Qualitative approaches have gained increasing popularity in medical and health sciences research due to their ability to enhance the understanding of people's attitudes, norms, beliefs, and their behaviour or conduct. Qualitative research methods can be employed on their own or, as in this thesis, jointly with quantitative methods. They are used here to compliment or offer more insights on the same social phenomena addressed by quantitative approaches. In this study, the aim was to enhance the depth of knowledge about HIV testing strategies in ways that would enhance the understanding and the validity of the study findings [91, 92]. Triangulation of qualitative methods was seen as a way to increase the credibility of our study findings. The methods used to collect the qualitative data were focus-group discussions (FGDs), in-depth interviews (IDIs), an open-ended question added to a quantitative tool, and observations. These methods are discussed in the following sections.

Focus-group discussions

A focus-group discussion (FGD) is described as a data collection tool that gathers people from similar backgrounds or experiences to discuss a specific topic of interest [93]. Focus-group discussions were used in this research to elicit people's perceptions and experiences surrounding HIV testing strategies. Two sets of FGDs (1B and 2C) were utilised. FGDs 1B were conducted at health facility level in each of the study district among the following groups: a) female out-patients, b) male out-patients, c) pregnant women attending ante-natal clinics and d) youths aged 18-24 years. Two FGDs were carried out in each group from the rural and urban setting in the districts (i.e. a total of eight groups in each of the three districts). The author did not carry out these interviews herself, but followed the research process closely. The second set of FGDs (2C) involved 10 FGDs that were conducted at community level in Malindi district among a) female youths, b) female adults, c) male youths, d) male adults and e) pregnant women, in both rural and urban settings, again with two FGDs carried out in each category.

FGD participants for both sets of groups were *purposively* selected. Purposive sampling is the common method for selecting informants for focus-group discussions [93]. [It is a non-probability sampling method also known as 'judgemental sampling' that is used to select participants based on the researchers personal judgement about which ones will be most representative or informative [94]pg. 729].

The first set of FGDs- 1B (Paper II)

This set of FGDs was conducted within the REACT project in the rural and urban settings of the three selected study districts. Discussions were conducted by experienced social scientists speaking the local languages. The individuals who carried out the FGDs from Kenya, Tanzania and Zambia respectively had been part of REACT since its inception. The process of recruitment of informants was done with the assistance of health providers at the health facilities, and interviews were conducted in a room provided within these facilities. The inclusion criteria were based on age, gender and use of the health facility. eight FGDs were conducted in each district /country consisting of 6 -10 participants in each session. The study informants were recruited immediately after use of the service to avoid recall bias. The topics addressed in the discussion guide were a follow-up of the topics addressed in the population-based survey (1A). The individuals who developed the guides also conducted the interviews, and were part of the analysis and the writing process of the paper (co-authors of Paper II). The

tool development and training took place during a REACT consortium held in 2006 in the Mbeya district in Tanzania.

The developed study guides included topics on selected programmes within the areas of HIV, malaria and obstetric care. The questions addressed experience with diverse interventions in place within the health service delivery systems, among them the HIV testing services. Only the questions on HIV were used for this particular study. The topics in the FGD guide of interest to the current study more specifically included the following:

1. What are your experiences with HIV prevention programs?
2. Should someone wish to know his or her status, where would they go?
3. How easy or difficult would it be to get an HIV test?
4. What are the reasons for young people not using VCT services?
5. How could VCT services be modified to make them more acceptable for young people?
6. What did you learn at the ante-natal clinic about mother-to-child transmission?

Before the start up of the discussions, the researchers introduced themselves and allowed everyone in the group to introduce themselves. The introduction was followed by an explanation of the study focus and its purpose before seeking verbal informed consent from those wishing to participate. All the discussions were audio-recorded and later transcribed.

The second set of FGDs – 2C (Paper II)

Whereas informants for the first set of FGDs (1B) were recruited at the health facilities, the second FGDs 2C aimed at recruiting regular community members. This was considered important in capturing perceptions also of people who may not utilise health facilities, as well as the experiences of those who may have used the HIV testing services, but had not felt free to discuss issues when recruited at health settings by the health providers. This set of FGD was motivated by the knowledge that vast numbers of people wish to test themselves for HIV but few end up going for the actual test. These FGDs were conducted only in Malindi district in Kenya. The study areas were chosen purposively as we wished to conduct research in the same area where the REACT project FGDs (1B) had been conducted to provide a wider community based knowledge within REACT. Thus Malindi town was selected as the urban setting and 'Gongoni 'as the rural setting. Topic guides were drafted and revised through several rounds during the research process. The process included the author of this thesis, two anthropologists and two (AB, IKN) other social scientists (MT, LN) in this sub-study. The

guides were first ‘pre-tested’ prior to the study in Malindi. This process was not carried out to ‘fix’ the questions, but to predict how long the interviews would take. This round was also useful in order to make an initial modification of some of the questions that emerged as unclear to the informants. We worked closely with community health workers in the recruitment of the study participants. The studies were conducted at convenient locations, e.g. the nearest community-based organization (CBO) or health centre. The rural interviews were conducted at Gongoni Health Centre and at the Malindi Educational Development Association (MEDA), a local CBO in Malindi town. A total of 10 focus-group discussions were conducted with the above mentioned categories of informants. Groups consisted of between 7 -10 participants. The discussions were conducted in a separate room provided at the two institutions which offered adequate, quiet and private environments enabling the researchers to audio- record the discussion with ease. Also in this study, the inclusion criteria applied was age and gender to enhance the opportunity for participants to discuss freely their opinions on HIV testing. An experienced social scientist moderated the FGDs. The author was present at every FGD, and observed the dynamics of the group, audio-recorded all the discussions and participated in probing whenever the responses were unclear or needed more depth and detail.

The aim was to explore perceptions and experiences related to VCT. In the course of the discussions and interviews, the particular experiences with provider initiated opt-out approaches in HIV testing came up strongly. The topic was followed up and scrutinized with additional probing questions. Before the start of each discussion, I gathered socio-demographic information of each individual, including age, marital status, level of education, occupation, history of HIV testing and where tested. This information enabled us to become acquainted with the informants before the discussions commenced. The participants were also curious about who we were, and what we were interested in discussing. These relaxed sessions gave us the chance to familiarise with the participants, and gain knowledge on who was speaking about HIV testing based on experience. The sessions started with the researchers (the moderator and I) introducing ourselves followed by an introduction of the topics for discussion and the polite request for consent. All discussions commenced after verbal informed consent was granted by each member of the group, and after a short introduction was given by each of the group members.

During the discussions, we did allow the participants to ask questions when they were uncertain, and we encouraged the participants to feel free to speak of their opinion. We discussed the fundamental principle of anonymity in research and also encouraged the participants to keep the information that was shared during the discussion to themselves
The groups differed in terms of participation. In most of the groups the participants were somewhat hesitant to talk initially, but in most groups the shyness did not last long, not the least in the discussion groups with the older participants (males and females in both settings). As the discussions progressed, almost all the informants showed a genuine interest in the topics, and voiced that these issues engaged them directly. In one of the FGDs with adult males in Malindi town, some of the participants did indicate that the time allocated for the discussion of such topics should be extended. The FGDs conducted among the younger participants lasted about two hours while the discussions with the elders lasted closer to three hours.

During all the discussions two voice recorders were used to audio-record. Word by word transcriptions were produced, but the recordings nonetheless remained important and the researchers would retrospectively listen to catch the tone of the expressions and the atmosphere during the interviews. At the end of the interview the moderator summed up by repeating the major issues that had emerged for clarity and additional feedback before thanking the informants for the important discussion. Transport costs were reimbursed to all the participants.

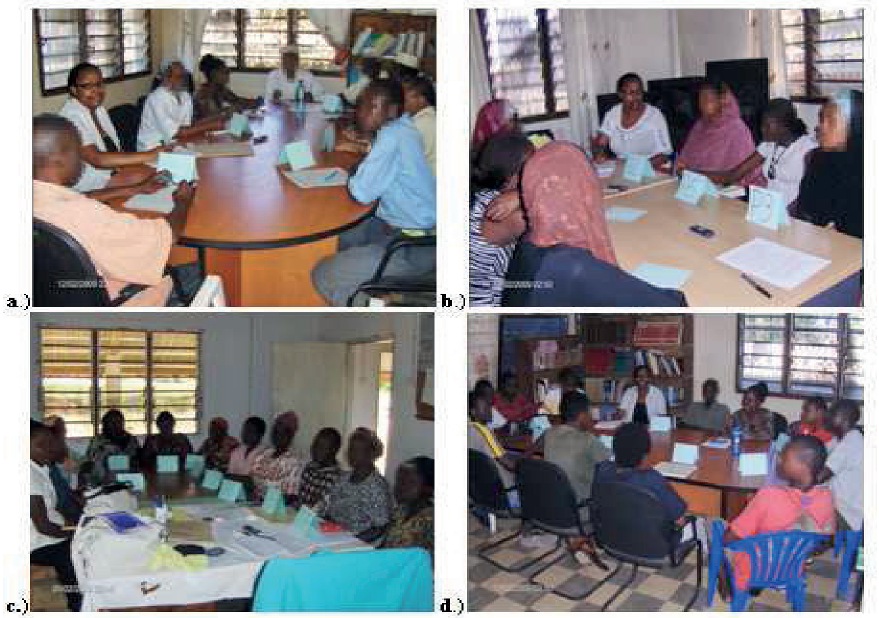

Picture 1: selected focus-group discussions with: a) male adults, b) young females, c) female adults and d) male youths

In-depth interviews- 2D (Paper II)

In-depth interviews (IDIs) were used to gain increased knowledge of experiences with HIV testing strategies. These were carried out at the same time period the FGDs (2C) in Malindi were conducted. This method of research inquiry (IDI) was added because of its strength in eliciting individual experience, opinions, feelings and their strength in enabling the researcher to address sensitive topics. IDIs were thus included to add potential insights of experiences with the HIV testing services that would be potentially difficult to voice in gatherings of people (FGDs). The venues used were the same as those used in connection with the FGDs conducted in Malindi district. The interview guides employed during the interviews were drafted and revised through several rounds during the process. This was carried out in collaboration with a group of social scientists who were part of the REACT project (some of whom were co-authors of paper II). The guide was translated into Swahili. The IDI interview guides intended to capture the details of HIV testing, counselling, perceptions and experiences related to the services. Recruitment of the participants was done purposively by the community health workers like for the focus-group discussions in Malindi town and the Gongoni area. Participants were recruited based on age (18 years and above) and sex (men and women) in urban and rural settings as was done for the FGDs (2C).

The interviews were conducted with 18 study participants. The interview guide was used in a very flexible manner, but was still useful in focusing the interviews. The interviews were conducted by myself together with an experienced social scientist (the same person acting as moderator for the FGDs), either in separate rooms or under a tree depending on the informant's preference. Sometimes the interviewee preferred to be interviewed at their place of work (e.g. a kiosk and a hair salon). At the beginning of the interviews, the majority of the informants were reluctant to offer information as they were wary of the audio-recorders. However, with the assurance that this was just an aid for the research and that the tape would not reach others than the researcher, the study participants gradually relaxed. We also assured the participants that the recorder was only recording the voices and was not taking videos as many had suspected. As the conversations progressed, the informants became more relaxed, and most of the informants during the course of the interview became eager to share their experiences or perceptions of HIV testing.

Swahili was the main language used, and the fact that the researchers were both conversant with this language was important for the flow of the conversation. Those who had not had direct personal experience with HIV testing shared their personal reasons for not seeking the services. Sometimes the informants did not share their own experience, but found it easier or more relevant to share a relative's experience. Most of the IDI's lasted one to two hours, most of which were highly informative in the sense that details of notions or experience with HIV services were elicited (at times in quite emotional terms).

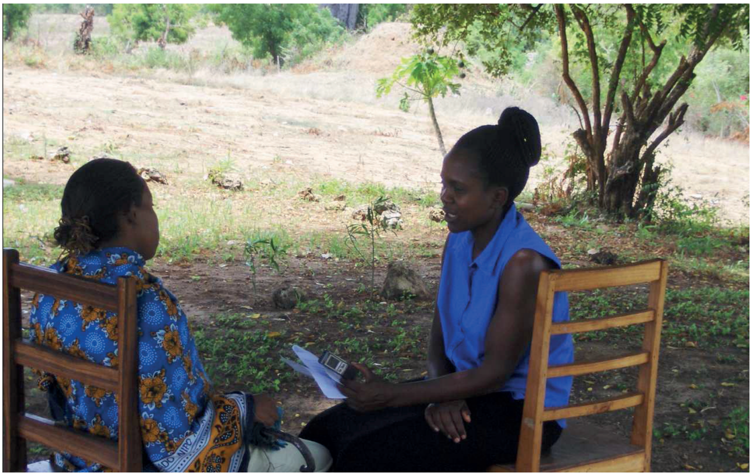

Picture 2: An in-depth interview with a young female in rural Malindi (Gongoni area)

Open-ended questions – 2B (Paper III)

Although not a method per se, we have nonetheless found that the open-ended question placed at the end of the responsiveness tool should be commented on under the qualitative part. Albeit this part of the study consisted merely of one open-ended question in the VCT exit survey questionnaire (2A), it turned out to yield very important insights. [Open-ended questions are added to a questionnaire when researchers wish to pursue a particular idea or responses in more detail [92] p.13]. In our study, we intended to assess characteristics relevant to responsiveness which we believed were not being captured using the closed-ended questions. The question was thus addressed to the same individuals who had participated in the survey. The question was phrased: "*In your view, are there any other characteristics beyond the ones we have discussed that you think should be included in a responsive VCT".* Since only one open-ended question was asked, we were able to ask everyone if they wished to respond to the question. Probing or follow-up questions was consistently applied to grasp as much as possible about aspects of relevance that had not been captured by the WHO tool. Before proceeding to this section of the questionnaire, the informants were asked if they would agree to continue with a question that had a different character. 300 participants responded (264 of the 328 VCT users and all the 36 health providers).The interviews were conducted by myself with the help of the four trained enumerators (the same that conducted the FBS-2A) in Swahili and English languages. The replies were hand-written. People were often asked to speak slowly or to repeat statements to allow important replies to be noted down.

Observations – 2E (Paper III)

Observational methods can be important both in itself, and as a method used to question or clarify responses given by the participants. This type of data collection method is commonly used to nuance or corroborate responses given by the informants [92]. Non-participant observational methods were used (paper III) in an attempt to clarify some of the concerns raised by our informants related to the responsiveness of the VCT. The observations were deemed relevant to capture issues regarding the location of the VCT, the dynamics between people using VCT, the reception of the ones seeking VCT by the health providers, body language or gestures by users, the VCT infrastructure, sitting arrangements before a VCT session, type of health provider and information education and communication (IEC) material available at the health facility amongst others. Details related to these and other observations were noted down in some detail by the author of this thesis. Where possible, photographs

were also taken after asking for permission. The observation techniques emerged as important in elucidating some of the issues raised by the study informants related to responsiveness concerns.

3.8 Qualitative data analysis

The analysis phase of the data for papers II (1B, 2C & 2D) and III (2B & 2E) started during the interviews. The more rigorous analysis took place after the data collection had been completed, and the interviews had been transcribed and translated. This process entailed a first vital step of reading through the material several times in a slow and very thorough manner. It was during this stage that the prime emerging themes were identified for both papers. Later stages implied going through all the material and meticulously coding the content so as not to miss relevant pieces of information. Two main types of analysis were used to analyse the qualitative material collected for this thesis, namely *framework analyses* for paper II and *thematic analysis* for paper III. These two approaches are however not experienced as being very dissimilar, as will be seen from the sections below. Both are inductive in the sense that they derive key themes from the data[92].

Transcriptions and translations

All the interviews (both focus group discussions and individual interviews) in both the REACT study (carried out in Kenya, Tanzania and Zambia) and in the sub-study carried out in the Malindi district, Kenya, were audio-taped and transcribed verbatim to the language employed during the interview (*Swahili* in Malindi and Mbarali and *Bemba* in Kapiri Mposhi). The focus-group discussions conducted within the frames of REACT were moreover translated to English at country level. As I had not been directly involved in the data collection of this first set of FGDs, I had to depend on word by word transcripts of the interviews as well as on discussions with the data collectors. I read all the interviews a number of times to familiarise myself with the data. In the process of analysis, I worked in close collaboration with the data collectors in REACT, one of whom was part of the write-up of paper II (E.S).

The transcription of the FGDs (2C) and IDIs (2D) conducted in Malindi district began in the field. This was done verbatim with assistance from the moderator and two other qualified social scientists with experience. All the transcribed material was later translated into English by professionals. To retain the original meanings conveyed, the translated text (English text)

of the sub-study in Malindi (2C and 2D) was typed directly under the original text (the non-translated text), thus keeping the 2 texts in the same document. This allowed the researchers to move back and forth between the original ('raw') text and the translated text for the confirmation of content and clarifications of meanings. Four of the five authors of paper II (MKN, AB, EHS and IKN) speak Swahili; the prime language employed during the interviews, and could therefore scrutinize the content and quality of the translated texts.

For paper III, responses from the open-ended questions were hand-written in Kiswahili or English depending on the language used during the interview. The observation notes were written down by the first author in English. Preparation of the data for analysis through translation to English from Swahili of the already written responses and typing the responses in Ms Word preceded the actual analysis.

Framework analysis:

Framework analysis was employed for the data acquired through FGDs (1B and 2C) and IDIs (2D) in paper II. This paper aimed at generating knowledge on experiences and perceptions with the implementation of the new HIV testing guidelines (the provider initiated opt out approach in PMTCT programmes). Framework analysis is a qualitative analysis that is specifically used and recommended for guiding analysis for applied research seeking to advice policy. It proposes a step-by-step framework as a guide through the analytical process [95].

In the process of analysis recurring themes or patterns as well as variation, nuances and contradictory or deviant cases, were sought during the review of the transcripts. A pattern quickly emerged revealing substantial frustration with the manner in which the provider initiated testing and counselling (PITC) was implemented. Statements from the informants that illustrated this point in most diverse ways were thus sought, including a search for deviant cases. The process of identifying the concrete quotes employed in the manuscript was extremely time-consuming as we wished to ensure that the statements emerged as 'representative' or 'typical' of the sentiments that were expressed. This phase involved all the five authors of paper II over quite a period of time, albeit to differing extents.

The process of handling the data for paper II using framework analysis can be divided into five steps: 'familiarization', 'identification of its framework', 'indexing', 'charting' and

‘interpretation’ [95, 96]. A few details relating to each of the steps and the manner in which we dealt with them are presented below:

Familiarization: This stage involved total immersion in the ‘raw’ data through listening to the tapes (for data set 2C & 2D), primarily through the thorough reading and reviewing of the full set of transcripts several times. This was a time-consuming task as the collected material was substantial, but it was a vital part in order to gain a sense of the overall content and topics covered by the interviews. This first step primarily involved three of the authors of paper II (MKN, AB and ES), with frequent consultations with the data collectors of the REACT data (1B). All co-authors of paper II had been part of the planning of the cross-country part of the REACT study, including the development of the interview guides. Relating to the sub-study in Malindi, familiarization with the data took place at this stage through a review of the transcripts, i.e. continuously through the data collection process, with a more systematic phase taking place after the translations of all the transcripts had been completed.

Identification of a thematic framework: The issues that emerged regarding experiences with HIV testing identified during the *familiarization stage* were listed in a word document. The main themes were: 1) the value of counselling in HIV services as experienced and perceived by the study informants, and 2) the challenges experienced by the informants with the PITC model in the HIV services. Both of the themes had numerous sub-themes that were listed in the document under each main category. This created an overall thematic framework that was drawn upon in further analysis of the data.

Indexing: In step three, the process of a thorough review of all the transcripts (of data sets1B, 2C & 2D) took place. The main themes were noted in the left-hand margins of the transcripts, with more detailed summarizing statements of the ways in which the theme were addressed at the right-hand side of the margins (often using a short quote from an informant to sum up the specifics of the content).

Charting: The material that was identified through the stage of indexing (the broader themes and the particularities/nuances of the informants’ revelations) was re-arranged at this point and placed in large charts. The information was eventually grouped in three columns: column one) the theme, i.e. the larger topic identified, column two) the cases or quotes (pieces of texts) from the interviews identified as particularly important under specific themes or summaries of

the findings, and column three) condensed versions of column two that were based on the summarized versions written in the right-hand margins of the transcripts. The chart was reviewed and discussed among the co-authors.

Interpretation: this took place throughout the entire research process from the interviews carried out in Malindi district until the last word had been written. With regards to the data from the first set of FGDs (1B), this process started during the familiarization stage. A major effort was put into identifying and discussing the thematic framework, and later the indexed material, i.e. the content of the large charts. At various stages in the analysis, smaller and larger groups of the co-authors convened to discuss the content of the charts and decide the material to be presented in paper II.

Thematic analysis:

Thematic analysis is said to be the most commonly used qualitative analysis in healthcare research [92]. The actual process did not emerge as different from framework analysis as it involves the grouping of data into themes created from recurring codes that are similar or connected to each other [92, 97]. This approach was employed during the analysis of data acquired through the open ended question (2B) that were used as the basis for Paper III. The analysis involved five major steps namely: 'familiarization with the material', 'generation of codes', 'searching for themes', 'revision of the themes' and 'interpretation' [98]. A limitation of this method is the lack of consideration of salient codes that do not recur [98]. The potential challenge was however not experienced as problematic because this particular sub-study focused on the 'responsiveness' concept in relation to VCT.

Familiarization with the data: This phase started during data collection. It was considered important to become thoroughly familiar with the material while I was working in the field with a small team. This allowed me to discuss the content which provided further guidance in both data collection and analysis of the data. Details of the content of the material were discussed right after the interviews and during the evenings. Post- fieldwork analysis involved reading repeatedly through all the transcripts of the collected material implying a systematic immersion in the data.

Generating initial codes: This stage of analysis began along with familiarization. It entailed re-reading the text and coding the material. A table was created on MS Word where the initial codes were noted down in long lists. The list of initial codes was rough and fairly unorganized,

but allowed a first attempt at noting down the main topic and themes that emerged from the interviews.

Searching for themes: Later, processes worked with the list of codes, as well as with the transcripts, i.e. with processes that involved new rounds of review. The list of the codes that were generated in the previous stage was revised and refined and more nuanced lists were created. In the left column was a list of major themes, in the middle section the relevant quotes/narratives (now de-contextualized from larger interviews) were located, and on the right side the codes related to every quote were placed. The relevant pieces of text were highlighted and extracted by cutting and pasting before being placed under the relevant themes. This stage involved both creating an increasing number of nuanced codes, as well as collating those that we considered similar, such as for example "*privacy"* and *"confidentiality*". The major themes remained similar to the responsiveness elements, but other more detailed themes were identified and added.

Reviewing and defining the themes: This stage entailed refining of the themes that had been identified. The process involved all the co-authors of the paper who read through the themes to generate concise and clear names for each theme. Most themes remained under the defined WHO responsiveness elements, but additional issues such as "c*ontinuity"* and *"follow-up"* were added.

Interpretations: Interpretation took place throughout the entire analysis process and stages (from the data collection phase through to the reviewing and defining of the themes stage*)* on to the identification of the more refined, nuanced themes and codes developed at later stages in the process. Through thorough reflection of the emerging codes, a particularly intense period of interpretation involved two persons (MKN and AB), both authors of paper III. When there was uncertainty, revision rounds involved a larger group of authors.

3.9 Ethical Clearance

Ethical clearance was obtained from the three countries. In Kenya, scientific and ethical approval was obtained from the Kenya Medical Research Institute (KEMRI) and the National Ethical Review Committee (NERC) (KEMRI SSC, number 1096). The sub-study in Malindi was granted a separate ethical approval by the same institutions (KEMRI SSC, number 1273). In Tanzania, research clearance was obtained from the Medical Research Coordinating

Committee (MRCC) of the National Institute of Medical Research (NIMR) number (NIMR/HQ/R.8a/Vol.IX/416), and in Zambia, from the University of Zambia Research Ethics Committee (assurance No. FWA00000338, IRB00001131 of IOR0000774). Written informed consent was obtained from all participants of the population-based survey prior to being interviewed. Verbal informed consent was obtained from all the study informants prior to the interviews. Confidentiality and anonymity of the study informants was maintained throughout the study.

4 RESULTS

4.1 PAPER I:

Inequalities in HIV testing exposure: results from population based surveys in Kenya, Tanzania and Zambia.

This study sought to determine factors associated with VCT exposure for HIV testing. A total of 5689 (i.e.1847 Malindi, Kenya, 1992 Mbarali, Tanzania and 1850 Kapiri Mposhi, Zambia) people in the three districts participated in the survey. The majority of the participants (77% N=5689) indicated that they knew a place where they could test for HIV but districts differed in this regard, i.e. Malindi 84.6%, Kapiri Mposhi 80.3% and Mbarali 67%. There was also a marked difference in readiness to be tested between the districts, i.e. 52.4% in Malindi, 83.3% in Mbarali and 46.1% in Kapiri Mposhi. However, the proportions reporting use of VCT or PMTCT for HIV testing was very low (20% VCT), considering the overall sample size n=5689 in the three districts.

This paper indicates that among the determinants of VCT, stigma and education emerged as the main factors associated with the use of VCT. These were indicated as consistent in all the three districts and in the full model that combined the three districts (N=5689), where those reporting higher levels of stigma were less likely to use VCT (AOR 0.6. 95% CI: 0.43-0.85). Overall (N=5689), there were inequalities in the use of VCT-based testing in relation to education. Those with higher levels of education were significantly more likely to use VCT (AOR 1.8, 95% CI 1.46-2.32). The positive association observed between education and use of VCT was consistent in the three study districts, and among both men and women. Furthermore, the results indicate a negative association between education and stigma (AOR 0.4, 95% CI 0.29-0.45), as indicated in figure 6 below.

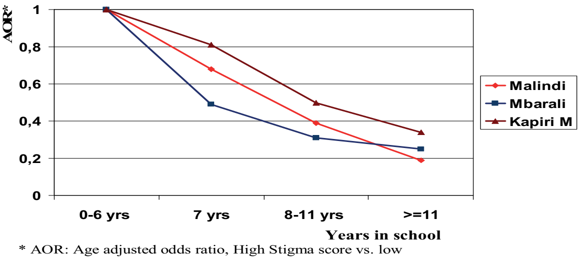

Figure 6: The association between stigma and educational attainment

4.2 *PAPER II:*

Practicing provider-initiated HIV testing in high prevalence settings: Consent concerns and missed preventive opportunities

The Proportions of the population that had ever been tested for HIV differed sharply between the study districts and particularly among women (54 vs. 27%) considering the extremes in the three districts. Women were much more likely to be tested than men in the districts that had scaled-up programmes for preventing mother-to-child transmission. Only minor gender differences appeared for VCT use.

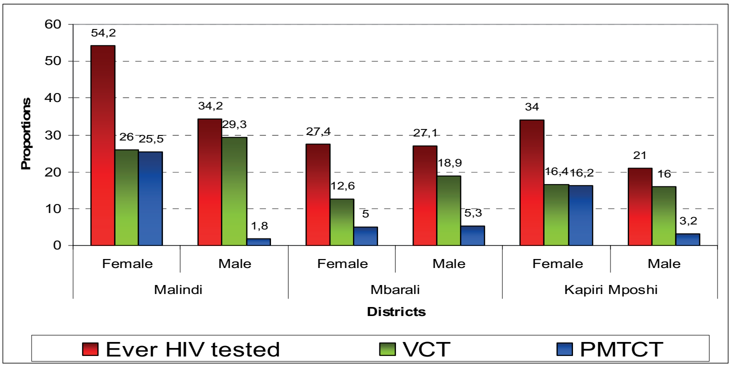

Figure 7: Proportions testing for HIV and proportions testing at VCT and PMTCT.

During the qualitative interviews, the counselling concept was expressed as an essential and highly valued part of HIV testing for preventive and support aspects. However, in Malindi district where the provider-initiated opt-out HIV testing model in PMTCT programmes had been rolled out extensively, HIV testing was accompanied by very limited pre-test counselling. The uneasiness with which this modification was met is indicated in the following quote by one of the informants:

"During the second pregnancy we were not given a choice. It was a must to get tested on HIV and then (after that) on the pregnancy. We were not asked; you enter the room for HIV testing and then you go for other tests. To tell you the truth, some there got quite scared that day when we were suddenly tested. People panicked a lot. So people were not happy, but it was a must that they do it." (Female 35, IDI, urban Malindi).

In the same district, post-test counselling in the opt-out approaches to testing at the ANC settings was experienced as very limited. The counselling focused mainly on the one who tested positive as expressed in the following quote.

"*They counsel you only when you are found to be HIV positive, but if you are not HIV positive you just get your results and go.* (Female pregnant, FGD, rural Malindi)

In the other two districts pre- and post-test counselling was reported to be practiced during HIV testing at the antenatal clinics.

The figure below gives a summary on the counselling experiences as with VCT and PMTCT based testing

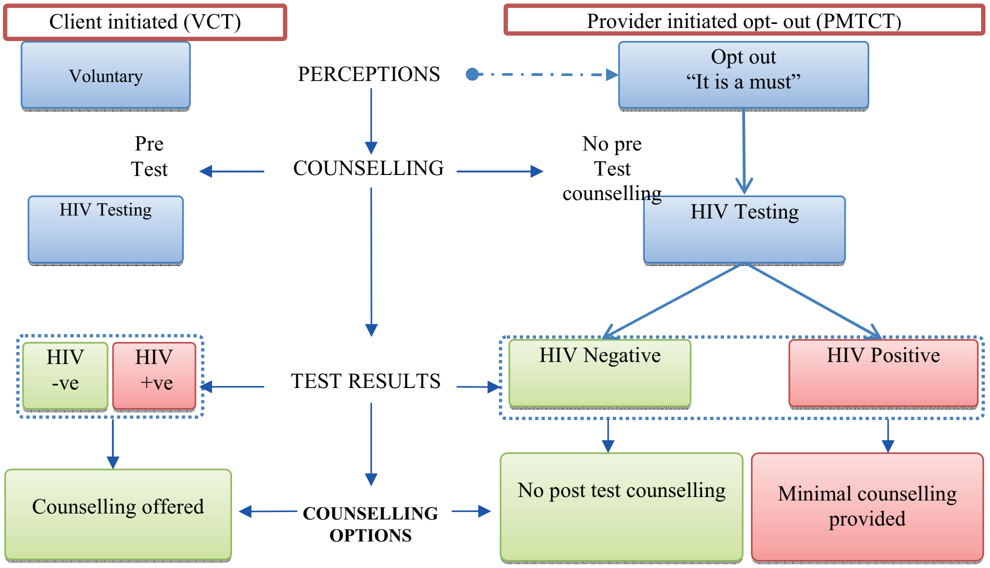

Figure 8: Counselling during HIV testing at PMTCT and VCT as experienced by the informants

The results from the population-based survey indicated that women were tested more than men, a difference which was more marked in the Malindi district. There was an expressed burden on women as they were made responsible to inform and bring their spouses to the clinics for testing at a vulnerable time in their lives. Informants expressed the need for the involvement of health personnel in bringing their spouses to test, as noted in the following quote:

"These counsellors should be many to help us because we are wives, and when you ask your husband to go to test himself he stays quiet refusing to talk. He tells you 'you get tested, if you are found to be ok, I am also ok'. He does not go." (Female pregnant, FGD, urban Malindi*)*

4.3 PAPER III:

A critical assessment of the WHO responsiveness tool: Lessons from voluntary HIV testing and counselling services in Kenya

A majority of the 'health providers' and 'users' interviewed indicated that the elements proposed by WHO to measure responsiveness were very important. E.g. confidentiality (97% users & 94% health providers) and autonomy (93% users & 83% health providers) were rated as very important by a high proportion of our respondents, and were moreover reported as aspects that were highly observed in the VCT room. However, the qualitative findings revealed aspects related to confidentiality, autonomy and other responsiveness elements that did not seem to be captured by the WHO tool. While 98% of the VCT users reported that confidentiality was highly observed, the qualitative data found issues regarding this element that were not captured by the quantitative responsiveness tool. An example in regards to this was the experienced inappropriate location of the VCT room, as expressed in the quotation:

> *"The VCT room should not be located next to the consultation room for confidentiality reasons. It should be situated in a private area where one is not seen by everyone when entering or coming out" (a 38 year female urban VCT).*

Although the majority reported that autonomy was observed during the VCT sessions, the qualitative data revealed that very little information was received on which to make an informed decision and that there was need for using local languages so that everyone could understand the information being given, as stated by a 18 year old female VCT user:
"*The counsellors should be able to speak the local languages because they give us a lot of information. As for me, I did understand some things but not all that the counsellor said"*

The qualitative findings thus revealed aspects that did not seem to be captured by the responsiveness tool but were considered vital when assessing VCT. The qualitative inquiry also identified other aspects such as access to social support, continuity of care or follow-up, and the quality of counselling and testing that were not well assessed by the responsiveness tool, but were experienced to be of relevance in assessing responsiveness.

5 DISCUSSION

5.1 Discussion of Methods

Assessment of the mixed methods approach

Two types of mixed methods designs were employed in this study. These were concurrent triangulation and a concurrent nested. The first was applied to paper II and the latter in paper III. In paper II, data analyses of the quantitative and qualitative data sets were carried out during the same phase starting with the quantitative results of the PBS data, followed by the qualitative findings. This design enabled us to determine the proportion of the population having used VCT and PMTCT based opt-out testing and to simultaneously explore people's experiences with the testing approaches with an emphasis on the provider initiated opt-out strategy, in a single study.

Paper II utilises four different data sets partly collected by different people. The data sets are the population-based survey (1A), two sets of FGDs (1B and 2C) and IDIs (2D), i.e. qualitative and quantitative data collected within the REACT project and two sets of qualitative data collected in Malindi. Data collection was conducted by people with relevant expertise, within epidemiology and social science (anthropology and sociology). Extensive training was carried out within the REACT project to 'calibrate' or coordinate tools and the approach of the data collectors. The major challenge with data set 2A was that I was not present to coordinate the data sets neither was I involved in the data collection. It was therefore imperative for me to communicate with the data collectors extensively during the data analysis. As described in the methods section, I was part of the data collection process in Malindi (2C and 2D), and I followed the data collection process of the REACT material through participation in the project and discussions with the data collectors.
However, there are weaknesses linked to such a complex design. Despite the highly trained data collectors, common interview guides and topic guides (used in the three REACT countries) and training, one cannot secure that concepts and approaches do not differ between professionals and setting. In fact, it is clear that each qualitative interview and focus groups will gain slightly different content depending on participants, contexts and data collectors. Probing in the qualitative interviews and focus group discussions will by necessity differ somewhat between the interviews. We noted that some of the qualitative interviews and FGDs had more 'rich information' or more articulate informants. Some of the topics were followed up by more probing, and thus provided richer description of the issues at stake. The

comparison between the qualitative data sets has thus taken place with considerable caution as to what extent one can draw broader patterns and relevance. We can obviously not generalize in a quantitative sense on the basis of these data sets.

The concurrent nested design applied in paper III differed from the concurrent triangulation in that the data (quantitative and qualitative) were collected in one phase with the same study participants. The predominant method was quantitative close ended questions but with one open-ended question included. The study design allowed the researchers to collect data simultaneously facilitating a broader understanding of responsiveness related to VCT use as viewed by the study participants in Malindi District. The design also allowed the researchers to question the same study participants using two different techniques. This appeared to be essential in identifying potential methodological or measurement biases.

A weakness of employing an extensive data collection tool which includes an open-ended option is that it can exhaust the study participants. The magnitude of this challenge cannot be assessed in this study. However, the fact that 64 participants declined from responding to the open-ended question (39 indicating they were satisfied with the VCT operations and 25 not wishing to participate further), can be an indication of the timing issue (about 30 minutes and more for the open-ended question in addition, to the 45-60 minutes used in the closed questions). The decline to respond to the open-ended question could also be linked to shyness of speaking more openly, or a fear of speaking more openly about the topic in question.

Applying mixing methods is challenging, and it does not by itself guarantee that the results will be strengthened. They should be used only when necessary [87, 88]. Experience with the use of mixed methods design in this study proved a valuable process of producing in-depth understanding of the complexities tied to HIV testing. A vast amount of topics and nuances of topics emerged during the qualitative inquiry. The surveys on the other hand allowed us to quantify some of the same questions thus providing both quality and depth. We believe the mixed methods approach enabled us generate data that would not have been obtained by a single method thus, increasing the awareness of the challenges linked to HIV testing strategies.

For time reasons, a concurrent design was chosen in the studies. It is likely that a sequential design would have yielded information that could have led to an earlier narrowing or focusing of the topics concentrated on in this thesis. A sequential design could have yielded more

detailed responses to the concerns we concentrated on in the three articles. This could have led to further in-depth exploration of, for example, the challenges or benefits with opt-out HIV testing approaches, allowing us to collect data even with the health providers to present their views.

There is a potential danger that collection of large data that may not be directly used or related to the different data sets may be encountered within concurrent designs. Hence, the potential waste of time in the collection and analysis of information that will not directly employed in the writings. We did end up with large amounts of qualitative data that were not directly employed in the three articles. I will argue that the broad approach chosen in the qualitative interviews was extremely beneficial. It provided the researcher with knowledge of the vast range of challenges connected to HIV testing, providing a broad understanding of the context within HIV testing.

Another key challenge with mixed methods designs emerges during the interpretation of the data. The potential challenges were found to be complex in terms of how we could reasonably relate the different data sets i.e. how the various sets of data and results could possibly inform or question the other. This was mainly a challenge in paper II where the qualitative themes (e.g. counselling, burden on women) were not matched by the quantitative. The process of presenting the data was also challenging (in a sequential manner or simultaneous manner following the diverse results and questions raised). Suggested approaches of interpreting mixed methods are: making inferences from the findings of each of the methods and bringing those inferences together at the discussion section or, bringing the findings from each of the methods in the discussion of the inferences [99]. The results in Papers II & III are presented in a manner where the various data sets inform each other in best possible ways, while still attempting to retain the differences between the data sets. In paper II, we analysed the two data sets and presented the findings separately in the results sections and merged the inferences in the discussion section. In Paper III, the quantitative and qualitative themes were moderately comparable, making it possible to present findings from the two methods within the themes. This was further expounded on in the discussion section.

The question of the length of papers was further challenging as both methods-, results- and discussion sections by necessity demand more space in mixed methods design. Another challenge encountered is the use of terminology which appears as rather confusing when the

terminology is to follow the acknowledged practice in the qualitative and quantitative approaches respectively. For example, terms commonly employed for study participants (respondents and informants respectively), inclusion of study participants (sampling and recruitment) creates a complex and potentially confusing picture, particularly when the material is to be presented in a concise article format. The landscape of methods terminology is not quite clear particularly within the qualitative research approaches where there are substantial debates about what terminology best captures the processes involved.

There are also more general issues related to mixed methods designs which still remain not well developed. A number of questions remain to be addressed and discussed in the literature. The call for separate descriptions and discussion of the methods has been accentuated, however, fundamental questions raised on whether mixed methods stands as a separate paradigm, need to be resolved [100].

Quantitative assessment (cross- sectional surveys)

Cross-sectional design was used in the population-based survey in the three districts, and in the facility-based survey in one of the districts. The population-based survey was conducted to provide baseline data for the REACT intervention study and was based on a representative sample of all adults in the three districts. Information was therefore, collected on a wide range of topics and included questions related to HIV testing (Papers I and II). The second study was conducted among VCT clients by employing a pre-tested evaluation tool on responsiveness (Paper III). By their design type, cross-sectional studies do not generate a sequence of events, making it difficult to establish causal relationships. The results from the analytical approach aimed at examining factors affecting VCT use (Paper I) should therefore, be interpreted with caution. Longitudinal designs would have been the gold-standard design; however, these designs are expensive and encounter serious biases (e.g. non-participation bias). Cross-sectional data are frequently used for this type of analysis and often yield results that are similar to those from longitudinal designs

Validity

Validity in epidemiology is defined as the lack of systematic error. It entails the entire study concept and establishes whether the results obtained meet internal and/or external validity. Internal validity looks at whether the study measured what it was intended to measure,

whereas external validity deals with whether the study findings can be generalized to the wider population [101].

Internal validity

To attain internal validity, accuracy of the inferences made in a study is vital. In this regard, various types of biases exist that can undermine internal validity. These biases are mainly in three categories, namely: selection bias, information bias, and confounding factors [101, 102].

Selection bias can result from improper selection of participants, e.g. the use of volunteers or self-selection, not using the same criteria in selecting all study participants and whether non-response differs from those that respond [101]. In Papers I and II where the population-based survey was used to determine factors associated with the use of VCT, the participants were selected based on probability sampling in all three districts; non-response was limited and did not differ by district or setting (rural/urban). In Paper III assessing responsiveness elements, we selected all clients of VCT during a given period of time. Refusal was limited, and the study participants were likely to be representative of VCT clients in the Malindi district.

Information bias can occur during the data collection phase, and when the effect is distorted by an error in measurement or misclassification of the responses [103]. Papers I and II used the population-based survey. Some of the biases could have been due to misclassification of the study participants. It is possible that questions on exposure to HIV testing can be affected by reporting bias due to the stigma attached to HIV. Efforts were made to minimise these biases as respondents were informed that their HIV status was not important in this study. Moreover, confidentiality and privacy were ensured when the questions were being asked. Some of the independent variables in the model employed in Paper I were based on relatively sensitive questions and could have generated response bias for social desirability. The most likely case is the indicator used for sexual risk behaviour, which is substantially under-reported, especially among women [103]. This bias is probably substantial, and the effect will be a dilution of the association.

Confounding is the mixing of effects where confusion in the results is brought about by an intricate relationship between independent variables and the outcome variable. An example in our study (Paper I) would be education attainment and stigma. Both these variables were associated with VCT use, with a strong mutual association. Potential confounding was

considered in our study and controlled by stratified analysis (Papers I & II) and multi-variable analysis using the Andersen model for Paper I, thereby comparing the crude and adjusted findings of VCT use.

External validity

For the population-based survey results, the question is mainly about the extent to which we can generalise above the three districts. The analyses of determinants of VCT showed great cross-country similarities, which suggests that the key findings might have regional generalizability or at least can be generalized to settings with severe HIV epidemics. In an attempt to capture views from most users in Paper III, we sampled all VCT facilities in the districts and all (users) that used these facilities over a month. These results (Paper III) probably apply to other VCT users in the district, and to VCT users in the country and similar settings.

Qualitative assessment

The qualitative research approaches in this study intended to capture the depth and detail related to HIV testing strategies and on the context in which testing takes place on a general level. More specifically, it sought to generate knowledge on perceptions and experiences with the HIV testing strategies (paper II) and diverse aspects related to the challenge of assessing responsiveness of health systems in the context of VCT (paper III). While the strengths and potentials of qualitative methods in generating materials with depth due to their inherent flexibility are quite clear, the approaches also have potential challenges and weakness not the least where the researcher is the primary instrument [104].

The aim of ensuring validity remains a central and desirable principle also in qualitative methods [91, 105]. The term '*credibility*' however, seems to capture the virtue of quality one is aiming for in a better way and is commonly employed in qualitative studies [91]. 'Relevance' or 'transferability', rather than generalizability, is another key quality aimed at, and will be addressed in the next sections.

Credibility

Mays and Pope describe ways of improving validity as triangulation, informant validation, clear exposition of the data collection methods and the analysis process, reflexivity, attention to 'negative' or 'contradictory cases and 'fair dealing' [105]. In both Papers II and III,

triangulation of methods was applied. In paper II the use of methods triangulation - using more than one approach to explore a phenomenon - as well as source triangulation - interviewing more than one category of informants about the same issue (differing in terms of age, gender and pregnant status) was used to obtain a broader and more in depth understanding of the phenomenon at stake. In paper III we also employed methods and source triangulation. Source triangulation in this context implied interviewing both users of the VCT and health providers (counsellors). The extensive methods triangulation implied the use of both quantitative (1A and 2A) and qualitative (1B, 2B, 2C, 2D and 2E) data sets for Papers II and III. The use of different methods to collect the data yielded a rich and detailed material that revealed the diverse aspects and angles of HIV testing. This enhanced our overall knowledge of this complex and challenging health service.

The methods triangulation strongly enhanced the validity of the data. Observations increased understanding of the informants' stated uneasiness with the location of the testing facilities in paper III. The in-depth interviews increased the depth of response brought up in the focus group discussions. At the same time, the different approaches have questioned the validity of certain responses. The different data sets have generated material that give reasons for questioning some of the findings collected with other methods, especially between the quantitative results and qualitative findings. An example in this case was the high observant of the responsiveness elements e.g. confidentiality and autonomy during the VCT process from the quantitative findings (paper III). Nevertheless the qualitative findings revealed additional relevant aspects within the same elements that were not captured by the tool. While generating informative and rich material, the potential pitfall of having to relate and properly handle the interpretation process of such vast material is apparent.

In all the focus group discussions, we grouped the informants according to sex and age as is the general norm to: create groups with relatively 'similar' backgrounds so as to enhance the chance for people to speak freely. The gender based division of the groups however, faced criticism by some of our informants in rural Malindi, as the informants felt that having a diverse group (men and women together) could have yielded an even more interesting discussion. In this study, attempts were made for clarity about the ways in which the data collection and analysis of the data took place. There are however, always potentials for other ways of viewing and presenting research findings. In this case, the process of analysis and

interpretation and the ways in which to present the material was discussed in quite some detail among the co-authors of Papers II and III.

Reflexivity in this study implied substantial consideration to ways in which the researcher, the study assistants and the research process potentially shape the data collected. Both the data collection phase and the interpretation of the data can and will to varying extent be influenced by the researcher's background, assumptions, perspectives or experiences [105]. Although the development of the study tools for the first set of FGDs (1B) in paper II was carried out by qualified and experienced researchers from social science and epidemiology (involved in the REACT project), only one of the authors (ES) of this paper was involved in the actual collection of this first set of FGDs. It is a limitation that the author of this thesis was not involved in the data collection of this set of focus group discussions, with the lack of potential for own probing and follow up during the interviews (FGDs 1B). The co-authors of paper II together with myself did however try to compensate to the extent possible by being intensely involved in the analyses of this data set. The data analysis was conducted jointly with several researchers (some of whom are co-authors) and in collaboration with those who collected the data seeking clarifications where data did not emerge as clear. The fact that all the interviews were recorded and transcribed word by word facilitated a sense of closeness to the data, although I am aware that the relation to this data set cannot be the same as the relation to the other data sets.

In the second data set employed in paper II (FGDs, 2C, and IDIs, 2D), I led the research process (from planning, data collection, data analysis and interpretation of findings). In addition to my own presence several experienced researchers were involved at all stages of this research process as well. Although drafts to the interview and topic guides were developed prior to the study, the research was guided by an open mind that left the possibility to modify the guides in the course of the research process. Modifications were carried out primarily through the first phase of the data collection of this study

In qualitative studies, the researcher is the 'instrument'. The author of this thesis was the principle researcher. As a woman, single, Kenyan but does not originate from the study area (Malindi) holding a research position at the Kenya Medical Research institute (KEMRI) meant that I was clearly not a typical inhabitant in the study area. To what extent is it likely to assume that these attributes influenced the study findings from Malindi district? Although

clearly an outsider, the researcher believes that by virtue of being a Kenyan and being able to speak Swahili (which is not only the national language but the prime language in this area) brought her close to the informants. Hence she was able to pick crucial messages that were passed in this language. It was sensed that there were expectations for some form of reimbursements upon participation since we were at times introduced as the 'project people' from KEMRI. The researcher and her assistants were very sensitive to the language used, the dress code (we wore long dresses and tied a 'kanga' a local cloth used by women that is tied around the waist) and our conduct, and thus tried to minimize the differences. We used public transport during the entire study period.

Not being natives of Malindi was however at times also experienced as an advantage. Our experience was that as outsiders people felt free to report issues or cases from the health facilities, as they felt certain that we did not know either persons or settings. Often we also experienced that our focus group participants got so engrossed in the topics of discussion amongst themselves that they seemed to forget that we were part of these sessions. What is more, some did indicate that more time was needed to discuss these issues as people became engaged and interested in the discussion.

Being a single woman may in this setting imply that you are not 'old enough' or 'have not lived long enough' to have the experience or even to be taken seriously by the society, especially if you have no children. It was therefore paramount to seek assistance from an experienced social scientist who was married and a mother. During the focus group discussions we did share the responsibility for asking questions and for probing.

Coding validation of the two data sets was done by seeking consensus among the ones involved in the process of analysis. If disagreement of interpretation occurred, we either dropped further pursuing of the issue or we presented the possible interpretations of the finding. An example was the disagreement that the following statement implied counselling versus information. "*All I can say is that people need information when testing, otherwise you just blame yourself.*" (A Male adult. Out-patient, FGD. Consensus was not reached here. The quote was presented to indicate the need for counselling. However, one of the co-authors failed to agree that it could be interpreted as counselling, and we dropped the quote. We also had rounds of discussion about what was problematic with the opt-out testing strategy. the issue was whether it was the guidelines that were problematic or the manner the guidelines

were implemented both among the co-author and the reviewers of paper II. We have reason to believe that the extensive discussion in the group, the critical reflection and the time put into the interpretation processes led to minimised assumptions and misinterpretations.

Transferability

Transferability relates to what quantitative approaches refer to as external validity. It should be noted that qualitative studies do not aim at generalizing the data collected in the strict quantitative sense. The findings in principle are applicable only to the specific settings they have been collected [91]. Although paper II does report findings from the three REACT districts, Malindi district had extensively scaled up the PITC opt-out approach of testing at the time of study. Hence, more relevant experience could be gathered from this setting. This limits the transferability of these findings to other REACT districts. Nevertheless, the transferability can be enhanced by the fact that similar findings have been recorded in other studies [106, 107]

The findings in paper III regarding the areas that did not seem to be fully covered by WHO's 'responsiveness tool' may be of relevance for other African settings. The issue at stake boils downs to a lack of sensitivity to stigma related concerns connected to the broader physical context of the VCT facilities, findings that also have been common in studies from the African continent [32, 108, 109]

5.2 Discussion of Main findings

In the coming sections, I briefly discuss the study's main findings starting with findings on HIV testing exposures and experiences from the second paper, followed by determinants of VCT use from the first paper and close with a discussion of the findings related to responsiveness of the third paper. The discussion also attempts to address cross cutting issues of relevance in all the papers.

HIV testing exposures and experiences

Marked differences in HIV testing exposures were revealed both by place and sex. Higher HIV testing exposure levels were reported among women in two of the districts (Malindi and Kapiri Mposhi) compared to the men, a finding that is consistent with recent country surveys [51, 110, 111]. This finding contrasts with the existing literature on HIV testing, when VCT is the main mode of testing men have been documented to be more likely to use VCT than

women [32, 112, 113]. The sudden increase in access to HIV testing among women is largely explained by the scaling up of the PITC opt-out strategy at the ANC settings that exposes all pregnant women attending these clinics to the HIV test.

Whereas this increase in testing can be quickly interpreted as a success in knowledge of own HIV status, findings from qualitative data sets of this study revealed disturbing experiences with the PITC opt-out approach to testing. For example, a lack of involvement in the consent process emerged from the findings of Malindi district where the opt-out strategy had been scaled-up extensively. The PITC model is initiated by the health provider hence consent can be problematic given the power differentials between patients and health providers in health care settings. In this study, it was clear that the users did not feel enabled to make own decision on testing, making it uncertain how consent was obtained. The implementation of the PITC opt-out model at the ANC settings seem to fuel gender inequities in that women are more exposed to the test than men. This consequently places uneasiness on women as the informants did reveal the burden placed on women by the health system being the ones asked to disclose their HIV positive status to their husbands, not the least to recruit their spouses for testing at an unusually vulnerable moment in time; at the time of pregnancy.

The findings on HIV test exposures were self reported and we did not confirm the test in this study. Accordingly, this could have introduced some misclassification bias. The most likely scenario is under-reporting; hence examining determinants in the model could have diluted associations. The study on experiences with the opt-out HIV testing model were only conducted in Malindi district, hence the findings regarding this limit us to just the Malindi community. However, recent studies now show similar experiences in other countries which could be an indication that these findings have relevance in similar contexts [46, 106, 107].

Counselling

The study findings presented in paper II indicate that high value is placed on HIV counselling. The inclusion of the pre and post test counselling in VCT was highly appreciated by the informants. It was talked of both as an important tool for boosting prevention and support for those infected. Given the potential benefits and the high value placed on counselling, the decision to diminish the importance of counselling in the recent HIV testing guidelines [26] emerges as problematic. Not the least the question emerges as in disagreement with visions of

a primary health care strategy that values the strengthening of the health systems through putting people at the centre of health care [114].

Pre-test counselling has been an issue of concern since the early debates on HIV testing. It has remained a matter of concern among other reasons in a context of promoting autonomy, i.e. the experience that the individual is taking the decision on testing. Proponents of routine testing have argued that pre-test counselling introduces inessential elements, some of which are also considered deterrent towards reaching the objective of normalising HIV testing. It is further claimed that pre-test counselling spends unnecessary large amounts of time in a manner that can instil fear on people rather than merely inform them, and may thus discourage them from being tested [36, 115]. On the basis of this study's findings, it is clear that fear may also be caused by no or rushed information sessions prior to testing. Such brief periods of introduction (pre-test information) to the testing session are now being promoted in the new guidelines.

Another aspect of the new policies on HIV testing approach has been linked to the concern that health systems in low income contexts are grappling with meagre resources, not the least being human resources, a resource that is highly required in delivering VCT services [116, 117]. The relationship between the health care providers and clients is however extremely vulnerable in an HIV context. On the basis of this study, reducing the time spent on counselling given to individuals going for HIV testing, in order to save resources and to meet health systems challenges emerge as problematic. This is so because the information and education presented to the clients emerged as vital in a context of client autonomy. Findings on experienced mandatory testing often phrased as 'it is a must' in the present study supports rising evidence from the same in the region, for example as presented in recent studies conducted in Uganda and Botswana [46, 106]. The opt-out testing model was praised for yielding higher test rates in the Botswana study which is a highly important dimension and aim in this context. However, in the same study, concerns were raised as to the manner this was achieved as a majority of the ones who took the test indicated that they were simply not able to refuse the test [46]. The potential impacts of receiving an HIV positive test result at a point in time when one does not feel prepared, can be highly problematic in handling of the situation [118].

The WHO/UNAIDS guidelines on HIV testing does maintain that post test counselling is necessary for HIV testing [26]. Our study findings however show that at the time of implementation there was a lack of post test counselling for clients testing negative. Post-test counselling offers the counsellor a chance to discuss with those who test HIV negative in order to encourage them to reduce risk in infection in a manner that is tuned to individual levels and demands. Research demonstrates that counselling can influence behavioural change. In particular post test- counselling has been documented to be effective in increasing condom use among discordant couples and child bearing women [25, 119-121]. Substantial efforts have in recent years naturally been focused on treatment. In this immensely important effort, prevention has however suffered. Prevention remains vital however in bringing about a reduction in the number of new infections. [122, 123]. Considering the individual and public health benefits that accompany HIV related counselling, the recent reduction of the importance of these services emerge as problematic as this study and the current literature indicate, several problematic ethical dimensions [47, 49, 106, 107]. This issue needs to be addressed so that the services are provided in a manner that ensures both increasing test rates and ART uptakes while retaining its vital preventive dimension and the individual's final possibility to either decline or take up the test.

The burden on women

Exposing pregnant women to test for HIV comes with good intention. The ante-natal clinics (ANC) provide an easy and unique opportunity to reach the pregnant women who are particularly exposed to the HIV risk. This is important both for the sake of the women as well as a vital mode to prevent vertical transmission. The concern of reaching women through ANC was raised as early as the mid 1990's, and has since been reported to be successful in increasing the proportions of women testing for HIV and receiving prevention of mother to child transmission services (PMTCT) [41, 124].

Our study adds to the evidence of more women than men having tested when the PMTCT program with an opt-out approach to testing is introduced. The well intentioned program does however have problematic outcomes. Exposing only women to testing in women centred programs in contexts where men have long since been known to be key to decisions making in a broad sense, has emerged as problematic. While these infant centred programs will importantly benefit the unborn and new born babies in preventing HIV, the fundamental lack of incorporation of the consideration of women's positions in both an economic, practical,

social, cultural and political way has been brought up in various recent writings [118, 125-127]. Some of the objections raised have been linked to the problem that the key decision makers in the family are commonly not involved in the decision making regarding the infant or the baby. The outcomes of the challenges met have led to immense uncertainty and suffering of the women,

The recent lack of post-test counselling of HIV negative women in the PMTCT programs seen in the implementation of the opt-out approaches to HIV testing could add to the many and complex challenges reported from PMTCT programs. A study in south Africa reported that pregnant women had four times the transmission risk compared to the non-pregnant women [128], indicating the immense risk this phase in a woman's life implies and the linked emergency of providing appropriate counselling and guidance to this category of women. The simultaneous and serious focus on horizontal transmission and vertical transmission in PMTCT programs seems ethical and vital in promoting health.

The findings in the present study further unleash women's experienced burden being the ones who are to encourage their spouses to go for HIV testing. Literature has shown that exposing women to testing and to pressures of disclosure to spouses, in some setting has led to domestic violence [47, 64, 129]. Such experiences have led to fear causing women to decide not to disclose their status. Not disclosing the test results has been reported among a high number of women (up to 86%) [130]. A blame has been placed on the PMTCT program in this context as it particularly exposes women to testing and to knowledge of own HIV status. A PMTCT program in Malawi was referred to as the 'divorce program' by the informants due to the high rates of marital conflict and divorce upon the disclosure of HIV positive status, in accordance with the programs' policy [118]. The burden on women in disclosing their status has been strongly enhanced by the PMTCT opt-out approach, as women who do not wish to get tested nonetheless end up being tested. Increased pressure is experienced when women are informed that no other tests at the ANC can be conducted if they refuse the HIV test and when they are made responsible to bring their spouses to undergo the HIV test. Innovative ways to include spouses when implementing the opt-out testing approach in PMTCT testing seem to be highly required to avoid venturing into a dangerous field e.g. women choosing not to disclose their status or violence upon disclosure which at times, lead to breakage of marriages. It should in this context be noted that some studies have shown that disclosure could also lead to supportive reactions as indicated in a review [130].

Alternatives ways to provide HIV testing that are more sensitive to the major challenges reported from the existing strategies are today sought for, and are underway in some countries. One of approaches that appear as potentially promising is the 'home based VCT approach' that is provided by health providers who visit and perform VCT in the people's homes. This approach has been found to be: more acceptable, encourage disclosure and reduce gender inequities in testing [109, 131, 132]. While this strategy may be effective, more studies have been called to explore the challenges and the cost effectiveness of this testing strategy [133].

Use of VCT services

Equal access to HIV testing is one of the important objectives of many national HIV programs [53]. The Andersen behavioural model was utilised in the analysis of paper I. This model has mainly been used to measure equitable access to health care in order to guide policies that in various ways promote equitable access to health care services. Inequity in use of HIV testing occur in this model when predisposing and or enabling factors determine who uses the health care services [89]. HIV stigma, one of the defined predisposing factors in this study, was associated with use of VCT for HIV testing (paper I). It was shown that those scoring lowest on stigma in all the three districts were significantly more likely to use VCT for HIV testing than those with highest scores. This finding is consistent with previous studies in the region [31, 65, 134, 135] and have added to the knowledge that it is vital to search for new and creative ways to face the challenges of HIV related stigma

Participants with higher educational attainment levels were more likely to use VCT compared to those with lower education levels. This finding concurs with several other studies including country surveys [32, 34, 65, 111, 136, 137]. As a key indicator of socio economic position, education attainment is likely to enable individuals to seek services and act on preventive messages. In this regards, a particularly notable finding in our study was the negative association between education attainment and stigma. The association was consistently observed in the three sampled districts. Similarly a study in Ghana indicated that individuals with limited education are more likely to stigmatise people living with HIV [138].

Applying the Andersen model to measure equity on use of HIV testing services faces a particular limitation since measurement of 'the need component' is less clearly defined as compared to help- seeking for acute illnesses. We applied a combination of variables to

compensate for this, each assumed to be potential indicators of need based on previous studies. The plausibility represents a major challenge in this regard. This is indicated by the fact that the strength of association with other factors was not substantially affected after adjustment of the need factors. We applied the original Anderson model [89]. However the Andersen model, has been extended during the years and future studies should include measures trying to capture dimensions of responsiveness and trust in the local health services as indicated in paper III and others [58, 89].

The responsiveness concept in assessing HIV testing services

Evaluation of health systems in low income countries has been described as weak, hence creative ways of assessing the non-health aspects that are of vital importance to utilization of the health services (in this case HIV testing) are vital. Responsiveness has been proposed as vital in assessing the non-health related aspect of health systems [22, 23, 59]. As shown in paper III, the elements outlined by WHO to measure responsiveness were rated as very important in assessing VCT by the informants of our study. In particular emphasis was placed on the elements that capture the respect for the person's domain such as confidentiality, dignity and autonomy that were rated as important by a very high number of the study participants. The qualitative findings likewise revealed important aspects of these elements, but provided more comprehensive information related to the various elements in terms of information that may be important in planning for further responsiveness studies. The aspects that emerged as vital when exploring responsiveness in the context of HIV testing are discussed in the next sections.

Confidentiality

According to WHO's responsiveness concept confidentiality is observed when information provided by the patient or about the patient and medical records are kept confidential, and when privacy during consultations is observed [22, 23]. The findings in paper III indicate that the tool captured vital experiences with the health system inside the service room (VCT facility), but was leaving out contextual issues that may influence confidentiality. For example the fear of meeting a known health provider who could breach confidentiality was expressed. This finding has been found to be one of the key factors hindering the use of VCT also in other studies [32, 33, 63]. It must be recalled that HIV infection since the early years of its discovery to date is linked to stigma [16, 65, 139, 140]. Thus the mere act of being seen at a VCT facility would readily be associated with HIV infection and linked to characteristics

of immorality and promiscuity. In this regard confidentiality emerges as an issue of immense concern at HIV testing facilities, not only inside the VCT room but also outside it.

Efforts to normalise HIV testing have been introduced through the provider initiated opt-out strategy. Some have argued that normalising the test to the populations, could be a way of normalising HIV with potentials to decrease stigma [11, 141, 142]. Indeed the opt-out strategy in the long run may imply a positive development in terms of increasing exposures to HIV testing, and thus in making the HIV infection less special. Albeit potentially promising in a confidentiality context, the manner in which the opt-out approach currently seems to be implemented, also beyond our study site, emerges as highly challenging from an ethical and human rights perspective [12, 47, 49]. Moreover paper II points to the concerns that have emerged due to the lack of pre and post- test counselling for those testing negative. This lack undermines autonomy one of the responsiveness elements which compromises confidence in the health providers and the services in general.

Autonomy

Autonomy as a WHO's responsiveness value involves the experience of decision making based on proper information, consultation, preferences and consent. Furthermore, the autonomy concept retains that respect is observed of rights of clients to refuse treatment, testing or the like [22, 23]. Our study findings in paper III revealed that autonomy emerged as a highly valued element by the majority of the respondents. Due to the pre-test counselling that is embedded in the testing procedures at the VCT settings, autonomy was reported as highly observed. The qualitative part of this study (paper III) enabled the revelation also of other important and seemingly crucial aspects related to 'autonomy' that were not captured by the WHO tool. This related to aspects as fundamental as providing information in a language that is understandable to the clients (paper III). From a responsiveness perspective providing information is obviously vital with regards to the communication of information to the patient and involvement of the patient in decision making. The approach is dependent on involvement of the patients in a dialogue about his/her own health and which places the patient in control.

Assessingt responsiveness in specific health services.

Previous studies on responsiveness target the entire health system, commonly at a country level or even multi-country studies for comparison. As our study and a few other studies have

pointed out [76, 143], the responsiveness component can also be useful in the assessment of specific services within the health system. Such approaches may be particularly fruitful in generating culturally sensitive information; considering aspects that are specific to the populations where the study is conducted. This allows us to make attempt at capturing the meanings of the elements as experienced by individual in particular contexts. I will argue that exploring responsiveness with regards to the implementation of the PITC model is particularly important. In light of the compromises made on counselling as found in our study (paper II), it is likely that the PITC model will greatly result in experiences of poor responsiveness due to the weak emphasis made on counselling and consent that is so crucial for autonomy. However, this topic requires further studies to generate in-depth knowledge.

6 Conclusions

The present study revealed that the factors associated with VCT varied to relatively little degree in the three study districts in Kenya, Tanzania and Zambia. The main variations related to HIV testing were relatively consistent across the three districts. These variations were primarily related to education attainment and to HIV stigma. This study thus confirms the finding that education is imperative in the scaling up of HIV testing efforts, and is moreover crucial in the fight against HIV associated stigma.

The differences revealed in HIV testing exposures across the three districts can be related to different timing of the scaling up of the provider initiated opt-out strategy in HIV testing in the districts. The PITC opt-out model also in our study emerged as beneficial with regards to improved access to HIV testing. While this in itself is important, the challenging equity dimensions of this testing service is important to note due to its roll out primarily in the mother to child transmission of HIV programmes. The PITC model of testing was by the informants in the present study experienced as placing limited effort in obtaining informed consent; pre-test counselling was not carried out, and even the recommended pre-test information was rarely practiced in the study area. Post-test counselling was moreover lacking for individuals testing HIV negative implying that vital preventive opportunities were lost. At the time of the data collection for the present study, the PITC model of testing implemented in PMTCT was experienced to put an unreasonable burden on women who were given the task of disclosing their status to their husbands and to recruit their spouses for HIV testing at a most vulnerable time in their life; while expecting a child.

The documented difficulty of recruiting people for VCT has been strongly associated with poor functioning health systems, not the least voids in the human resource situation at the health facilities. A responsive health service environment can in this context be deemed vital. WHO's 'responsiveness tool' is to measure experienced levels of responsiveness of the health system. This dimension was perceived as highly relevant in assessing HIV testing services. Assessment of WHO's tool in the present study revealed that adjustments of the tool seem to be demanded in order to facilitate an exploration of elements that are relevant to the particular study settings. Our study demonstrates that assessment and recognition of locally specific experiences and views are particularly important when the aim is to grasp how the health system can become more responsive in the future. The findings related to the importance of considering factors of concern outside the VCT room has relevance beyond this particular study setting, and is central in most settings where HIV stigma is rampant and a hindrance to HIV testing.

7 Implications for Policy and Research

7.1 Policy considerations

The study findings point to the need of urgently reconsidering the manner in which the PMTCT based PITC opt-out is implemented. This is vital in order to protect client's rights by enhancing their autonomy, and thus to empower the users to make their own decision regarding HIV testing. Further to allow the users to benefit from the process through access to HIV prevention, efforts to implement couple counselling and testing to avoid the many challenges linked to the opt-out approach implemented through PMTCT projects are needed. In some settings the promotion of home-based VCT have shown promising signs not the least in terms of equitable access to testing and in terms of reducing the stigmatizing contexts surrounding the VCT facilities. The home based testing services target the entire family. Further assessment related to the involvement of lay counsellors as attempted in Botswana could fruitfully be made in attempts to ease the work load of the health workers who at times cannot cope with the workload associated with resource demanding HIV testing services. Continued promotion of formal education is needed for a large number of reasons. In this context as education has been demonstrated to be associated with higher VCT use and with less HIV related stigma. Education plays a key part in a nation's health building efforts as higher educated people are more likely to make autonomous decisions and respond to health

promotion efforts. The responsiveness dimensions is vital in an HIV context, and WHO's tool that is to assess the responsiveness of health systems urgently needs to be adapted to local settings and used more extensively in efforts to evaluate local health systems.

7.2 Future research recommendations

The PITC opt-out strategy was widely scaled up without previous testing through community randomised trials. The major findings in the present study indicate the importance of proper validations of such programs or strategies before wider scaling up, not the least with regards to responsiveness perspectives. This study has assessed perceptions and experiences with the PITC model from community perspectives, but further research is needed to explore potential benefits or challenges as experienced in other settings and also to capture the views of health care workers. Health systems' responsiveness would be vital to explore the PITC model in order to further assess the potential challenges or strengths that are experienced in the implementation of this new HIV testing model.

Educational attainment was seen as negatively associated with stigma. Future studies need to be conducted to further assess this association by employing a more comprehensive stigma measurement instrument to capture different dimensions of stigma. Furthermore, stigma measurement tools need to be validated in different socioeconomic and cultural contexts to better understand stigma dynamics and to better guide stigma preventive efforts.

Accountability for reasonableness is an ethically-based framework for fair decision-making being promoted by the REACT project. This framework draws attention to the importance of equity, quality and trust in health systems. In the light of our findings, AFR conditions emerge as vital in assessing decision-making processes. The publicity element was e.g. lacking during the introduction of the PITC model of testing. Informants were not informed about the change in testing policies, and little was done to prepare them for the test. This creates an environment that paves the way for distrust in the health system. A major lesson learnt is that more research is needed that apply rights- and ethically based frameworks, such as AFR, into decision making in order to assess the acceptability of programs and interventions before considerations of scaling up are made.

8 References

1. Merson MH, Malley JO, Serwadda D, Apisuk C: **The history and challenge of HIV prevention** *The Lancet* 2007, **372**(9637): 475-488

2. UNAIDS: **UNAIDS Report on the Global AIDS Epidemic: 2010** Geneva, Switzerland; 2010.

3. KAISER FAMILY FOUNDATION: **The global HIV/AIDS epidemic fact sheet**. Washington DC; 2010.

4. CDC MMWR: **Pneumocystis Pneumonia --- Los Angeles**. 1981, **30**(21):1-3.

5. Curran JW: **The epidemiology of AIDS: current status and future prospects**. *Science* 1985, **229**(4720):1352.

6. **The Global HIV/AIDS Timeline** [http://www.kff.org/hivaids/timeline/hivtimeline.cfm]

7. CDC MMWR: **Opportunistic infections and Kaposi's sarcoma among Haitians in the United States**. CDC; 1982:353-354.

8. Clumeck N., Mascart-Lemone F., De Maubeuge J, Brenez D, Marcelis. L.: **Aquired Immune Deficiency Syndrome in black African**. *The Lancet* 1983, **1**:642.

9. UNAIDS: **Voluntary Counselling and Testing (VCT)**. Geneva: UNAIDS; 2000.

10. Bayer R: **Public health policy and the AIDS epidemic-An end to HIV exceptionalism?** *New England journal of Medicine* 1991, **324**(21):1500-1504.

11. Bayer R, Edington: **HIV testing, Human Rights, and Global AIDS policy: Exceptionalism and its Discontents**. *Journal of Health Politics and law* 2009, **34**(3):301-323.

12. Obermeyer MO, Osborn M: **The utilization of testing and counselling for HIV: A review of the social and behavioural evidence**. *Am J Public Health* 2007, **97**(10):1762-1774.

13. CDC MMWR: **Current Trends Additional Recommendations to Reduce Sexual and Drug Abuse-Related Transmission of Human T-Lymphotropic Virus Type III/ Lymphadenopathy-Associated Virus** *MMWR* 1986, **35**(10):152-155.

14. Fehrs LJ: **Trial of anonymous versus confidential Human Immunodeficiency Virus testing**. *Lancet* 1988, **332**(8607):379.

15. Preston JB: **Confidentiality in the HIV antibody testing process**. *Journal of Nurse-Midwifery* 1989, **32**(5):283-286.

16. Blendon R, Donelan K: **Discrimination against people with AIDS: the public perspective**. *The New England journal of medicine* 1988, **319**(15):1022-1025.

17. Herek GM, Capitanio J: **Public Reactions to AIDS in the United States: A Second Decade of Stigma**. *American Journal of Public Health* 1993, **83**(4).

18. Centers for Disease Control (CDC): **Current Trends Additional Recommendations to Reduce Sexual and Drug Abuse-Related Transmission of Human T-Lymphotropic Virus Type III/ Lymphadenopathy-Associated Virus** *Morbidity and Mortality weekly Report (MMWR)* 1986, **35**:152-155.

19. Mccusker J, Stoddard AM, Mayer KH, Zapka J, Morrison C, Saltzman S: **Effects of HIV Antibody Test Knowledge on Subsequent Sexual Behaviours in a Cohort of Homosexually Active Men**. *American Journal of Public Health* 1988, **78**(4):462-466.

20. Soskilne CL: **Importance of counselling in HIV antibody testing**. *CMAJ* 1988, **139**:709-710.

21. UNAIDS technical update: **Counselling and HIV /AIDS**. 1997.

22. Darby C, Valentine N, Murray CL, De Silva A: **World Health Organization: Strategy on measuring responsiveness**. In *GPE Discussion Paper series. Volume 23*. WHO; 2000.

23. Murray CJL, Evans DB: *Health systems performance assessment: Debates, methods and empiricism*. Geneva: World Health Organization; 2003.

24. Joint publication of IPPF South Asia Regional Office and UNFPA: **Integrating HIV voluntary counselling and testing services into reproductive health settings: Stepwise guidelines for programme planners, managers and service providers** 2004.

25. Coates TJ: **Efficacy of voluntary HIV-1 counseling and testing in individuals and couples in Kenya, Tanzania and Trinidad: a randomised trial**. *Lancet* 2000, **356**:103-112.

26. WHO, UNAIDS: **Guidance on provider-initiated HIV testing and counselling in health facilities**. Geneva, Switzerland; 2007.

27. Creek TL, Alwano MG, Molosiwa RR, Roels TH KT, Mwasalla V, Lloyd ES, Mokomane M, Hastings PA, Taylor AW, PH K: **Botswana's Tebelopele Voluntary HIV Counseling and Testing Network: Use and Client Risk Factors for HIV Infection, 2000-2004**. *J Acquir Immune Defic Syndr* 2006, **43**(2):210-218.

28. Denison JA, O'Reilly KR, Schmid GP, Kennedy CE, Sweat MD: **HIV voluntary counseling and testing and behavioral risk reduction in developing countries: A meta analysis, 1990-2005**. *AIDS Behav* 2007(12):363-373.

29. Mola OD MM, Asghar RJ, Gimbel-Sherr KH, Gimbel-Sherr S, Micek MA, Gloyd SS: **Condom use after voluntary counselling and testing in central Mozambique.** *Trop Med Int Health* 2006 **11**(2):176-181.

30. Cremin I, Nyamukapa C, Sherr L, Hallett TB, Chawira G, Cauchemez S, Lopman B, Garnett GP, Gregson S: **Patterns of Self-reported Behaviour Change Associated with Receiving Voluntary Counselling and Testing in a Longitudinal Study from Manicaland, Zimbabwe.** *AIDS Behav* 2009, **10**.

31. Babalola S: **Readiness for HIV testing among young people in northern Nigeria: The roles of social norm and perceived stigma**. *AIDS Behav* 2006, **11**(10):759-769.

32. Fylkesnes K, Haworth A, Rosenvard C, Kwapa P: **HIV counselling and testing: Overemphasizing high acceptance rates a threat to confidentiality and the right not to know**. *AIDS* 1999, **13**(17):2469-2474.

33. Irungu T, Varkey P, Cha S, Patterson J: **HIV voluntary counselling and testing in Nakuru, Kenya: Findings from a community survey**. *British HIV Association HIV Medicine* 2008, **9**(2):111-117.

34. Sherr L, Lopman B, Kakowa M, Dube S, Chawira G, Nyamukapa C, Oberzaucher N, Cremin I, Gregson S: **Voluntary counselling and testing: uptake, impact on sexual behaviour andHIV incidence in a rural Zimbabwean cohort.** *AIDS* 2007, **21**:851-860.

35. Connor EM, Sperling RS, Gelber R, Kiselev P, Scott G, O'Sullivan MJ, VanDyke R, Bey M, Shearer W, Jacobson RL: **Reduction of maternal-infant transmission of human immunodeficiency virus type 1 with zidovudine treatment. Pediatric AIDS Clinical Trials Group Protocol 076 Study Group**. *The New England journal of medicine* 1994, **331**(18):1173-1180.

36. Koo DJ, Begier EM, Henn MH, Sepkowitz KA, Kellerman SE: **HIV counselling and testing: Less targeting more testing**. *American Journal of Health Behavior* 2006, **96**(6):962-964.

37. WHO: **The Right to Know: New Approaches to HIV Testing and Counselling**. 2003.

38. Kim JY: **Scaling up access to care in resource constrained settings: What is needed?** In *Plenary address at the fifteenth International AIDS conference, Bangkok* 2004.

39. De Cock KM, Mbori-Ngacha D, E. M: **Shadow on the continent: public health and HIV/AIDS in Africa in the 21st century**. *Lancet* 2002, **360**(9326):67-72.

40. Rennie S, Behets F: **Desperately seeking targets: the ethics of routine HIV testing in low-income countries**. *Bulletin of the World Health Organization* 2006, **84**(1):52-57.

41. Creek TL, Ntumy R, Seipone K, Smith M, Mogodi M, Smit M, Legwaila K, Molokwane I, Tebele G, Mazhani L *et al*: **Successful introduction of routine opt-out HIV testing in antenatal care in Botswana**. *J Acquir Immune Defic Syndr* 2007, **45**(1):102-107.

42. Moses A, Zimba C, Kamanga E, Nkhoma J, Maida A, Martinson F, Mofolo I, Joaki G, Muita J, Spensley A *et al*: **Prevention of mother-to-child transmission: program changes and the effect on uptake of the HIVNET 012 regimen in Malawi**. *Aids* 2008, **22**(1):83-87.

43. Nakanjako D, Kamya M, Kyabayinze D, Mayanja-Kizza H, Freers J, Whalen C, E. K: **Acceptance of routine testing for HIV among adult patients at the medical emergency unit at a national referral hospital in Kampala, Uganda.** *AIDS Behav* 2007, **11**:753-758.

44. Perez F, Zvandaziva C, Engelsmann B, Dabis F: **Acceptability of routine HIV testing ("opt-out") in antenatal services in two rural districts of Zimbabwe**. *J Acquir Immune Defic Syndr* 2006, **41**(4):514-520.

45. Steen TW, Seipone K, Anderson MG, Kejelepula M, Keapoletswe K, Moffat HJ: **Two and a Half years of routine HIV testing in Botswana**. *AIDS* 2007, **44**(4):484-488.

46. Weiser SD, Heisler M, Leiter K, Percy-de Korte F, Tlou S, DeMonner S, Phaladze N, Bangsberg DR, Iacopino V: **Routine HIV testing in Botswana: a population-based study on attitudes, practices, and human rights concerns**. *PLoS Med* 2006, **3**(7):1013-1022.

47. Maman S, King E: **Changes in HIV testing policies and the Implications for Women**. *Journal of Midwifery & Women's Health* 2008, **53**(3):195-201.

48. Gruskin S, Ahmed S, Ferguson L: **Provider initiated HIV testing and counselling in health facilities- what does this mean for the health and human rights of pregnant women?** *Developing World Bioethics* 2007, **8**(1):23-32.

49. Yeatman SE: **Ethical and public health considerations in HIV counselling and testing: Policy implications**. *Studies in Family Planning* 2007, **38**(4):271-278.

50. Kenya National Bureau of Statistics (KNBS)and ICF Macro: **Kenya Demographic and Health Survey 2008-09.** Calverton, Maryland: KNBS and ICF Macro. ; 2010.

51. Central Statistical Office (CSO), Ministry of Health (MOH), Tropical Diseases Research Centre (TDRC), University of Zambia, Macro International Inc: **Zambia Demographic and Health Survey 2007**. Calverton, Maryland, USA: CSO and Macro International Inc.; 2009.

52. Tanzania Commission for AIDS (TACAIDS), Zanzibar AIDS Commission (ZAC), National Bureau of Statistics (NBS), Office of the Chief Government Statistician (OCGS), Macro International Inc: **Tanzania HIV/AIDS and Malaria Indicator Survey 2007-08**. Dar es Salaam,: TACAIDS, ZAC, NBS, OCGS, and Macro International Inc; 2008. .

53. National AIDS and STI Control Programme, Ministry of Public health and sanitation Kenya: **National guidelines for HIV testing and counselling in Kenya**. Nairobi 2008.

54. Ministry of Health Zambia, National AIDS council, UNGASS: **Zambia Country Report: Monitoring the Declaration of Commitment on HIV and AIDS and the Universal Access**. 2010.

55. WHO: **Health systems: improving performance**. Geneva: World Health Organization; 2000.

56. World Health Organization maximising positive synergies collaborative group: **An assessment of interactions between global health iniitatives and country health systems**. *Lancet* 2009, **373**:2137-2169.

57. De Silva A: **A framework for measuring responsiveness**. In *GPE Discussion Paper series No 32*. Geneva: WHO; 2000.

58. De Silva A, Valentine N: **Measuring responsiveness: Results of a key informant survey in 35 countries.** In *GPE Discussion Paper series No 21*. Geneva: WHO; 2000.

59. Murray CJ, Frenk J: **A framework for assessing the performance of health systems**. *Bull World Health Organ* 2000, **78**(6):717-731.

60. Whitehead M: **The concepts and principles of equity and health**. *International journal of health services* 1995, **22**(3):429.

61. Chang WC: **The meaning and goals of equity in health**. *J Epidemiol Community Health* 2002, **56**(7):488-491.

62. Braveman P, Gruskin S: **Defining equity in health**. *J Epidemiol Community Health* 2003, **57**:254–258.

63. Fylkesnes K, Siziya S: **A randomized trial on acceptability of voluntary HIV counselling and testing**. *Tropical Medicine and International Health* 2004, **9**(5):566-572.

64. Pool R, Nyanzi SI, Whitworth JAG: **Attitudes to voluntary counselling and testing for HIV among pregnant women in rural south-west Uganda**. *AIDS CARE* 2001, **13**(5):605-615.

65. Kalichman SC, L.C. S: **HIV testing attitudes, AIDS stigma, and voluntary HIV counselling and testing in a black township in Cape Town, South Africa**. *Sex Transm Infect* 2003(79):442–447.

66. Wringe A, Isingo R, Urassa M, Maiseli G, Manyalla R, Changalucha J, Mngara J, S. K, Zaba B: **Uptake of HIV voluntary counselling and testing services in rural Tanzania: implications for effective HIV prevention and equitable access to treatment**. *Tropical Medicine and International Health* 2008, **13**(3):319-327.

67. Ministry of Health, National Aids and STI Control Programme: **AIDS in Kenya trends, Interventions and Impact**. 7th edition. Nairobi: Ministry of Health, National AIDS and STI Control Programme; 2005:104.

68. April MM, Walensky RP, Chang Y, Pitt J, Freedberg KA, Losina E, Paltiel DA, Wood R: **HIV Testing Rates and Outcome in a South African Community, 2001-2006: Implications for expanded screening policies**. *Journal of Acquired Immne Deficiency Syndromes* 2009, **51**(3):310-316.

69. Grovesa AK, Mamana S, Msomib S, Makhanyab N, Moodleyb D: **The complexity of consent: women's experiences testing for HIV at an antenatal clinic in Durban, South Africa**. *AIDS Care* 2010, **22**(5):538-544.

70. Rennie S, Mupenda B: **Ethics of mandatory premarital HIV testing in Africa: The case of Goma, Democratic Republic of Congo**. *Developing World Bioethics* 2008, **8**(2):126-137.

71. Csete J, Schleifer R, Cohen J: **"Opt-out" testing for HIV in Africa: a caution**. *Lancet* 2004, **363**(9407):493-494.

72. Becker J, Tsague L, Sahabo R, Twyman P: **Provider initiated testing and counselling (PITC) for HIV in resource-limited clinical settings: Important questions unanswered**. *PanAfrican hournal* 2009.

73. Valentine N, Silva A, Murray CJL: **Estimating responsiveness level and distribution for 191 countries: Methods and results**. In *GPE Discussion paper Series: No 22*. Geneva: World Health Organization; 2000.

74. Ugurluoglu O, Celik Y: **How Responsive Turkish health care system is to its citizens: The views of Hospital managers**. *J Med Syst* 2006, **30**(6):421-428.

75. Hsu CC, Chen L, Hu YW, Yip W, Shu CC: **The dimensions of responsiveness of a health system: A Taiwanese perspective**. *BMC Public Health* 2006, **6**(72).

76. Bramesfeld A, Wedegartner F, Elgeti H, Bisson S: **How does mental health care perform in respect to service users' expectations? Evaluating inpatients and outpatients care in Germany with the WHO responsiveness concept**. *BMC Health Service Research* 2007, **7**(99):1-12.

77. Martin D, Singer P: **A strategy to improve priority setting in health care institutions** *Health Care Analysis* 2003, **11**(1):59-68.

78. Maluka S, Hurtig A, Sebastián M, Shayo EH, Byskov J, Kamuzora P: **Decentralization and health care prioritization process in Tanzania: from national rhetoric to local reality**. *Int J Health Plann Mgmt* 2010, **10**.

79. Byskov J, Bloch P, A. B, Hurtig AK, Fylkesnes K, Kamuzora P, Kombe Y, Kvåle G, Marchal B, Martin DM *et al*: **Accountable priority setting for trust in health systems – the need for research into a new approach for strengthening sustainable health action in developing countries** *BMC Health Research Policy and Systems* 2009, **7**(23):24 October 2009.

80. Ministry of Finance and planning Kenya: **Malindi district development plan 2002-2008**. Nairobi: Republic of Kenya; 2002-2008.

81. Michelo C, Sandøy I, Dzekedzeke K, Siziya S, Fylkesnes K: **Steep HIV prevalence declines among young people in selected Zambian communities (1995-2003)**. *BMC Public Health* 2006, **6**(279):

82. Fylkesnes K, Ndhlovu Z, Kasumba K, Musonda RM, M. S: **Studying dynamics of the HIV epidemic: population-based data compared with sentinel surveillance in Zambia** *AIDS* 1998, **12**(1227-1234.)

83. Ministry of Health Kenya: **Malindi district annual health report 2004**. 2005.

84. National Coordinating Agency for Population and Development: **Malindi District Strategic Plan 2005 - 2010 for Implementation of the National Population Policy for Sustainable Development**. Ministry of Planning and National Development; 2005.

85. Central census office National bureau of statistics: *The united republic of Tanzania: 2002 population and housing census. Volume 2*. Dar es Salaam; 2003.

86. Mbarali district council: **District Investment Profile 2010**. Mbarali; 2010.

87. Johnson RB, Onwuegbuzie AJ: **Mixed Methods Research: A research paradigm whose time has come**. *Educational Researcher* 2004, **33**(7):14-26.

88. Creswell JW, Plano clark VJ, Hanson WE: *A handbook of mixed methods in social and behavioral research*. California: Sage publications Inc; 2003.

89. Andersen RM: **Revisiting the behavioural model and access to health cae: Does it matter?** . *Journal of health and social behavior* 1995, **36**:1-10.

90. Gelberg L, Andersen RN, Leake BD: **The behavioral model for vulnerable populations: Application to medical care use and outcomes for homeless people**. *HSR: health services reserch* 2000, **34**(6):1273-1302.

91. Malterud K: **Qualitative reserch: standards, challenges and guidelines**. *The Lancet* 2001, **358**:483-488.

92. Pope C, Mays N: *Qualitative research in health care*. Third edition. Oxford, UK: Blackwell Ltd; 2006.

93. Dawson S, Manderson L: *A manual for the use of focus groups*. Boston, USA: International Nutrition foundation for developing countries (INFDC); 1993.

94. Polit DE, P.Hungler B: *Nursing Research: Principles and Methods* Sixth edition. New York: Lippincott; 1999.

95. Lacey A, Luff D: *Trent focus for research and development in primary Health Care: An introduction to qualitative data analysis*: Trent Focus Group; 2001.

96. Pope C, Ziebland S, Mays N: **Qualitative research in health care: Analysing qualitative data**. *BMJ* 2000, **320**:114-116.

97. Buetow S: **Thematic analysis and its reconceptualization as 'saliency analysis'**. *Journal of Health Service and Policy* 2010, **15**(2):123-125.

98. Braun V, Clarke V: **Using thematic analysis in psychology**. *Qualitative reserch in psychology* 2006, **3**:77-101.

99. Cathain A.O, Murphy E, Nicholl J: **Integration and publications as indicators of "Yield" from Mixed methods studies**. *Journal of Mixed methods research* 2007, **1**(2):147-163.

100. Teddlie C, Tashakkori A: *Major issues and controversies in the use of mixed methods in the social and behavioral sciences*. California: Sage publications; 2003.

101. Grimes DA, Schulz KF: **Bias and causal associations in observational research**. *The Lancet* 2002, **359**:248-252.

102. Rothman KJ, Greenland S: *Modern Epidemiology*. Second edition: Lippincott-Raven; 1998.

103. Sandøy I, Michelo C, Siziya S, Fylkesnes K: **Associations between sexual behaviour change in young people and decline in HIV prevalence in Zambia.** *BMC Public Health 2007 Apr 23;7:60* 2007, **7**(60).

104. Barrett JR: **The researcher as instrument: learning to conduct qualitative research through analyzing and interpreting a choral rehearsal**. *Music Education Research* 2007, **9**(3):417-433.

105. Mays N, Pope C: **Qualitative researcg in health care assessing quality in qualitative reserch**. *BMJ* 2000, **320**.

106. Larsson EC, Thorson A, Pariyo G, Conrad P, Arinaitwe M, Kemigisa M, Eriksen J, Tomson G, Ekström AM: **Opt-out HIV testing during antenatal care: experiences of pregnant women in rural Uganda**. *Health Policy Plan* 2011.

107. Awiti Ujiji O, Rubenson B, Ilako F, Marrone G, Wamalwa D, Wangalwa G, Ekstrom AM: **Is 'Opt-Out HIV Testing' a Real Option among Pregnant Women in Rural Districts in Kenya?** *BMC Public Health* 2011, **11**(151).

108. Beteganya MH, Abdulwadud OA, Kiene SM: **Home based HIV voluntary counselling and testing in developing countries**. *Cochrane database of systematic Reviews* 2007(4).

109. Angoti N, Bula A, Gaydosh L, Kimchi EZ, Thornton RL, Yeatman SE: **Increasing the acceptability of HIV counselling and testing with three C's: Convinience, confidentiality and credibility.** *Social Science and Medicine* 2009, **68**(12):2263-2270.

110. Ministry of Medical Services, Ministry of Public Health and Sanitation: **Kenya AIDS Indicatory Survey (KAIS)**. 2009.

111. Tanzania Commission for AIDS, Zanzibar AIDS Commission , National Bureau of Statistics, Macro International Inc: **Tanzania HIV/AIDS and Malaria Indicator Survey 2007-08 In Preliminary Report**. 2008:1-58.

112. Taegtmeyer M, Kilonzo N, Mung'ala L, Morgan G, Theobald S: **Using gender analysis to build voluntary counselling and testing responses in Kenya**. *The transactions of the Royal society of Tropical Medicine and Hygiene* 2005, **100**:305-311.

113. De Graft- Johnson J, Paz-Soldan V, Kasote A, Tsui A: **HIV voluntary counseling and testing service preferences in rural Malawi population**. *AIDS and Behavior* 2005, **9**(4):475-483.

114. WHO: **Primary health care now more than ever**. Geneva: World Health Organization; 2008.

115. Manavi K, Welsby P.D: **HIV testing should no longer be accorded any special status**. *BMJ* 2005, **330**:492-493.

116. Travis p, Bennett S, Haines A, Pang T, Bhutta Z, Hyder AA, Pielemeier NR, Mills A, Evans T: **Overcoming health-systems constraints to achieve the Millennium Development Goals**. *Lancet* 2004, **364:** :900–906.

117. The Joint learning initiative Africa working group: **The Health Workforce in Africa Challenges and Prospects**. 2006.

118. Junga J, Blystad A: **'The divorce program': gendered experiences of HIV positive mothers enrolled in PMTCT programs- the case of rural Malawi**. *International breast feeding journal* 2010, **5**(14).

119. Higgins DL: **Evidence for the effects of HIV antibody counseling and testing on risk behaviors**. *JAMA (Chicago, Ill)* 1991, **266**(17):2419.

120. Allen S, Serufilira A, Bogaerts J, Van de Perre P, Nsengumuremyi F, Lindan C, Carael M, Wolf W, Coates T, Hulley: **Confidential HIV testing and condom promotion in Africa. Impact on HIV and gonorrhea rates**. *The journal of American medical association* 1992, **268**(23).

121. Allen S, Tice j, Van de Perre P, Serufilira A, Hudes E, Nsengumuremyi F, Bogaerts J, Lindan C, Hulley S: **Effect of serotesting with counselling on condom use and seroconversion among HIV discordant couples in Africa**. *BMJ* 1992, **304**.

122. **Global HIV prevention: The access and funding gap** [globalhivprevention.org/pdfs/pwg_access_factsheet_6_07.pdf]

123. United Nations Development Program: **Thailand's response to HIV/AIDS: progress and challenges**. New York: United Nations Development program; 2004

.

124. Chandisarewa W, Stranix-Chibanda L, Chirapa E, Miller A, Simoyi M, Mahomva A, Maldonado Y, Shetty AK: **Routine offer of antenatal HIV testing ("opt-out"**

approach) to prevent mother-to-child transmission of HIV in urban Zimbabwe. *Bull World Health Organ* 2007, **85**(11):843-850.

125. Blystad A, van Esterik P, de Paoli MM, Sellen DW, Leshabari SC, Moland KM: **Reflections on global policy documents and the WHO's infant feeding guidelines: lessons learnt.** *International breast feeding journal* 2010, **5**(18).

126. Leshabari S, Koniz-Booher P, Åstrøm AN, De Paoli MM, Moland KM: **Translating global recommendations on HIV and infant feeding to the local context: the development of culturally sensitive counselling tools in the Kilimanjaro Region, Tanzania**. *BMC Implementation science* 2006, **1**(22).

127. Moland KMI, Esterik PV, Sellen D, De Paoli MM, Leshabari SC, Blystad A: **Ways ahead: protecting, promoting and supporting breastfeeding in the context of HIV**. *International breastfeeding journal* 2010, **5**(1):19.

128. Moodleya D, Esterhuizenb TM, Pathera T, Chettya V, Ngaleka L: **High HIV incidence during pregnancy: compelling reason for repeat HIV testing**. *AIDS* 2009, **23**:1255–1259.

129. The Center for Reproductive Rights, Federation of Women Lawyers—Kenya: **At Risk: Rights Violations of HIV-Positive Women in Kenyan Health Facilities**. United States; 2008

130. Medley A, Garcia-Moreno G, McGill S, Maman S: **Rates, barriers and outcomes of HIV serostatus disclosure among women in developing countries: implications for prevention of mother-to-child transmission programmes**. *Bull World Health Organ* 2004, **82**(4):299-304.

131. Mutale W, Michelo C, Jurgensen M, Fylkesnes K: **Home-Based voluntary HIV counselling and testing found highly acceptable and to reduce inequalitites**. *BMC public Health* 2010, **10**.

132. Negin J, Wariero J, Mutuo P, Jan S, Pronyk P: **Feasibility, acceptability and cost of home-based HIV testing in rural Kenya**. *Tropical Medicine and International Health* 2009, **14**(8):1-7.

133. Bateganya M, Abdulwadud OA, SM. K: **Home-based HIV voluntary counselling and testing (VCT) for improving uptake of HIV testing.** *Cochrane Database Syst Rev 2;(7):CD006493* 2010(7 Art. No.: CD006493. DOI:10.1002/14651858.CD006493.pub4.).

134. Pettifor A, MacPhail C, Suchindran S, Delany-Moretlwe S: **Factors Associated with HIV Testing Among Public Sector Clinic Attendees in Johannesburg, South Africa**. *AIDS Behav* 2010, **14**:913–921.

135. Koku EF: **Desire for, and Uptake of HIV Tests by Ghanaian Women: The Relevance of Community Level Stigma.** *J Community Health* 2010.

136. Hutchinson PL, Mahlalela X: **Utilization of Voluntary counselling and testing services in the Eastern Cape, south Africa**. *AIDS Care* 2006, **18**(5):446-455.

137. MacPhaila C, Pettifora A, Moyo W, Rees H: **Factors associated with HIV testing among sexually active South African youth aged 15-24 years**. *AIDS care* 2009, **21**(4):456-467.

138. Ulasi CI, Preko PO, Baidoo JA, Bayard B, Ehiri JE, Jolly CM J, PE. J: **HIV/AIDS-related stigma in Kumasi, Ghana**. *Health place* 2009, **15**(1):255-262.

139. Ogden J, Nyblade L: **Common at Its Core: HIV-Related Stigma Across Contexts**. International Center for Research on Women (ICRW). 2005:15-30.

140. Skinner D, Mfecane S: **Stigma, discrimination and the implications for people living with HIV/AIDS in South Africa**. *Journal of Social Aspects of HIV/AIDS* 2004, **1**(3):157-164.

141. De Cock KM: **From exceptionalism to normalisation: a reappraisal of attitudes and practice around HIV testing**. *BMJ British medical journal* 1998, **316**(7127):290.

142. De Cock KM, Bunnell R, Mermin J: **Unfinished business--expanding HIV testing in developing countries**. *The New England journal of medicine* 2006, **354**(5):440-442.

143. Bramesfeld A, Klippel U, Seidel G, Schwartz FW, Dierks M: **How do patients expect the mental health service system to act? Testing the WHO responsiveness concept for its appropriateness in mental health care.** *Social Science & Medicine* 2007, **65**(5):880-889.

9 Original papers Paper I – III

Status of the papers

Paper I: Submitted
Paper II: Published
Paper III: Published

I

Inequalities in voluntary counselling and testing exposure: results from population based surveys in Kenya, Tanzania and Zambia.

Mercy K. Njeru *[1, 2], Marte Jurgensen [2], Lumbwe Chola[2, 3], Charles Michelo [4], Jens Byskov[5] James Muttunga[1], Senkoro Kesheni [6], Knut Fylkesnes [2]

[1] Centre for Public Health Research, Kenya Medical Research Institute, Nairobi, Kenya

[2] Centre for International Health, University of Bergen, Faculty of Medicine and Dentistry, University of Bergen, Bergen Norway

[3] Central Statistical Office, Box 31908, Lusaka, Zambia

[4] Department of Public Health, University of Zambia

[5] DBL- Centre for Health Research and Development, Faculty of Life Sciences, University of Copenhagen, Frederiksberg, Denmark

[6] National Institute for Medical Research, Dar Es Salaam, Tanzania

*Corresponding author

Email addresses:

MKN: Mercy.Njeru@cih.uib.no

MJ: Marte.Jurgensen@cih.uib.no

LC: Lumbwe.Chola@cih.uib.no

CM: ccmichelo@yahoo.com

JB: jby@life.ku.dk

JM: muttunga@yahoo.com

SK: ksenkoro@nimr.or.tz

KF: knut.fylkesnes@cih.uib.no

ABSTRACT

Background

Delivery of HIV testing and counselling services were substantially scaled up in the mid 1990s employing the voluntary counselling and testing (VCT) model. This model is client initiated and is motivated by an individuals' own right to know their status and who actively seeks VCT. High willingness to be HIV tested has been expressed however studies have reported low uptake of VCT. We aimed at investigating HIV testing exposure and determinants of VCT use in selected districts in Kenya, Tanzania and Zambia.

Methods

Data were from a population-based survey collected as part of the EU funded intervention study project "REsponse to ACountable priority setting for Trust in health systems" (REACT). Stratified random cluster sampling was employed with stratification by district and residence (urban vs. rural). The total number of respondents was 5689 (1847 in Malindi, Kenya; 1992 in Mbarali, Tanzania; and 1850 in Kapiri Mposhi, Zambia). The behavioural model of health services use suggested by Andersen was modified and used for our analysis to fit an assumption on factors involved in seeking VCT services.

Results

The proportions reporting to have ever used VCT varied across district (Malindi 27.7%, Mbarali 15.7% and Kapiri Mposhi 16.2%) and the respective exposures to PMTCT-related testing by gender (male and female) in the districts was: 1.8% and 25.5% in Malindi, 5.3% and 5.0% in Mbarali and 3.2% and 16.2% in Kapiri Mposhi. Overall (n=5689), individuals with higher levels of education were more likely to use VCT compared to those with lower levels (AOR 1.8, 95% CI, 1.46-2.32), and individuals reporting highest level of stigma were less likely to have used VCT vs. lowest (AOR 0.6. 95% CI: 0.43-0.85). Furthermore, there was a strong negative association between educational attainment and stigma in VCT exposure.

Conclusion

The marked variation in exposure to HIV testing in general seemed largely to be explained by the extent in which PMTCT based provider-initiated testing had been scaled up, and exposure to VCT differed to a less extent by district. Stigma appeared to be negatively associated with VCT, and this underscores the importance of more effective efforts to combat stigma and for innovative approaches in implementing HIV testing.

BACKGROUND

HIV testing and counselling (HTC) has since the early history of the HIV pandemic been offered mainly through voluntary counselling and testing (VCT) services. This client initiated model has been defined as a confidential process by which people who are motivated by the need and right to know their status, seek and undergo individual counselling voluntarily, to enable them make informed choices about being tested for HIV [1]. This enabled many to know their sero-status during the testing and ample time was availed for counselling irrespective of the test outcome. It is therefore noteworthy that VCT creates a critical preventive opportunity besides being a prerequisite for access to treatment, care and support services for those infected [1, 2]. Some few years back a new HIV testing model was introduced which in principle sharply contrasted the old model. Testing services within this approach are initiated by the service providers and are applied routinely through an opt-in or opt-out approach at health facilities [3]. However, these services are extensively applied through programmes that only target specific populations, such as pregnant women in the prevention of mother to child transmission (PMTCT) programme. Most nations now aim to apply provider-initiated testing to all patients that visit a health facility [4].

Despite high proportions expressing willingness to be HIV tested and the rapid scale up of services, use of VCT services have been low even when services have been made readily available [5-8]. Explanations have been advanced seeking to explain these varying and low acceptability test rate dynamics. There seem to be consistent evidence that stigma, low trust in the local health care services, confidentiality concerns, and adverse consequences upon disclosure are important barriers to VCT [7-13]. Studies conducted in Zambia and Tanzania have shown inequalities in access, indicated by higher utilization in urban than rural areas and among individuals with higher levels of education compared with groups with low education [7, 14]. National HIV authorities, having declared equal access a central strategic goal, are now

seriously challenged with regards to service design. Facility-based testing has for long been the standard way of offering these services in countries with generalised epidemics, but alternative ways of service delivery (e.g. home based HIV testing) have been shown to be more acceptable and to reduce inequalities [15-17].

The low acceptability of VCT calls for innovative ways of service design. The provider initiated model might be a response in this regard, as HIV test rates have been observed to be increasing in recent years, especially among women in certain settings in Africa, and the scaling up of provider initiated testing could explain part of this [18, 19]. However, there has been growing criticisms to the provider initiated HIV testing concerning the low emphasis put on voluntariness which raises ethical concerns [20-23]. Recent studies suggest that counselling is often limited or left out, and this is paving the way for missed opportunities in prevention and for ethically unacceptable handling of the consent process [24-26] . Further, observed increasing gender differentials in HIV test rates seem largely to be due to the scaling up of the provider initiated model in ante-natal care programs [21, 22, 24].

This study originates from the EU funded five-year intervention study "REsponse to ACcountable priority setting for Trust in health systems" (REACT) at district level in Kenya, Tanzania and Zambia. In this intervention an explicit ethical framework for legitimate and fair priority setting, accountability for reasonableness (AFR) is being promoted to provide guidance for decision-makers to broaden stakeholder involvement and jointly with them identify and consider a wider range of relevant values in priority setting decisions. A baseline was established, and the service and health outcome evaluation concerned changes in quality, equity and trust [27]. HIV control programmes were defined as one of several evaluation domains of the project due to the high HIV prevalence in the project countries [28-30]. The data for the outcome evaluation were not collected as it was evident that the AFR application had not

progressed enough during the project period to expect any associated change in the outcomes. The baseline, however, provides a representative data set to do cross sectional status analyses between the three case studies. The concepts and analytical model in this paper thus provides an alternative approach to gaining added insight associated with equal access in VCT use. In this study we investigated exposure to VCT services and assessed factors that may be associated with use of VCT at district levels in three African countries.

METHODS

Study area and population

A population based survey was conducted in 2007 in three African districts namely; Malindi, Kenya; Mbarali, Tanzania; and Kapiri Mposhi, Zambia, assumed to be comparable in terms of disease burden and health systems, as described elsewhere [27]. HIV is a common concern in the three project countries with a marked geographical prevalence variation. The estimated adult (aged 15-49 years) prevalence in the selected districts is: 16% in Malindi district [31], 7.9% in Mbarali [32] and 13% in the rural and 32.2% in urban Kapiri Mposhi [33, 34]. The population estimates in the study areas were 350,000, 235,000, and 200,000, respectively for Malindi, Mbarali and Kapiri Mposhi districts [32, 35, 36].

Sampling procedures and sample size

A multi-stage stratified random cluster sampling strategy was used to draw the samples. In each district, the sampling frame was stratified into rural and urban areas. For statistical purposes each district is subdivided into Census Supervisory Areas (CSAs) and these are in turn subdivided into Standard Enumeration Areas (SEAs). The CSAs and SEAs are the areal units with unique identification, and the numbers of households were used as a measure of size for selecting SEAs using probability proportional to size of the stratum (stratified by urban and rural). A fixed number of households were selected randomly from every SEA in the rural and urban stratum. At the household level, one male and one female aged 15 and above were randomly selected to participate in the survey. The intended sample size was 2000 adults aged 15 and older in each district. In the sample size calculation the survey was handled as a baseline survey, and the following assumptions were employed: a 0.5 probability of the outcome, a precision of 0.05, minimum measurable change of 0.1, power of 0.80, and the design effect considered at 2. A total of 49 clusters (10, Malindi, 19 Mbarali, and 20 Kapiri

Mposhi) were selected from the urban stratum and 67 clusters (16 Malindi, 26 Mbarali, 25 Kapiri Mposhi) from the rural stratum.

Data collection and organization

Data were collected by trained enumerators using a structured questionnaire and was administered largely in English in Kapiri Mposhi and Swahili in Malindi and Mbarali. The data collection tools were developed and standardised for application in three countries within a standard operating procedure for training of staff, pilot testing of the tools inclusive of revisions and standardised sampling plans for the selection of study participants. Data collected included information on socio-demographic characteristics, education, HIV stigma, HIV risk behaviours, exposure to HIV-related information, cost of health services; self rated health and readiness to re-test.

The model

The behavioural model of health services use that was originally suggested by Andersen was considered and applied to guide variables selection and the analysis of this study, after modifying for VCT use. The Andersen model has been extensively used to explore factors affecting access to health care services and it hypothesises health care use as a function of demographic or predisposing factors, enabling factors and perceived need [37]. The predisposing factors in the original model adapted in this study, included demographic factors (age and gender), and social structure which include education and ethnicity and health beliefs (attitudes and beliefs). Enabling factors include both community and personal enabling resources e.g. income and health insurance. Perceived need and evaluated need make up the need factor. In this model self rated health is key in measuring perceived need whilst evaluated need is achieved after a patient has presented at a health provider [37]. Unequal access is thus seen as occurring when predisposing and enabling variables determine use. For use in this study, the model had to be modified in order to make it applicable for assessment of VCT use,

primarily due to the less obvious need attribute when seeking HIV testing services as compared to seeking care due to an illness, as symptoms or illness may not be the only motivation for seeking HIV testing.

The literature on factors affecting HIV testing was used to guide the inclusion of measures. We hypothesized that predisposing factors are directly or indirectly associated with VCT use. The modified Andersen model that we applied included demographic characteristics, including sex, age and marital status [8, 38-41], together with belief (HIV-related stigma in this case) [7-9, 42, 43] as the predisposing factors. Secondly, we hypothesized that urban residence, educational attainment, exposure to HIV information, and perceived low cost of health care as enabling factors being positively associated with VCT use [7, 8, 40, 41]. The behaviour model includes educational attainment as a predisposing factor, but in our model, this is seen as the key indicator of socioeconomic position and thus relates closely to resources and opportunities. Educational attainment was therefore considered mainly to have an enabling influence. HIV testing presents a unique scenario as compared to illness behaviour, as motivation to test may not only be related to symptoms. To cover this we defined the indicators of need in this study to be self rated health, HIV-related risk behaviour and readiness to test. Self-rated health has been found to be the single most important indicator of health care use in studies from different parts of the world [44, 45] and to be an indicator of deteriorating health related to HIV infection [13]. It is regarded as central in measuring need for health care use as patient's perception of their health is related to their vulnerable state [46]. Readiness (considered as a proxy for intention in this study) to test was considered as a need factor since it has been defined as the proximal determinant of behaviour and has been described as the motivation needed for a particular behaviour [47]. We hypothesized that low self-rated health; HIV-related risk behaviours and readiness to test would positively affect VCT use [13, 37, 48].

Data analysis

Data were double entered in Epi-info and analysis was conducted using SPSS version 15.0. Descriptive statistics were used to present the socio-demographic characteristics, stigma, residence, education, cost, exposure to HIV information, self-rated health, risky behaviour, readiness to test and knowledge of place of testing. Bivariate analyses of VCT use, stratified by district and sex, were used to measure crude odds ratios (ORs) with 95% confidence intervals (CIs). Only those variables that were significant in the bivariate analyses for VCT use qualified for further analyses in the model. A step-wise logistic regression analysis of VCT use was employed. At step I we controlled for predisposing factors, at step II we added the enabling factors, and in the full model (step III), all variables were included (predisposing, enabling and need). The regression analyses were performed in two ways: in one analysis we stratified by district (Table 3) and in the other we pooled together the entire sample (i.e. all the three districts together). Potential interactions were examined both through stratified analyses (district and sex) and in the logistic regression analysis by inclusion of interaction variables (products of original variables) and no significant interactions were found.

Dependent variables

The dependent variables used in this study were based on the following question: "Have you ever been HIV tested? (1 = yes, 2 = no). If that answer was "yes", respondents were asked "Was it voluntarily or required? And the following answer alternatives were mentioned: 1 = VCT-related, 2 = PMTCT-related, 3 = mandatory/required, 4 = other, specify. A dichotomous variable "ever VCT use" (0, 1) was constructed where those responding "VCT-related" were coded 1 and all other respondents coded 0.

Independent variables

The independent variables were categorised into predisposing, enabling and need factors as described above. Age was categorised in five groups as shown in the tables. Marital status was dichotomised to ever married (Single=0 vs. separated, widowed, cohabiting/married=1). Summative indices were made to create a stigma variable from three questions that entailed: a.) buying from a HIV positive shopkeeper, b.) if a HIV positive teacher should be allowed to teach and c.) Whether it should remain a secret if a family member was HIV positive. The stigma variable was categorised into four levels (lowest to highest). For bivariate analysis of association with education, the stigma variable was dichotomised into low or high (Figure 1). Residence was dichotomised into rural=0 vs. urban=1 (peri-urban and urban). Years of education were categorised into four groups as presented in all tables. Two questions phrased as ‘Does the cost of health services requiring immediate cash payment influence your utilisation of them?’ (1 = not at all, 2 = little, 3 = fairly, 4 = much, 5 = very much) and ‘Does the distance to the nearest health services influence your utilisation of them?’ (1 = not at all, 2 = little, 3 = fairly, 4 = much, 5 = very much) were used to construct the cost variable for the HIV information index, the question used was phrased as; ‘How regularly do you receive HIV related information from the following sources?’ Community health workers, Health clinic workers, Peer educators, Media (radio or TV), or other, the responses were: (1 = never, 2 = sometimes, 3 = often): These were dichotomised as 0 = never and 1= sometimes or often. Self-rated health was measured according to established standard in a single question:’ how would you say your health is at the moment?’ The responses ranged from 1=very poor to 5=excellent. These were dichotomised to poor or fair vs. good or excellent (0, 1). To measure HIV-related risk behaviour we employed a question on number of non-marital/non-regular sex partners and this was dichotomised into none vs. one or more (casual sex partners). The single question on readiness was phrased as ‘Have you considered going for a (or another) HIV test?’

(0 = no, 1 = yes). A single question phrased as 'Do you know a place where you can get a confidential HIV test?' (1 = yes, 2 = no) was used to determine knowledge of a test place.

Ethical Clearance

Ethical clearance was obtained from the three countries. In Kenya scientific and ethical approval was obtained from the Kenya Medical Research Institute (KEMRI) and from the National Ethical Review Committee (NERC) of Kenya (KEMRI SSC number 1099). In Tanzania research clearance was obtained from the Medical Research Coordinating Committee (MRCC) of the National Institute of Medical Research (NIMR) number NIMR/HQ/R.8a/Vol.IX/416, and in Zambia from the University of Zambia Research Ethics Committee (assurance No. FWA00000338, IRB00001131 of IOR0000774). Written informed consent was obtained from all participants of the population based survey prior to being interviewed. Confidentiality and anonymity of the study informants was maintained throughout the study.

RESULTS

Survey participation and population characteristics

An overall sample (n= 5689) including participants from all 3 countries was drawn as follows: Malindi, Kenya n=1847, Mbarali, Tanzania n=1992 and Kapiri Mposhi, Zambia n=1850. The overall response rate was 93.4% (n=5689) and non-response did not differ by district. The majority of the study participants were or had been married, and this did not differ much by gender except from Malindi district that had a substantial number of single men (34%). There was a marked difference in educational attainment between Mbarali and the other two districts, with lower educational levels in Mbarali. Moreover, in Malindi and Kapiri Mposhi districts women reported lower educational levels than men, while not much difference in education between men and women was observed in Mbarali district (Table 1).

HIV testing, knowledge and intention to test.

The overall proportion reporting ever being tested for HIV in the three districts was 33.1%. The proportions testing in Malindi (44.2%) was significantly higher ($p < 0.001$) than both Mbarali (27.3%) and Kapiri Mposhi (28.3%). Overall, women were significantly more likely to have ever been tested for HIV than men (38.0% vs. 27.7%, $p < 0.001$). At district level this difference was found both in Malindi (54% vs. 34%) and Kapiri Mposhi (34% vs. 21%), but not in Mbarali (27% vs. 27%). The proportion reporting to have been tested through PMTCT by gender (male and female) was: 1.8% and 25.5% in Malindi, 5.3% and 5.0 in Mbarali and 3.2% and 16.2% in Kapiri Mposhi. Most of the participants (overall 77%) indicated that they knew a place where they could test for HIV (Table 1), but districts differed in this regard, i.e. Malindi 84.6%, Kapiri Mposhi 80.3% and Mbarali 67%. Awareness of where to test did not differ by sex, except in Mbarali where men were significantly ($p<0.001$) more likely to know a test place than the women (Table 1). There was a marked difference in readiness to be tested between districts, i.e. 52.4% in Malindi, 83.3% in Mbarali and 46.1% in Kapiri Mposhi.

Among those stating readiness to be tested in the overall sample, 40.3% (53.7% Malindi, 29.8% Mbarali, and 45.3% Kapiri Mposhi) reported to have been tested for HIV in the past.

VCT utilization and determinants

The proportions reporting to have ever been tested through VCT in the three districts was 20%. This was higher in Malindi (28%) compared to the other two districts (16%) (Table 2).

Predisposing factors

There were observed differences in VCT use by age, with the youngest and oldest age group reporting the lowest levels of VCT use (table 2 and 3). This remained so even after controlling for other variables in the full model (table 4). Table 2 further shows that in the districts with higher proportions of overall HIV testing (Malindi and Kapiri Mposhi) there were no significant gender differences in VCT use, where there was higher utilization of PMTCT based HIV testing among women (table 1). Women in Mbarali district were significantly less likely to use VCT compared to men, and this remained significant after adjusting for other factors in table 3 (AOR 0.6, 95% CI 0.46-0.79). Persons who had ever been married were slightly more likely to use VCT in all the three districts but this was not significant in the model. High levels of stigma were associated with lower levels of VCT use among both men and women (Table2), but in the stratified full model this association was significant only in Malindi district (Table 3). In the full model (n=5689) the likelihood of VCT use was 1.6 higher among those reporting the lowest levels of stigma compared to the group reporting the highest levels of stigma.

Enabling factors

Overall, rural residents were less likely to use VCT (AOR, 0.8, 95% CI, 0.63-0.99), as shown in table 4, but in the stratified analyses this difference remained significant only in Kapiri Mposhi (Table 3). Educational attainment was positively associated with VCT use for both men and women in all the three districts (Tables 2 and 3) and in the full model of the overall analyses (Table 4). Cost was not associated with use of VCT in any of the three districts in our

study hence this variable was not included in the test of the model. In the binary analysis exposure to HIV information was associated with use of VCT in the three districts (Table 2). However, after controlling for need factors in the logistic regression model for VCT this remained significant only in Malindi district (Table 3). Also in the overall sample increasing access to HIV information was slightly associated with increased use of VCT, but this did not remain significant after controlling for the need factors (table 4).

Need factors

In the binary analysis self-rated health was only significantly associated with VCT use among men in Malindi (Table 2), and in the stratified stepwise analyses shown in Table 3 this variable did not appear to be significantly associated with VCT use therefore, it was not included in the overall model (Table 4). Casual sex did not appear to be significantly associated with VCT use in the binary analyses and was therefore excluded for further analysis. Readiness for being HIV tested was strongly associated with VCT use in all the three districts (Table 2) and remained an important determinant in the stratified full model in all the districts (Table 3) and in the full model when considering all districts together (Table 4).

Relationship between education and stigma

Figure 1 illustrates the negative association between education and stigma consistently across the three districts; comparing extremes where the lowest level of education was the reference group (Malindi OR 0.4, 95% CI 0.25-0.50, Mbarali OR 0.3, 95% CI 0.19-0.52, Kapiri Mposhi OR 0.4, 95% CI 0.31-0.54).

DISCUSSION

The results of this study showed marked contrast in HIV testing exposure between Malindi district and the other two districts. This is likely to reflect differences in service availability since the determinants of testing were fairly consistent across the three districts. VCT exposure ranged between 16% and 29% for both men and women in the three districts. PMTCT programme represents an additional opportunity to increase HIV testing levels mainly for child bearing women, and for this testing service the contrast in exposure was more marked compared to VCT. This sharp difference may be a reflection of the contrasts between districts in scaling up of provider initiated testing through PMTCT. For the likelihood of VCT use, the enabling factor which had the greatest effect was educational attainment, with higher test levels among the highest educated. This can be judged as an unequal effect on use. Stigma appeared also to affect VCT use independently. A noteworthy finding in this regard was the strong and negative association between educational attainment and stigma across districts. Given that educational attainment affect stigma, unequal effect of education in regards to VCT use can either be directly associated or indirectly associated through stigma. Consequently, efforts to reduce stigma have potential to affect variations in use. The effects of other enabling factors were small or insignificant.

One of the important objectives of many national HIV programs is equal access to HIV testing. The Andersen behavioural model has mainly been used to measure degree of equal access to health care given equal need in order to guide policies that promote equitable access. In this model, inequity in access is seen to occur when predisposing and /or enabling factors determine who uses health care services [37]. A limitation when applying the model on use of HIV testing services relates to the fact that need is much less clearly defined as compared to help seeking for an acute illness. We applied a combination of variables each assumed to be potential indicators of need, and therefore the plausibility represent a major challenge. This

might be indicated by the fact that the strength of associations of other factors was not substantially affected after adjustment of the need variables, hence analysing equity issues related to VCT use, as demonstrated in this study, was difficult.

Our results show that in these three settings there was unequal access to HIV testing services. We found variations both related to predisposing and enabling factors. Overall, women were more likely to have been tested for HIV than men in Malindi and Kapiri Mposhi, consistent with previous research from other African countries [49-51]. In line with the country progress reports [52, 53], this difference observed in our study is explained by the high test rates through PMTCT programmes in the two districts. Provider-initiated testing among women attending ante-natal clinics can therefore be seen to yield higher test rates among their target group. However, this type of testing, when employed in specialised programmes targeting only women, may introduce a strong gender imbalance in HIV testing exposure by reaching only a very small number of men. Studies have shown that male involvement in PMTCT is a feasible and acceptable strategy that can improve HIV testing [54, 55]. However, despite wide promotion of couple counselling, sufficient implementation has been lacking. Innovative approaches to reach couples are therefore urgently needed. Furthermore, the provider-initiated approach through ANC will not reach all women. Women who are not in their child bearing age or those who choose not to utilise the health facilities will not be reached by these services. VCT, on the other hand, targets both men and women and has been shown to engage men slightly more than women [56].

HIV-related stigma has been defined as the "devaluation of individuals living or associated with HIV/AIDS" [57]. It is a complex phenomenon and has been reported in many studies as one of the strongest barriers to seeking VCT, hence delaying access to prevention, support or treatment [40, 58, 59]. In this study, the likelihood of VCT use was 1.6 times higher among

those scoring lowest on the stigma index vs. those with the highest scores. Lower VCT use among those with higher levels of stigma has also been shown in Malawi, South Africa and Nigeria [5, 9, 60, 61]. This may not be a surprising finding. However, as treatment access is increasing, authors have argued that stigma will decrease as AIDS will become a manageable chronic disease [62]. Contrary to this, our findings point to persistent and prevailing stigma even in settings where treatment is available [52, 53]. Further research is needed to elucidate these arguments. Moreover, studies have shown that alternative service provision, such as home-based or mobile VCT, may yield much higher test proportions, as the stigma associated with clinic-based VCT is decreased [17, 63].

Educational attainment was also shown to be strongly associated with use of VCT in all the three districts, with an overall odds of VCT exposure 1.8 (95% CI 1.46-2.32) when comparing the lowest vs. highest levels of education. This finding concurs with previous research in African settings [7-9, 39, 40]. Education is seen as a key indicator of socioeconomic position and is likely to enable individuals to perceive and act on preventive messages. Furthermore, our results revealed educational attainment to be strongly associated with stigma across the three districts. This is a noteworthy observation. Assuming the relevance of the theory that processes of change in behaviours and attitudes takes place through diffusion of innovation from higher socioeconomic status and downwards [64], it might be suggestive of a scenario where stigma could be reduced starting with the highly educated down to the less educated in the population.

From our results cost did not appear to be associated with VCT use in all the districts. Cost has in the past been noted to be a potential barrier to utilization of health services, particularly among the poor [65]. VCT services differ from other health services in most African settings as they do not require any user fee, being one of the indicator questions for cost in this study. It is

possible the results on cost might have been reversed if enquiry was made on waiting times, travelling cost and other opportunity costs as has been done in a previous study [65].

Among the defined need variables in this study, results indicated that self-rated health, and risk behaviour were not associated with VCT. Illness and treatment access has been described as important motivation for testing. Moreover, self rated health is a significant need factor proposed by Andersen [37]. In the scenario of HIV testing, it may be problematic to assume self-rated health as a need factor since one can stay symptom-free for a long time and many may delay seeking testing possibly due to the long-standing stigma attached to the illness. Risky behaviour was also not positively associated with use of VCT. This confirms previous findings [7], and it has been suggested that those who report high risk behaviours do not necessarily perceive themselves to be at risk, as shown in a study from Ethiopia [66]. The only need factor significantly associated with VCT use was readiness to test, which was the strongest predictor of VCT use in our study. Authors have, however, argued that such a hypothetical question may overestimate readiness in the population, and that different modes of HIV testing service delivery may influence readiness and acceptability [63].

This study shows differentials in factors influencing utilization of both VCT use. We argue that acceptability of HIV testing services can be further addressed by fair and inclusive priority setting mechanisms in these settings, and some studies have shown that when locally accepted testing services are developed with the participation of the community, test rates may be substantially increased [17, 63]. The accountability for Reasonableness (AFR) defined as a fair and ethically based framework for decision making which is employed in the REACT project, draws attention to and aims to improve equity and also gaining trust for health systems. This intended effect of REACT is being assessed in the evaluation of its process of application, but the effect on service and health outcomes has not yet been possible to measure. A recent study

conducted in Tanzania confirms the need for more participatory and mutually accountable processes in priority setting to improve uptake of health services [67]. These decision making mechanism are vital in the context of HIV testing considering that this is critical to HIV prevention, treatment and care. Bridging the access gaps to testing is vital and previous studies have documented alternative ways of offering these services in an acceptable manner through provision of Home based HIV testing [13, 15, 63].

Adopting a cross-sectional study design enabled us to conduct the study in three African districts, hence strengthening the generalizability of the results. However, our results mainly generate associations and limit the ability to draw causal inferences. Stepwise analysis in logistic regression was used to control for confounding. As some of the indicators used in the study were sensitive issues, i.e. sexual behaviour, information bias is likely to have influenced the results to some extent. A well known example that is related to one of the need variables is that women in particular tend to under-report the number of sexual partners [68]. Consistent with this, we note that very few women in all three districts reported casual partners. Comparative information on the availability of HIV testing services was not available in detail in the three districts, and can therefore not exclude the possibility that variation in exposures were affected by factors not measured in our study. We measured individual stigmatizing attitudes with only three questions. It is possible that these questions did not fully capture the different dimensions of individual stigma [69]. In addition, stigma research has been criticized for being too focused on individual factors, overlooking the contextual factors shaping stigma [70, 71]. It is a limitation to our study that we did not investigate community stigma or structural factors.

Conclusion

HIV testing exposure seemed to vary across the districts. This was partly related to the extent to which PMTCT (provider initiated) based testing had been scaled up. Exposure to VCT differed to a less extent by district, but stigma appeared to be associated negatively with VCT use. This underscores the importance of more effective efforts to combat stigma and innovative approaches in implementing HIV testing. Moreover the strong negative association between stigma and education could be suggestive of a need to target those with least education to increase test levels, whilst empowering them with education. The recently introduced provider initiated HIV testing strategies could provide an effective avenue to increase HIV testing in the populations.

Competing interests

The authors declare that they have no competing interests.

Author's contributions

MKN was involved in: conceptualising the objective of this paper, analysing the data, interpreting the findings, literature search and writing of the manuscript. MJ was involved in interpretations of the findings, literature search and extensive revisions of the manuscript. LC participated in literature search and revised the manuscript. CM was involved in the questionnaire development, development of the Standard operating procedures (SOPs), data collection in Kapiri Mposhi district and revised the manuscript. JB was involved in conceptualizing the REACT project, development of the Standard operating procedures and revising the manuscript. JM was involved in the development of the questionnaire, data collection in Malindi district and revision of early drafts of the manuscript. SK was involved in development of the questionnaire and data collection in Mbarali district and revising early

drafts of the manuscript. KF was involved in: conceptualizing the REACT project, development of the questionnaire and the Standard operating procedures (SOPs) as well as the objective of the present manuscript, analysing and interpreting the data and revised the manuscript. All authors read and approved the final manuscript.

Acknowledgements

This study was made possible through funding and support from the European Union sixth framework programme (INCO-2003-A.1.2, contract PL517709) for the Specific Targeted Research and Innovation Project REACT project. The REACT (REsponse to ACcountable priority setting for Trust in health systems) project is a specific targeted research and innovation project whose full title is “Strengthening of fairness and accountability in priority setting for improving equity and access to quality health care at district level in Tanzania, Kenya and Zambia." The authors are grateful to all the study participants in the three countries who provided their time for the interviews and the team from the National Central Bureau of Statistics in all the districts that assisted in collecting the data. Special thanks go to the REACT team members in all the project countries that were involved in the planning and collection of data.

References

1. UNAIDS: **Voluntary Counselling and Testing (VCT)**. Geneva: UNAIDS; 2000.
2. UNAIDS, WHO: **UNAIDS/WHO policy statement on HIV testing**. 2004.
3. WHO, UNAIDS: **Guidance on provider-initiated HIV testing and counselling in health facilities**. Geneva, Switzerland; 2007.
4. National AIDS and STI Control Programme, Ministry of Public health and sanitation Kenya: **National guidelines for HIV testing and counselling in Kenya**. Nairobi 2008.
5. Babalola S: **Readiness for HIV testing among young people in northern Nigeria: The roles of social norm and perceived stigma**. *AIDS Behav* 2006, **11**(10):759-769.
6. Irungu T, Varkey P, Cha S, Patterson J: **HIV voluntary counselling and testing in Nakuru, Kenya: Findings from a community survey**. *British HIV Association HIV Medicine* 2008, **9**(2):111-117.
7. Fylkesnes K, Haworth A, Rosenvard C, Kwapa P: **HIV counselling and testing: Overemphasizing high acceptance rates a threat to confidentiality and the right not to know**. *AIDS* 1999, **13**(17):2469-2474.
8. Sherr L, Lopman B, Kakowa M, Dube S, Chawira G, Nyamukapa C, Oberzaucher N, Cremin I, Gregson S: **Voluntary counselling and testing: uptake, impact on sexual behaviour and HIV incidence in a rural Zimbabwean cohort.** *AIDS* 2007, **21**:851-860.
9. Kalichman SC, L.C. S: **HIV testing attitudes, AIDS stigma, and voluntary HIV counselling and testing in a black township in Cape Town, South Africa**. *Sex Transm Infect* 2003(79):442–447.
10. Njeru MK, Blystad A, Nyamongo IK, Fylkesnes K: **A critical assessment of the WHO responsiveness tool: lessons from voluntary HIV testing and counselling services in Kenya**. *BMC Health Service Research* 2009, **9**(243).
11. Obermeyer MO, Osborn M: **The utilization of testing and counselling for HIV: A review of the social and behavioral evidence**. *Am J Public Health* 2007, **97**(10):1762-1774.
12. Pool R, Nyanzi SI, Whitworth JAG: **Attitudes to voluntary counselling and testing for HIV among pregnant women in rural south-west Uganda**. *AIDS CARE* 2001, **13**(5):605-615.
13. Fylkesnes K, Siziya S: **A randomized trial on acceptability of voluntary HIV counselling and testing**. *Tropical Medicine and International Health* 2004, **9**(5):566-572.
14. Wringe A, Isingo R, Urassa M, Maiseli G, Manyalla R, Changalucha J, Mngara J, S. K, Zaba B: **Uptake of HIV voluntary counselling and testing services in rural Tanzania: implications for effective HIV prevention and equitable access to treatment**. *Tropical Medicine and International Health* 2008, **13**(3):319-327.
15. Mutale W, Michelo C, Jurgensen M, Fylkesnes K: **Home-Based voluntary HIV counselling and testing found highly acceptable and to reduce inequalities**. *BMC public Health* 2010, **10**.
16. Wolff B, Nyanzi B, Katongole G, Ssesanga D, Ruberantwari A, Whitworth J: **Evaluation of a home-based voluntary counselling and testing intervention in rural Uganda**. *Health policy,and planning* 2005, **20**(2):109-116.
17. Negin J, Wariero J, Mutuo P, Jan S, Pronyk P: **Feasibility, acceptability and cost of home-based HIV testing in rural Kenya**. *Tropical Medicine and International Health* 2009, **14**(8):1-7.
18. Creek TL, Ntumy R, Seipone K, Smith M, Mogodi M, Smit M, Legwaila K, Molokwane I, Tebele G, Mazhani L *et al*: **Successful introduction of routine opt-out HIV testing in antenatal care in Botswana**. *J Acquir Immune Defic Syndr* 2007, **45**(1):102-107.

19. April MM, Walensky RP, Chang Y, Pitt J, Freedberg KA, Losina E, Paltiel DA, Wood R: **HIV Testing Rates and Outcome in a South African Community, 2001-2006: Implications for expanded screening policies**. *Journal of Acquired Immne Deficiency Syndromes* 2009, **51**(3):310-316.
20. Yeatman SE: **Ethical and public health considerations in HIV counselling and testing: Policy implications**. *Studies in Family Planning* 2007, **38**(4):271-278.
21. Maman S, King E: **Changes in HIV testing policies and the Implications for Women**. *Journal of Midwifery & Women's Health* 2008, **53**(3):195-201.
22. Rennie S, Behets F: **Desperately seeking targets: the ethics of routine HIV testing in low-income countries**. *Bulletin of the World Health Organization* 2006, **84**(1):52-57.
23. Grovesa AK, Mamana S, Msomib S, Makhanyab N, Moodleyb D: **The complexity of consent: women's experiences testing for HIV at an antenatal clinic in Durban, South Africa**. *AIDS Care* 2010, **22**(5):538-544.
24. Njeru MK, Blystad A, Shayo EH, Nyamongo IK, K F: **Practicing provider initiated HIV testing in high prevalence settings: Consent concerns and missed preventive opportunities** *BMC Health Services Research* 2011 **11**(87).
25. Awiti Ujiji O, Rubenson B, Ilako F, Marrone G, Wamalwa D, Wangalwa G, Ekstrom AM: **Is 'Opt-Out HIV Testing' a Real Option among Pregnant Women in Rural Districts in Kenya?** *BMC Public Health* 2011, **11**(151).
26. Larsson EC, Thorson A, Pariyo G, Conrad P, Arinaitwe M, Kemigisa M, Eriksen J, Tomson G, Ekström AM: **Opt-out HIV testing during antenatal care: experiences of pregnant women in rural Uganda**. *Health Policy Plan* 2011.
27. Byskov J, Bloch P, A. B, Hurtig AK, Fylkesnes K, Kamuzora P, Kombe Y, Kvåle G, Marchal B, Martin DM *et al*: **Accountable priority setting for trust in health systems – the need for research into a new approach for strengthening sustainable health action in developing countries** *BMC Health Research Policy and Systems* 2009, **7**(23):24 October 2009.
28. **Epidemiological fact sheets on HIV and AIDS, Kenya** [http://www.who.int/globalatlas/predefinedReports/EFS2008/full/EFS2008_KE.pdf]
29. **Epidemiological fact sheet on HIV and AIDS, united republic of Tanzania** [http://www.unaids.org/en/CountryResponses/Countries/united_republic_of_tanzania.asp]
30. **Epidemiological fact sheet on HIV and AIDS, Zambia** [http://www.unaids.org/en/CountryResponses/Countries/zambia.asp]
31. Ministry of Finance and planning Kenya: **Malindi district development plan 2002-2008**. Nairobi: Republic of Kenya; 2002-2008.
32. Tanzania Commission for AIDS, Zanzibar AIDS Commission , National Bureau of Statistics, Inc: MI: **Tanzania HIV/AIDS and Malaria Indicator Survey 2007-08 In Preliminary Report,** . 2008:1-58.
33. Fylkesnes K, Ndhlovu Z, Kasumba K, Musonda RM, M. S: **Studying dynamics of the HIV epidemic: population-based data compared with sentinel surveillance in Zambia** *AIDS* 1998, **12**(1227-1234.).
34. Michelo C, Sandøy I, Dzekedzeke K, Siziya S, Fylkesnes K: **Steep HIV prevalence declines among young people in selected Zambian communities (1995-2003)**. *BMC Public Health* 2006, **6**(279): .
35. Ministry of Health Kenya: **Malindi district annual health report 2004**. 2005.
36. Republic of Zambia, Central Statistical Office: **2000 CENSUS OF POPULATION AND HOUSING, ZAMBIA**. 2003.
37. Andersen RM: **Revisiting the behavioural model and access to health care: Does it matter?** . *Journal of health and social behavior* 1995, **36**:1-10.

38. Peltzer K, Matseke G, Mzolo T, Majaja M: **Determinants of Knowledge of HIV status in South Africa: results from a population -based HIV survey**. *BMC Public Health* 2009, **9**(174).
39. MacPhail C, Pettifor A, Moyo W, Rees H: **Factors associated with HIV testing among sexually active South African youth aged 15-24 years**. *AIDS care* 2009, **21**(4):456-467.
40. Hutchinson PL, Mahlalela X: **Utilization of Voluntary counselling and testing services in the Eastern Cape, south Africa**. *AIDS Care* 2006, **18**(5):446-455.
41. Matovu JKB, Gray R, Makumbi F, M.J W, Serwadda D, Kigozi G, Sewankambo NK, Nalugoda F: **Voluntary HIV counselling and testing acceptance, sexual risk behaviour and HIV incidence in Rakai Uganda**. *AIDS* 2005, **19**:503-511.
42. Chesney M, Smith AW: **Critical delays in testing and care: the potential role of stigma**. *Am Behav Scientist* 1999, **42**:1162-1174.
43. Mahajana AP, Saylesc JN, Patela VA, Remiend RH, Sawiresa SR, Ortize DJ, Szekeresa G, Coatesa TJ: **Stigma in the HIV/AIDS epidemic: a review of the literature and recommendations for the way forward**. *AIDS* 2008, **22**(2):67-79.
44. Fylkesnes K, Johnsen R, O: F: **The Tromsø study:factors affecting patient-initiated and provider-initiated use of health care services.** . *Sociology of Health and Illness* 1992, **1**(14):271-297.
45. Manor O, Matthews S, C: P: **Self-rated health and limiting longstanding illness: inter-relationships with morbidity in early adulthood**
International Journal of Epidemiology 2001, 30:600-607. *Journal of Epidemiology* 2001, **30**:600-607.
46. Gelberg L, Andersen RM, Leake BD: **The Behavioral Model for Vulnerable Populations: Application to Medical Care Use and Outcomes for Homeless People**. *Health Services Research* 2000, **34**(6):1273-1302.
47. Armitage CJ, Conner M: **Social cognition models and health behaviour: A structured Review**. *Psychology and Health* 2000, **15**:173-189.
48. Leaitya S, Sherrb L, Wellsa H, Evansa A, Millera R, Johnsona M, Elfordb J: **Repeat HIV testing: high-risk behaviour or risk reduction strategy?** *AIDS* 2000(14):547-552.
49. Mitchell S, Cockcroft A, Lamothe G, Andersson N: **Equity in HIV testing: evidence from a cross-sectional study in ten Southern African countries**. *BMC Int Health Hum Rights* 2010, **10**(1):23.
50. Peltzer K, Matseke G, Mzolo T, Majaja M: **Determinants of knowledge of HIV status in South Africa: results from a population-based HIV survey**. *BMC Public Health* 2009, **9**:174.
51. MacPhail C, Pettifor A, Moyo W, Rees H: **Factors associated with HIV testing among sexually active South African youth aged 15-24 years**. *AIDS care* 2009, **21**(4):456-467.
52. National AIDS Control Council, UNGASS: **United Nations General Assembly Special Session on HIV and AIDS Country Report – Kenya**. 2010.
53. Ministry of Health Zambia, National AIDS council, UNGASS: **Zambia Country Report: Monitoring the Declaration of Commitment on HIV and AIDS and the Universal Access**. 2010.
54. Gliemann JO, Tchendjou PT, Miric M, Gadgil M, Butsashvil M, Eboko F, Perez-Then E, Darak S, Kulkarni S, Kamkamidze G *et al*: **Couple - oriented prenatal HIV counseling for HIV primary prevention: an acceptability study**. *BMC Public Health* 2010, **10**(197).
55. Farquhar C, Kiarie J, Richardson B, Kabura M, John F, Nduati R, Mbori-Ngacha D, John-Stewart G: **Antenatal couple counseling increases uptake of interventions to prevent HIV-1 transmission.** *J Acquir Immune Defic Syndr* 2004 **37**(5):1620-1626.

56. Taegtmeyer M, Kilonzo N, Mung'ala L, Morgan G, Theobald S: **Using gender analysis to build voluntary counselling and testing responses in Kenya**. *The transactions of the Royal society of Tropical Medicine and Hygiene* 2005, **100**:305-311.
57. UNAIDS: **UNAIDS fact sheet on stigma and discrimination.** 2003.
58. Skinner D, Mfecane S: **Stigma, discrimination and the implications for people living with HIV/AIDS in South Africa**. *Journal of Social Aspects of HIV/AIDS* 2004, **1**(3):157-164.
59. Ogden J, Nyblade L: **Common at Its Core: HIV-Related Stigma Across Contexts**. International Center for Research on Women (ICRW). 2005:15-30.
60. Pettifor A, MacPhail C, Suchindran S, Delany-Moretlwe S: **Factors Associated with HIV Testing Among Public Sector Clinic Attendees in Johannesburg, South Africa**. *AIDS Behav* 2010, **14**:913–921.
61. Koku EF: **Desire for, and Uptake of HIV Tests by Ghanaian Women: The Relevance of Community Level Stigma.** *J Community Health* 2010.
62. Abadía-Barrero CE, Castro A: **Experiences of stigma and access to HAART in children and adolescents living with HIV/AIDS in Brazil** *Social Science & Medicine* 2006, **62**(5):1219-1228.
63. Angoti N, Bula A, Gaydosh L, Kimchi EZ, Thornton RL, Yeatman SE: **Increasing the acceptability of HIV counselling and testing with three C's: Convenience, confidentiality and credibility.** *Social Science and Medicine* 2009, **68**(12):2263-2270.
64. Rogers EM: *Diffusion of Innovations*. Fifth edition edition. New York: Free press 2003.
65. Hjortsberg CA, Mwikisa CN: **Cost of access to health services in Zambia** *Health policy and planning* 202, **17**(1):71-77.
66. Sahlu T, Kassa E, Agonafer T, Tsegaye A, Rinke de Wit T, Gebremariam H, Doorly R, Spijkerman I, Yeneneh H, Coutinho RA *et al*: **Sexual behaviours, perception of risk of HIV infection, and factors associated with attending HIV post-test counselling in Ethiopia**. *AIDS* 1999, **13**:1263-1272.
67. Maluka S, Hurtig A, Sebastián M, Shayo EH, Byskov J, Kamuzora P: **Decentralization and health care prioritization process in Tanzania: from national rhetoric to local reality**. *Int J Health Plann Mgmt* 2010, **10**.
68. Sandøy I, Michelo C, Siziya S, Fylkesnes K: **Associations between sexual behaviour change in young people and decline in HIV prevalence in Zambia.** *BMC Public Health 2007 Apr 23;7:60* 2007, **7**(60).
69. Genberga BL, Hlavkab Z, Kondaa KA, Mamanc S, Chariyalertsakd S, Chingonoe A, Mbwambof J, Modibag P, Rooyenh HV, Celentano DD: **A comparison of HIV/AIDS-related stigma in four countries: Negative attitudes and perceived acts of discrimination towards people living with HIV/AIDS**. *Social Science & Medicine* 2009, **68** (12).
70. Castro A, Farmer P: **Understanding and Addressing AIDS-Related Stigma:From Anthropological Theory to Clinical Practice in Haiti**. *Am J Public Health* 2005, **95**(1):53-59.
71. Parkera R, Aggleton P: **HIV and AIDS-related stigma and discrimination:a conceptual framework and implications for action**. *Social Science & Medicine* 2003, **57** 13-24.

Table 1: Socio- demographic characteristics of the respondents by gender

Characteristics		Malindi district		Mbarali district		Kapiri Mposhi district	
Total (N= 5689, 100%)		N=1847, 32.5%		N=1992, 35.0		N=1850, 32.5%	
		Male	Female	Male	Female	Male	Female
		%=50.4 n(931)	%=49.6 n(916)	% = 49.4 n(984)	%=50.6 n(1008)	%=44.3 n(819)	%=55.7 n(1031)
Age	15-19	15.4(142)	12.5(113)	8.4(83)	11.2(113)	9.4(77)	13.9(143)
	20-24	16.3(151)	21.3(192)	13.1(129)	23.9(241)	11.7(95)	20.9(215)
	25-29	13.3(123)	23.3(210)	18.6(183)	22.2(224)	19.1(156)	20.4(210)
	30-39	31(287)	30(271)	35.5(349)	33.3(336)	36.1(294)	30.2(310)
	40-49	24(222)	13(117)	24.4(240)	9.3(94)	23.7(193)	14.5(149)
Marital	Single	33.4(308)	13.6(124)	16.3(160)	10.4(105)	20.9(171)	15.2(157)
	Ever married	66.6(614)	86.4(790)	83.7(824)	89.6(903)	79.1(646)	84.8(873)
Stigma	Lowest stigma	46.7 (405)	37.9(314)	35.5(333)	29.8(273)	34.6(273)	36.1(350)
	Low stigma	25 (217)	27.3(226)	28(262)	26.5(243)	31.1(245)	28.6(277)
	Moderate stigma	21.2 (184)	25.6(212)	29.3(275)	32.5(298)	23.3(184)	22.7(220)
	High stigma	7.1 (62)	9.1(75)	7.1(67)	11.1(102)	11.0(87)	12.6(122)
Residence	Urban	34.4(320)	34.5(316)	47.5(467)	46.4(468)	48.7(399)	50.4(520)
	Rural	65.6 (611)	65.5(600)	52.5(517)	53.6(540)	51.3(420)	49.6(511)
Yrs of Educ	0-6 yrs	37.6 (350)	59.6(546)	20.4(201)	25.6(258)	28.1(230)	42.2(435)
	7 yrs	12 (112)	7.1(65)	69.9(688)	66.7(672)	24.3(199)	25.6(264)
	8-10 yrs	30.2 (281)	21.1(193)	3.9(38)	3.6(36)	26.5(217)	23.7(244)
	>= 11 yrs	20.2 (188)	12.2(112)	5.8(57)	4.2(42)	21.1(173)	8.5(88)
Cost	least costly	54.9	54.3(504)	61.5 (491)	58.6(581)	40.7(578)	46.3(330)
	Costly	45.1	45.7(414)	38.5(414)	41.4(363)	59.3(409)	53.7(480)
HIV info:	None	17.9 (163)	15.9(144)	34.6(340)	26.2(263)	47.2(384)	40.1(411)
	One source	22.4 (204)	25.9(235)	16.0(157)	17.7(178)	26.0(212)	32.5(333)
	Two sources	24.6 (224)	26.6(241)	13.6(134)	21.1(212)	17.9(146)	18.8(193)
	>=3 sources	35.1 (319)	31.6(286)	35.7(351)	35.0(352)	8.8(72)	8.5(87)
Health	Good	70 (642)	63.7(580)	72.2(710)	74.7(753)	81.3(662)	80.4(822)
Casual partner	>=1	21.7 (202)	8.0(73)	8.8(87)	3.0(30)	22.2(182)	10.6(109)
Readiness to test	Yes	54.1(492)	52.5(476)	86.7(835)	84.2(824)	42.4(346)	49.1(503)
Know test place	Yes	85.5 (783)	83.8(760)	71.8((707)	62.5(630)	80.4(656)	80.2((824)
Ever HIV test	Yes	34.2(314)	52.2(494)	27.9(273)	28.5(286)	21.1(173)	34.2(350)
Ever use VCT	Yes	29.3(273)	26.0(238)	18.9(186)	12.6(127)	16.0(131)	16.4(169)
Ever use PMTCT	Yes	1.8(17)	25.5(234)	5.3(52)	5.0(50)	3.2(26)	16.2(167)

Table 2: Determinants by gender on use of VCT

Variable	***Malindi district***				***Mbarali district***				***Kapiri Mposhi district***			
	Male		Female		Male		Female		Male		Female	
Ever tested at VCT	29.3%, n= 931		26%, n=916		18.9 %, n=984		12.6%, n= 1008		16 %, n=819		16.4%, n=1031	
		OR CI		OR CI		OR CI		OR CI		OR CI		OR CI
**Predisposing factors***												
Age: 15-19	12	1	23	1	6	1	10.6	1	13	1	7.7	1
20-24	37.7	4.5(2.39-8.31)	28.6	1.3(0.76-2.36)	19.4	3.8(1.30-10.79)	10.8	1.0(0.56-.1.85)	15.8	1.3(0.56-2.82)	15.8	2.3(1.10-4.60)
25-29	39.8	4.9(2.48-9.54)	25.2	1.1(0.61-2.07)	22.4	4.5(1.48-13.67)	18.8	1.9(1.08-3.50)	14.1	1.1(0.46-2.63)	18.1	2.7(1.25-5.61)
30-39	32.1	3.5(1.94-6.21)	25.5	1.1(0.74-1.75)	20.6	4.1(1.28-12.82)	12.2	1.2(0.67-2.03)	18	1.5(0.79-2.74)	20.1	3.0(1.56-5.77)
40-49	25.2	2.5(1.46-4.21)	25.6	1.1(0.66-1.99)	17.9	3.4(1.16-10.02)	6.4	0.6(0.24-1.37)	15.5	1.2(0.60-2.53)	15.4	2.2(1.14-4.21)
Marital Single	27.6	1	22.6	1	13.8	1	10.5	1	12.3	1	11.5	1
Ever married	30.6	1.2(0.89-1.51)	26.5	1.2(0.77-1.98)	19.9	1.6(0.90-2.69)	12.8	1.3(0.68-2.35)	17	1.5(0.88-2.44)	17.3	1.6(0.94-2.78
Stigma Lowest stigma	36	1	31.5	1	21.3	1	19.4	1	18.3	1	17.1	1
Low stigma	27.2	0.7(0.48-0.91)	27	0.8(0.58-1.11)	21	1.0(0.66-1.46)	13.2	0.6(0.41-0.98)	18	1.0(0.61-1.57)	19.1	1.1(0.74-1.76)
Moderate stigma	25.5	0.6(0.40-0.92)	22.2	0.6(0.37-1.04)	14.9	0.6(0.38-1.11)	8.1	0.4(0.20-0.65)	13	0.7(0.38-1.19)	16.4	0.9(0.60-1.50)
High stigma	14.5	0.3(0.12-0.73)	17.3	0.5(0.19-1.11)	16.4	0.7(0.34-1.53)	5.9	0.3(0.10-0.66)	12.6	0.6(0.31-1.33)	13.1	0.7(0.43-1.24)
**Enabling factors***												
Res: Urban	36.9	1	32	1	17.8	1	12.4	1	21.3	1	19	1
Rural	25.5	0.6(0.38-0.89)	22.8	0.6(0.37-1.07)	19.9	1.2(0.77-1.72)	12.8	1.0(0.65-1.65)	11	0.5(0.29-0.72)	13.7	0.7(0.46-0.99)
Educ yrs 0-6 yrs ξ	24	1	23.3	1	16.4	1	9.3	1	12.6	1	12.2	1
7 yrs	22.3	0.9(0.53-1.57)	27.7	1.3(0.73-2.19)	18.2	1.1(0.66-1.93)	12.8	1.4(0.77-2.69)	12.6	1.0(0.62-1.60)	16.7	1.4(0.91-2.29)
8-10 yrs	31.3	1.4(1.05-1.99)	26.4	1.2(0.78-1.81)	23.7	1.6(0.66-3.79)	11	1.2(0.36-4.17)	16.6	1.4(0.83-2.28)	17.7	1.8(1.10-2.84)
>= 11 yrs	40.4	2.2(1.46-3.16)	37.5	2.0(1.13-3.46)	33.3	2.5(1.19-5.43)	31	4.4(2.00-9.54)	23.7	2.2(1.27-3.64)	27.3	2.7(1.44-5.09)
Cost: least cost	32.6	1	28	1	18.2	1	12	1	16.7	1	16.2	1
Costly	27.4	0.8(0.54-1.12)	24.2	0.8(0.59-1.15)	19.4	1.1(0.77-1.54)	13.5	1.1(0.77-1.71)	14.5	0.9(0.57-1.27)	16.6	1.0(0.77-1.40)
HIV info: None	20.2	1	18.1	1	17.4	1	8.7	1	13	1	17.3	1
One source	38.4	1.6(1.00-2.45)	25.1	1.5(0.92-2.53)	17.2	1.0(0.60-1.63)	9.6	1.1(0.51-2.38)	17.5	1.4(0.89-2.24)	14.1	0.8(0.52-1.18)
Two sources	32.6	1.9(1.27-2.85)	32.8	2.2(1.32-3.71)	22.4	1.4(0.81-2.34)	13.2	1.6(0.90-2.81)	16.4	1.3(0.77-2.24)	15	0.8(0.52-1.38)
>=3 sources	33.2	2.0(1.17-3.28)	25.5	1.6(0.84-2.90)	19.9	1.2(0.82-1.71)	16.5	2.1(1.23-3.45)	25	2.2(1.18-4.19)	25.3	1.6(0.89-2.95)
**Need factors***												
Health: Poor / fair	22.6	1	26	1	17.9	1	11.8	1	11.8	1	19.5	1
Good /excellent	32.4	1.6(1.13-2.38)	26	1.0(0.78-1.29)	19.3	1.0(0.79-1.52)	12.9	1.1(0.63-1.94)	16.9	1.5(0.92-2.49)	15.7	0.8(0.49-1.21)
Casual partner None	28.3	1	26.6	1	18.6	1	12.4	1	17.1	1	16.3	1
> =1	33.2	1.3(0.90-1.76)	30.1	1.3(0.74-2.13)	21.8	1.2(0.73-2.05)	20.0	1.8(0.69-4.58)	12.1	0.7(0.45-1.00)	17.4	1.1(0.70-1.68)
Readiness No	17.7	1	19.3	1	7.8	1	8.4	1	7.2	1	7.5	1
Yes	40.4	3.1(1.85-5.37)	32.4	2.0(1.38-2.90)	21.1	3.2(1.70-5.85)	13.8	1.8(0.86-3.57)	28	5.0(3.27-7.63)	25.8	4.3(2.96-6.26)

This table presents proportions and crude odds ratio and 95% CI of bi-variates analysis

Table 3: Determinants of VCT utilization using Andersen's model by district

Variable		*Malindi, N=1847*			*Mbarali, N= 1992*			*Kapiri Mposhi, N=1850*		
		**Step I* ADJ OR 95%CI**	**Step II** ADJ OR 95%CI**	**Step III*** ADJ OR 95%CI**	**Step I* ADJ OR 95%CI**	**Step II** ADJ OR 95%CI**	**Step III*** ADJ OR 95%CI**	**Step I* ADJ OR 95%CI**	**Step II** ADJ OR 95%CI**	**Step III*** ADJ OR 95%CI**
Predisposing factors										
**Age:**	15-19	1	1	1	1	1	1	1	1	1
	20-24	2.4(1.63-3.63)	2.2(1.45-3.21)	2.1(1.41-3.13)	1.5(0.90-2.55)	1.5(0.86-2.54)	1.4(0.83-2.46)	1.7(0.87-3.14)	1.4(0.74-2.72)	1.4(0.71-2.67)
	25-29	2.1(1.30-3.46)	1.8(1.15-2.86)	1.8(1.10-2.89)	2.2(1.32-3.80)	2.1(1.26-3.64)	2.1(1.22-3.56)	1.7(0.81-3.58)	1.6(0.73-3.32)	1.6(0.75-3.45)
	30-39	2.0(1.26-3.25)	1.7(1.09-2.77)	1.8(1.14-2.74)	1.7(0.98-2.87)	1.6(0.90-2.71)	1.6(0.91-2.73)	2.0(1.03-4.03)	1.7(0.87-3.47)	2.0(1.01-4.01)
	40-49	1.6(0.89-2.86)	1.5(0.86-2.71)	1.5(0.88-2.67)	1.3(0.71-2.39)	1.2(0.65-2.27)	1.2(0.65-2.30)	1.6(0.77-3.18)	1.4(0.67-2.82)	1.7(0.83-3.40)
**Sex:**	Male	1	1	1	1	1	1	1	1	1
	Female	0.8(0.61-1.08)	0.8(0.61-1.11)	0.8(0.60-1.10)	0.6(0.48-0.79)	0.6(0.46-0.78)	0.6(0.46-0.79)	1.1(0.78-1.50	1.2(0.84-1.70)	1.1(0.75-1.55)
**Marital S.**	Single	1	1	1	1	1	1	1	1	1
	Ever Married	1.0(0.68-1.54)	1.1(0.74-1.66)	1.2(0.84-1.80)	1.2(0.70-1.97)	1.3(0.78-2.18)	1.3(0.80-2.21)	1.2(0.68-2.00)	1.5(0.81-2.64)	1.3(0.73-2.34)
**Stigma:**	Lowest Stigma	1	1	1	1	1	1	1	1	1
	Low stigma	0.7(0.58-0.92)	0.8(0.63-0.98)	0.8(0.64-1.04)	0.8(0.63-1.08)	0.8(0.63-1.11)	0.8(0.63-1.12)	1.1(0.74-1.52)	1.1(0.79-1.63)	1.1(0.76-1.54)
	Moderate stigma	0.6(0.41-0.93)	0.7(0.52-1.06)	0.8(0.54-1.11)	0.5(0.35-0.77)	0.6(0.37-0.85)	0.6(0.37-0.88)	0.8(0.55-1.11)	0.9(0.66-1.33)	1.0(0.72-1.52)
	High stigma	0.4(0.21-0.76)	0.5(0.25-0.91)	0.5(0.24-0.89)	0.5(0.25-0.90)	0.6(0.29-1.05)	0.6(0.30-1.10)	0.7(0.43-1.07)	0.7(0.46-1.15)	1.0(0.61-1.52)
Enabling factors										
**Residence:**	Urban		1	1		1	1		1	1
	Rural		0.7(0.48-1.14)	0.7(0.45-1.06)		1.1(0.70-1.45)	1.1(0.74-1.55)		0.7(0.47-0.96)	0.6(0.44-0.90)
**Yrs of Educ.**	0-6 yrs		1	1		1	1		1	1
	7yrs		0.9(0.65-1.37)	1.0(0.68-1.39)		1.1(0.74-1.74)	1.1(0.72-1.72)		1.1(0.80-1.62)	1.1(0.78-1.60)
	8-10yrs		1.1(0.78-1.47)	1.0(0.74-1.48)		1.4(0.63-3.10)	1.3(0.59-2.95)		1.4(0.91-2.18)	1.3(0.82-2.05)
	> = 11yrs		1.5(1.14-1.88)	1.5(1.14-1.97)		2.8(1.65-4.87)	2.6(1.53-4.59)		2.0(1.24-3.35)	1.9(1.10-3.25)
**HIV info:**	None		1	1		1	1		1	1
	One source		1.4(1.05-1.85)	1.3(1.04-1.75)		0.9(0.56-1.35)	0.9(0.55-1.33)		1.1(0.84-1.41)	0.9(0.70-1.15)
	Two sources		1.8(1.28-2.42)	1.7(1.23-2.22)		1.3(0.86-2.05)	1.3(0.84-2.02)		1.0(0.63-1.44)	0.7(0.45-1.03)
	>=3 sources		1.4(0.91-2.13)	1.3(0.87-2.03)		1.3(0.93-1.80)	1.2(0.89-1.74)		1.7(1.02-2.76)	1.2(0.72-1.85)
Need factors										
**Health:**	Poor / fair			1			1			1
	Good / excellent			1.2(0.93-1.59)			1.0(0.71-1.33)			0.9(0.65-1.23)
**Readiness:**	No			1			1			1
	Yes			2.5(1.66-3.75)			2.3(1.36-3.93)			4.6(3.45-6.02)

*No significant interaction found so we did not stratify by gender. Step I: controlled for predisposing factors Nagelkerke's R^2 (Malindi 0.023, mbarali 0.027, Kapiri mposhi 0.011), ** step II controlled for enabling factors. Nagelkerke's R^2 (Malindi 0.065, mbarali 0.065, Kapiri mposhi 0.053) *** step III controlled for the need factors Nagelkerke's R^2 (Malindi 0.117, mbarali 0.077, Kapiri mposhi 0.155). NB:Only factors that were significant as indicated in table 2 were included..*

Table 4: Results of logistic regression analysis of VCT utilization

Variable		19.8%, N= 5689			
		**Bivariates**	**Step I*** **ADJ OR 95%CI**	**Step II**** **ADJ OR 95%CI**	**Step III***** **ADJ OR 95%CI**
Predisposing factors					
**Age:**	15-19	1	1	1	1
	20-24	1.9(1.47-2.46)	2.0(1.43-2.68)	1.8(1.33-2.41)	1.8(1.30-2.36)
	25-29	2.1(1.59-2.71)	2.1(1.47-2.96)	1.9(1.37-2.64)	1.9(1.36-2.69)
	30-39	1.9(1.55-2.44)	2.0(1.42-2.79)	1.7(1.25-2.40)	1.8(1.33-2.53)
	40-49	1.7(1.27-2.16)	1.6(1.12-2.36)	1.5(1.01-2.09)	1.5(1.06-2.20)
**Sex:**	Male	1	1	1	1
	Female	0.8(0.69-0.93)	0.8(0.69-.096)	0.8(0.70-1.00)	0.8(0.68-0.98)
**Marital S.**	Single	1	1	1	1
	Ever Married	1.1(0.92-1.43)	1.0(0.71-1.27)	1.2(0.91-1.60)	1.2(0.95-1.63)
**Stigma**	Lowest stigma	1	1	1	1
	Low stigma	0.8(0.67-0.95)	0.8(0.68-0.95)	0.9(0.75-1.059	0.9(0.74-1.05)
	Moderate stigma	0.6(0.46-0.74)	0.6(0.46-0.74)	0.7(0.57-0.88)	0.7(0.58-0.92)
	High stigma	0.5(0.32-0.63)	0.5(0.33-0.65)	0.6(0.40-0.78)	0.6(0.43-0.85)
Enabling factors					
**District:**	Malindi	1		1	1
	Mbarali	0.5(0.37-0.65)		0.5(0.38-0.69)	0.4(0.26-0.50)
	Kapiri Mposhi	0.5(0.38-0.67)		0.5(0.41-0.70)	0.5(0.41-0.69)
**Residence:**	Urban	1		1	1
	Rural	0.8(0.61-1.03)		0.8(0.65-1.01)	0.8(0.63-0.99)
**Yrs of Educ.**	0-6yrs	1		1	1
	7yrs	0.9(0.73-1.16)		1.0(0.86-1.29)	1.1(0.86-1.29)
	8-10 yrs	1.5(1.18-1.81)		1.2(0.98-1.55)	1.2(0.93-1.51)
	> = 11yrs	2.3(1.82-2.92)		1.8(1.44-2.24)	1.8(1.46-2.32)
**HIV info:**	None	1		1	1
	One source	1.3(1.03-1.53)		1.1(0.95-1.35)	1.0(0.87-1.24)
	Two sources	1.6(1.29-2.06)		1.3(1.08-1.66)	1.2(0.96-1.49)
	>=3 sources	1.7(1.38-2.11)		1.3(1.06-1.68)	1.2(0.93-1.50)
Need factor					
**Readiness:**	No	1			1
	Yes	2.5(1.96-3.10)			3.0(2.32-3.87)

Step I: controlled for predisposing factors ***Nagelkerke's R^2 0.014*** *, ** step II controlled for enabling factor* ***Nagelkerke's R^2 0.057*** *s.*** step III controlled for the need factors* ***Nagelkerke's R^2 0.095****. NB: Only factors that were significant as indicated in table 2 were included.*

Figure 1. The association between stigma and educational attainment

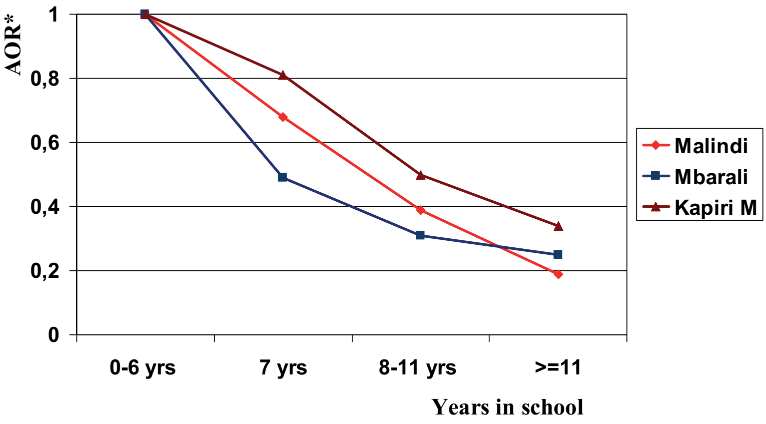

* AOR: Age adjusted odds ratio, High Stigma score vs. low

Stigma 0= Lowest stigma and 1= highest stigma (cut-off point in dichotomising stigma was into score 0(lowest stigma) to 1(low stigma) and 2(moderate stigma) to 3(High stigma)

II

Njeru et al. BMC Health Services Research 2011, 11:87
http://www.biomedcentral.com/1472-6963/11/87

BMC Health Services Research

RESEARCH ARTICLE **Open Access**

Practicing provider-initiated HIV testing in high prevalence settings: consent concerns and missed preventive opportunities

Mercy K Njeru[1,2*], Astrid Blystad[3,2], Elizabeth H Shayo[4,3,2], Isaac K Nyamongo[5] and Knut Fylkesnes[2]

Abstract

Background: Counselling is considered a prerequisite for the proper handling of testing and for ensuring effective HIV preventive efforts. HIV testing services have recently been scaled up substantially with a particular focus on provider-initiated models. Increasing HIV test rates have been attributed to the rapid scale-up of the provider-initiated testing model, but there is limited documentation of experiences with this new service model. The aim of this study was to determine the use of different types of HIV testing services and to investigate perceptions and experiences of these services with a particular emphasis on the provider initiated testing in three selected districts in Kenya, Tanzania, and, Zambia.

Methods: A concurrent triangulation mixed methods design was applied using quantitative and qualitative approaches. A population-based survey was conducted among adults in the three study districts, and qualitative data were obtained from 34 focus group discussions and 18 in-depth interviews. The data originates from the ongoing EU funded research project "REsponse to ACountable Priority Setting for Trust in Health Systems" (REACT) implemented in the three countries which has a research component linked to HIV and testing, and from an additional study focusing on HIV testing, counselling perceptions and experiences in Kenya.

Results: Proportions of the population formerly tested for HIV differed sharply between the study districts and particularly among women (54% Malindi, 34% Kapiri Mposhi and 27% Mbarali) ($p < 0.001$). Women were much more likely to be tested than men in the districts that had scaled-up programmes for preventing mother to child transmission of HIV (PMTCT). Only minor gender differences appeared for voluntary counselling and testing. In places where, the provider-initiated model in PMTCT programmes had been rolled out extensively testing was accompanied by very limited pre- and post-test counselling and by a related neglect of preventative measures. Informants expressed frustration related to their experienced inability to 'opt-out' or decline from the provider-initiated HIV testing services.

Conclusion: Counselling emerged as a highly valued process during HIV testing. However, counselling efforts were limited in the implementation of the provider-initiated opt-out HIV testing model. The approach was moreover not perceived as voluntary. This raises serious ethical concerns and implies missed preventive opportunities inherent in the counselling concept. Moreover, implementation of the new testing approach seem to add a burden to pregnant women as disproportionate numbers of women get to know their HIV status, reveal their HIV status to their spouse and recruit their spouses to go for a test. We argue that there is an urgent need to reconsider the manner in which the provider initiated HIV testing model is implemented in order to protect the client's autonomy and to maximise access to HIV prevention.

* Correspondence: mercy.njeru@cih.uib.no
[1]Centre for Public Health Research, Kenya Medical Research Institute, Nairobi, Kenya
Full list of author information is available at the end of the article

BioMed Central

© 2011 Njeru et al; licensee BioMed Central Ltd. This is an Open Access article distributed under the terms of the Creative Commons Attribution License (http://creativecommons.org/licenses/by/2.0), which permits unrestricted use, distribution, and reproduction in any medium, provided the original work is properly cited.

Background

HIV counselling and testing (HCT) services are a crucial part of prevention and are necessary as a prerequisite for supporting, caring and providing treatment to the HIV infected. Preventive counselling gives individuals an opportunity to receive relevant information, to correct misconceptions about HIV, to assess risk and to motivate behaviour change if necessary [1,2]. Preventive effects of the voluntary counselling and testing (VCT)-package (risk-reduction counselling in relation to testing) are reasonably well documented [3,4], but to what extent knowledge of ones own status alone leads to behaviour change is difficult to address in ethically acceptable research efforts. HCT also has further been noted to have the potential to encourage openness, hence contributing to the reduction of fear and stigma in society [1,2,5]. Despite the assumed benefits and scaling-up of HCT services over the last decade the demand for the services has been disappointingly low in countries with the most serious epidemics [6-9]. This has resulted in efforts to establish alternative HIV testing models such as the provider-initiated (PITC) model, which differs substantially from the established client-initiated model.

Client-initiated HIV counselling and testing is commonly referred to as voluntary counselling and testing (VCT). It is motivated by an individual's right to know his or her HIV status, and it takes place when an individual seeks counselling and testing at a facility that offers the services. This type of HIV testing and counselling service is offered widely, mainly through facilities integrated in health settings, mobile services and in stand-alone facilities that are located away from health care facilities such as community-based settings and night services [5,9-15]. Provider-initiated testing in contrast to the client initiated testing is recommended by a health provider to people attending a health facility [15]. This approach to testing can be offered with an opt-in or an opt-out approach, the latter being prominent in specialized programmes such as prevention of mother-to-child-transmission programmes (PMTCT) [15]. The difference between the two is that with the opt-in approach patients need to affirmatively agree to test before the test is conducted, whereas in opt-out approaches clients must actively decline after the pre-test information is offered, if they do not want the test to be performed [15]. Offering testing to all individuals seeking health care services is assumed to increase HIV test rates and thus improve access to treatment. HIV testing has been recommended for all pregnant women, but with a preservation of the woman's right to decline or opt- out. Those who decline are however encouraged to undergo HIV testing at a subsequent visit [15].

The new guidelines recommend pre-test information in place of pre-test counselling when practicing PITC [15]. However, critics argue that individuals tested for HIV must be allowed to evaluate the information provided to them during individual pre-test counselling sessions, and come to their own conclusion regarding whether or not they wish to be tested [16,17]. This is deemed vital for ensuring trust in the health services not the least in the prevention, care and treatment of HIV. A trusting relationship between the health care provider and patient (interpersonal trust) within the context of HIV testing has been seen as essential in enhancing the quality of the interaction and for constructively encouraging necessary behaviour change [18].

The increase in HIV test rates documented in certain settings has been partly attributed to a rapid scale-up of provider-initiated testing services [19-21]. However, the roll-out of this testing model has been criticised from an ethical and human rights perspective for paving the way to neglect of informed consent [16,17,22], and for reducing the amount of counselling that accompanies the HIV test [15,23]. Despite the early voicing of ethical and human rights concerns related to the provider-initiated model of HIV testing, there is little empirical evidence from Africa related to client experiences with this model of testing. This study is an attempt to explore the perceptions and experiences of the testing services with a particular emphasis on the provider initiated opt-out HIV testing as practised at Antenatal Clinics (ANC) where the PMTCT programme is located. Moreover we investigated the exposure to HIV testing in the adult populations in three African districts: Malindi, Kenya; Mbarali, Tanzania; and Kapiri Mposhi in Zambia, where the EU-funded multi-country project, REACT (Response to A Countable priority setting for Trust in health systems) is currently implemented. The project draws upon the ethical framework 'Accountability for Reasonableness' (AFR), for legitimate and fair priority setting. It is applied to provide decision makers with guidance to enhance trust, quality and equity in health systems [24].

Methods

Study design

A mixed method approach was applied in which quantitative and qualitative data were collected using a concurrent triangulation design [25] as illustrated in Figure 1. Quantitative methods were used to determine the proportions of people undergoing HIV tests and the proportions utilizing the various HIV testing services. The qualitative in-depth interviews (IDIs) and focus group discussions (FGDs) were employed to explore informants' experiences and perceptions of the HIV testing services

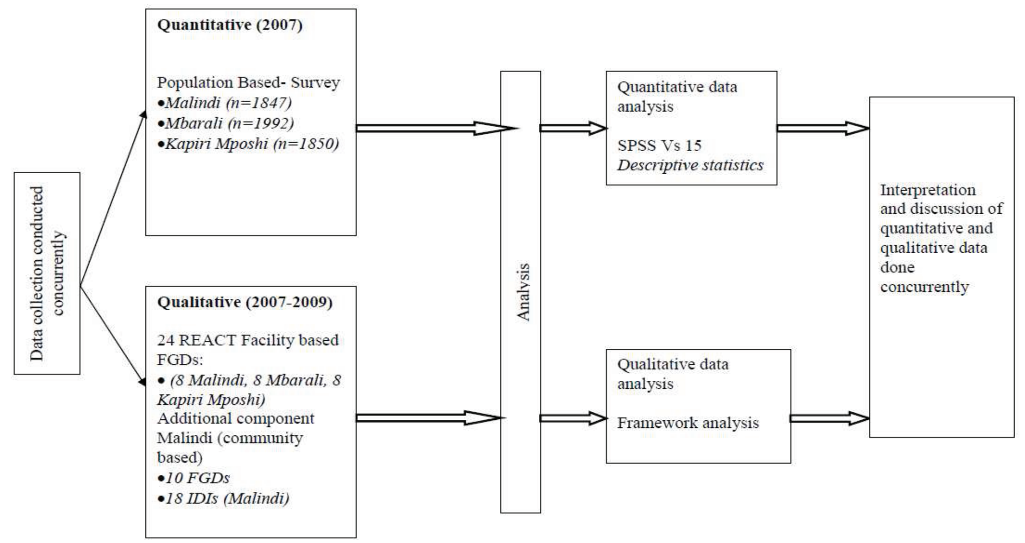

Figure 1 Study design.

with an emphasis on experiences with the provider initiated testing model.

Study area and population

The study was carried out at three different study sites, namely: Malindi district in Kenya, Mbarali district in Tanzania and Kapiri Mposhi district in Zambia. The districts were selected on the basis of assumed similarities [24], but closer look at the health statistics reveals differences in key indicators, among them HIV prevalence. HIV control programmes were defined as one of several evaluation domains of the REACT project. The national HIV prevalence levels within these countries show marked geographical variation; the overall national levels for adults aged 15-49 years have been estimated at 7.7% in Kenya [26], 6.2% in Tanzania [27] and 15.2% in Zambia [28]. In the selected study districts the HIV prevalence's are estimated at: 16% in Malindi district [29], 7.9% in Mbarali district [30], and 13% in rural and 32.2% in urban Kapiri Mposhi district [31,32]. Malindi has an estimated population of 350,000 [29,33], Mbarali 235,000 [30] and Kapiri Mposhi 300,000.

Quantitative study

The quantitative study conducted in 2007 comprised a population-based survey that identified household members at community level in the three districts as part of the REACT project. A stratified random cluster sampling technique was employed with stratification by district and urban vs. rural populations. The intended sample size was 2000 in each district. The samples were selected from the national statistical master sample frames in all three districts. The steps in sampling were random selection by probability proportional to size of enumeration areas at the strata (clusters); random selection of an equal number of households within each of the randomly selected clusters; and random selection of one male and one female from each household as study participants.

Data collection and analysis

A questionnaire was administered in English in Kapiri Mposhi and Swahili in Malindi and Mbarali. Socio-demographic measures included age, residence, sex, marital status, educational attainment and the times of visits to different types of health facilities during the previous 12 months. Other measures included previous HIV testing and the type of testing (e.g. client-initiated VCT or provider-initiated PMTCT). Data were entered in Epi-info and analysed using SPSS version 15 for Microsoft Windows. The Pearson chi-square test was used to evaluate differences between groups regarding proportions of HIV test exposure and socio-demographic characteristics. P-values below 0.05 were considered statistically significant.

Qualitative study

The qualitative data set originates from two sources. The first set consists of 24 FGDs (eight from each country) conducted in 2007 in Mbarali and in 2008 in Malindi and

Kapiri Mposhi as part of the REACT project. These FGDs were carried out among individuals seeking health services at health care facilities that served the sampled clusters. The second data set consists of 10 FGDs and 18 in-depth interviews (IDIs) conducted in one of the study districts (Malindi) in February 2009. The FGDs in both data sets explored the practices, ideas and experiences related to HIV testing models. The IDIs explored more thoroughly experiences and perceptions related to the provider initiated opt-out strategy.

Recruitment of informants and data collection

Participants and the health facilities in the first set of 24 FGDs were recruited purposely. The groups consisted of: female out-patients, male out-patients, pregnant women attending ante-natal clinics and youths aged 18 to 24 years. Questions developed for use in the population-based survey guided the development of the topic guides used for this set of FGDs. The FGD's were conducted by trained social scientists. In the second phase of data collection, in the sub study carried out in Malindi, 10 FGDs and 18 IDIs were conducted at community level. The age of the FGD participants ranged between 18 years and 68 years while that of the IDI participants ranged between 18 to 50 years. Informants for these group discussions and interviews were recruited in urban and rural settings among five categories of informants: female youths, female adults, male youths, male adults and pregnant women. The interviews in the second phase of data collection were conducted by the first author (MKN) with assistance from an experienced social scientist with post-graduate training.

For each FGD (in both data sets) and IDI socio-demographic data on age, marital status, level of education, occupation and spouse occupation were recorded. The purpose of the meeting and the main themes for discussion were introduced by the moderator prior to the start of the sessions. Participants were given the chance to discuss the given themes thoroughly and emerging ideas were followed up with further questions. Observations of the group dynamics during discussions were recorded by an assistant. The FGDs lasted 2 to 3 hours while the IDIs lasted between 1 and 2 hours. All the conversations were audio-taped.

The contents of the FGDs and IDIs were first transcribed verbatim and carefully translated to English - within the same document - with emphasis on retaining the meaning of the culturally-embedded concepts and expressions. 'Framework analysis', a recognised framework for applied qualitative research was employed. Data analysis for all the data sets involved five main steps: familiarization, identification of a framework, indexing, charting and interpretation [34,35]. The main analysis was carried out manually by four of the authors who speak Swahili (MKN, AB, EHS and IKN). Familiarization with the data implied immersion in the raw data several times through listening to the tapes and reading the full sets of transcripts. A framework was developed from emerging issues identified during the familiarization stage. The main emerging themes were related to what was experienced as the value of counselling and the challenges experienced with the implementation of the PITC model of HIV testing by the study participants. These themes were further explored through a thorough and time consuming review of each transcript. Codes were written on the margins of the transcripts leading to the development of a chart matrix. The chart was thoroughly discussed and interpreted in meetings among all the co-authors.

Ethical aspects

Ethical clearance for the umbrella project (REACT) was obtained from research clearance organizations in the three countries prior to the study; in Kenya scientific and ethical approval was obtained from the Kenya Medical Research Institute (KEMRI) and from the National Ethical Review Committee (NERC) of Kenya. In Tanzania research clearance was obtained from the Medical Research Coordinating Committee (MRCC) of the National Institute of Medical Research (NIMR), and in Zambia from the University of Zambia Research Ethics Committee. Written informed consent was obtained from all participants of the population-based survey prior to the interviews, and oral consent was obtained for the FGDs and IDIs. Confidentiality and anonymity of the study informants were emphasised and maintained throughout the study.

Results

Participation in the survey

A total of 6088 persons were sampled, and the achieved sample size was almost equal among district and urban/rural strata except for Malindi where the rural stratum was over-sampled. The overall response rate was 93.4% (n = 5689) and non-response did not differ by district.

Population characteristics and health care use

The project districts differed with regard to the educational attainment of the participants, and there was a particular contrast between Mbarali and the other two districts; e.g. in the urban population the proportion with nine or more years in school was 9.7% in urban Mbarali, compared to 48% in urban Malindi ($p < 0.001$) and 44.8% in Kapiri Mposhi ($p < 0.001$. Malindi and Kapiri Mposhi were characterised by a marked differential in the level of education in general, and with inequalities between the urban and rural populations in particular; e.g. the proportions of urban versus rural populations with nine or more years of schooling were

48% versus 16.4% in Malindi ($p < 0.001$) and 44.8% versus 22.6% in Kapiri Mposhi ($p < 0.001$). In contrast, the population in Mbarali appeared to have small attainment differentials, e.g. more than 70% spent 5-8 years in school and there were no urban/rural differentials (Table 1). There was a higher likelihood of being married in the rural populations in the three countries. Self-reported health care use among the survey participants indicated that the health care system was solely public in Kapiri Mposhi, in contrast to the situation in Malindi where there was substantial use of private facilities and in Mbarali where there was a mixture of public, private and faith-based facilities.

Exposure to HIV Testing

Close to a third (33.5%, n = 5649) of the respondents had been tested for HIV, but there were differences among districts (Malindi 44.2%, n = 1829; Mbarali 27.3%, n = 1982; Kapiri Mposhi 28.3%, n = 1838). Women were significantly ($p < 0.001$) more likely to have been tested than men (38%, n = 2936 vs. 28%, n = 2710). These differences between men and women were also reflected within the districts (Figure 2). Women in Malindi were more likely to have been tested than women in Mbarali (54%, n = 911 vs. 28%, n = 1004) and Kapiri Mposhi (54% vs. 34%, n = 1023) ($p < 0.001$), while women in Kapiri Mposhi were more likely to have been tested than women in Mbarali (Figure 2). Only minor gender differences were observed for VCT based testing, mainly in Mbarali district (Figure 2). In the Mbarali district, about 9% of the women indicated they had been tested in other places e.g. private clinics and hospitals. In the same district, the likelihood of having been tested did not differ by gender, and there was a relatively low coverage of PMTCT based testing. Malindi district had a notably higher testing rate than the other two regarding HIV tests through VCT (Figure 2).

Perceptions and experiences with counselling during HIV testing

In the following section findings from the focus group discussions and the in-depth interviews are presented.

The value of counselling in HIV testing

During the interviews, counselling was brought up by the informants as an essential and appreciated part of the HIV testing regimes. Both the prevention and the support aspects emerged as highly valued parts of these sessions.

The preventive aspect

The preventive opportunities inherent in the counselling concept emerged as important in our interviews and discussions.

> *"The importance of counselling emerges when a person who has not been infected gets advice and follows it, because s/he[1] will not get this disease."* (Female 26 years old, urban Malindi). *"Counselling is very important because if you are counselled you get the courage*

Table 1 Socio-demographic characteristics of the respondents

Characteristics	*Malindi N = 1847*				*Mbarali N = 1992*				*Kapiri Mposhi N = 1850*			
**Residence**	**Urban**		**Rural**		**Urban**		**Rural**		**Urban**		**Rural**	
	**%**	**n**	**%**	**n**	**%**	**n**	**%**	**n**	**%**	**n**	**%**	**n**
**Age**												
15-19	10.9	(68)	15.5	(187)	12.4	(116)	7.6	(80)	12.1	(111)	11.7	(109)
20-24	23.9	(149)	16.1	(194)	18.7	(175)	18.4	(195)	18.1	(166)	15.5	(144)
25-29	21.2	(132)	16.8	(203)	19.9	(186)	20.9	(221)	20.0	(184)	19.5	(182)
30-39	30.3	(184)	30.6	(369)	32.2	(301)	36.3	(384)	33.2	(302)	32.4	(302)
40-49	13.8	(88)	21.0	(253)	16.8	(157)	16.7	(177)	16.2	(148)	20.8	(194)
**Sex**												
Male	50.6	(318)	50.3	(613)	49.9	(467)	48.9	(517)	43.4	(399)	45.1	(420)
Female	49.4	(310)	49.7	(606)	50.1	(468)	51.1	(540)	56.6	(520)	54.9	(511)
**Marital status**												
Single	28.8	(180)	20.8	(252)	16.8	(157)	10.2	(108)	20.6	(189)	14.9	(139)
Ever Married	63.1	(394)	74.2	(899)	83.1	(777)	89.6	(947)	70.6	(647)	75.5	(703)
**Yrs of Educ**												
0 yrs	7.8	(49)	29.8	(363)	10.9	(102)	10.8	(114)	3.9	(36)	7.5	(70)
1-4 yrs	11.9	(75)	15.6	(190)	7.2	(67)	6.5	(69)	7.3	(67)	19.1	(178)
5-8 yrs	32.2	(202)	38.2	(466)	72.3	(676)	76.2	(805)	44.0	(404)	50.8	(473)
9-10 yrs	14.6	(92)	9.0	(110)	4.2	(39)	2.0	(21)	23.4	(215)	15.7	(146)
>11yrs	33.4	(210)	7.4	(90)	5.5	(51)	4.5	(48)	21.4	(197)	6.9	(64)

*Differences were calculated using Pearson chi square test.

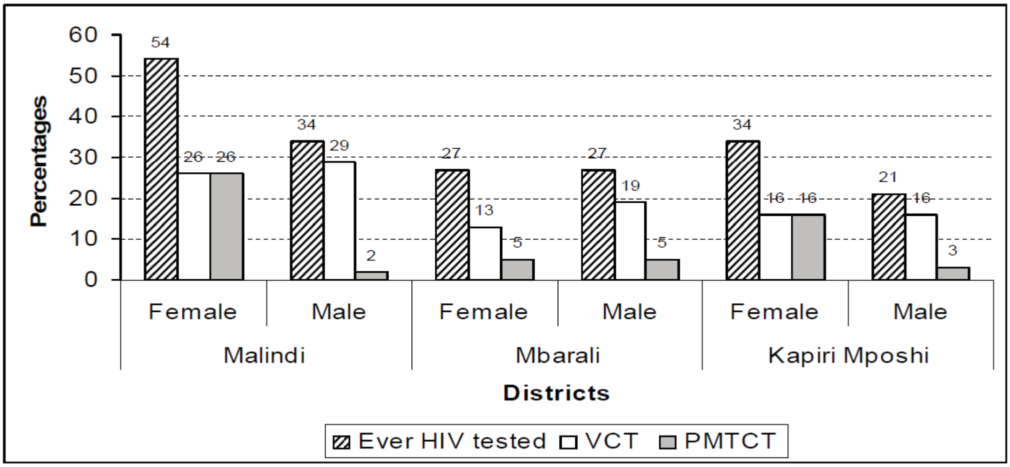

Figure 2 Proportions testing for HIV test and proportions testing VCT and PMTCT.

> *or the strength to prevent being infected as you are told the way forward."* (Female youth, FGD, urban Malindi)

Informants acknowledged that preventive counselling was practised when they underwent VCT.

> *"As for me I was tested at the VCT. There they really counselled me on HIV and on how I can protect my life."* (Male adults, FGD, urban Malindi)

The support dimension

The need for counselling as an important dimension in supporting those already infected with HIV was a point that emerged in both the IDIs and the FGDs across the three districts. Both male and female informants from the three countries expressed the view that sufficient post-test counselling has the potential to reduce worry, fear and blame as illustrated in the quotations below:

> *"Because you may have come there for testing and if you are found (meaning: if you are found to be HIV positive) you will have a lot of worry because in your heart you wonder: 'now I have been found, now what will I do?' Then you have a lot of thoughts, but if you find a person that gives you counselling or ideas on what you will do, it will cool your heart. You will be able to settle a little bit."* (Male 43 years old, rural Malindi)
> *"Because if you have been made aware through counselling, even if you tested positive, there will be no fear, that's why some people declare that they are HIV positive, they had seminars where they were counselled and that is why they have that courage. But if one discloses his status as positive here people will talk about him; some will even deny him drinking water because of fear"* (Male adults, FGD, Mbarali)

The time dimension

The need for sufficient time during counselling in order to convey the messages clearly was experienced as vital in the testing services. More time was expressed as important in order to fully conceptualise and understand the information presented before embarking on the testing so that one would be prepared to receive the test results whatever the outcome might be.

> *"When I say we need education, I mean we need counselling, we need counselling that is offered step by step until we are ready to test."* (Male adults, FGD, Mbarali)
> *"Not that when you enter the facility and after the counselling you are immediately asked if you are ready for the test. No! That also can cause a lack of willingness to test. The counsellor should counsel me and tell me I have the right to go for testing. Therefore, if the time for counselling is increased, I see that as an improvement".* (Female adult, FGD, rural Malindi)

Informants indicated that at the VCT service centres testing is only done after counselling and when the

client is ready. This view is expressed for example by an informant in Kapiri Mposhi who noted:

> "*When you go for VCT, you are counselled and after that, if you are ready to know your status, that's when you are tested.*" (Male youth, FGD, Kapiri Mposhi.)

A female informant from Malindi echoed these views thus:

> "*When you get to the VCT, first of all they offer counselling. You are asked many questions and you continue up to the end, and that is when you are tested.*" (Female 50 years old, rural Malindi)

Challenges experienced with the implementation of the PITC model in HIV testing

The following section draws primarily on the findings from Malindi district.

The study findings indicate that in this district, where the provider initiated testing approach had been scaled up extensively, challenges were faced in the manner in which this new model was implemented.

The threat to counselling

There were differences in the experiences related to HIV counselling at the ANC services in the three districts. In Malindi where the provider initiated opt-out HIV model had been scaled up extensively at the ANC clinics, informants expressed limited or lack of the pre-test counselling and post-test counselling was not present for HIV-negative individuals, as expressed in the following dialogue from Malindi:

> • *Moderator: Do they counsel you before testing for HIV?*
> • *Participant 2: They counsel you only when you are found to be HIV positive, but if you are not HIV positive you just get your results and go.* (Female pregnant, FGD, rural Malindi)

However, informants from Mbarali and Kapiri Mposhi reported the presence of both pre and post test counselling at the ANC clinics during HIV testing as presented below:

> "*We were counselled and it was recommended that we test for HIV before becoming pregnant, but if we are pregnant already we also have to be counselled and tested; say you are found positive, you are to be given drugs to protect the child from infection during delivery.*" (Female pregnant, FGD, Mbarali, Tanzania)

> "*When we went for counselling we were taught. They started teaching us how someone can live positively and strongly. There were papers on the wall, and they showed us what a person looks like before HIV and after. And for us who were pregnant, we were taught how to take care of ourselves and how to protect the unborn baby (if found HIV positive), even during delivery.*" (Female Out- patient adults, FGD, Kapiri Mposhi)

HIV testing as Mandatory

In Malindi our informants reported that the HIV test within PMTCT was no longer voluntary. A common phrase that was used to describe the new testing model was "it is a must", a point noted by both female and male respondents:

> "*I was not tested at a VCT centre, but at that place for women (ANC clinic). Because when you are pregnant, you are tested on many things, but first they must test you for AIDS.*" (Female pregnant 40 years old, urban Malindi)
> "*Here let's say women and men go for (HIV) testing, but a majority of them are women because the woman must be tested when she goes to the clinic.*" (Male 34 years old, urban Malindi)

In Malindi, the study informants explained that little was done at the ANC station to prepare the women for the testing. As a result the women were taken by surprise and panicked when they understood that they were to be tested for HIV.

> "*During the second pregnancy we were not given a choice. It was a must to get tested on HIV and then (after that) on the pregnancy. We were not asked; you enter in the room for HIV testing and then you go for other tests. To tell you the truth, some there got quite scared that day when we were suddenly tested. People panicked a lot. So people were not happy, but it was a must that they do it.*" (Female 35 years old, urban Malindi

The large majority of our informants were disturbed by the lack of choice provision in the new provider initiated approach to the testing services. A few informants, who were from areas where the provider initiated opt-out approach had not been introduced, approved of the new approach and expressed the view that the testing should be mandatory.

> "*It should be made simple such that when you go to the district hospital for any reason, you should be*

tested. They should not wait for voluntary counselling. He/she should be tested and provided with life lengthening drugs. Why don't they offer testing services immediately? In my opinion as soon as a person goes there, there are no reasons for delaying him or her, he/she should be tested and if found positive then he/she should start using medication." (Male, FGD, Mbarali Tanzania)

Despite the fact that the option to opt-out or decline a test is a part of the PITC model, our informants explained that it was in practice not possible to decline HIV testing at the ANC. Opting out implied that further care is declined.

"It was said that according to the rules of the hospital if someone reaches the time of delivery and does not have HIV results she is not received." (Female 35 years old, urban Malindi)
"If you refuse to test they don't examine your stomach. So when it is time for delivery they don't accept you." (Female pregnant, FGD, Urban Malindi)
M: They tell you they are testing you whether you like it or not?
I: Whether you like it or not. If you choose to run away, where will you go? There is no need for running away because you will just have to come back. (Female pregnant 40 years old, urban Malindi)

The expressed burden on women

Many more women than men were tested for HIV as indicated by the population-based survey (Figure 2). This difference was more marked in Malindi district. Our informants described the burden that this places on the women. Another recurring theme was the difficulty of revealing information to partners that also they should go for testing. The burden was partly revealed through statements where our informants expressed the need to involve the health personnel in bringing their spouses to test as shown in the quote below.

"These counsellors should be many to help us because we are wives, and when you ask your husband to go to test himself he stays quiet refusing to talk. He tells you 'you get tested, if you are found to be ok, I am also ok'. He does not go." (Female pregnant, FGD, urban Malindi*)*

This finding was further confirmed by men in the male FGD discussion where men's attitudes towards spouses testing and further clarification on the challenges faced by the women on disclosure is illustrated in the following quote.

'Let me say what men say to their wives, because of their mentality: "Now aren't you the one who has gone for testing and 'you are mine' isn't that right? You have been tested and you are ok? Now what problem do I have?" (Male adult, FGD, rural Malindi)

The challenge that a disproportionately large number of women gets to know their HIV status implies numerous difficulties as the woman will be confronted with the fact that she is the one found to be HIV positive as the following quote reveals

"You know also there are many incidents which have come up because you find that when a woman is heavy (pregnant) it's like the husband forces the wife to go for testing, you see? If anything bad arises (meaning if she is HIV positive) he starts questioning the wife, and asks 'where did it come from?'" (Female pregnant, FGD, rural Malindi)

However in very few cases it was expressed that women too forced their husbands to test as expressed in the following quote

"Mostly women are the ones who force men to go for testing when she is about to be married, or if a wife suspects her husband has another side marriage, she forces the husband to get tested". (Female 33 years old, urban Malindi)

Further there was blame towards the PMTCT program as illustrated in the following quote.

"Now you can see that it is easy to be infected because at this moment when the young women start bearing children and visit the hospital, they have HIV testing done at the PMTCT centre. When they are found to be infected and when the woman returns home, there is a lot of 'stigma', because this is a young woman who has delivered, and it thus looks as if she is the one responsible for bringing the disease home, while it is her husband who came to infect her when she was expecting. You will note that this disease has kind of brought in other very difficult situations, especially in the areas of testing at the PMTCT." (Female adult, FGD, rural Malindi)

There was a strongly expressed need to involve the men or husbands when pregnant women are tested.

> *"For me, I would feel good if there was a way these men can also be forced to go for testing instead of waiting until their wives get pregnant."* (Female pregnant, FGD, rural Malindi)

The consequence of primarily testing women was experienced as severe.

> *"It is important that a pregnant mother is tested along with the husband at the PMTCT, so that they can be counselled and come together for testing. Otherwise you even see many marriages breaking down. Therefore counselling both husband and wife is very important."* (Female adult, FGD, rural Malindi)

Discussion

The population-based survey conducted in the selected study districts in the three countries revealed marked country differences in HIV testing exposures. These differences are primarily linked to differences in the roll-out of the provider-initiated opt-out approach to testing in the PMTCT programs at the ANCs.

Our findings indicate that the manner in which the provider initiated opt-out testing model is being interpreted and implemented has serious unintended negative effects. Firstly a reduced focus on counselling in general was found, and limited or no preventive counselling taking place for persons with HIV negative test results was particularly brought up. Secondly the provider-initiated opt-out strategy was perceived to impede the much-valued consent process inherent in pre-test counselling. The practice was experienced as an obstruction to choice as it was considered impossible to opt-out of the test. Thirdly, the scaling-up of the provider initiated opt-out model through PMTCT was found to result in striking gender differences in knowledge of personal HIV status with consequent burden on women. The survey revealed striking gendered differences between countries related to HIV testing, with a female to male ratio of 1.6 in Malindi and Kapiri Mposhi districts, the districts that presented a high exposure in HIV testing as part of PMTCT. No gender difference appeared in Mbarali district where the exposure to PMTCT was relatively low. The study revealed that the strategy was perceived to add an unreasonable burden on women who increasingly have to communicate the test results to their partners when they are pregnant as well as recruit them to go for testing.

The provider-initiated opt-out testing model has been described as successful in achieving higher numbers of individuals to test [21,36-39]. Our results indicate that substantially more women seem to have been tested in areas where this model had been scaled up. The provider initiated testing and counselling guidelines, recommend that post test counselling is performed after an HIV test [15]. On implementation, our results revealed however, that there was reduced focus on counselling during HIV testing at the ANC settings. The informants expressed the view that no or limited preventive counselling took place for persons with negative test results within the new approach, as summarised in Figure 3. The expansion of testing, which has been closely linked to rapidly-increasing financial support for treatment, has led many countries to shift their focus towards treatment with less focus on HIV prevention [40]. Post-test counselling for persons with a negative test result offers vital opportunities for prevention. It gives health care providers the opportunity to assess the degree of risk related to the client's lifestyle and enables him/her to define and communicate potential behaviour change and ways to sustain this behaviour change [2,41].

Despite the expressed importance of post-test counselling for prevention by our informants, the findings suggest that this was often lacking for people with negative test results. This is in our view an alarming development, and implies increasing numbers of missed opportunities for prevention, as some recent studies have indicated. A study from South Africa showed that pregnant women had four times the transmission risk of other women [42] indicating the importance of reaching this particular category with messages focusing on prevention of horizontal transmission. The increased sexual risk-taking that followed a negative test result in a cohort conducted in Zimbabwe [43] moreover indicates the potential dangers of leaving out individuals who have tested HIV negative without proper preventive counselling. In line with this argument a recent population-based open cohort study related to behaviour change associated with VCT showed substantial risk reduction, particularly among women, regardless of their HIV status [44].

The finding of limited pre-test counselling in our study is consistent with other publications that have raised the issue of neglected counselling within this testing model [10,16,22]. Counselling is one of the three Cs' (consent, confidentiality and counselling) perceived as core in HIV testing [45]. Given that counsellors are properly trained to assist in the process of attaining consent, pre-test counselling plays a fundamental role. It promotes the individual's autonomy by offering an opportunity to make an informed decision that is critical for HIV testing [2,41]. Individual autonomy is an important element of 'responsiveness'; a fundamental goal of health systems [46,47] and critically important in HIV testing [48].The implementation of the provider initiated opt-out testing model perceived as mandatory as reflected in the formulation " it is a must" by our informants, not only contradicts the current global

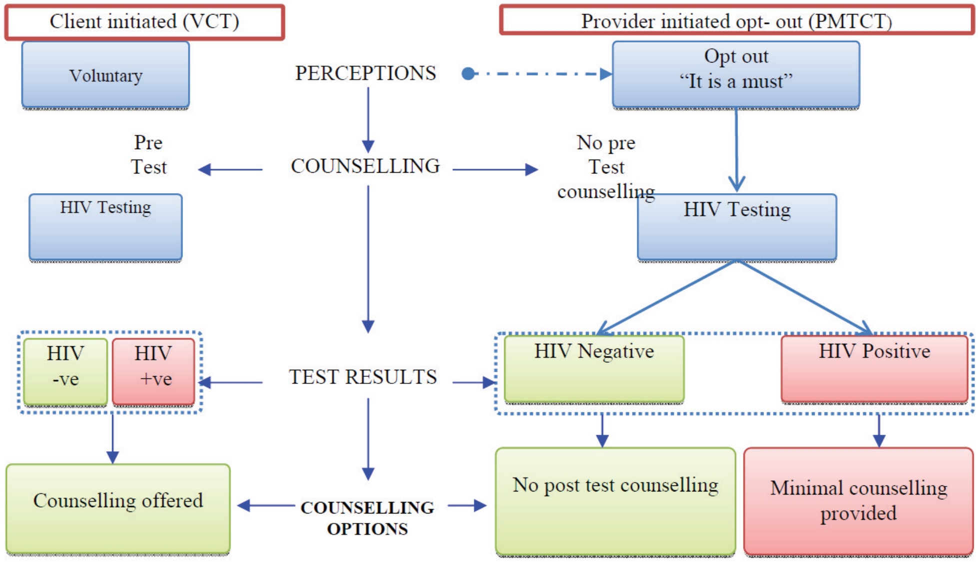

Figure 3 The client initiated (VCT) and Provider initiated Opt- out (PMTCT) HIV testing process as experienced by our informants.

guidelines, which emphasize the rights of clients to decline or opt-out of HIV testing when offered [15,45] but also national guidelines on HIV testing [5]. Similar concerns have been raised in Botswana. Even though high proportions were in favour of the opt-out testing model, a majority (68%) felt that it was not possible to refuse a test [49,50]. Studies and reports from Kenya and Uganda reveal that women seeking maternal care were not provided with the opportunity to give informed consent, nor counselled prior to or after testing [51-53].

Such scenarios have created an important discussion related to the classical dilemmas on how to balance public health benefits with the individual good. One major argument has been that the benefit of mandatory testing in preserving human life (as the HIV positive individual gets the opportunity to receive treatment) outweighs arguments related to human rights and autonomy [54]. This argument has gained grounds in the context of prevention of vertical transmission. It is in our view important to be aware of what is lost in the process when PITC models are not implemented with caution and with fundamental respect for the key principles on which it is based. The provider initiated opt-out testing has thus substantially increased access to prevention of vertical transmission of the infection [21,55], which was one of the main intentions behind the changes in testing approaches [2,56] in ANC settings. While there is increasing demonstration of success related to the provider initiated opt-out model in terms of testing coverage, we argue that the ways in which the PITC model is implemented calls for a consideration of aspects beyond the proportions exposed to testing and the proportions offered treatment.

Our findings on HIV testing exposure have furthermore indicated unforeseen inequity challenges emerging from the fact that with scaling up of the provider initiated opt-out testing in PMTCT programs; women are exposed to testing to a much larger extent than men. Previous studies have demonstrated that the mere act of being tested for HIV can be enough to subject a woman to domestic violence [51]. The wide recognition of potential adverse effects of disclosure to husbands adds seriously to the burden of knowing ones HIV status [16,57]. It has been demonstrated that women with HIV positive test results may be confronted with severe violence that includes physical and verbal abuse also from staff at the health facilities [51]. In Kenya such challenges have led to the development of a tribunal that aims to handle legal HIV-related issues including discrimination against people living with HIV [58]. With the adverse, negative effects upon women that have been documented upon receiving an HIV test result, the enormous numbers presently being tested through the opt-out approaches in PMTCT programs raise serious ethical concerns. We argue that implementation of this testing approach should include partners to avoid venturing into a dangerous field which could lead

women to choose not to disclose their status. A review carried out on rates barriers and outcomes of disclosure indicated that higher rates of women choose not to disclose (up to 86%). However it must be noted that majority of those who actually disclosed their HIV status reported supportive reactions from their partners [59]

In a context where there is the challenge of under-utilization of skilled attendance at birth services [60], confidence and trust are values at stake in the maternal health services. Testing pregnant women for HIV without their consent and without counselling to prepare them with a minimum of knowledge on how to live with either a HIV negative or a HIV positive status, can further diminish their confidence in the health providers and the health system, and can drive them away from vital maternity-related services. The weakened image of the health providers is part of a tendency also influenced by new public health management approaches [23]. The challenges faced by health providers in implementing the new testing model may certainly to some extent, be attributed to the weak health systems in the region [61]. Sub Saharan Africa suffers a high burden of disease and the ratios of health workers per population are extremely low [62]. In contexts where the health system is grappling with limited resources, poor provider behaviour to clients has also been outlined among the many challenges facing HIV service delivery [61].

Unlike a VCT setting where very few seek testing services, the ANC environments are characterised by large numbers of the women as the main purpose is to test for mother and child related issues and not to test for HIV. Within such a scenario, health care providers may be overwhelmed by the high numbers of clients they receive for testing, hence influencing the manner in which the testing services are offered. This is an important part of the context in which health care providers work and coming mothers receive their HIV testing results, including little time for serious consideration of opt out processes of extended educational sessions. We thus argue that the 'blame' cannot primarily be placed on the health care workers. Our data e.g. reveals that pre and post test counselling are sufficiently observed at VCT. Strengthening the health system therefore seems to be vital if PITC implementation is to take place in the manner it was intended to. In this regard, involving lay counsellors in the testing and counselling services in these busy clinics as done in Botswana could be considered to reduce the burden on the health workers [39].

We argue that the challenges faced when implementing the provider initiated opt-out approach to HIV testing in many low income contexts, call for a renewed resource and rights-based momentum. Accountability for reasonableness, an ethically-based framework for fair decision-making and the fair priority-setting employed in the larger project of which this study is a part, draws our attention to the importance of trust in health systems [63]. The framework holds that securing trust in health systems will require approaches and arrangements that in a fundamental sense promote fairness and equity in a manner that respects human rights. From such a perspective implementing PITC for HIV in ways that compromises the right to obtain preventive information, and, for pregnant women, the right to opt-out or bypass consultation with their spouses are highly problematic. Trust between client and provider is critically important in an HIV context where vital but sensitive messages are to be communicated [18]. However, this cannot be secured through approaches implemented in a manner that threaten what has been considered as key in HIV testing; namely the possibility of receiving preventive information. The concern related to the extra burdens placed on women in vulnerable states of pregnancy adds to this scenario. We thus argue that HIV testing service delivery needs to be strengthened so that the trust and momentum that has been gained through the client-initiated services is retained. Respect for individual autonomy in HIV testing is essential as seen from the fundamental responsiveness goal of health systems [46,47].

Adopting a concurrent triangulation mixed methods design in this study reduces the potential weaknesses of using a single method. This design was chosen in order to capture both population testing exposure levels as well as people's experiences with the testing strategies. The data that was generated offered an opportunity to make inter-country comparisons, and in particular to compare setting differences with regard to levels of HIV testing exposure. The data collection tools in the three districts were similar in terms of indicator questions (the survey) and guidelines for the qualitative data collection. The additional qualitative component that was added in one of the districts (Malindi) elicited further information regarding experiences and perceptions of the provider initiated opt-out testing model. This added component strengthened the data as the additional FGDs and IDIs also enabled us to capture the perceptions and personal experiences of our informants at the community level, in addition to the facility-based information collected from all three districts.

As this additional study was carried out in 2009, a time when the PITC model had been implemented in most settings, it enabled us to capture experiences and perceptions of this new HIV testing model. However, conducting this study only in one district (Malindi) puts restrictions on the possibility for inter-country comparisons with regard to experiences and perceptions related to the provider initiated opt-out HIV testing models. Recruiting the respondents in this additional study

(Malindi) from the community did not always reap direct experiences with the ANC testing; hence, further studies where respondents are recruited at the ANC settings could be considered. Our findings also suggest that the PITC model could have been practiced in a manner that seemed acceptable in the other two districts. This finding concurs with a recent study from Mbale district in Uganda [64]. Nevertheless, it is likely that, at the time, the data was collected in these settings (Kapiri Mposhi and Mbarali) the PITC opt-out model had not been scaled up extensively. We have reason to believe that the findings of this study are applicable in many other settings as similar concerns are raised in recent studies conducted in Kenya and Uganda, and concerns documented from Tanzania, on practices of testing pregnant women without their consent or proper counselling [50,52,53,65].

Conclusion

The manner in which the new provider initiated opt-out HIV testing model is being implemented was found to have resulted in high neglect of pre- and post test counselling. This could hamper the inherent preventive potential in HIV testing contexts. Moreover, the lack of counselling was found to hinder people's ability to decline testing, hence raising serious ethical concerns. Furthermore, the new testing model appeared to add an unreasonable burden to pregnant women, in that the approach is implemented on a large scale primarily among women in the PMTCT programs, who are made responsible to recruit their spouses to go for a test. Further research is needed to: explore challenges from the perspective of health care workers and how to strengthen the health systems and in particular the ante natal clinics, so as to effectively implement the new testing model, assess how the opt-out testing approaches are implemented in other high HIV prevalence settings and to explore further how 'Accountability for reasonableness' or other rights-based frameworks can be drawn upon in ways that ensure that new strategies attempting to improve access HIV services are implemented in a manner that retains trust and a minimum of the patients' rights. Our findings indicates that there is an urgent need to reconsider the manner in which the opt-out approach to HIV testing is presently being implemented in order to protect each client's autonomy, to protect the right to access HIV prevention, and to protect pregnant women from an unreasonable additional burden.

Note

[1] *s/he: in Swahili there is no separate word for she and he*

Acknowledgements

This study was made possible through funding and support from the EU-funded REACT project and the Norwegian government's Quota program which provided financial support to conduct further research in Malindi. The REACT (REsponse to ACcountable priority setting for Trust in health systems) project is a specific targeted research and innovation project whose full title is "Strengthening of fairness and accountability in priority setting for improving equity and access to quality health care at district level in Tanzania, Kenya and Zambia." The authors are grateful to all the study participants in the three countries who provided their time for the interviews and the team from the National Central Bureau of Statistics in all the districts that assisted in collecting the data. Special thanks go to Jens Byskov, who was central in conceptualizing the REACT project, and who ensured that both the quantitative and qualitative data were collected in all three districts. He also gave input to the manuscript. Charles Michelo was central in the development of the survey tools, supervised the acquisition of the qualitative data from Kapiri Mposhi district and gave input to the manuscript. Paul Bloch offered valuable input to the manuscript. We also wish to thank Mary Tuba for her involvement in drafting the qualitative guides for the additional study in Malindi as well as taking part in the data collection in Zambia, and Dr Yeri Kombe for coordinating the REACT activities in Kenya and for ensuring that all translations of the qualitative study tools and data collected in Malindi were thoroughly done. We are very grateful to the REACT team members from all the countries for their involvement in planning and data collection (Lillian Nyandieka, James Muttunga and Joseph Mutai from the Centre for Public Health Research, Kenya Medical Research Institute; Senkoro Kesheni from the National Institute for Medical Research, Tanzania; and Oliver Mweemba from the Department of Community Medicine, School of Medicine, University of Zambia).

Author details

[1]Centre for Public Health Research, Kenya Medical Research Institute, Nairobi, Kenya. [2]Centre for International Health, Faculty of Medicine and Dentistry, University of Bergen, Bergen, Norway. [3]Departments of Public Health and Primary Health Care, Faculty of Medicine and Dentistry, University of Bergen, Bergen, Norway. [4]National Institute for Medical Research, Dar Es Salaam, Tanzania. [5]Institute of Anthropology, Gender and African studies, University of Nairobi, Nairobi, Kenya.

Authors' contributions

MKN was involved in conceptualising the objective of this paper, developing the interview guides for the second phase of qualitative interviews carried out in Malindi, conducting the interviews, analysing the quantitative and qualitative data, interpreting the findings and writing the manuscript. AB was involved in: developing REACT study tools, developing the interview guides for the additional study in Malindi, analysing and interpreting the qualitative findings and in extensive revision of the manuscript. EHS was involved in developing the REACT qualitative study tools, collecting the qualitative data in Mbarali, analysing the qualitative data and revising the manuscript. IKN coordinated the REACT qualitative studies in Kenya, reviewed the study guides for the second phase of qualitative interviews, was involved in analysing and interpreting the qualitative data and revised the manuscript. KF was involved in conceptualizing the REACT project as well as the objective of the present manuscript, he took part in the development of the design and data collection tools for the population-based survey, analysing the quantitative part of the study and revising the manuscript extensively. All authors read and approved the final manuscript.

Competing interests

The authors declare that they have no competing interests.

Received: 1 May 2010 Accepted: 21 April 2011 Published: 21 April 2011

References

1. Joint publication of IPPF South Asia Regional Office and UNFPA: **Integrating HIV voluntary counselling and testing services into reproductive health settings: Stepwise guidelines for programme planners, managers and service providers.** 2004.

2. WHO: **The Right to Know: New Approaches to HIV Testing and Counselling.** 2003.
3. Denison JA, O'Reilly KR, Schmid GP, Kennedy CE, Sweat MD: **HIV voluntary counseling and testing and behavioral risk reduction in developing countries: A meta analysis, 1990-2005.** *AIDS Behav* 2007, **12:** 363-373.
4. Voluntary, HIV-1, Counseling, and, Testing, Efficacy, Study, Group: **Efficacy of voluntary HIV-1 counseling and testing in individuals and couples in Kenya, Tanzania and Trinidad: a randomised trial.** *Lancet* 2000, **356**:103-112.
5. National AIDS and STI Control Programme, Ministry of Public health and sanitation Kenya: **National guidelines for HIV testing and counselling in Kenya.** Nairobi; 2008.
6. Babalola S: **Readiness for HIV testing among young people in northern Nigeria: The roles of social norm and perceived stigma.** *AIDS Behav* 2006, **11(10)**:759-769.
7. Fylkesnes K, Haworth A, Rosenvard C, Kwapa P: **HIV counselling and testing: Overemphasizing high acceptance rates a threat to confidentiality and the right not to know.** *AIDS* 1999, **13(17)**:2469-2474.
8. Irungu T, Varkey P, Cha S, Patterson J: **HIV voluntary counselling and testing in Nakuru, Kenya: Findings from a community survey.** *British HIV Association HIV Medicine* 2008, **9(2)**:111-117.
9. Fylkesnes K, Siziya S: **A randomized trial on acceptability of voluntary HIV counselling and testing.** *Tropical Medicine and International Health* 2004, **9(5)**:566-572.
10. Obermeyer MO, Osborn M: **The utilization of testing and counselling for HIV: A review of the social and behavioral evidence.** *Am J Public Health* 2007, **97(10)**:1762-1774.
11. Angoti N, Bula A, Gaydosh L, Kimchi EZ, Thornton RL, Yeatman SE: **Increasing the acceptability of HIV counselling and testing with three C's: Convenience, confidentiality and credibility.** *Social Science and Medicine* 2009, **68(12)**:2263-2270.
12. Beteganya MH, Abdulwadud OA, Kiene SM: **Home based HIV voluntary counselling and testing in developing countries.** *Cochrane database of systematic Reviews* 2007, **4.**
13. Matovu JKB, Makumbi EF: **Expanding access to voluntary HIV counselling and testing in sub-Saharan Africa: alternative approaches for improving uptake, 2001-2007.** *Tropical Medicine and International Health* 2007, **12(11)**:1315-1322.
14. Matovu JK, Kigozi G, Nalugoda F, Wabwire-Mangen F, Gray RH: **The Rakai Project counselling programme experience.** *Tropical Medicine and International Health* 2002, **7(12)**:1064-1067.
15. WHO, UNAIDS: **Guidance on provider-initiated HIV testing and counselling in health facilities.** Geneva, Switzerland; 2007.
16. Maman S, King E: **Changes in HIV testing policies and the Implications for Women.** *Journal of Midwifery & Women's Health* 2008, **53(3)**:195-201.
17. Gruskin S, Ahmed S, Ferguson L: **Provider initiated HIV testing and counselling in health facilities- what does this mean for the health and human rights of pregnant women?** *Developing World Bioethics* 2007, **8(1)**:23-32.
18. Gilson L: **Trust and the development of health care as a social institution.** *Social Science and Medicine* 2003, **56**:1453-1468.
19. April MM, Walensky RP, Chang Y, Pitt J, Freedberg KA, Losina E, Paltiel DA, Wood R: **HIV Testing Rates and Outcome in a South African Community, 2001-2006: Implications for expanded screening policies.** *Journal of Acquired Immne Deficiency Syndromes* 2009, **51(3)**:310-316.
20. Ministry of Health, National Aids and STI Control Programme: **AIDS in Kenya trends, Interventions and Impact.** Nairobi: Ministry of Health, National AIDS and STI Control Programme;, 7 2005, 104.
21. Creek TL, Ntumy R, Seipone K, Smith M, Mogodi M, Smit M, Legwaila K, Molokwane I, Tebele G, Mazhani L, Shaffer N, Kilmarx PH: **Successful introduction of routine opt-out HIV testing in antenatal care in Botswana.** *J Acquir Immune Defic Syndr* 2007, **45(1)**:102-107.
22. Yeatman SE: **Ethical and public health considerations in HIV counselling and testing: Policy implications.** *Studies in Family Planning* 2007, **38(4)**:271-278.
23. Branson BM, Handsfield HH, Lampe MA, Janssen RS, Taylor AW, Lyss SB, Clark JE: **Revised recommendations for HIV testing of adults, adolescents, and pregnant women in health-care settings.** *Journal of the National Medical Association* 2008, **100(1)**:131-147.
24. Byskov J, Bloch PAB, Hurtig AK, Fylkesnes K, Kamuzora P, Kombe Y, Kvåle G, Marchal B, Martin DM, Michel C, Ndawi B, Ngulube TJ, Nyamongo I, Olsen1 ØE, Onyango-Ouma W, Sandøy IF, Shayo EH, Silwamba G, Songstad NG, Tuba M: **Accountable priority setting for trust in health systems - the need for research into a new approach for strengthening sustainable health action in developing countries.** *BMC Health Research Policy and Systems* 2009, **7(23)**:24, October 2009.
25. Creswell JW, Plano Clark VJ, Hanson WE: *A handbook of mixed methods in social and behavioural research* California: Sage publications Inc; 2003.
26. **Epidemiological fact sheets on HIV and AIDS, Kenya.** [http://www.who.int/globalatlas/predefinedReports/EFS2008/full/EFS2008_KE.pdf].
27. **Epidemiological fact sheet on HIV and AIDS, united republic of Tanzania.** [http://www.unaids.org/en/CountryResponses/Countries/united_republic_of_tanzania.asp].
28. **Epidemiological fact sheet on HIV and AIDS, Zambia.** [http://www.unaids.org/en/CountryResponses/Countries/zambia.asp].
29. Ministry of Finance and planning Kenya: **Malindi district development plan 2002-2008.** Nairobi: Republic of Kenya; 2002.
30. Tanzania Commission for AIDS, Zanzibar AIDS Commission, National Bureau of Statistics, Inc: MI: **Tanzania HIV/AIDS and Malaria Indicator Survey 2007-08 In Preliminary Report.** 2008, 1-58.
31. Fylkesnes K, Ndhlovu Z, Kasumba K, Musonda RM, Sichone M: **Studying dynamics of the HIV epidemic: population-based data compared with sentinel surveillance in Zambia.** *AIDS* 1998, **12(1227-1234)**.
32. Michelo C, Sandøy I, Dzekedzeke K, Siziya S, Fylkesnes K: **Steep HIV prevalence declines among young people in selected Zambian communities (1995-2003).** *BMC Public Health* 2006, **6(279)**.
33. Ministry of Health Kenya: **Malindi district annual health report 2004.** 2005.
34. Lacey A, Luff D: *Trent focus for research and development in primary Health Care: An introduction to qualitative data analysis* Trent Focus Group; 2001.
35. Pope C, Ziebland S, Mays N: **Qualitative research in health care: Analysing qualitative data.** *BMJ* 2000, **320**:114-116.
36. Moses A, Zimba C, Kamanga E, Nkhoma J, Maida A, Martinson F, Mofolo I, Joaki G, Muita J, Spensley A, Hoffman I, Van der Horst CM, UNC Project Call to Action Program: **Prevention of mother-to-child transmission: program changes and the effect on uptake of the HIVNET 012 regimen in Malawi.** *Aids* 2008, **22(1)**:83-87.
37. Nakanjako D, Kamya M, Kyabayinze D, Mayanja-Kizza H, Freers J, Whalen CEK: **Acceptance of routine testing for HIV among adult patients at the medical emergency unit at a national referral hospital in Kampala, Uganda.** *AIDS Behav* 2007, **11**:753-758.
38. Perez F, Zvandaziva C, Engelsmann B, Dabis F: **Acceptability of routine HIV testing ("opt-out") in antenatal services in two rural districts of Zimbabwe.** *Journal of Acquired Immune Deficiency Syndromes* 2006, **41(4)**:514-520.
39. Steen TW, Seipone K, Anderson MG, Kejelepula M, Keapoletswe K, Moffat HJ: **Two and a Half years of routine HIV testing in Botswana.** *AIDS* 2007, **44(4)**:484-488.
40. **Global HIV prevention: The access and funding gap.** [http://globalhivprevention.org/pdfs/pwg_access_factsheet_6_07.pdf].
41. Chippindale S, French L: **HIV counselling and the psychosocial management of patients with HIV/AIDS.** *BMJ* 2001, **322**:1533-1535.
42. Moodleya D, Esterhuizenb TM, Pathera T, Chettya V, Ngaleka L: **High HIV incidence during pregnancy: compelling reason for repeat HIV testing.** *AIDS* 2009, **23**:1255-1259.
43. Sherr L, Lopman B, Kakowa M, Dube S, Chawira G, Nyamukapa C, Oberzaucher N, Cremin I, Gregson S: **Voluntary counselling and testing: uptake, impact on sexual behaviour and HIV incidence in a rural Zimbabwean cohort.** *AIDS* 2007, **21**:851-860.
44. Cremin I, Nyamukapa C, Sherr L, Hallett TB, Chawira G, Cauchemez S, Lopman B, Garnett GPSG: **Patterns of Self-reported Behaviour Change Associated with Receiving Voluntary Counselling and Testing in a Longitudinal Study from Manicaland, Zimbabwe.** *AIDS Behav* 2009, **10.**
45. Joint United Nations Programme on HIV/AIDS (UNAIDS): **UNAIDS/WHO Policy Statement on HIV testing.** 2004.
46. Darby C, Valentine N, Murray CL, De Silva A: **World Health Organization: Strategy on measuring responsiveness.** In *GPE Discussion Paper series. Volume 23.* WHO; 2000.
47. Murray CJL, Evans DB: *Health systems performance assessment: Debates, methods and empiricism* Geneva: World Health Organization; 2003.
48. Njeru MK, Blystad A, Nyamongo IK, Fylkesnes K: **A critical assessment of the WHO responsiveness tool: lessons from voluntary HIV testing and counselling services in Kenya.** *BMC Health Service Research* 2009, **9(243)**.

49. Jaffe HW: **Increasing Knowledge of HIV Infection Status through Opt-Out Testing.** *Journal of Bioethical Inquiry* 2009, **6**:229-233.
50. Weiser SD, Heisler M, Leiter K, Percy-de Korte F, Tlou S, DeMonner S, Phaladze N, Bangsberg DR, Iacopino V: **Routine HIV testing in Botswana: a population-based study on attitudes, practices, and human rights concerns.** *PLoS Med* 2006, **3(7)**:1013-1022.
51. The Center for Reproductive Rights, Federation of Women Lawyers–Kenya: **At Risk: Rights Violations of HIV-Positive Women in Kenyan Health Facilities.** United States; 2008.
52. Awiti Ujiji O, Rubenson B, Ilako F, Marrone G, Wamalwa D, Wangalwa G, AM E: **Is 'Opt-Out HIV Testing' a Real Option among Pregnant Women in Rural Districts in Kenya?** *BMC Public Health 8* **11(1)**:151, 2011, 11(151).
53. Larsson EC, Thorson A, Pariyo G, Conrad P, Arinaitwe M, Kemigisa M, Eriksen J, Tomson G, Ekström AM: **Opt-out HIV testing during antenatal care: experiences of pregnant women in rural Uganda.** *Health Policy Plan* 2011.
54. Clark AP: **Mother- to- child transmission of HIV in Botswana: An ethical perspective on Mandatory testing.** *Dev World Bioeth* 2006, **6(1)**:1-12.
55. Chandisarewa W, Stranix-Chibanda L, Chirapa E, Miller A, Simoyi M, Mahomva A, Maldonado Y, Shetty AK: **Routine offer of antenatal HIV testing ("opt-out" approach) to prevent mother-to-child transmission of HIV in urban Zimbabwe.** *Bull World Health Organ* 2007, **85(11)**:843-850.
56. WHO, UNAIDS, UNICEF: **Towards Universal access: Scaling up priority HIV/AIDS interventions in the health sector.** Geneva, Switzerland; 2008.
57. Pool R, Nyanzi SI, Whitworth JAG: **Attitudes to voluntary counselling and testing for HIV among pregnant women in rural south-west Uganda.** *AIDS CARE* 2001, **13(5)**:605-615.
58. PlusNews Global HIV/AIDS news and analysis: **KENYA: Special tribunal for HIV-related issues.** *PlusNews Volume 2010* Nairobi; 2010.
59. Medley A, Garcia-Moreno G, McGill S, Maman S: **Rates, barriers and outcomes of HIV serostatus disclosure among women in developing countries: implications for prevention of mother-to-child transmission programmes.** *Bull World Health Organ* 2004, **82(4)**:299-304.
60. **Skilled attendant at birth–2007 updates.** [http://74.125.77.132/search?q=cache:NssBGdCa9fQJ:whqlibdoc.who.int/hq/2007/WHO_RHR_07.16_eng.pdf+Skilled+attendant+at+birth%E2%80%942007+updates&cd=5&hl=en&ct=clnk].
61. Travis p, Bennett S, Haines A, Pang T, Bhutta Z, Hyder AA, Pielemeier NR, Mills A, Evans T: **Overcoming health-systems constraints to achieve the Millennium Development Goals.** *Lancet* 2004, **364**:900-906.
62. The Joint learning initiative Africa working group: **The Health Workforce in Africa Challenges and Prospects.** 2006.
63. Martin D, Singer P: **A strategy to improve priority setting in health care institutions.** *Health Care Analysis* 2003, **11(1)**:59-68.
64. Byamugisha R, Tumwine JK, Ndeezi G, Karamagi CAS, Tylleskär T: **Attitudes to routine HIV counselling and testing, and knowledge about prevention of mother to child transmission of HIV in eastern Uganda: a cross-sectional survey among antenatal attendees.** *Journal of International AIDS society* 2010, **13(52)**.
65. The guardian reporter: **Involuntary HIV tests of pregnant women faulted.** *The Guardian* Dar es salam; 2010.

Pre-publication history

The pre-publication history for this paper can be accessed here:
http://www.biomedcentral.com/1472-6963/11/87/prepub

doi:10.1186/1472-6963-11-87
Cite this article as: Njeru et al.: Practicing provider-initiated HIV testing in high prevalence settings: consent concerns and missed preventive opportunities. *BMC Health Services Research* 2011 **11**:87.

Submit your next manuscript to BioMed Central and take full advantage of:

- Convenient online submission
- Thorough peer review
- No space constraints or color figure charges
- Immediate publication on acceptance
- Inclusion in PubMed, CAS, Scopus and Google Scholar
- Research which is freely available for redistribution

Submit your manuscript at
www.biomedcentral.com/submit

BioMed Central

III

BMC Health Services Research

BioMed Central

Research article

Open Access

A critical assessment of the WHO responsiveness tool: lessons from voluntary HIV testing and counselling services in Kenya

Mercy K Njeru*[1,2], Astrid Blystad[4,2], Isaac K Nyamongo[3] and Knut Fylkesnes[2]

Address: [1]Centre for Public Health research, Kenya Medical Research Institute, Nairobi, Kenya, [2]Centre for International Health, Faculty of Medicine and Dentistry, University of Bergen, Bergen, Norway, [3]Institute of Anthropology, Gender and African studies, University of Nairobi, Nairobi, Kenya and [4]Department of Public Health and Primary Health Care, Faculty of Medicine and Dentistry, University of Bergen, Bergen, Norway

Email: Mercy K Njeru* - mercy.njeru@cih.uib.no; Astrid Blystad - astrid.blystad@isf.uib.no; Isaac K Nyamongo - inyamongo@uonbi.ac.ke; Knut Fylkesnes - knut.fylkesnes@cih.uib.no

* Corresponding author

Published: 22 December 2009

BMC Health Services Research 2009, **9**:243 doi:10.1186/1472-6963-9-243

Received: 19 December 2008
Accepted: 22 December 2009

This article is available from: http://www.biomedcentral.com/1472-6963/9/243

© 2009 Njeru et al; licensee BioMed Central Ltd.
This is an Open Access article distributed under the terms of the Creative Commons Attribution License (http://creativecommons.org/licenses/by/2.0), which permits unrestricted use, distribution, and reproduction in any medium, provided the original work is properly cited.

Abstract

Background: Health, fair financing and responsiveness to the user's needs and expectations are seen as the essential objectives of health systems. Efforts have been made to conceptualise and measure responsiveness as a basis for evaluating the non-health aspects of health systems performance. This study assesses the applicability of the responsiveness tool developed by WHO when applied in the context of voluntary HIV counselling and testing services (VCT) at a district level in Kenya.

Methods: A mixed method study was conducted employing a combination of quantitative and qualitative research methods concurrently. The questionnaire proposed by WHO was administered to 328 VCT users and 36 VCT counsellors (health providers). In addition to the questionnaire, qualitative interviews were carried out among a total of 300 participants. Observational field notes were also written.

Results: A majority of the health providers and users indicated that the responsiveness elements were very important, e.g. confidentiality and autonomy were regarded by most users and health providers as very important and were also reported as being highly observed in the VCT room. However, the qualitative findings revealed other important aspects related to confidentiality, autonomy and other responsiveness elements that were not captured by the WHO tool. Striking examples were inappropriate location of the VCT centre, limited information provided, language problems, and concern about the quality of counselling.

Conclusion: The results indicate that the WHO developed responsiveness elements are relevant and important in measuring the performance of voluntary HIV counselling and testing. However, the tool needs substantial revision in order to capture other important dimensions or perspectives. The findings also confirm the importance of careful assessment and recognition of locally specific aspects when conducting comparative studies on responsiveness of HIV testing services.

Background

The World Health Organization (WHO) advises that evaluation of performance of any health action should be centred on the 3 fundamental goals of a health system: improving health, enhancing responsiveness to the user's expectations, and assuring fairness of any financial contribution [1-4]. In this context, patient surveys aimed at generating knowledge to make health services more responsive to the user's needs and expectations are becoming increasingly important [5]. Responsiveness in the context of health systems has been defined as "the outcome that can be achieved when health institutions and institutional relationships are designed in such a way that they are cognisant of and respond appropriately to the universally legitimate expectations of individual" [6,7]. This very broad definition can be viewed from 2 perspectives. Firstly, the user of the health-care system is seen as a consumer where greater responsiveness becomes a means of attracting consumers. Secondly, responsiveness is seen safeguarding the rights of patients to adequate and timely care [6].

A responsive health system needs to contribute to the enhancement of health by creating a conducive environment that increases the likelihood of individuals seeking care earlier, increases the openness in their interactions with the health-care providers, and improves their assimilation of health information [7]. Responsive health systems can contribute by reducing barriers to the use of health services, making responsiveness a strong determinant of trust in them [8]. Two major components have been defined by WHO in attempts to measure responsiveness, namely respect for persons, which captures aspects of individual interaction with the health system, and client orientation, which includes several aspects of consumer satisfaction [1,3,6]. WHO also developed 7 elements as the central elements needed to measure the responsiveness of a health system and consequently validated a questionnaire that was used to measure levels of responsiveness in surveys [3,9]. This tool has since been employed in several studies [10-12]. Responsiveness is one of the central parameters in health-care performance [4], making surveys measuring responsiveness instrumental in providing evidence that can guide resource allocation and management strategies [6].

The comparability of different health systems using a single tool to measure performance has been questioned [13]. Studies conducted on health-related responsiveness in Turkey and Taiwan for example, found that recognition of the value of culturally specific aspects, demographic structures and country specific factors should be taken into account when assessing responsiveness. Their advice is that responsiveness ranking countries should be done on the basis of tools that take into account the views of their own citizens [10,12].

The responsiveness of health systems is of particular importance in the context of HIV, due to the heavy stigma associated with this infection. In these contexts, highly responsive health systems are of vital importance for trust and acceptability. A case of concern is the low uptake of voluntary HIV counselling and testing (VCT). VCT has been defined as a confidential process by which people undergo individual counselling to enable them to make an informed choice about being tested voluntarily for HIV [14], and to consider their own HIV related risk. These services are pivotal in meeting the commitment of "universal access to prevention, treatment and care" in an HIV context [14-16]. Despite the importance given to VCT, studies from sub-Saharan Africa have shown that while readiness for VCT is high, utilization is still low, even in places where services are readily available [17,18]. Uptake is particularly low when offered from centres located in general health clinics. Limited trust has been suggested as part of the cause of poor acceptability (the difference between intention and actual use) of VCT [17,19-22]. In a context of stigma and high sensitivity, responsiveness emerges as a highly relevant concept in evaluating HIV prevention and care programs.

About 8% of Kenyan adults (15-49 years) are estimated to have HIV [23]. A wide range of preventive, care, support and treatment interventions have been instituted over the past 20 years to meet the epidemic. Among them is relatively rapid scaling up in HIV counselling and testing services [24]. However, little is known about how these VCT services respond to the expectations and needs of the people. In this study we investigated the applicability and relevance of the WHO developed elements (dignity, autonomy, confidentiality, prompt attention, quality of basic amenities and choice of providers) proposed to measure responsiveness, within the context of VCT services as a district level in Kenya.

Methods

Study design

The study was initially intended to use quantitative methods, but the pilot study indicated the need for mixed methods so as to explore other elements of responsiveness that were not captured by the closed questions within the quantitative survey. Mixing quantitative and qualitative methods can be used to add insights likely to be missed when only a single method is used and to increase generalizability to the results [25]. A concurrent nested study design [26] was adapted to enable the researchers to gain broader perspectives of responsiveness, by adding a qualitative open-ended question to the quantitative questionnaire. This type of design enables the collection of quantitative and qualitative data during one phase and it involves interviewing the same persons using different techniques, which in turn could help to identify measurement and methodological problems [26,27].

The study was conducted from October to November 2007 in Malindi district, in Kenya where the EU-funded five year intervention study "REsponse to ACcountable priority setting for Trust in health systems" (REACT) is being conducted [28]. The intervention being applied is an explicit ethical framework for legitimate and fair priority setting, accountability for reasonableness (AFR). The values being focused in the evaluation of the intervention are quality, equity and trust [28]. In addition to Kenya, the REACT research project is ongoing in Tanzania and Zambia. The present paper is a result of a study within the frame of REACT. Malindi district was chosen due to the relative similarity to the other two districts within the REACT project in terms of disease burden, health system and population [28]. HIV control programmes were defined as one of the several evaluation domains of the project due to the high HIV prevalence in the project countries. The adult (15-49) HIV prevalence is between 15%-17% in Malindi [29].

Research tools

The quantitative study

The World health organization developed and validated a questionnaire to measure responsiveness that incorporates the 7 elements indicated in Table 1, with varying number of questions related to each element [30]. The present study applied the tool that had been implemented in a previous study (among key informants in 35 countries) [3], but tailored it to address 6 of the 7 elements to fit the study setting. The element '*access to social support networks during care*' was omitted because the questions within this section were not deemed applicable for VCT. These questions were relevant in the context of inpatient care [4,7].

To ensure equivalence of the original version, a bilingual English to Swahili translator with medicine, epidemiology and public health background (who also has an understanding of the local language in Malindi area) was asked to perform a back-translation after the English WHO questionnaire had been translated to Swahili by a professional. Where differences were noted, the issues were discussed among the 2 translators, as well as with Swahili speakers at the study area.

Social demographic questions that were included in the questionnaire for the VCT user's i.e. individuals who have utilized VCT services and for the health-care providers whom we refer to as 'providers', captured information on the type of VCT visited, geographical location, sex and age. Marital status was also mapped in the questionnaire for the VCT users.

The health-care providers' questionnaire rated the sub-elements on a 4 point scale ranging from 'never' (1) to 'always' (4) or 'very poor' (1) to 'very good' (4). To meas-

Table 1: Elements as defined in the WHO responsiveness concept [4,7]

Element	**Question Handles (Sub-elements)**
Dignity	The element implies that individuals are treated with respect by being welcomed at the health-care unit and addressed respectfully. It also implies being treated with concern, and being examined in a manner that respects the client's privacy and the right of individuals with infectious diseases such as HIV to be safeguarded.
Autonomy	This element deals with involvement in decision making, and assumes that this can only happen if the users are provided with relevant information, consulted on preferences, and that patients' consent is sought before any proceeding. It also implies that respect is observed on the right of patients of sound mind to refuse treatment.
Confidentiality	This element of responsiveness is related to high maintenance of confidentiality of any information that is provided by the patient, confidentiality of medical records and information about individuals, and privacy during consultations by health providers.
Prompt Attention	This element is defined as care provided readily and as soon as necessary. It includes short waiting-times for treatment or consultations, short-lists for consultations, reasonable waiting-times for appointments, fast care for emergencies as well as the accessibility of the health facility.
Quality of Basic Amenities	This element deals with the extent to which the health facility's physical infrastructure is welcoming and pleasant. It mainly includes clean surroundings, maintenance, adequate furniture, sufficient ventilation, clean water, clean toilets and clean linen.
Choice of Provider	This element is related to the health-care institutions and health providers. It is defined as the power or opportunity to the selection of a provider which requires more than one option. It deals with patients being able to access health services without much difficulty, ability to choose a health-care provider within a health-care unit, individuals being able to get a second opinion, and ability of individuals to get appropriate specialist care.
Social Support	In Hospitals: visits, having special foods, religious practices.

ure the perceived importance of the elements of responsiveness, the study participants (both users and providers), were asked to indicate how important they felt the WHO elements or aspects were on a scale from 0 (not at all important) to 10 (very important). The questionnaire was administered as an exit questionnaire to the users of VCT to generate responses based on their immediate experience with the facilities. A total of 328 VCT users and 36 health-care providers were interviewed.

The qualitative study

The qualitative part of the study consisted of an open-ended question that was added to the quantitative questionnaire as well as the use of observations. The open-ended question was added to allow informants to respond in their own words which in turn permit understanding of responsiveness as seen by the informants. The question sought to bring up potential issues of relevance for responsiveness that were not captured by the existing responsiveness tool. Observation was used in this study to add to our informant's responses. The open question was phrased thus, "in your view are there any other characteristics (other that the ones we have discussed) that you think should be included in a responsive VCT?" A number of probe questions were added to generate more in-depth information on this topic. For example we asked: "What should be done to make VCT services more responsive and to increase its utilization? Probe: Why do you think so? Who should be responsible?" Further probes were formulated depending on the initial responses given. The responses from the users were recorded through hand writing. As the informants felt more comfortable when their responses were not electronically recorded, 4 of the 36 provider's responses were tape recorded.

Study setting

The survey was carried out among all the VCT counsellors or health-care providers who were available as well as among all the users of VCT services in the 15 VCT existing in the district at the time of study (October - November 2007). Most of the VCT facilities in Malindi are integrated or situated within health facilities where there are many other points of provider-initiated HIV testing and counselling such as maternal and child health clinics, tuberculosis clinics and general outpatient services among others. HIV testing and counselling at the VCT facility is mainly client initiated. Anonymity - as documented in the use of code numbers and mother's names was practiced in the Malindi VCT facilities to ensure confidentiality. Those who tested HIV positive were provided with further referral following the ministry of health recommended procedure in order to maintain confidentiality.

Study population

A total of 331 VCT users were asked to participate, out of which 328 accepted to be interviewed for the quantitative part. All the 36 counsellors approached agreed to participate. The study participants were recruited after being informed about the study focus, the voluntary nature of the study and after assurance of confidentiality and anonymity. The inclusion criteria for the 'providers' was VCT staff; while the users were individuals above 18 years who had utilized existing VCT service. The open ended question was addressed to a total of 264 out of the 328 users. Of the remaining 64 participants, 39 of them declined to be interviewed further because they were satisfied with the existing VCT services and 25 did not wish to respond further.

Data collection

The interviews and Observations

All the interviews were carried out face-to-face by the first author [MKN] with assistance from trained and experienced local interviewers. Data was obtained through exit interviews which were seen as the best option for this study in order to avoid recall and recognition bias around perceptions, attitudes and experiences encountered by the VCT users. The quantitative section of the interviews lasted between 30 minutes and one hour, while the qualitative interviews (consisting of the open ended question), took another 30 minutes or more. Among the health providers, English was the primary language of communication, with Swahili words occasionally employed where necessary, while English, Swahili and some Giriama (local language) words were used among the VCT users. All interviews were conducted in a private area or a room provided within the VCT vicinities.

The collected information was kept in a locked cupboard to ensure confidentiality. In addition to the interviews, individual observational field notes were written on a daily basis. These consisted of:exact locations of the VCT, observed dynamics amongst people using VCT, reception of clients by providers, gestures by users, the VCT infrastructure, sitting arrangements before a VCT session, type of health provider and information, education and communication (IEC) material available at the site.

Data Analysis

Quantitative data was analyzed using SPSS version 15 for Microsoft Windows. Graphs and tables were produced using Microsoft Excel and Microsoft Word. In accordance with the WHO approach in a previous study [3], performance outcomes were dichotomised into good performance (combining responses very good and good or always and usually) and poor performance (combining responses poor and very poor or never and sometimes). For the user's data set, 'yes' responses were classified as good performance and 'no' as poor. The importance question was grouped into very important (combining responses 9 and 10), important (5-8) and less important (1-4).

The open-ended responses were mainly translated to English and thematic analysis was employed. Thematic analysis has the following stages that were adapted in the analysis; familiarization with the material, identification of a thematic framework, indexing or coding, mapping and interpretation [31]. The coding process was conducted so as to identify specific pieces of data which were relevant to the responsiveness elements in order to add information.

Ethical aspects

Scientific and ethical approval was obtained from the Kenya Medical Research Institute (KEMRI) and the National Ethical Review Committee (NERC) of Kenya prior to conducting the study (KEMRI SSC number 1273). Written informed consent was obtained from all participants prior to the interviews.

Results

Demographic characteristics

Forty-four percent of the providers were based at the rural VCT centres, with 14% in peri-urban and 42% in urban centres. Among the users, 31% attended the rural, 18% the peri-urban and 51% the urban VCT centres. There were more female users (65%) than male (35%) as indicated in Table 2.

Table 2: Demographic characteristics of the respondents

Characteristic	*Providers*		*Users*	
	n	%	n	%
Setting				
Rural	16	44.4	100	31
Peri-urban	5	13.9	60	18
Urban	15	41.7	168	51
Sex				
Male	20	55.6	115	35
Female	16	44.4	213	65
Age				
18-24	3	8.3	88	27
25-29	11	30.6	68	21
30-34	4	11.1	44	13
35-39	9	25.0	44	13
40 and more	9	25.0	66	20
Type of VCT				
Integrated[1]	32	88.9	268	82
Stand alone[2]	4	11.1	17	5
Mobile clinic[3]	-	-	43	13
Marital status				
Married	-	-	178	55
Single never married	-	-	79	22
Cohabiting/living as married	-	-	38	10
Separated/divorced	-	-	24	7
Widowed	-	-	20	6

[1]Integrated: VCT facilities that are located within a health facility or hospital, [2]stand alone: a VCT that is not within a facility. [3]Mobile seek users in the community on certain days.

The structuring of the findings is done by the responsiveness elements which have further been defined in Table 1. The quantitative and the qualitative findings pertaining to the same element are presented within the same section. Responses from the open-ended questions are referred through direct quotes from study informants. Findings based on the observation or field notes are referred to separately.

Confidentiality was regarded by almost all users (97%) as well as by the providers (94%) as a very important factor to be considered in VCT (Fig. 1). All the providers reported that the confidentiality of information from medical records and consultation sessions was highly observed, while 98% of the users reported that confidentiality during consultations was highly met at the VCT centres (Tables 3)

Both the open-ended responses and the observations to some extent conflicted with the impression that a very high degree of confidentiality was assured at the VCT centre. The qualitative data revealed issues pertaining to confidentiality and privacy that were not captured by the responsiveness tool. Various aspects of the maintenance of privacy and confidentiality were pointed out as lacking. One aspect that was brought up in a number of responses, was the unease experienced at the possibility of meeting health-care providers known by the user, as expressed in the following statement: "*People are scared of going to the VCT because they are afraid that they will know the counsellor and he/she might tell the results to others*" *(a 26 year old female urban VCT user)*. Besides such expressions of the importance of anonymity, a substantial desire to maintain privacy during VCT consultations also emerged in a large number of statements pertaining to the physical arrangement of the VCT facility. An informant noted that "*The VCT room should not be located next to the consultation room for confidentiality reasons. It should be situated in a private area where one is not seen by everyone when entering or coming out*" *(a 38 year female urban VCT user)*. Field observations revealed that at most of the VCT centres, the testing and counselling room was located just opposite or next to the consultation room, as shown in additional file 1. On some occasion, it was possible to hear conversations taking place in the next room. The concept of confidentiality is further illustrated by the following statement from an 18 year male VCT user from a rural setting "*The providers should find a way of making the VCT consultations more private and confidential through more mobile VCT services*".

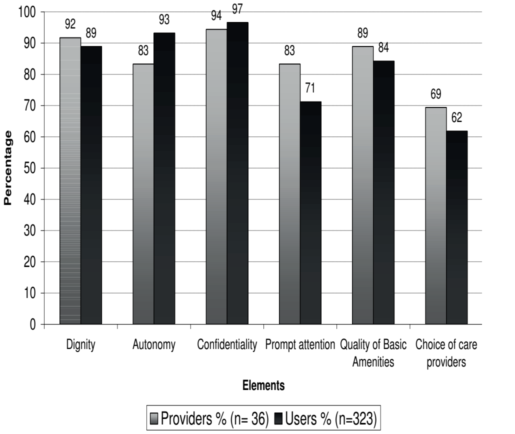

Figure 1
Proportions of Study participants who felt that responsiveness elements were very important[1]. [1]Very important: cut-off point in the rating from 1-10 was 9 or above.

Dignity: A large majority of both the users (89%) and the providers (92%) indicated this element to be very important (Fig. 1). Most of the sub-themes within this element (such as being treated with respect, being encouraged to discuss concerns freely, being encouraged to ask questions and observation of privacy during testing and counselling) were reported to be met, ranging between 97% and 100% among the providers and between 91% and 99% of the users (Table 3). Hence, like confidentiality, dignity was reported to be observed to a very high degree at the VCT facilities.

In relation to this element, the informants brought up aspects that were not captured by the responsiveness tool. Some of the informants pointed out that the providers should greet waiting clients as a gesture of respect. The concerns that were raised were particularly related to the lack of making clients feel relaxed outside the VCT room. Informants commented in a number of ways on a strong unease experienced upon arrival at the VCT centres. "*The providers need to respect the clients by showing them courtesy. For example asking kindly what one is coming for upon arrival. Then they can direct the person to the VCT room" (a 26 year urban VCT user)*.

The physical arrangement of the facility also emerged in the assessment of the observance of dignity at the VCT centre. At times, the location of the entrance, the reception area and the VCT rooms made it necessary for the arriving users to move into highly congested sitting or reception areas in order to ask for directions. A couple of times the users asked the research team for assistance in finding the VCT room. If there was no one present at the entrance to assist, it was quite likely that apprehensive users would not move into the crowd and would shy away from the facility. A respectful health-care provider that would greet the user and discretely direct the user to the VCT room upon arrival was commonly missed.

Autonomy: Again, a large majority of the users (93%) reported this element to be very important. A slightly lower proportion (83%) of the providers found autonomy to be very important (Fig. 1). As with the assessment of confidentiality and dignity, almost all of the providers and users reported that autonomy was highly endorsed at the VCT centres (Table 3).

Some important insights were gained from the qualitative responses. A large number of users reported that they received too little information to be able to make informed decisions and they missed more posters and reading materials on HIV. Take-home material in the local language was particularly asked for so that the topics brought up could be dwelt on in quiet and less stressful surroundings. The issue of the use of the local language was also pointed out in a counselling context where informants needed a language that would make complex messages clear and more understandable to the user. "*The counsellors should be able to speak the local languages because they give us a lot of information. As for me, I did understand some things but not all that the counsellor said" (18 year old female user, peri-urban VCT user)*.

Quality of basic amenities: Like the other elements, the majority of the users and providers scored highly in terms of the importance of this element(84% and 89%). Both groups of the respondents gave high scores on the performance of the element, except those on clean water and toilets which users scored relatively low, primarily because they did not know whether such facilities existed (Table 3).

When we asked the WHO-defined questions on basic amenities, questions and expressions indicated that the informants did not see anything particularly wrong with neither hygiene or maintenance nor content of the current VCT centres, which all had the same lay-out. Most of the informants came from humble homes (additional file 2), and the appearance of the VCT structures emerged to be satisfactory. There was general concern about space, however, especially where it was needed to ensure confidentiality. The providers were also concerned about the lack of space: "*Sometimes we have many clients and there is only one room. If we could have extra rooms where we could have four sessions going on at the same time, this would reduce the waiting time so that you don't lose people (a female counsellor, urban VCT)*. The field-notes recorded that space was some-

Table 3: Proportions reporting good performance* of the responsiveness sub-elements at VCT

Element	*Sub-elements*	*Providers %*	*Users %*
Dignity[1]	Treated with respect	100	99
	Patients encouraged to discuss concerns freely	98	97
	Patients encouraged to ask question about the disease (prevention treatment and care)	97	91
	Patients privacy during testing and counselling	100	95
	Patients privacy during counselling		86
Autonomy[1]	Patients provided with information on prevention and care of HIV	100	95
	Patients consent sought before testing	100	99
	Patient Counselled		98
Confidentiality[1]	Confidentiality observed during consultations	100	98
	Confidentiality of information observed	100	-
	Confidentiality of medical records	100	-
Choice of care[1] provider	Choice between health care providers at VCT	50	29
	Choice between VCT	64	64
Quality of basic amenities[2]	Cleanliness of VCT	97	96
	Furniture availability	89	81
	Maintenance	95	93
	Access to clean water	94	-
	Cleanliness of toilets	91	-
	Testing Kit	100	-
Prompt attention[3]	Waiting times	83	76

*Good performance: cut-off point in dichotomizing ratings on [1]always and, usually, [2]very good and good and [3]30 minutes or less.

times so limited that the counsellors had to compromise confidentiality. On one occasion a counsellor had to counsel a client outside the VCT room because another was being attended to inside the room. The person being counselled outside looked uneasy because the mothers queuing at the clinic opposite were all curious about what was being said.

Prompt Attention: A higher proportion of the provider's (83%) indicated this element to be very important than the users (71%). 76% of the users reported that they waited 30 minutes or less before consultation, while 83% of the providers reported that most users waited 30 minutes and less (Table 3).

The open-ended responses however indicated substantial variation in waiting times, and that these naturally were related to the number of providers available and availability of space. A point brought up by many of the informants was the need to have the counselling services extended to later hours to allow people to visit the VCT centre at a convenient time.

" The centre should be operating up to late hours so that those who are shy to be seen can come for testing when they are comfortable" (a 27 year male urban VCT user)

Choice of Provider: In terms of importance, this element was assessed as the least important of the WHO elements. Only 62% of the users and 69% of the providers rated it as very important (Fig 1). The results also indicate that this element was reported to be the least observed or met within the VCT services. A majority of the users (71%) reported that a choice of health provider was not offered while 50% of the providers indicated that this element was rarely observed. 36% of the users experienced that there was no choice of VCT centres (Table 3).

Access to health services (in this case VCT) was mentioned as one of the sub-elements that should be covered by this element, as indicated in Table 1. The applied tool did not include questions that measure accessibility. Results from the open-ended interviews suggested that accessibility was an issue that highly influenced VCT utilization and choice of VCT centre. *"The costs of coming to the centre should be free. It is not easy to get to the VCT because it is far from my home and the transport money is expensive" (A 35 years old male urban VCT user)*. A number of informants suggested that VCT should ideally reach people in their homes or in their home environments, which could be facilitated through e.g. home-based VCT or through more mobile VCT units than are commonly offered.

Other relevant aspects that emerged from the study

Three aspects - access to social support, continuity and follow-up, and quality of counselling and testing were not captured by the WHO tool but came up from the qualitative responses and could be essential to responsiveness in the context of VCT.

Access to social support: This element was (as stated earlier) not included in our survey due to the nature of the questions included in the WHO tool. However in the present study, the open-ended responses revealed the importance of this element from a social-economic support point of view. Many informants mentioned that support groups should be linked to VCT centres to encourage more people to utilize the services. "*There should be a partnership between the VCT and the help groups to encourage more clients to come and get tested" (a 48 year female rural VCT user)*. For others, support groups were considered important in providing some provisional social economic support. "*To help us who are positive. I don't have anywhere to sleep and I have no strength" (a 48 year female urban VCT user)*.

Continuity and Follow-up: Responses on follow-up care after testing and counselling were also strongly called for. Firstly, the responses indicated the need for VCT centres to attend to the users' needs and expectations in a more comprehensive manner by offering follow-up counselling both to those found positive and those found negative. Provision of drugs to those infected, provision of more condoms for prevention, family planning services and testing for other sexually transmitted diseases were also considered essential to be provided at a VCT centre. Laboratory tests were found to be a central issue "*A VCT centre should have reagents and a CD4 count machine that is working. The stopping make a portion of the clients drop out, and others suffer because in most cases people feel more free when they are taking the CD4 counts. Then they understand their viral development. That's when they get the morale to come back and start on medication" (a female urban VCT provider)*. Other informants indicated the need for all VCT centres to provide comprehensive care. "*Action should be taken on the spot. There should be no referrals to other places. Some of the places we are referred to, are very far away and we need transport to reach there" (39 year female, urban VCT provider)*.

Secondly, for the VCT centres that were integrated in a comprehensive care clinic, there was an expressed desire for reliable care by the same provider: "*those providers that work at the VCT unit should remain at the facility permanently because the movement of the providers to different department brings confusion for example related to medication of ARV follow up. Despite the records, the discussions and compromises that are not recorded are very important" (female urban VCT provider)*.

The quality of counselling and testing: Various aspects related to the quality of the services emerged as central during the responses to the open-ended questions. This calls for a section that addresses quality of the services within the responsiveness tool. The number, availability and training of professional health personnel were reported by many as being insufficient. "*There should be highly trained counsellors who will counsel their clients properly so that the client leaves the place without doubts of what to do" (A 36 year male urban VCT user)*. Some expressed a lack of trust in the knowledge on HIV and AIDS provided by the providers "*The counsellors should tell the truth because some of the clients have more knowledge about HIV and AIDS than the providers" (a 27 year male urban user)*. Some of the concerns relating to the training of the counsellors that were expressed by the providers themselves confirmed the lack of sufficient knowledge. "*There is a need for refresher courses because some of the staff members were trained a long time ago. You hear them counselling clients on the corridor's but it is difficult to correct them as they are older and they feel that they know" (a female urban VCT provider)*.

Discussion

Efforts to measure health systems responsiveness are still weak; and to our knowledge the responsiveness concept has not previously been applied to HIV-related services. In the present study, we found that all the elements that are suggested to measure responsiveness by the WHO tool deemed very important by most users and providers at VCT centres. The elements capturing respect for person's (confidentiality, dignity and autonomy) were more frequently identified as very important compared to the elements capturing client orientation (quality of basic amenities, prompt attention and choice of care providers). These results differ somewhat from a responsiveness study conducted on health systems in general in 41 countries which reported that prompt attention was the most important element, but were similar in the sense that dignity and autonomy were highly valued [32,33]. The high importance of confidentiality brought out in this study corresponds well with observations from resource-poor settings showing this element as a critical factor affecting acceptability or uptake of VCT [17,18,20-22] which is a key intervention in HIV/AIDS prevention and care [34]. Confidentiality seems to be a major factor explaining the very high acceptance rates that have been achieved when VCT has been offered at home [22,35]. Similar results were seen in a pilot study conducted in 2 districts in Kenya, which showed high acceptance of home-based HIV testing [35]. Protection of confidentiality in these settings is not only seen as an important aid to continuum of care, but crucial in reducing stigma [36].

Concern has been expressed over in the literature on how well the issue of confidentiality is handled relative to HIV

testing [17,20,34,37]. The present study revealed various aspects of confidentiality that are not captured by the employed responsiveness tool. A finding that pertains to a number of elements in the tool was that the questions seem to capture the client's experiences with the health system at the time of VCT service provision inside the consultation room, but leaves out contextual aspects that may influence responsiveness substantially. Furthermore, the users expressed worries about meeting someone they knew among the clinic staff who might breach the confidentiality. This finding is in concurrence with results from various studies on factors affecting readiness and use of VCT [17,18,20], and is in particular seen as a barrier to HIV testing in places where women are prone to divorce and domestic violence if their spouses get to know they are HIV positive [38,39].

Unlike other health services, VCT services are often linked to HIV infection which is itself a stigmatized state. Stigma on the other hand affects the dignity of those infected by portraying them as persons with loose morals [40-42]. Understandably, confidentiality would be an issue of concern in the case of VCT services compared to other health services such as testing for malaria. In this case, efforts to normalise HIV testing represented by the provider initiated (opt-out) strategy are increasingly employed [43]. However, this strategy puts the process under the control of the provider when clients may not be psychologically prepared for the test. Consequently, provider-initiated testing is in greater danger of meeting with lower responsiveness than client-initiated testing done at VCT sites. The opt-out strategy has been strongly criticised for putting a low focus on counselling with the risk of undermining autonomy and reducing the focus on the preventive aspects of HIV testing [37,39,44].

Providing enough information in a language that is understandable to the users or clients is an important basis of the autonomy of the client. It was disturbing in this regard to find the informants responding to the survey questions that they were indeed informed, provided with information and given a high degree of autonomy in the counselling context, and then later to find that they understood little of the language spoken. Provider's and users of the VCT facilities expressed high expectations on provision of HIV knowledge at the facility. Providers however pointed out a need for more courses while the users indicated a lack of trust in the quality of the knowledge of HIV and AIDS among some of the providers. Information is a powerful tool in prevention and care of HIV [45], and is of critical importance in counselling processes covering both psycho-social and the preventive aspects [16]. This also challenges the responsiveness tool particularly in covering the communication process and content of information presented extensively.

Some aspects of client orientation did not appear to have been captured by the tool. Responses related to the Choice of care provider indicated that access to HIV testing has been seriously hampered by unaffordable indirect costs, such as long distances to travel to the VCT. This may in part explain the low proportion tested in high HIV prevalence countries [33]. Access to social support was also indicated as important from different perspectives. Economic support is one of the important measures of the continuum of care for HIV; if absent, it can be a barrier to better care for the people living with the virus [33].

Scaling up of HIV testing has been ongoing in many sub-Saharan countries, and in Kenya there were over 900 VCT sites in 2008 [35]. The scaling up has put priority on decentralizing VCT services as much as possible, a strategy that succeeded in making testing and preventive counselling much more easily available over a relatively short period of time. Our informants seemed concerned by the limitation of these services, revealed by the suggestions to apply the principle of continuity or follow-up of care. *"Then what, if diagnosed HIV positive or HIV negative?"* was a common question raised by users. Considering the high degrees of stigma that corresponds to fewer disclosures of HIV status [46], follow-up services and functioning referral becomes vital in this context. Comprehensive care clinics (CCC) have been established in central hospitals to offer integrated HIV services. The CCC offers a variety of services including some of what were mentioned by our informants like STIs and ART delivery [47]. From a perspective of HIV prevention, decentralised acceptable VCT services with high focus on preventive counselling should clearly be given higher priority compared to spending more on decentralising comprehensive care. However, the quality of counselling services appeared in this study to need further strengthening, and counsellors should be given a clear mandate in terms of follow up including referral. As an additional responsiveness element, continuity contributes to better quality of care [48] and is an expectation not only related to HIV infection as suggested by our respondents, but to other chronic illnesses, as described elsewhere [49]. Embracing continuity, comprehensiveness and integration within responsiveness is in line with visions of primary health-care [50].

Previous studies on responsiveness have focused mainly on the evaluation of the entire health systems within or amongst different countries [3,9,10,12,32] but our analysis concurs with other studies [11,49] in suggesting that the responsiveness concept can also be applied in specific parts of the health system. However, there are weaknesses in our study. We expected that the VCT users' views would have been more critical than the providers, as indicated in previous studies [51]. The failure to capture such a discrepancy may have been aggravated by the fact that we conducted the exit interviews at the facilities after entrust-

ing the providers to alert the study participants. Conducting exit interviews at a VCT setting was challenging because the test results of the respondents could have emotionally influenced the responses. Some of the respondents could have given rushed responses due to the long waiting hours and the study setting could have been a challenge as opposed to home setting. In an attempt to minimize this challenge, we tried to make our respondents comfortable before embarking on the interviews and we made it clear that the test results were not important to our study.

The survey suggested a very high performance of the responsiveness elements at the VCT facilities, but mixing research methods helped us unveil issues that would not have been captured by the quantitative part of the study alone. A weakness of the qualitative part of the study was the taking of notes rather than audio-recording. Only four of these interviews were audio recorded thus, it is possible that not all the informants' statements were fully captured. We did however attempt to recode as much as possible by asking the informants to talk slowly and to repeat central messages so they could be recorded word by word. We also made observations and took photographs to visualize the settings.

Conclusions

The findings of this study go a step further than other studies in identifying potential weaknesses in the responsiveness tool and in identifying dimensions that could be incorporated in the WHO tool. It is likely that the current findings apply more widely than to Malindi alone. The responsiveness elements proposed by WHO were all given very high ratings in the context of voluntary HIV counselling and testing. However, the study findings indicate that the tool will need substantial adjustments to capture important dimensions and perspectives. Firstly, adjustments are needed to penetrate dimensions related to the elements most valued by the respondents (confidentiality, dignity and autonomy). Secondly, there is a need to add elements that are not covered by the applied tool, such as the need to address not only dimensions inside the facility but aspects of the surrounding environment such as location of the facility potential to securing confidentiality outside the VCT room, follow-up care as well as social support. Thirdly, assessment and recognition of locally specific aspects and meanings of the elements seems of particular importance before conducting comparative studies on responsiveness of HIV testing services.

Competing interests

The authors declare that they have no competing interests.

Authors' contributions

MKN prepared the study proposal, coordinated and supervised data collection, conducted the interviews, analyzed the data, interpreted the findings and wrote the manuscript. AB was involved in developing the study proposal and analyzing the qualitative data, and also took an active role in interpreting the findings and revising the manuscripts. IKN contributed in developing the study proposal and took an active part in revising the manuscript. KF was involved in developing the study proposal, analyzing the quantitative part of the data and took an active part in the interpretation and revision of the manuscripts.

Additional material

Additional file 1

VCT facility located next to a general consultation room*. A photo showing how close the VCT location is to the general consultation room. The closeness of these two rooms made it almost impossible to access VCT in fear of being seen by a known provider or other users.*
Click here for file
[http://www.biomedcentral.com/content/supplementary/1472-6963-9-243-S1.DOC]

Additional file 2

A typical residential home in rural Malindi vs VCT facility*. A photo showing the kind of homes that most of the study participants live in (left) in comparison to the VCT facility (right) they were requested to evaluate.*
Click here for file
[http://www.biomedcentral.com/content/supplementary/1472-6963-9-243-S2.DOC]

Acknowledgements

We thank the Norwegian government's Quota program for financial support. The authors are grateful to the VCT counsellors, all the study participants who provided their time for the interviews and the team from the Central Bureau of Statistics in Malindi district that assisted in collecting the data. We also thank the EU-funded REACT project within which this study has been conducted and the Kenya Medical Research institute (KEMRI) for allowing it to be conducted. We are very grateful to the Centre for Public health research (CPHR) - KEMRI for their input in improving the study proposal, Dr. Yeri Kombe for his administrative and scientific assistance during the study, Moses Mwangi for his assistance in sampling and data management and analysis, and Titus Kitaka for his assistance with transportation at the field sites. All authors read and approved the final manuscript.

References

1. Murray CJ, Frenk J: **A framework for assessing the performance of health systems.** *Bull World Health Organ* 2000, **78(6):**717-731.
2. WHO: *Health systems: improving performance* Geneva: World Health Organization; 2000.
3. De Silva A, Valentine N: **Measuring responsiveness: Results of a key informant survey in 35 countries.** In *GPE Discussion Paper series No 21* Geneva: WHO; 2000.
4. Murray CJL, Evans DB: *Health systems performance assessment: Debates, methods and empiricism* Geneva: World Health Organization; 2003.
5. Clearly P: **The increasing importance of patient surveys.** *BMJ* 1999, **319:**720-721.
6. De Silva A: **A framework for measuring responsiveness.** In *GPE Discussion Paper series No 32* Geneva: WHO; 2000.

7. Darby C, Valentine N, Murray CL, De Silva A: **World Health Organization: Strategy on measuring responsiveness.** In *GPE Discussion Paper series Volume 23*. WHO; 2000.
8. Gilson L: **Trust and the development of health care as a social institution.** *Social Science and Medicine* 2003:1453-1468.
9. Valentine N, Silva A, Murray CJL: **Estimating responsiveness level and distribution for 191 countries: Methods and results.** In *GPE Discussion paper Series: No 22* Geneva: World Health Organization; 2000.
10. Hsu Cc, Chen L, Hu YW, Yip W, Shu CC: **The dimensions of responsiveness of a health system: A Taiwanese perspective.** *BMC Public Health* 2006, **6(72):**.
11. Bramesfeld A, Wedegartner F, Elgeti H, Bisson S: **How does mental health care perform in respect to service users' expectations? Evaluating inpatients and outpatients care in Germany with the WHO responsiveness concept.** *BMC Health Service Research* 2007, **7(99):**1-12.
12. Ugurluoglu O, Celik Y: **How Responsive Turkish health care system is to its citizens: The views of Hospital managers.** *J Med Syst* 2006, **30(6):**421-428.
13. Navarro V: **Can health systems be compared using a single measure of performance?** *American Journal of Public Health* 2002, **92(1):**31-34.
14. UNAIDS: **Voluntary Counselling and Testing (VCT).** Geneva: UNAIDS; 2000.
15. Merson MH, O'Malley J, Serwadda D, Apisuk C: **The history and challenge of HIV prevention.** *Lancet* 2008, **372:**475-488.
16. UNAIDS, WHO: **UNAIDS/WHO policy statement on HIV testing.** 2004.
17. Fylkesnes K, Haworth A, Rosenvard C, Kwapa P: **HIV counselling and testing: Overemphasizing high acceptance rates a threat to confidentiality and the right not to know.** *AIDS* 1999, **13(17):**2469-2474.
18. Irungu T, Varkey P, Cha S, Patterson J: **HIV voluntary counselling and testing in Nakuru, Kenya: Findings from a community survey.** *British HIV Association HIV Medicine* 2008, **9(2):**111-117.
19. Shin S, Kang H, Moneyham L: **Characteristics of individuals seeking voluntary counselling and testing for HIV infection in South Korea.** *Journal of the association of nurses in AIDS care* 2007, **18(5):**27-33.
20. Fylkesnes K, Siziya S: **A randomized trial on acceptability of voluntary HIV counselling and testing.** *Tropical Medicine and International Health* 2004, **9(5):**566-572.
21. Beteganya MH, Abdulwadud OA, Kiene SM: **Home based HIV voluntary counselling and testing in developing countries.** *Cochrane database of systematic Reviews* 2007.
22. Angoti N, Bula A, Gaydosh L, Kimchi EZ, Thornton RL, Yeatman SE: **Increasing the acceptability of HIV counselling and testing with three C's: Convinience, confidentiality and credibility.** *Social Science and Medicine* 2009, **68(12):**2263-2270.
23. **Epidemiological fact sheets on HIV and AIDS, Kenya** [http://www.who.int/globalatlas/predefinedReports/EFS2008/full/EFS2008_KE.pdf]
24. Ministry of Health, National Aids and STI Control Programme: *AIDS in Kenya trends, Interventions and Impact* 7th edition. Nairobi: Ministry of Health, National AIDS and STI Control Programme; 2005.
25. Johnson RB, Onwuegbuzie AJ: **Mixed Methods Research: A research paradigm whose time has come.** *Educational Researcher* **33(7):**14-26.
26. Creswell JW, Plano Clark VJ, Hanson WE: *A handbook of mixed methods in social and behavioral research* California: Sage publications Inc; 2003.
27. Kelle U: **Combining qualitative and quantitative methods in research practice: purposes and advantages.** *Qualitative research in psychology* 2006, **3(4):**293-311.
28. Byskov J, Bloch P, A B, Hurtig AK, Fylkesnes K, Kamuzora P, Kombe Y, Kvåle G, Marchal B, Martin DM, Michel C, Ndawi B, Ngulube TJ, Nyamongo I, Olsen1 ØE, Onyango-Ouma W, Sandøy IF, Shayo EH, Silwamba G, Songstad NG, Tuba M: **Accountable priority setting for trust in health systems - the need for research into a new approach for strengthening sustainable health action in developing countries.** *BMC Health Research Policy and Systems* 2009, **7(23):**. 24 October 2009
29. Ministry of Finance and planning K: **Malindi district development plan 2002-2008.** Nairobi: Republic of Kenya; 2002.
30. **Key informant survey on responsiveness** [http://www.who.int/responsiveness/surveys/Ques%20for%20KIS%20WHR2000.pdf]
31. Lacey A, Luff D: *Trent focus for research and development in primary Health Care: An introduction to qualitative data analysis* Trent Focus Group; 2001.
32. Valentine N, Darby C, Bonsel G: **Which aspects of non clinical quality of care are most important? Results from WHO's general population surveys of health systems responsiveness in 41 countries.** *Social science and Medicine* 2008, **66(9):**1939-1950.
33. Kerssens J, Groenewegen P, Sixma H, Boerma W, Eijk I: **Comparison of patient evaluations of health care quality in relation to WHO measures of achievement in12 European countries.** *Bull World Health Organ* 2004, **82(2):**106-114.
34. Makoae MG, Jubber K: **Confidentiality or continuity? Family caregivers' experiences with care for HIV/AIDS patients in home-based care in Lesotho.** *Journal of Social Aspects of HIV/AIDS* 2008, **5(1):**36-46.
35. National AIDS and STI Control Programme, Ministry of Public health and sanitation Kenya: **National guidelines for HIV testing and counselling in Kenya.** *Nairobi* 2008.
36. Andrew SF, Ian JH, Alice D, David SM: **Barriers to better care for people with AIDS in developing countries.** *BMJ* 2004, **329:**1281-1283.
37. Yeatman SE: **Ethical and public health considerations in HIV counselling and testing: Policy implications.** *Studies in Family Planning* 2007, **38(4):**271-278.
38. Pool R, Nyanzi SI, Whitworth JAG: **Attitudes to voluntary counselling and testing for HIV among pregnant women in rural south-west Uganda.** *AIDS CARE* 2001, **13(5):**605-615.
39. Maman S, King E: **Changes in HIV testing policies and the Implications for Women.** *Journal of Midwifery & Women's Health* 2008, **53(3):**195-201.
40. Davis PC, Rankin LL: **Gay Men and Loose Women - Southeast Idaho Mexican Migrant Women's Beliefs about Who Gets HIV.** *Californian Journal of Health Promotion* 2006, **4(3):**42-46.
41. Ogden J, Nyblade L: **Common at Its Core: HIV-Related Stigma across Contexts.** International Centre for Research on Women (ICRW); 2005:15-30.
42. Skinner D, Mfecane S: **Stigma, discrimination and the implications for people living with HIV/AIDS in South Africa.** *Journal of Social Aspects of HIV/AIDS* 2004, **1(3):**157-164.
43. Creek TL, Ntumy R, Seipone K, Smith M, Mogodi M, Smit M, Legwaila K, Molokwane I, Tebele G, Mazhani L, Shaffer N, Kilmarx PH: **Successful Introduction of Routine Opt-Out HIV Testing in Antenatal Care in Botswana.** *J Acquir Immune Defic Syndr* 2007, **45(May 1 2007):**102-107.
44. Obermeyer MO, Osborn M: **The utilization of testing and counselling for HIV: A review of the social and behavioural evidence.** *Am J Public Health* 2007, **97(10):**1762-1774.
45. Bruce J: **Fundamental elements of the quality of care: a simple framework.** *Studies in family planning* 1990, **21(2):**61-91.
46. Smith R, Rossetti K, Peterson BL: **A meta analysis of disclosure of one's HIV-positive status, stigma and social support.** *AIDS Care* 2008, **20(10):**1266-1275.
47. Taravella S: **Comprehensive Care centres and Antiretroviral drugs: bringing new life to Kenyans within HIV.** Family Health International, Institute for HIV/AIDS; 2005.
48. Haggerty J, Reid R, Freeman G, Starfield B, Adair C, McKendry R: **Continuity of care: A multidisciplinary review.** *BMJ* 2003, **327:**1219-1221.
49. Bramesfeld A, Klippel U, Seidel G, Schwartz F, Dierks M: **How do patients expect the mental health service system act? Testing the WHO responsiveness concept for its appropriateness in mental health care.** *Social Science and Medicine* 2007:880-889.
50. WHO: **Primary health care now more than ever.** Geneva: World Health Organization; 2008.
51. Blendon RJ, Kim M, Benson JM: **The public versus the World Health Organization on health system performance.** *Health Affairs* 2001, **20(3):**10-20.

Pre-publication history

The pre-publication history for this paper can be accessed here:

http://www.biomedcentral.com/1472-6963/9/243/prepub

10 APPENDICES

I

REACT File, EU Project Office

KENYA MEDICAL RESEARCH INSTITUTE

P.O. Box 54840-00200 NAIROBI, Kenya
Tel: +254 (020) 2722541, 2713349, 0722-205901, 0733-400003, Fax: +254 (020) 2720030
E-mail: director@kemri.org; info@kemri.org Website: www.kemri.org

K.E.M.R.I.
CENTRE FOR PUBLIC HEALTH RESEARCH
P.O. Box 20752
7 SEP 2006
NAIROBI

KEMRI/RES/7/3/1 **17th Aug 2006**

Dr. Y Kombe,
CPHR,
NAIROBI.

Noted 8/09/06

Dear Sir,

Re: SSC Protocol No. 1096 (Revised) – **Strengthening fairness and accountability in health systems priority setting for improving equity and across to quality health care in Malindi District, Kenya, by Y Kombe *et al***

During the 136th Meeting of the KEMRI/National Ethical Review Committee held on the 15th of August 2006 the above protocol was discussed.

The committee notes that this study aims to promote and evaluate the application of Accountability For Reasonableness (AFR) as a tool to improve health services in the areas of equity, accessibility and quality. Due consideration has been given to ethical issues therefore the study is granted approval.

You are responsible for reporting to the Ethical Review Committee any changes to the protocol or in the Informed Consent Document. This includes changes to research design or procedures that could introduce new or more than minimum risk to human subjects.

You may proceed with your study.

RCMKithinji

R.C.M. Kithinji
For: Secretary,
KEMRI/National Ethical Review Committee

THE UNITED REPUBLIC OF
TANZANIA

National Institute for Medical Research
P.O. Box 9653
Dar es Salaam
Tel: 255 22 2121400/390
Fax: 255 22 2121380/2121360
E-mail: headquarters@nimr.or.tz
NIMR/HQ/R.8a/Vol. IX/416

Ministry of Health
P.O. Box 9083
Dar es Salaam
Tel: 255 22 2120262-7
Fax: 255 22 2110986

03rd March 2006

Dr Peter Kamuzora
University of Dar es Salaam
P O Box 35091
DAR ES SALAAM

CLEARANCE CERTIFICATE FOR CONDUCTING MEDICAL RESEARCH IN TANZANIA

This is to certify that the research entitled: Strengthening fairness and accountability in priority setting for improving equity and access to quality health care at district level, in Tanzania, Kenya and Zambia. *(Kamuzora P et al)* whose Principal Investigator is Peter Kamuzora, has been granted ethics clearance to be conducted in Tanzania.

The Principal Investigator of the study must ensure that the following conditions are fulfilled:

1. Progress report is made available to the Ministry of Health and the National Institute for Medical Research, Regional and District Medical Officers after every six months.
2. Permission to publish the results is obtained from National Institute for Medical Research.
3. Copies of final publications are made available to the Ministry of Health and the National Institute for Medical Research.
4. Any researcher, who contravenes or fails to comply with these conditions, shall be guilty of an offence and shall be liable on conviction to a fine.

Name: Dr Andrew Y Kitua

Signature

CHAIRMAN
MEDICAL RESEARCH
COORDINATING COMMITTEE

Name: Dr Gabriel L Upunda

Signature

CHIEF MEDICA L OFFICER
MINISTRY OF HEALTH

THE UNIVERSITY OF ZAMBIA

RESEARCH ETHICS COMMITTEE

Telephone: 260-1-256067
Telegrams: UNZA, LUSAKA
Telex: UNZALU ZA 44370
Fax: + 260-1-250753
E-mail: unzarec@zamtel.zm

Ridgeway Campus
P.O. Box 50110
Lusaka, Zambia

Assurance No. FWA00000338
IRB00001131 of IORG0000774

15 September, 2006
Ref.: 002-05-06

Dr Jens Byskov
C/O Prof. S. Siziya
Department of Community Medicine
School of Medicine
University of Zambia
LUSAKA

Dear Dr Byskov,

RE: RESEARCH PROPOSAL ENTITLED: **"STRENGTHENING FAIRNESS AND ACCOUNTABILITY IN PRIORITY SETTING FOR IMPROVING EQUITY AND ACCESS TO QUALITY HEALTH CARE AT DISTRICT LEVEL IN TANZANIA, KENYA AND ZAMBIA"**

The above research proposal was presented to the Research Ethics Committee meeting on 16 May, 2006 where changes were recommended. We would like to acknowledge receipt of the corrected version with clarifications. The proposal has now been approved. Congratulations!

CONDITIONS:

- This approval is based strictly on your submitted proposal. Should there be need for you to modify or change the study design or methodology, you will need to seek clearance from the Research Ethics Committee.
- If you have need for further clarification please consult this office. Please note that it is mandatory that you submit a detailed progress report of your study to this Committee every six months and a final copy of your report at the end of the study.
- Any serious adverse events must be reported at once to this Committee.
- Please note that when your approval expires you may need to request for renewal. The request should be accompanied by a Progress Report (Progress Report Forms can be obtained from the Secretariat).

Yours sincerely,

for Prof. J. T. Karashani, MB, ChB, PhD
CHAIRMAN

Date of approval: 15 September, 2006 **Date of expiry:** 14 September, 2007

REACT Scientific Committee

1st March 2010
Review of Request for use of data by Mercy Karimi Njeru, KEMRI

Intro

On 16 February 2010, the scientific Committee received a request for use of REACT data by Mercy Karimi Njeru.

Mercy Karimi Njeru is a researcher at the Centre for Public Health Research (CPHR) of KEMRI (Kenya) and has been involved in the REACT project from the start. She is working on a PhD called "*Assessment of Equity and Responsiveness of the VCT Centers and Priority setting processes in HIV approaches: A study of health care workers, users and community members of Malindi District*" (see annex), at the Centre for International Health, University of Bergen (Norway). Prof. Fylkesnes, Blystad, Nyamongo and Kvale and dr. Kombe are her supervisors.

The request

Mercy requests to use more data than she applied for in 2008 in order to do comparisons in analysis. This will be disseminated through published material in peer reviewed journals and through a thesis that make up the requirement fro a PhD at the University of Bergen.

In the meanwhile, her PhD study has been refocused on HIV testing strategies using a mixed methods approach. Her study is particularly connected to Work package 6 of the REACT project. The study identifies potential gaps within HIV testing services (part of the health system) necessary to be strengthened for trust, equity and quality. This is critical for HIV prevention. The identified gaps also provide a potential platform for changes in the HIV testing and counselling services using the fair and legitimate priority setting conditions of AFR (the intervention being applied in the REACT project).

Mercy requests the use of the following data:

1. PBS sections on HIV from all the three districts
2. Facility based focus group discussions sections on HIV from all 3 districts.

It should be noted that she has collected herself additional data in Malindi that will be used together with the requested data.

Assessment of the request according to SOP Accessing and Publishing REACT data

Besides the procedures regarding authorship of publications, to which PhD students will adhere, this SOP identifies a number of additional conditions for PhD studies:

1. "The students/researchers conducting *associated studies* must operate through, and have formalised collaborative agreements with, one or more REACT partner institutions."

- In this case, this condition has been met: Mercy actually works for KEMRI, a REACT partner institution.

2. "In case of PhD studies, the PhD student is entitled to have first authorship on PhD publications if meeting the above criteria for authorship credit."

    - Adherence to the authorship rules will be followed up once publications are being written on the basis of this work, so that the rights of other researchers involved in the data collection and analysis are respected.

3. "The study must not interfere with REACT interventions and research activities in the field unless the nature of interference is properly described, considered and accepted by REACT"

    - As far as can be seen at this stage, this is not a concern given that the data collection has already been carried out.

Additional information

An important point is the involvement of the PhD candidate in the studies from which she will use data. Mercy participated in the design of data collection tools for the work packages 6-9 during the Kunduchi workshop (November 2005). During the workshop Mombasa workshop, she worked in the group assigned to develop the *data collection (PBS) tool* for work package 6 to 9. During May 2007 Machakos workshop, she presented her concept for a PhD study, which was accepted at CIH, University of Bergen.

Recommendation of the Scientific Committee

Given the relevance of the research topic and the research questions of this PhD study, and their coherence with the overall REACT research questions, the SciComm recommends to the Steering Committee to give the green light to Mercy Karimi Njeru to allow her to use the specified collected data.

In accordance with the SOP 'Accessing and Publishing REACT data', we would propose a condition: the publications derived from field work in which the candidate was not directly involved should be co-written with REACT staff who actually designed the field study or were involved in analysis of the data. The candidate can be first author if the Vancouver Declaration rules are respected.

Similar as to the previous request for use of data, the co-supervisor should ensure that the interests of other PhD students or researchers, who were (also) involved in the study design, data collection, and data analysis, are respected.

In follow up of the meeting at Kunduchi (October 2009), it should be stressed that any potential publication from this study should be announced to the SciCoM so that any REACT researcher wishing to contribute to this paper could identify him/herself and participate in writing.

Bruno Marchal for the Scientific Committee

10. April 2011
This final approval of data use is hereby confirmed
Jens Byskov
REACT COORDINATOR

Jens Byskov, Specialist in Public Health

DBL - Centre for Health Research and Development

Faculty of Life Sciences, University of Copenhagen
Thorvaldsensvej 57, DK 1871 Frederiksberg, Denmark

jby@life.ku.dk, Tel. +45 35331418, Cell +45 23393204,
Fax 35331433. www.dbl.life.ku.dk

K.E.M.R.I
RECEIVED
23 AUG 2007
P.O. Box 20752-00202, NAIROBI.
CENTRE FOR PUBLIC HEALTH RESEARCH

KENYA MEDICAL RESEARCH INSTITUTE

P.O. Box 54840 - 00200 NAIROBI, Kenya
Tel: (254) (020) 2722541, 2713349, 0722-205901, 0733-400003; Fax: (254) (020) 2720030
E-mail: kemri-hq@nairobi.mimcom.net: director@kemri.org; Website: www.kemri.org

ESACIPAC/SSC/2101 **22nd August, 2007**

M. K. Njeru

Thro'

Director, CPHR
NAIROBI

forwarded 27/08/07

REF: SSC No. 1273 – Assessment of Equity and Responsiveness of the VCT centres priority-setting processes in HIV approaches: A study of health care workers, users and community members at Malindi district, Kenya

I am pleased to inform you that the above mentioned proposal in which you are the PI, was approved for implementation by the KEMRI Scientific Steering Committee (SSC), during its 137th SSC meeting held on 3rd July, 2007 and has since been forwarded to the Ethical Review Committee (ERC) for consideration.

The SSC however, advises that work on this project can only start when ERC approval is received.

C. Mwandawiro, PhD
SECRETARY, SSC

KENYA MEDICAL RESEARCH INSTITUTE

P.O. Box 54840 - 00200 NAIROBI, Kenya
Tel: (254) (020) 2722541, 2713349, 0722-205901, 0733-400003; Fax: (254) (020) 2720030
E-mail: kemri-hq@nairobi.mimcom.net: director@kemri.org; Website: www.kemri.org

KEMRI/RES/7/3/1 **AUGUST 28, 2007**

FROM: **SECRETARY, KEMRI/National Ethical Review Committee**

THROUGH: **THE DIRECTOR, CPHR** Forwarded 29/08/07

TO: **Ms. Mercy K. Njeru**

RE: **SSC No. 1273 (Revised) - Assessment of Equity and Responsiveness of the VCT Centres and Priority setting processes in HIV approaches: A Case Study of Health Care Workers, Users and Community Members of Malindi District**

Reference is made to your letter dated 22nd August 2007. We acknowledge receipt of the revised proposal and the Kiswahili-translated Informed Consent Document (ICD).

The Committee notes the revisions made to the ICD clearly defining the informed consent process and is satisfied that due consideration has been given to the ethical issues that may arise from the study.

The study is hereby granted approval for a period of one (1) year effective today, the 28th day of August 2007 to 27th August 2008.

Please note that you are responsible for reporting any changes to the approved protocol prior to implementation. This includes changes to research design or procedures that could introduce new or more than minimum risk to the research participants.

Yours faithfully,

C. Wasunna,
For: SECRETARY,
KEMRI/NATIONAL ETHICAL REVIEW COMMITTEE

PBS (1A)

II

Questionnaire population based survey REACT 2006 (WP 6-9)

- Type of setting (1 = rural, 2 = peri-urban, 3 = urban) ☐
-
- ID number ☐☐☐☐☐

  (ID number is composed of country code (1=Kenya, 2=Tanzania 3= Zambia) cluster number (01-45) and household number (01-XX)

- Number of household members? ☐☐

- Date of interview (dd/mm/yyyy) __ __/ __ __ /__ __ __ __

Fill in the information for all adults in the household age 15-49 in the table below

	Age (in years) (9= don´t know)	Sex (1 = male) (2 = female)	Availability (1 = present) (2 = absent)	Selected by randomization for interview[1] (1 = yes) (2 = no)	Willingness to participate[2] (1 = accepted) (2 = refused)
Adult 1					
Adult 2					
Adult 4					
Adult 5					
Adult 6					
Adult 7					
Adult 8					
Adult 9					
Adult 10					

- Status of Interview? (1 = completed, 2 = not completed) ☐

IF ALL REJECT STOP HERE AND MOVE TO THE NEXT HOUSEHOLD

[1] Select randomly one male and one female from the household members present age 15-49.
[2] If only one male or one female in your sample continue with the questionnaire but add another household to the list.

1. What is your age? (indicate age in years, or 9 = don't know) ☐☐

2. Date of birth (dd/mm/yyyy): __ __/ __ __ /__ __ __ __

3. Sex (1 = male, 2 = female) ☐

4. What is your ethnic background? (or what language is your mother tongue?) ☐
From numbered List of all possible. Also write actual response here……………………….

5. For how long have you been living continuously in this area? ☐☐
(0 = if less than 1 year, else indicate the number of years)

6. What is your marital status? (1 = single, never married, 2 = cohabitating/living as married, 3 = married, 4 = widowed, 5 = separated/divorced) ☐

If "single/never married", skip question 7.

7. How old were you when you first got married? ☐☐
(indicate age in years or 9 = don't know)

8. Did you ever attend school? (1 = yes, 2 = no) ☐

9. What is the highest level of school you have attended? ☐
(1 = informal, no formal education, 2 = primary school, not completed,
3 = primary school completed, 4 = secondary school, not completed,
5 = secondary school, completed, 6 = college/certificate, 7 = higher education)

10. How many years of education have you completed? ☐☐

Question 11: Do not ask someone who is older than 30, assume "no"

11. Are you still in school? (1 = yes, 2 = no) ☐

If "yes", skip question 12

12. What type of work do you do? ☐
(1 = not working, 2 = self-employed, 3 = family member assisting in the household/farm work, 4 = employee, 5 = employer, 6 = housewife)

13. Does your household have? (1 = yes, 2 = no)

- **a.** Electricity ☐
- **b.** A radio ☐
- **c.** TV ☐
- **d.** A refrigerator ☐
- **e.** A bicycle ☐
- **f.** A plough ☐
- **g.** A donkey ☐
- **h.** Cattle ☐

14. Material of floor. *Record observation.* (1 = concrete only, 2 = covered concrete, 3 = mud, 4 = wooden only, 5 = other, specify:……………

15. Has your household had enough food to eat during the last full year (12 months)? Would you say usually, sometimes, seldom or never? (1 = usually, 2 = sometimes, 3 = seldom, 4 = never)

16. How would you say your health is at the moment? *Probe if necessary*
(1 = very poor, 2 = poor, 3 = fair, 4 = good, 5 = very good)

17. How many times did you visit the following types of health services during the past 12 months (ask one by one type, and fill in the actual response):

a. public health centre/dispensary
b. public hospital
c. private (for profit) health facility
d. Mission/NGO health facility
e. traditional/informal/local sector

18. How many times in the past 12 months were you admitted to hospital or other health facility (99 = don't know)?

19. What type of health facility is nearest to you? (1 = public health centre/dispensary, 2 = public hospital, 3 = private (for profit) health facility, 4 = mission/NGO health facility, 5 = don't know, 6 = other, specify:……………………………….)

20. How would you in general rate the services offered at the nearest health facility? *Read the rating options:* (1 = very poor, 2 = poor, 3 = fair, 4 = good, 5 = very good)

21. How do you consider the availability of drugs at the nearest health facility? (1 = never available, 2 = rarely available, 3 = sometimes available, 4 = mostly available, 5 = always available, 6 = not applicable)

22. Where would you go to first if you had any of the following problems (*probe*):

	Mission/NGO clinic/hospital (1)	Public clinic/ hospital (2)	Private clinic/ hospital (3)	Traditional/ informal healers/doctors (4)	self-medication (5)	other - specify (6)	Do not know (7)
Serious headache							
Low back pain							
Diarrhoea							
Minor Injury							
Malaria							
Febrile child							
Labour							
STI[1]							

([1] Sexually transmitted infection; explain as genital sores, discharge and/or swelling in genital area)

23. The last time you visited a clinic or hospital, where did you go?
(1 = public facility, 2 = private facility, 3 = mission facility, 4 = not applicable) ☐

If "not applicable", go to 25

24. Was this facility the nearest one? (1 = yes, 2 = no) ☐

25. Does the cost of health services requiring immediate cash payment influence your utilisation of them? (1 = not at all, 2 = little, 3 = fairly, 4 = much, 5 = very much ☐

26. Does the distance to the nearest health services influence your utilisation of them? Rating: (1 = not at all, 2 = little, 3 = fairly, 4 = much, 5 = very much) ☐

27. How were you treated by the staff the last time you sought treatment at a health clinic or hospital? (1 = very badly, 2 = badly, 3 = fairly, 4 = well, 5 = very well) ☐

For the oldest among the tw household respondnents only

28. Does your household have any mosquito nets that can be used while sleeping? (1 = yes, 2 = no) ☐

29. If no to 28, why not? (1 = too expensive, 2 = not wanted, 3 = not available, 4 = don't know, 5 = other, specify:…………………………..) ☐

If "no" to 28, go to 32

30. Have the mosquito nets been treated with insecticide during the last 3 months? (1 = yes, 2 = no, 3 = don't know) ☐

31. Who slept under a mosquito net last night? (indicate with tick marks for each category of household members)

	All	Some	None	Not applicable
Children <5 years				
Pregnant women				
Other household members				

32. Are ITNs easily available in your district? (1 = yes, 2 = no, 3 = don't know) ☐

33. Apart from mosquito nets, are you currently using any other personal protective or preventive measures against malaria infection? (1 = yes, 2 = no, 3 = don't know) ☐

34. If yes to 33, what are you using? (1= coils, 2 = repellents, 3 = other, specify:……………….) ☐

35. Has any member of the household been ill with fever and/or convulsions during the last 4 weeks? (1 = yes, 2 = no, 3 = don't know) ☐

If "no" to 35, go to 41

36. Was advice sought for the fever/convulsion? (1 = yes, 2 = no, 3 = don't know) ☐

37. If yes to 36, where was advice sought first for the fever/convulsions?
(1 = public clinic/hospital, 2 = private clinic/hospital, 3 = mission/NGO health facility, 4 = traditional/informal healer, 5 = family members or neighbours, 6 = other, specify:……..…) ☐

38. If no to 36, why not? (1 = self-medication, 2 = quick recovery, 3 = other, specify:………..) ☐

39. Did the ill household member take any drugs for the fever and/or convulsions?
(1 = yes, 2 = no, 3 = don't know) ☐

40. Where did the drugs come from?
(1 = public clinic/hospital, 2 = private clinic/hospital, 3 = mission/NGO health facility,
4 = traditional/informal healer, 5 = community outlet/kiosk, 6 = home stock,
7 = other, specify:………………………………..) ☐

For women only:

I am now going to ask you some questions about pregnancy and motherhood

41. Have you ever been pregnant? (1 = yes, 2 = no) ☐

If "no" to 41, go to 48

42. During your last pregnancy,

Did you attend an antenatal clinic? (1 = yes, 2 = no) ☐

If yes, how many times ☐

To your knowledge, were you given drugs to prevent malaria at any such occasion ?
(1 = yes, 2 = no, 3 = don't know) ☐

43. Have you ever given birth? (1 = yes, 2 = no) ☐

If "no" to 43, go to 48

44. How long ago did you have your last delivery? (indicate number of months ago) ☐☐☐

45. Where did you deliver? *Probe*
(1 = at home, 2 = in public health centre/dispensary, 3 = public hospital, 4 = private health facility,
5 = mission/NGO health facility, 6 = other, specify:……………………………….) ☐

If delivered in a health facility, indicate name and place of facility:………………………..

46. What type of delivery was it? *Probe*
(1 = normal/vaginal delivery, 2 = caesarean section, 3 = vacuum extraction, 4 = don't know) ☐

47. If "caesarean section", what was the reason for delivering by caesarean section? (1 = emergency c-section because of medical reasons, 2 = elective c-section because of medical reasons known beforehand, 3 = personal choice, 4 = don't know) ☐

For all:

I am now going to ask you some questions about HIV/AIDS and sexual behaviour

48. Can a pregnant women who is infected with HIV transmit the infection to her unborn baby? (1 = yes, 2 = no, 3 = don't know) ☐

49. Is there anything which can be done to avoid transmission of HIV to the unborn baby? (1 = nothing, 2 = take herbs/traditional medicine, 3 = take ARVs from clinic, 4 = other, specify:…………………………………….) ☐

Question 50: Do not ask if person is married or have children, assume "yes".

50. Have you ever been sexually active? (1 = yes, 2 = no) ☐

If "no" to 50, go to 56

51. If yes, how many sexual partners did you have in the last 12 months? ☐☐

a) How many of these were marital/regular partners ☐☐

b) How many of these were casual partners ☐☐

52. Are condoms readily available in your district? (1 = never available, 2 = rarely available, 3 = sometimes, 4 = mostly available, 5 = always available, 6 = don't know) ☐

53. Did you use a condom the last time you had sex? (1 = yes, 2 = no) ☐

54. If "no" to 53, why not? (1 = too expensive, 2 = not available, 3 = not wanted, 4 = don't know, 5 = other, specify:…………………………………………….) ☐

Questions 55 & 56: Only ask if person has had sex with casual partners as indicated in question 51b.

55. Did you use a condom the last time you had sex with a *casual* partner? (1 = yes, 2 = no) ☐

56. How often do you use a condom with a casual partner? (1 = always, 2 = sometimes, 3 = never) ☐

57. How regularly do you receive HIV related information from the following sources? (1 = never, 2 = sometimes, 3 = often):

- **a.** Community health workers ☐
- **b.** Health clinic workers ☐
- **c.** Peer educators ☐
- **d.** Media (radio or TV) ☐

☐

e. Other, specify:……………………….

58. If you knew that a shopkeeper or food seller had the HIV/AIDS virus, would you buy fresh food from him/her? (Yes=1, No=2, don't know = 3

59. If a female teacher has the HIV/AIDS virus but is not sick, should she be allowed to continue teaching in school? (Yes=1, No=2, don't know =3

60. If a member of your family became infected with the HIV/AIDS virus, would you want it to remain a secret? (1 = yes, 2 = no, 3 = don't know)

(for q 58-60: we have given only 2 alternative yes or no

61. Do you know a place where you can get a confidential HIV test? (1 = yes, 2 = no)

62. Have you ever been HIV tested? (1 = yes, 2 = no, 9 = not answered)

63. If "no" to 62, why have you never taken a test? (1 = no access to testing, 2 = no knowledge of where to be tested, 3 = concerned about confidentiality, 4 = no wish to know/fear of knowing status, 5 = discouraged by partner, 6 = other, specify:………………………………………..)

If "no" to 62, go to 67

64. If "yes" to 62, was it voluntarily or required? (1 = voluntary/VCT-related,
2 = voluntary/PMTCT-related, 3 = mandatory/required,
4 = other, specify:…………………..…..)

65. How long ago since you were last tested? (indicate number of months)

66. We do not want to know your result, but did you receive the result? (1 = yes, 2 = no)

67. Have you considered going for a (or another) HIV test? (1 = yes, 2 = no)

68. Have you suffered from a sexually transmitted infection in the last 12 months?
(Yes=1, No=2)
(Explain STIs as e.g. genital sores, discharge, swelling in genital area)

69. *If No to 68, skip 69*
Did you receive treatment for this STI from a clinic? (Yes=1, No=2)

Thank you for participating!

FGDs first set (1B)

Guidelines WP6-9

Setting: FGDs among these 4 groups:
Female outpatients (x 2)
Male outpatients (x 2)
Pregnant women attending ANC (x 2)
Adolescents/youth aged 18-24 (x 2)

SOPs have been developed. The following are guidelines on the themes to be discussed - with focus on equity, quality and trust - and how it relates to the selected programmes (HIV, generalised care, malaria, and obstetric care)

Questions developed for use in the population-based survey are seen as indicator questions for studying changes over time relevant to equity, quality and trust by programme. The research team members should therefore make sure that they are familiar with this questionnaire. The ideal setting will be that many of the same themes included in the indicator questions can also be brought up at the group's level and possibly more penetrating discussion.

Outpatients (men and women separately)

General care

Introduction: We would like to learn about your perceptions of the health services. We would particularly like to know if the health services at the health facilities you know are trusted among people, and if they are perceived to be accessible and of good quality.

* How are the official health services in this area? OR What is your experiences with…
* What health service / health facility /-ies do you trust more / less?
  - Dispensary, health centre, hospital, local healing (government-voluntary agency?)
  - Probe: why? examples?
  - Probe: to what extent does trust in different health services differ with what people suffer from? (with 'diffuse/chronic illness to healers, malaria to the formal health system'..)
  - What should change for you to trust the dispensary / health centre / hospital more?
* What is your experience regarding the availability of drugs / medicine?
  - At the dispensary, health centre, hospital, local healers?
* What is your experience with the conduct of the health providers (courtesy, confidentiality, privacy)?
  - At the dispensary, health centre, hospital, local healers?
  - Differing categories of providers? (nurse assistants, nurses, physicians, healers)
  - In what way could health providers modify their conduct to improve your confidence in them / your respect for them?

Malaria

Introduction: Malaria is a major cause of illness and death. We would like to ask about your experience with preventive and treatment options.

* What measures do people in this area take to prevent malaria infection?
* Do you have experience with mosquito nets / mosquito nets treated with insecticide (ITN)?
  - Have you heard about / seen the ITN nets?
  - How easy / difficult is it to get these nets?
  - Why are you using / not using an ITN net?
* What is the availability of drugs for malaria treatment?
  - At the dispensary, health centre, hospital, local healing?

HIV

Introduction: Many people are also infected with HIV. We would like to ask about the preventive and treatment options in this area.

* If someone would like to know his / her HIV status, where would he / she go for a test?
  - How easy / difficult would it be to get a test?
* What are your experiences with HIV prevention programs?
  - Which campaign or group have presented the most trust-worthy solutions? (peer education, community health workers, media, particular health facilities)
  - To what extent are condoms useful to prevent HIV infection?
  - Are condoms easily available in this area?
  - What are people's perceptions about condoms?
* How are people who are infected with HIV treated?
  - By the community?

- By health providers (dispensary, health centre, hospital, healers)?

* How easy is it for someone infected with HIV to get access to treatment?
    - Perceptions about the treatment options?

Thank you very much for your time!

Adolescents/youth aged 18-24

Introduction: We would like to learn about young people's perceptions of the health services. We would particularly like to know if the health services at the health facilities you know are trusted among young people, and if they are perceived to be accessible and of good quality.

General care

* How are the official health services in this area? OR What is your experiences with…

* What health service / health facility /-ies do you trust more / less?

- Dispensary, health centre, hospital, local healing (government-voluntary agency?)
- Probe: why? examples?
- Probe: to what extent does trust in different health services differ with what people suffer from? (with 'diffuse / chronic illness to healers, malaria to the formal health system')
- What should change for you to trust the dispensary / health centre / hospital more?

* What is your experience regarding the availability of drugs / medicine?

- At the dispensary, health centre, hospital, local healers?

* What is your experience with the conduct of the health providers (courtesy, confidentiality, privacy)?

- At the dispensary, health centre, hospital, local healers?
- Differences between categories of providers? (nurse assistants, nurses, physicians, healers)
- In what way could health providers modify their conduct to improve your confidence in them / your respect for them?

HIV/STD

Introduction: Many people are also infected with HIV today. We would like to ask about your thoughts about the available preventive and treatment options.

* If someone would like to know his / her HIV status, where would he / she go for a test?

- How easy / difficult would it be easy to get a test?
- What are the reasons for young people not using VCT services?
- How could VCT services be modified to make them more acceptable for young people?

* What are your experiences with HIV prevention programs?

- Which campaign or group presented the most trust-worthy solutions? (peer education, community health workers, media, particular health facilities)
- Visibility of HIV programmes (map programmes observed by the participants, such as peer education, community health workers, condom campaigns, magazines, billboards etc.)
- What kinds of the involvement do the young have in HIV campaigns?
- To what extent are condoms useful to prevent HIV infection?
- Are condoms easily available in this area?
- Are condoms used by young people in this area?

* How are people who are infected with HIV treated by the community and by the health providers (at the dispensary, health centre, hospital, local healers)?

- If a family member became HIV infected, would it be better to keep it a secret?

* How easy is it for someone infected with HIV to get access to treatment?

- Perceptions about the treatment options?

* What can a person who suspects that he / she has been infected with a sexually transmitted infection do?
    - Where do most people go for treatment of STI's (dispensary, health centre, hospital, local healers?)
    - Availability of drugs for treating STI's?
    - Perceptions of STI treatment services?

Thank you very much for your time!

Pregnant women attending ANC

General care

Introduction: We would like to learn about your perceptions of the health services. We would particularly like to know if the health services at the health facilities you know are trusted among people, and if they are perceived to be accessible and of good quality.

* How are the official health services in this area? OR What is your experiences with…
* What health service / health facility /-ies do you trust more / less?
  - Dispensary, health centre, hospital, local healing (government-voluntary agency?)
  - Probe: why? examples?
  - Probe: to what extent does trust in different health services differ with what people suffer from? (with 'diffuse/chronic illness to healers, malaria to the formal health system..')
  - What should change for you to trust the dispensary / health centre / hospital more?

* What is your experience regarding the availability of drugs / medicine?
  - At the dispensary, health centre, hospital, local healers?

* What is your experience with the conduct of the health providers (courtesy, confidentiality, privacy)?
  - At the dispensary, health centre, hospital, local healers?
  - Differences between categories of providers? (nurse assistants, nurses, physicians, healers)
  - In what way could health providers modify their conduct to improve your confidence in them / your respect for them?

Obstetrics

* Where do women commonly deliver in this area?
  - Why?
  - What are your perceptions of deliveries at hospital/clinic level?
  - What alternatives are there?

Malaria

Introduction: Malaria is a major cause of illness and death. We would like to ask about your experience with preventive and treatment options.

* What measures do people in this area take to prevent malaria infection?
* Do you have experience with mosquito nets / mosquito nets treated with insecticide (ITN)?
  - Have you heard about them, seen them?
  - How easy / difficult is it to get them?
  - Why are you using / not using a net?

* What is the availability of drugs for malaria treatment?
  - At the dispensary, health centre, hospital, local healing?

HIV

Introduction: Many people are also infected with HIV. We would like to ask about your experience with preventive and treatment options.

* If someone would like to know his/her HIV status, where would he/she go for a test?
  - How easy / difficult would it be easy to get a test?

* What did you learn at the antenatal clinic about mother to child transmission of HIV?

- Knowledge about possibilities of avoiding HIV transmission from mother to child?
- What did the midwife inform about infant feeding options for HIV positive women?

* What are your experiences with HIV prevention programs?
  - Which campaign or group present the most trust-worthy solutions? (peer education, community health workers, media, particular health facilities)
  - To what extent are condoms useful to prevent HIV infection?
  - Are condoms easily available in this area?
* How are people who are infected with HIV treated by the community and by health providers (dispensary, health centre, hospital)?
  - If a family member became HIV infected, will it be better to keep it a secret?
* How easy is it for someone infected with HIV to get access to treatment?
  - Perceptions about the treatment options?

Thank you very much for your time!

FBS (2A & 2B)

IV

Questionnaire 1

RESPONSIVENESS QUESTIONNAIRE FOR HEALTH CARE PROVIDERS

**Name of Interviewer**
**Type of VCT** **(**1=Integrated, 2=Stand alone, 3=Community Based 4=Mobile) ☐ ☐<br><br>**Funded by:** (1=Faith Based organization, 2=Community, 3= Private, 4= Government)<br>**Name of Funder:**……………………………………..

Type of setting (1= Rural, 2= Peri-urban, 3= Urban) ☐

ID number ----------------------------

Date of interview (dd/mm/yyyy) __ __/ __ __ /__ __ __ __

Personal information:

1. What is your year of birth? (Indicate 9 if don't know) ☐☐☐☐
2. Sex (1 = male, 2 = female) ☐
3. What is your designation: (please check appropriate box)
4. Administrator ☐
   Doctor ☐
   Nurse ☐
   Counsellor ☐
   Other specify………………..
5. For how long have you been working here?
   (0= if less than 1 year, else indicate the number of years ………
6. Name of the VCT/ site? ……………………………………………

Now I will ask you questions about responsiveness of the VCT. Responsiveness means the following:

- Being treated with dignity
- Being attended promptly
- Having autonomy
- Having personal information kept confidential
- Having a choice of health care provider
- Having the health care provider communicate with you in a way you understand.
- Having amenities in the health care environment that are of an acceptable standard.

6. Dignity

Please circle the relevant response

		Never	Sometimes	Usually	Always
R1.1	How often are patients treated with respect	1	2	3	4
R1.2	How often are patients encouraged to discuss concerns freely	1	2	3	4
R1.3	How often are the patient encouraged to ask questions about the diseases, treatment and care	1	2	3	4
R1.4	How often is respect shown for the patient's desire for privacy during testing and counselling?	1	2	3	4

7. Autonomy

Please circle the relevant response

		Never	Sometimes	Usually	Always
R2.1	How often are patients provided with information on prevention and care of HIV?	1	2	3	4
R2.2	How often are patients consulted about their preferences over alternative HIV management options?	1	2	3	4
R2.3	How often is a patient's consent sought before testing	1	2	3	4

8. Confidentiality

Please circle the relevant response

		Never	Sometimes	Usually	Always
R3.1	How often are consultations carried out in a manner that protects a patient's confidentiality?	1	2	3	4
R3.2	How often is the confidentiality of information provided by patients preserved (except when it is needed by other health providers to facilitate care and treatment)?	1	2	3	4
R3.3	How often is the confidentiality of patients' medical records preserved (except if the information is needed by other health providers)?	1	2	3	4

9. Prompt attention

Please circle the relevant response

		Time in minutes				
R4.1	How long do clients often spend at centre waiting for consultations?	15 minutes and less	15-30	30-45	45-60	More than 60 min

10. Quality of Basic amenities

Please circle the relevant response

		Very Poor	Poor	Good	Very good		
R6.1	How would you rate the cleanliness of VCT centre?	1	2	3	4	**None**	**Don't Know**
R6.2	How would you rate the maintenance of VCT buildings?	1	2	3	4	5	6
R6.3	How would you rate the availability of furniture in the VCT?	1	2	3	4	5	6
R6.4	How would you rate access to clean water at the centre?	1	2	3	4	5	6
R6.5	How would you rate the cleanliness of toilets at the centre?	1	2	3	4	5	6
R6.6	How would you rate the testing kit	1	2	3	4	5	6

11. Choice of care provider

Please circle the relevant response

		Never	Sometimes	Usually	Always
R7.1	How often do individuals have a choice between health care providers at the VCT?	1	2	3	4
R7.2	How often do individuals have a choice between VCT centres?	1	2	3	4
R7.3	How often do individuals have an opportunity to see a specialist, if they wish to?	1	2	3	4

12. Please give a value between 0 and 10 to indicate your personal rating of <u>how important</u> the aspect is. Where 0 means not at all important and 10 means extremely important.

Code	Aspect / element	Abbreviation	Value
R8.1	Dignity	DIG	
R8.2	Autonomy	AUT	
R8.3	Confidentiality	CON	
R8.4	Prompt Attention	PRA	
R8.5	Quality of Basic amenities	QBA	
R8.6	Choice of Provider / Institution	CCP	

13. In your view are there any other characteristics beyond the ones we have discussed that you think should be included in a responsive VCT?
……………………………………………………………………………………………………
……………………………………………………………………………………………………
……………………………………………………………………………………………………
……………………………………………………………………………………………………
…………………………………………………………….

Thank you for your time

Questionnaire 2

RESPONSIVENESS EXIT QUESTIONNAIRES FOR USERS

**Name of researcher**
**Type of VCT (**1=Integrated, 2=Stand alone, 3=Community based, 4=Mobile) ☐<br><br>**Funded by:** (1=Faith Based organization, 2=Community, 3= Private, 4= Government) ☐<br>**Name of Funder**………………………………….

Type of setting (1= Rural, 2= Peri-urban, 3= Urban)

ID number ----------------------------

Date of interview (dd/mm/yyyy) __ __/ __ __ /__ __ __ __

1. What is your year of birth? (Indicate 9 if don't know) (Indicate age in years, or 9 = don't know) ☐☐☐☐

2. Sex (1 = male, 2 = female) ☐

3. What is your ethnic background? (Or what language is your mother tongue?)
   From numbered List of all possible. Also write actual response here…………………….

4. What is your marital status? (1 = single, never married, 2 = cohabitating/living as married, 3 = married, 4 = widowed, 5 = separated/divorced) ☐

5. Name of VCT / Site …………………………………..

Now I will ask you questions about responsiveness of the VCT. Responsiveness means the following:

- Being treated with dignity
- Being attended promptly
- Having autonomy
- Having personal information kept confidential
- Having a choice of health care provider
- Having the health care provider communicate with you in a way you understand.
- Having amenities in the health care environment that are of an acceptable standard.

6. **Dignity**
   Please circle the relevant response

		Yes	No	Other specify
S1.1	Were you treated with respect?	1	2	
S1.2	Were you encouraged to discuss your concerns freely?	1	2	
S1.3	Were you encouraged to ask questions about the disease? (Treatment and care)	1	2	
S1.4	Was there any privacy during testing?	1	2	
S1.5	Was there any privacy during counselling?	1	2	

7. **Autonomy**
Please circle the relevant response

		Yes	No	Other specify
S2.1	Were you provided with information on prevention and care of HIV?	1	2	
S2.2	Were you consulted about your preferences over alternative HIV management options?	1	2	
S2.3	Was your consent sought before testing?	1	2	
S2.4	Were you counselled?	1	2	

8. **Confidentiality**
Please circle the relevant response

		Yes	No	Other specify
S3.1	Was the consultations carried out in a manner that was confidential?	1	2	

9. **Prompt attention**
Please circle the relevant response

		Time in Minutes				
S4.1	How long did you spend waiting for consultations?	**15 minutes and less** 1	**15-30** 2	**30-45** 3	**45-60** 4	**More than 60 min** 5

10. **Quality of Basic amenities**
Please circle the relevant response

		Very Poor	Poor	Good	Very Good		
S6.1	How would you rate the cleanliness of VCT?	1	2	3	4	**None**	**Don't' Know**
S6.2	How would you rate the maintenance of the VCT building?	1	2	3	4	5	6
S6.3	How would you rate the adequacy/ availability of furniture at the VCT?	1	2	3	4	5	6
S6.4	How would you rate access to clean water at Centre?	1	2	3	4	5	6
S6.5	How would you rate the cleanliness of toilets at the centre?	1	2	3	4	5	6

11. **Choice of care provider**
Please circle the relevant response

		Yes	No	Other Specify
S7.1	Did you have a choice between health care providers at the VCT?	1	2	
S7.2	Did you have a choice between VCTs?	1	2	
S7.3	Did you wish to see a specialist?	1	2	
S7.3	Did you have an opportunity to see a specialist?	1	2	

12. **Please give a value between 0 and 10 to indicate your personal rating of <u>how important</u> the aspect is. Where 0 means not at all important and 10 means extremely important.**

**Code**	**Aspect / element**	**Abbreviation**	**Value**
S8.1	Dignity	DIG	
S8.2	Autonomy	AUT	
S8.3	Confidentiality	CON	
S8.4	Prompt Attention	PRA	
S8.5	Quality of Basic amenities	QBA	
S8.6	Choice of Provider / Institution	CCP	

13. **In your view are there any other characteristics beyond the ones we have discussed that you think should be included in a responsive VCT?**
……………………………………………………………………………………………………
……………………………………………………………………………………………………
……………………………………………………………………………………………………
……………………………………………………………………………………………………
………………………………………………….

Thank you for your time

FGDs and IDI (2C & 2D)

V

FGD guide for data set- 2C

1. **Je katika mila na desturi zenu 'Ushauri' una umuhimu gani.? Huwa unahusu nini?**
   - *How important is counselling in your cultural and social setting?*

   Probe: (What does it entail, define?)

2. **Ushauri hutolewa kukiwa na hali gani? Ni aina gani ya ushauri itumiwayo katika vituo vya Kupimwa Ukimwi kama VCT?**
   - *What circumstances call for counselling? (Examples), What kind of counselling is used at the HIV testing e.g. at VCT and PMTCT Centres?*

   Probe:

3. **Je watu wanaweza kufaidika na huduma za ushauri? Ni kwa njia gani?**
   - *Can people benefit from HIV counselling services?*

   Probe: How?

4. **Watu huchukuliaje kupimwa ukimwi katika eneo hili?**
   - *What are people's perceptions regarding HIV testing in this area?*

   Probe: Why?

5. **Ukimwi unafaa kupimwa katika mazingira gani?**
   - *In what kinds of environment should HIV testing be done? (Gender, Personnel training, location & infrastructure, equipment)*

   Probe:

6. **Je wafikiria kwamba kuna haja ya kuboresha upimaji wa ukimwi na huduma za VCT ?**
   - *Do you think that there is need for improvement in the area of HIV testing and VCT services?*

   Probe: Why, how, who should be responsible?

7. **Je ni yapi yakuboreshwa yanayoweza kukubalika na kustahimilishwa?**

   What improvements are acceptable and sustainable?

   Probe: How? When? Carried out by whom?

IDI guide data set 2 D

1. **Je katika mila na desturi zenu 'Ushauri' una umuhimu gani.? Huwa unahusu nini?**
    a. *How important is counseling in your cultural and social setting?*
    b. *Probe:What does it entail?*

2. **Ushauri hutolewa kukiwa na hali gani? Ni aina gani ya ushauri itumiwayo katika vituo vya VCT?**
    a. *What circumstances call for counseling? (examples), What kind of counseling is used at the VCT Centres and/ or PMTCT(only HIV or more group /individual?).*
    b. *Probe: What was your experience? How was it gone?*

3. **Je watu wanaweza kufaidika na huduma za ushauri? Ni kwa njia gani?**
    a. *Can people benefit from HIV counseling services?*
    b. *Probe: How? What do people say about counseling here (positive , negative)?*

4. **Unafikiria ama unaamini kikao kizuri cha ushauri kinafaa kiwe namna gani?**
    i. *Mshauri mzuri ni wa namna gani?*
    ii. *Ni mazingira ya aina gani ambayo ni mazuri kwa kutoa ushauri?*
    iii. *Ni nini linalofanya ushauri kuwa wa kuaminika?*

5. **Watu huchukuliaje kupimwa ukimwi katika eneo hili?**
    a. *What are people's perceptions regarding HIV testing in this area?*
    b. *Probe: Why?*

6. **Je waweza kuzungumzia vile ambavyo ungejisikia kabla ya kupimwa, wakati wa kupimwa na baada ya kupimwa ukimwi?**

experiences before a test, during the test and after the test

7. **Watu hapa huwachukuliaje wale waliopimwa ukimwi**

How do people perceive those who have tested for HIV here?

8. **Wanao enda kupimwa ukimwi zaidi ni wanawake ama wanaume?**

(Ni kwa nini?)
Those who go for tests here mainly are they men or women?
Probe: Why?

9. **Ni hali gani zingekufanya kutotumia (kukataa) huduma za kupimwa ukimwi?**

10. **Ukimwi unafaa kupimwa katika mazingira gani?**
    a. *Under what environment should HIV testing be done? (Gender, Personnel training, location &infrastructure, equipment)*

11. **Je wafikiria kwamba kuna haja ya kuboresha upimaji wa ukimwi na huduma za VCT ?**
    a. *Do you think that there is need for improvement in the area of HIV testing and VCT services (why)?*

12. Je ni yapi yakuboreshwa yanayoweza kukubalika na kustahimilishwa?

a. *What improvements are acceptable and sustainable? (Who)?*

Errata

Thesis

Section 3.6 ninth sentence: reference [84] should be [89]

Paper III
Figure 1 users “n=323” should read “n=328”
