# Supplementary material for: The accountability for reasonableness approach to guide priority setting in health systems within limited resources – findings from action research at district level in Kenya, Tanzania, and Zambia
Source: Health Res Policy Syst. 2014 Aug 20;12:49. doi: 10.1186/1478-4505-12-49 (PMC4237792; doi:10.1186/1478-4505-12-49)
Supplement: Additional file 2 — The REACT Consortium. [file 1478-4505-12-49-S2.doc]

## Inclusion criteria

- to be involved in REACT for at least 3 years
- specifically having been involved in one or more of the following activities:
- general conceptual development the REACT project
- development of work packages, concept papers, protocols and/or tools
- data collection
- development of data analysis strategy
- data analysis
- writing up
- dissemination

Exclusion criteria:

- Inactive staff
- Researchers doing associated studies with minimal use of REACT data

Institution of employment or team attachment for the project. Adresses:

1. DBL - Centre for Health Research and Development, Faculty of Life Sciences, University of Copenhagen, Thorvaldsensvej 57, DK 1871 Frederiksberg, Denmark
2. Centre for International Health (CIH) and Department of Public Health and Primary Health Care (ISF), University of Bergen, PO Box 7804, 5020 Bergen, Norway
3. Department of Public Health, Institute of Tropical Medicine, Nationalestraat 155, B 2000 Antwerpen, Belgium
4. Umea International School of Public Health (UISPH) Umea University, SE 90185 Umea, Sweden
5. Institute of Development Studies, University of Dar Es Salaam, PO Box 35169, Dar Es Salaam, Tanzania
6. National Institute of Medical Research (NIMR), PO Box 9653, Dar Es Salaam, Tanzania
7. Primary Health Care Institute (PHCI) PO Box 235, Iringa, Tanzania
8. Centre for Public Health Research, Kenya Medical Research Institute (KEMRI), Box 20752, Nairobi 00202, Kenya
9. Institute of Anthropology, Gender and African Studies (IAGAS) University of Nairobi, PO Box 30197, Nairobi 00100, Kenya
10. Department of Community Medicine (DCM), School of Medicine, PO Box 50110, University of Zambia, Zambia
11. Institute of Economic and Social Research (INESOR), PO Box 30900, University of Zambia, Zambia
12. Department of Health Policy, Management and Evaluation and the Joint Centre of Bioethics, University of Toronto, 88 College St, Toronto ON, M5G-1L4, Canada

| **Title, name, special REACT role** | **No. of the institutional team attached to. Designation or qualification.** | **Web, E-mail, Skype name. Country Code, Phone, Fax (F), Cell (C), Home (H)** |
| --- | --- | --- |
| Dr. Jens Byskov Coordinator, Team Leader | 1. Specialist in Public Health | [jby@life.ku.dk](mailto:jby@life.ku.dk) 35331418 C 23393204, H 45889334 |
| Dr. Paul Bloch | 1. Senior Researcher | [pabc@steno.dk](mailto:pabc@steno.dk) |
| Dr Øystein Evjen Olsen  Project advisor | 1. Senior research and capacity building advisor DBL and PHCI.  Evjen Olsen Health Systems Commitment | [oystein.olsen@cih.uib.no](mailto:oystein.olsen@cih.uib.no) [oysteineo@gmail.com](mailto:oysteineo@gmail.com)  No: +4740834583, Tz: +255786261886/ Ke: +254702976900 |
| Professor Douglas Martin, Project advisor, University of Toronto.  Project Advisor | 12. Dep. of Health Policy, Management and Evaluation and the Joint Centre of Bioethics, 88 College St., Toronto ON Canada M5G-1L4 | [douglas.martin@utoronto.ca](mailto:douglas.martin@utoronto.ca) Country code 1  416-978-6926, F416-978-1911 http://www.utoronto.ca/cpsrn/html/home.html |
| Professor Gunnar Kvåle, Team Leader | 2. MD, PhD (Mobil 41479180) | HREF="mailto:Gunnar.kvale@cih.uib.no" 55588563, H56300339, C 41479180 |
| Professor Knut Fylkesnes | 2. Professor | HREF="mailto:knut.fylkesnes@cih.uib.no"  55588576, C 91535360, H55328104 |
| Mr. Nils Gunnar Songstad | 2. Researcher | HREF="mailto:nils.gunnar.songstad@cih.uib.no"  55588336 C90733969, C255 787 202 812 |
| Ms. Ingvild F. Sandoy | 2. MD, researcher | Ingvild.Sandoy@cih.uib.no |
| Dr. Bjørg Evjen-Olsen | 2. Associate professor (part time only) MD, PhD | HREF="mailto:bjorg.olsen@cih.uib.no",  CIH: 55 588570 / Cell: +47 99295097 |
| Dr. Astrid Blystad | 2. Senior researcher | HREF="mailto:Astrid.blystad@isf.uib.no"  55586161 |
| Dr. Bruno Marchal | 3. Research fellow | [bmarchal@itg.be](mailto:bmarchal@itg.be) +32.32476384 |
| Dr. Anna-Karin Hurtig  Team Leader | 4. | [anna-karin.hurtig@epiph.umu.se](mailto:anna.karin.hurtig@epiph.umu.se)  90 7851164 F 90 138977 C37141090, H 90 194301 |
| Fredrik Norström | 4. Statistician | [fredrik.norstrom@epiph.umu.se](mailto:fredrik.norstrom@epiph.umu.se) 907858962, C705383543, H90120499 |
| Dr. Peter Kamuzora,  Country Coordinator, Team Leader | 5. Senior Lecturer | [petkamu@udsm.ac.tz](mailto:petkamu@udsm.ac.tz) , [pckamuzora@yahoo.com](mailto:pckamuzora@yahoo.com)  222410067/75, F /393/237, C 754801222 |
| Dr. Aggrey Kihombo | 5. Senior Lecturer | [akihombo@yahoo.com](mailto:akihombo@yahoo.com) 232604382 C784761975, F232604382 |
| Dr. Stephen Maluka | 5. Lecturer | E-mail: [stephenmaluka@yahoo.co.uk](mailto:stephenmaluka@yahoo.co.uk) Cell phone: +255 784 50 78 03 |
| Leon M. Mboera | 6. Senior Researcher | lmboera@nimr.or.tz. |
| Elisabeth Shayo | 6. Research scientist | [bshayo@yahoo.com](mailto:bshayo@yahoo.com) 222121400, C 754262924 |
| K. Senkoro | 6. Statistician | [ksenkoro@nimr.or.tz](mailto:ksenkoro@nimr.or.tz), [ksenkoro@yahoo.com](mailto:ksenkoro@yahoo.com) C787897613, 762106274, (755669319) |
| Mr. Emmanuel Makundi | 6. Research Scientist | [emakundi@nimr.or.tz](mailto:emakundi@nimr.or.tz) [elirurr@hotmail.com](mailto:elirurr@hotmail.com) C 754381351 |
| Dr. Benedict T. Ndawi  Team Leader | 7. Previous Director, PHCI | [bndawi@phci.ac.tz](mailto:btndawi@phci.ac.tz), [bndawi@yahoo.com](mailto:bndawi@yahoo.com)  26 2702633, F 118, C 754360986 |
| Mr. R.B. Momburi | 7. Acting Director | [rbmomburi@phci.ac.tz](mailto:rbmomburi@phci.ac.tz), [rbmomburi@yahoo.com](mailto:rmomburi@yahoo.com)  262702143 , C 754449761, 755883908 |
| Dr. Boniface Kasululu | 7. District Medical Officer | Mbarali, Mbeya |
| Dr. Yeri Kombe,  Country Coordinator, Team Leader | 8. Director, CPHR Kemri | [yerikom@yahoo.com](mailto:Yerikom@yahoo.com); [ykombe@kemri-nuitm.or.ke](mailto:ykombe@kemri-nuitm.or.ke) 202727646, 202725016/7 C.734257864 H202729013 |
| Ms. Mercy Karimi Njeru  8. | 8. Research Officer | [karimimercy@yahoo.com](mailto:karimimercy@yahoo.com)  202723006 |
| James N. Muttunga | 8. Chief Research Officer | [muttunga@yahoo.com](mailto:muttunga@yahoo.com), muttungaj@gmail.com C722808506, 202725016/7 |
| Mr. Joseph Mutai | 8. Senior Research Officer | [jmutai@kemri-nuitm.or.ke](mailto:jmutai@kemri-nuitm.or.ke), [joemutai@yahoo.com](mailto:joemutai@yahoo.com), C725082352 |
| Ms. Lillian Nyandieka | 8. Researcher – social science | [lnnyandieka@yahoo.com](mailto:lnnyandieka@yahoo.com) , 2725017/18 C723511331 |
| Dr. Anisa Omar | 8. Director of Public health, Coast Region | 722796220, [medicare@jambo.co.ke](mailto:medicare@jambo.co.ke) |
| DHE Mr. Matole | 8. Staff DHMT, Malindi |  |
| Professor Isaac Nyamongo, Team Leader | 9. Director, IAS University of Nairobi | [inyamongo@uonbi.ac.ke](mailto:inyamongo@uonbi.ac.ke) 203 744123 Fax same C 722 706 839 |
| Dr. W. Onyango-Ouma | 9. Senior Research Fellow | [onyango.ouma@uonbi.ac.ke](mailto:onyango.ouma@uonbi.ac.ke), [onyaouma@yahoo.com](mailto:onyaouma@yahoo.com)  202 444-9004/2121 Ex.2029/2058 C733716497 |
| Salome Bukachi | 9. Research Fellow | [sallybukachi@yahoo.com](mailto:sallybukachi@yahoo.com) C726771808 |
| Jared Maaka Siso | 9. Research Fellow & PhD student | [jared_maaka@yahoo.co.uk](mailto:jared_maaka@yahoo.co.uk), C722 283 857 |
| Professor, Seter Siziya | 10. Associate Professor of Medical Statistics | [ssiziya@yahoo.com](mailto:ssiziya@yahoo.com)  C 966748988/ 955752646 |
| Charles Michelo Country Coordinator, Team Leader | 10. Researcher. Chair of Department | [ccmichelo@yahoo.com](mailto:ccmichelo@yahoo.com), [cmichelo@hotmail.com](mailto:cmichelo@hotmail.com) +47 92618747  211266181 C 966754920, 979232403 |
| Selestine H. Nzala | 10. Lecturer | [selestinenzala@yahoo.com](mailto:selestinenzala@yahoo.com) C979176779 |
| Dr Selia Nga'njo. | 10. Lecturer | snganjop@yahoo.com |
| Carolyn M’soni | 10. Social Scientist | carolmsoni2003@yahoo.com |
| Oliver Mweemba | 10. Lecturer | [Mweemba2@yahoo.com](mailto:Mweemba2@yahoo.com) 1256181 |
| Joseph Zulu | 10. Researcher | [josemumba2000@yahoo.com](mailto:josemumba2000@yahoo.com), joseph.zulu@unza.zm |
| Mary Tuba  10. | 10. Medical Anthropologist | [mary_tuba2004@yahoo.co.uk](mailto:mary_tuba2004@yahoo.co.uk),  211228359 C979070493 |
| Dr. Charles Mwinuna | 10. District Director of Health, Kapiri Mposhi District | Charles Mwinuna [charlesmwinuna@yahoo.com](mailto:charlesmwinuna@yahoo.com) C 977862960 |
| Dr. Thabale Jack-Ngulube,  Team Leader | 11. Senior Research Fellow | [chessore@zamnet.zm](mailto:chessore@zamnet.zm), [thabalejackngulube@yahoo.com](mailto:thabalejackngulube@yahoo.com), [thabalejackngulube@gmail.com](mailto:thabalejackngulube@gmail.com) 211228359 (Chessore), 1294131, C955914844/978064903, F211228359 |

Institutional addresses:

**Supplementary institutional contact information**

| **Participant institution Title, name, special REACT role** | **Institutional Address, designation – (qualifications if desired)** | **Web, E-mail, *Skype name*. Country Code, Phone, Fax (F), Cell (C), Home (H)** |
| --- | --- | --- |
| **1. DBL-Centre for Health Research and Development (DBL)** | **University of Copenhagen, Faculty of Life Sciences**  **Thorvaldsensvej 57**  **DK 1871 Frederiksberg**  **Denmark** | [**www.DBL.life.ku.dk**](http://www.DBL.life.ku.dk/)  **Country code 45**  **35332828 F/35331433** |
| **2. Centre for International Health (CIH)** | **University of Bergen**  **Årstadveien 21 5th floor**  **N-5009 Bergen** | **HREF="http://www.cih.uib.no/" Country code 47**  **+47 55 588570/60. F 79** |
| **3. Prince Leopold Institute of Tropical Medicine (ITM)** | **Dep. of Public Health, Prince Leopold Institute of Tropical Medicine. Nationalestraat 155, B 2000 Antwerpen**  **Belgium** | [**www.itg.be**](http://www.itg.be/) **Country code 32**  **32476286. F /58** |
| **4. Umea International School of Public Health (UISPH)** | **Umea University**  **SE 90185 Umea**  **Sweden** | [**www.umu.se/phumed/epidemi**](http://www.umu.se/phumed/epidemi  )  **Country code 46**  **907851328. F 901138977** |
| **5. Institute of Development Studies (IDS)** | **University of Dar Es Salaam**  **P.O. Box 35169**  **Dar Es Salaam**  **Tanzania** | [**www.ids.udsm.ac.tz**](http://www.ids.udsm.ac.tz/)  **Country code 255**  **22 2410755 F/237** |
| **6. National Institute of Medical Research (NIMR)** | **P.O. Box. 9653 , Dar Es Salaam, Tanzania. Ocean Road.** | [**www.nimr.or.tz**](http://www.nimr.or.tz/) **Country code 255** [**nimrhqs@nimr.or.tz**](mailto:nimrhqs@nimr.or.tz)  **222 1 30770/1864 F /21360** |
| **7. Primary Health Care Institute (PHCI)** | **P.O.Box 235**  **Iringa**  **Tanzania** | [**www.phci.ac.tz**](http://www.phci.ac.tz/) **Country code 255**  **26 2702633 F 26 2702118** |
| **8. Centre for Public Health Research (CPHR)** | **Kenya Medical Research Institute (KEMRI), Box 20752, Nairobi 00202, Kenya.**  **Premises of Kenyatta National Hospital – next to the post office.** | [**www.kemri.org/CPHR**](http://www.kemri.org/CPHR)**,** [**cphr@kemri-nuitm.or.ke**](mailto:cphr@kemri-nuitm.or.ke)  **Country code 254**  **T. 20 2 72725016/7/8 F. 20 2 725012** |
| **9. Institute of Anthropology, Gender and African Studies (IAGAS)** | **University of Nairobi, P.O. Box 30197, Nairobi 00100, Kenya.**  **University Way, Education Building** | [**www.uonbi.ac.ke**](http://www.uonbi.ac.ke/) **Country code 254**  **203 744123**  [**director-aags@uonbi.ac.ke**](mailto:director-aags@uonbi.ac.ke) |
| **10. (10 a. by contract) Department of Community Medicine (DCM)** | **School of Medicine, P.O. Box 50110, University of Zambia (UNZA), Lusaka** | [**www.unza.zm/schools/medicine**](http://www.unza.zm/schools/medicine) **Country code 260**  **1 252641, 294131, F 1 294291** |
| **11. (10 b. by contract) Institute of Economic and Social Research (INESOR)** | **University of Zambia, P.O.Box 30900, Lusaka, Zambia.**  **Off Munali Road, Kaunda Square** | [**www.unza/inesor/unza**](http://www.unza/inesor/unza) [**inesor@zamnet.zm**](mailto:inesor@zamnet.zm) **Country code 260**  **1-294131/271/673, 1-295055 F 1-294291/253952** |
| **12. Associated: Joint Centre of Bioethics** | **Dep. of Health Policy, Management and Evaluation and the, 88 College St., Toronto ON Canada M5G-1L4** | [**douglas.martin@utoronto.ca**](mailto:douglas.martin@utoronto.ca) **Country code 1**  **416-978-6926, F416-978-1911 http://www.utoronto.ca/cpsrn/html/home.html** |
