# Supplementary material for: The accountability for reasonableness approach to guide priority setting in health systems within limited resources – findings from action research at district level in Kenya, Tanzania, and Zambia
Source: Health Res Policy Syst. 2014 Aug 20;12:49. doi: 10.1186/1478-4505-12-49 (PMC4237792; doi:10.1186/1478-4505-12-49)
Supplement: Additional file 4 — Equinet. [file 1478-4505-12-49-S4.pdf]

EQUINET

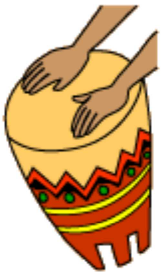

PUBLICATIONS

Newsletter - current issue

Bibliography on equity in health

Equinet publications

EQUINET CONFERENCE

OUR WORK

Meetings

Grants

Training

THEME AREAS OF OUR WORK

Equity in health

Values, policies and rights

Health equity in economic and trade policies

Poverty and health

Equitable health services

Human resources for health

Public-private mix

Resource allocation and health financing

Equity and HIV/AIDS

Governance and participation in health

Monitoring equity and research to policy

ABOUT US

Funders and partners

Steering committee

Links

HOME

Subscribe to the Equinet newsletter.
Your E-mail Address :

Subscribe

Or send an email to editor@equinetafrica.org with 'subscribe' in the subject line.

EQUINET Secretariat
Training and Research Support Centre (TARSC)
Box CY2720

EQUINET AFRICA

EQUINET, the Regional Network on Equity in Health in Southern Africa, is a network of professionals, civil society members, policy makers, state officials and others within the region who have come together as an equity catalyst, to promote and realise shared values of equity and social justice in health.

EQUINET gathers people to overcome isolation, give voice and promote networking using bottom-up approaches built on shared values. We have come together in a spirit of self determination and collective self reliance working through existing government, civil society, research and other mechanisms and institutions i n the Southern African Development Community (SADC) region and in southern and East Africa.

EQUINET is building a forum for dialogue, learning, sharing of information and experience and critical analysis. We do this to build knowledge and perspectiv es, shape effective strategies, strengthen our voice nationally, regionally and globally and our strategic alliances to influence policy, politics and practice towards health equity and social justice.

EQUINET's work covers a wide range of areas identified as priorities for health equity, within the political economy of health, health services and inputs to health, covered in the theme areas shown on this site. EQUINET is governed by a steering committee with representatives from fourteen institutions in southern Afr ica and is co-ordinated at the Training and Research Support Centre Zimbabwe.

CALL FOR APPLICANTS One Day Workshop 4 October 2014 Cape Town South Africa (post GSHSR) on "PARTICIPATORY ACTION RESEARCH IN PEOPLE CENTRED HEALTH SYSTEMS"- Call Closes on 30 June 2014!

This call below is for a ONE day workshop after the Global Symposium on Health Systems Research and is directed to all countries globally. We welcome you to apply! There is a separate call for delegates from East and Southern Africa for a three day skills workshop including this one day meeting.

This call invites applicants from all regions globally to participate and share experiences in an International Workshop on

Causeway
Harare, Zimbabwe
Tel + 263 4 705108/708835
Fax + 263 4 737220
Email: admin@equinetafrica.org

Feedback form

Site developed by Fahamu

‘Participatory action research in people centred health systems’ being held on 4th October Fountains Hotel, Cape Town, 4 October 2014 hosted by TARSC and pra4equity network in the Regional Network for Equity in Health in east and southern Africa (EQUINET) and Asociación Latinoamericana de Medicina Social (ALAMES), following the Global Symposium on Health Systems Research (30th Sept–3rd October).

More...

CALL FOR APPLICANTS FROM EAST AND SOUTHERN AFRICA: EQUINET THREE DAY SKILLS WORKSHOP ON PAR 4-6 OCTOBER 2014, Cape Town - CALL CLOSES JUNE 30!!

The call below is directed to people from East and Southern Africa only for a three day skills workshop on PARTICIPATORY ACTION RESEARCH IN PEOPLE CENTRED HEALTH SYSTEMS hosted by EQUINET that will include the one day post GSHSR workshop hosted by EQUINET and ALAMES on 4 October as the first day and a follow up two days on 5-6 October only for delegates from East and Southern African countries as an activity of the pra4equity learning network in EQUINET.

This call invites applicants from east and southern African countries to participate and share experiences in a three day skills workshop of the EQUINET pra4equity learning network on an ‘Participatory action research in people centred health systems’ being held on 4-6 October in Cape Town South Africa, following the Global Symposium on Health Systems Research (30th Sept–3rd October). It includes at the same venue on the first day (4 October) the one day post GSHSR workshop on PAR hosted by EQUINET and ALAMES (for which there is a separate call open to all regions), and the following two days (5-6 October) the skills training for the east and southern African delegates, hosted by EQUINET.

More...

Meetings

EQUINET Satellite Session at the GSHSR 30 September 1200-1400: New resources and opportunities for participatory research in health systems: areas of focus for Health systems Global”

EQUINET, COPASAH and Rotterdam Global Health Initiative Erasmus University, for the participatory cluster in the SHaPeS TWG for Health Systems Global is hosting a satellite workshop at the GSHSR on ”New resources and opportunities for participatory research in health systems: areas of focus for Health systems Global” on Tuesday, 30 September from 12.00 – 14.00. We welcome all interested in this work! The satellite session will be convened by the three organisations in the participatory cluster of the SHaPeS Technical working Group in HSR global. It will present and discuss with delegates interested in the cluster the

issues, resources and capacities for the field and how these could be developed through the TWG, and will make available work we have done to date, particularly through EQUINET and COPASAH. It will review the experience of using participatory action research, (PAR), community monitoring and innovations in social media in transforming local health systems, the challenges faced and the areas for future participatory work in HSR. It will launch the EQUINET, AHPSR and IDRC methods reader on participatory action research and web tools from COPASAH. The session will identify field building inputs in terms of the resources, capacity building, methods and opportunities that need to be taken forward by the participatory cluster of the SHaPeS Technical Working Group and the people who are interested in playing a role in the different areas of work. Contact admin@equinetafrica.org with GSHSR SATELLITE in the subject line for further detail or to let us know of your participation.

Grants

Equinet newsletter feed

EQUINET NEWS

MONITORING EQUITY AND RESEARCH POLICYFulfilling the Health Agenda for Women and Children: The 2014 Report - Mutual learning and reverse innovation - where next? - Handbook on Health Inequality Monitoring: with a special focus on low- and middle-income countries - Health policy and systems research in access to medicines: a prioritized agenda for low- and middle-income countries - A practical and systematic approach to organisational capacity strengthening for research in the health sector in Africa - Science for health diplomacy: complex problems need complex solutions - Biomedical research, a tool to address the health issues that affect African populations - Building research capacity in Africa: equity and global health collaborations - Adaptation and implementation of local maternity dashboards in a Zimbabwean hospital to drive clinical improvement - The parameters of the current legal framework for health research: Forms of health research which are regulated and obligations imposed on researchers

from Equinet newsletter, Produced by EQUINET steering committee

Latest Equinet Publications

Policy brief 37: Health Centre Committees as a vehicle for social participation in health systems in east and southern Africa
TARSC, CWGH, Medico (2014)
Themes: Governance and participation in health
Country: East and southern Africa region

URL: EQ Polbrief 37 HCC 2014.pdf

Read more...

Policy brief 38: Taking the WHO Global code of Practice on the International Recruitment of health Personnel in Africa from bottom drawer to negotiating table and action in Africa

U Limpopo, Mustang MC, ECSA, ACHEST, TARSC (2014)

Themes: Human resources for health, Governance and participation in health

Country: East and southern Africa region

URL: EQ Polbrief 38 Code 2014.pdf

Read more...

Policy brief 36: Increasing African agency in the design of Performance Based Financing

EQUINET, U Sheffield, et al (2014)

Themes: Resource allocation and health financing, Governance and participation in health

Country: East and southern Africa region

URL: EQ Polbrief 36 Gov.pdf

Read more...

EQUINET Discussion paper 102: African participation and partnership in performance-based financing: A case study in global health policy

Barnes A; Brown G; Harman S; Papamichail A; Banda P; Hayes R; Muliamba C (2014)

Themes: Equitable health services, Resource allocation and health financing, Governance and participation in health

Country: East and southern Africa region

URL: EQ GHD PBF Diss102.pdf

Read more...

EQUINET Discussion paper 103: The engagement of east and southern African countries on the WHO Code of Practice on the International Recruitment of Health Personnel and its implementation'

Dambisya YM; N Malema; C Dulo; S Matinhure; P Kadama (2014)

Themes: Equitable health services, Human resources for health, Governance and participation in health

Country: East and southern Africa region

URL: GHD Code Final rep Diss103 June2014.pdf

Read more...

Discussion paper 101: Health centre committees as a vehicle for social participation in health systems in east and southern Africa

Loewenson R; Machingura F; Kaim B; Training and Research Support Centre (TARSC); Rusike I (CWGH) (2014)

Themes: Equitable health services, Governance and participation in health

Country: East and southern Africa region

URL: EQUINET HCC Diss paper 101 FINAL.pdf

Read more...

Strengthening health centre committees as a vehicle for social participation in health in east and southern Africa: Regional Meeting Report, Harare, Zimbabwe 30 January - 1 February 2014

EQUINET; TARSC; CWGH; Medico Int (2014)

Themes: Equitable health services, Governance and participation in health

Country: East and southern Africa region

URL: EQ HCC Mtg Rep FEB2014.pdf

Read more...

Training Workshop on Participatory methods for a

people centred health system, 7-10 October 2013
Training and Research Support Centre; COPASAH; EQUINET (2013)
Themes: Equitable health services, Governance and participation in health
Country: East and southern Africa region
URL: PRA Reg Mtg Rep Oct2013.pdf

Read more...

Building empowered communities for health:_A film on health literacy and participatory approaches to health in Zambia
TARSC, Lusaka District Health Management Team, Ministry of Health Zambia September 2013 (2013)
Themes: Equitable health services, Governance and participation in health
Country: Zambia
URL: http://vimeo.com/72914294

Read more...

Discussion paper 100: Laws on management of public and private funds for health in Zimbabwe
Bhala B (2013)
Themes: Values, policies and rights, Resource allocation and health financing
Country: Zimbabwe
URL: Diss 100 Zim laws on funds Sep2013.pdf

Read more...

Next 10 publications
PUBLICATIONS | OUR WORK | ABOUT US | HOME
