# Supplementary material for: The accountability for reasonableness approach to guide priority setting in health systems within limited resources – findings from action research at district level in Kenya, Tanzania, and Zambia
Source: Health Res Policy Syst. 2014 Aug 20;12:49. doi: 10.1186/1478-4505-12-49 (PMC4237792; doi:10.1186/1478-4505-12-49)

Community Monitoring for Accountability and Equity in Health

This young and evolving field can be defined as the "systematic documentation and review of the availability, accessibility and quality of health services against specific government commitments or standards by actual beneficiaries of services, for the purpose of doing advocacy with providers and policy makers to improve the services".

What is relevant about community monitoring work?

Community monitoring is a bottom-up approach sustained by participatory processes. The documentation and analysis is carried-out by citizens themselves. Through social mobilization, monitoring results are used to demand concrete actions to bring about changes in service improvements and equitable distribution of resources. Since community monitoring is carried-out within and by socially excluded populations, it ads legitimacy to equity concerns and to specific demands for more responsive and people centered health systems.

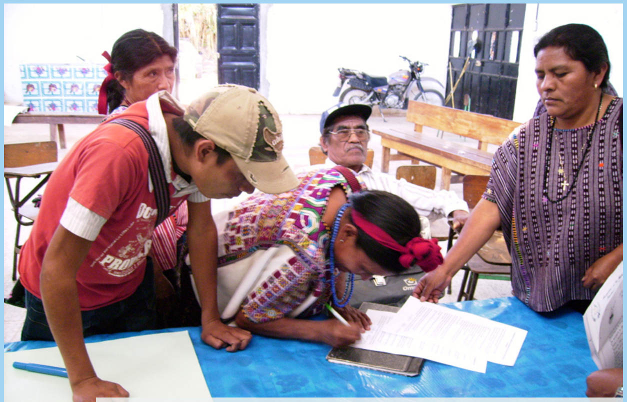

A Community group signing the monitoring report. Guatemala. Photo: Ismael Gomez

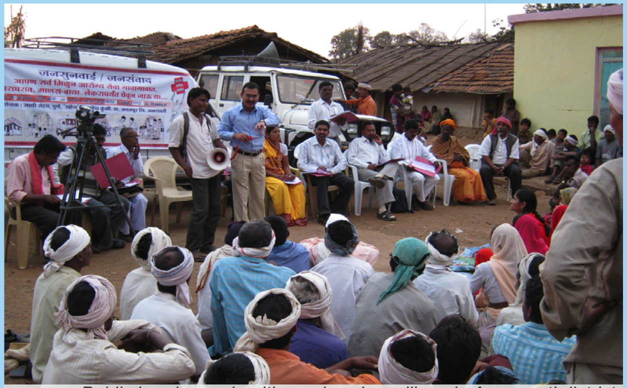

Public hearing on health services in a village in Amaravati district, Maharashtra state, India Photo credit - KHOJ / SATHI

What are the key features of community monitoring for accountability in health?

Although there is a variety of approaches to implement community monitoring, it is possible to identify some essential features:

1) It is based on an **'accountability framework'** and linked to government responsibility for the provision and/or overseeing of health services as a right to the people.

2) It reflects the **'people's or community perspective'** on health services and how governments are responding to their health rights towards realizing its health-related commitments.

3) It is an **'empowering process'** where capacities of participating people/community is enhanced to address power imbalances that affect their health.

4) It is linked to **'advocacy/action plan'** with the aim as changing or improving the implementation of health policies or programs and not a stand-alone activity with information collection as an end in itself.

A community of practitioners for community monitoring-COPASAH

COPASAH is an acronym meaning 'Community of Practitioners on Accountability and Social Action in Health'. COPASAH is a network in which practitioners come together through their common interest and passion for the field of community monitoring for accountability in health. COPASAH provides a space for the exchange of experiences, resources, capacities and methods that can lead to improve the production and dissemination of conceptual, methodological and practical outputs. These outputs are then used for the strengthening of the field and contribute to improving networking possibilities and capacity building among member organizations.

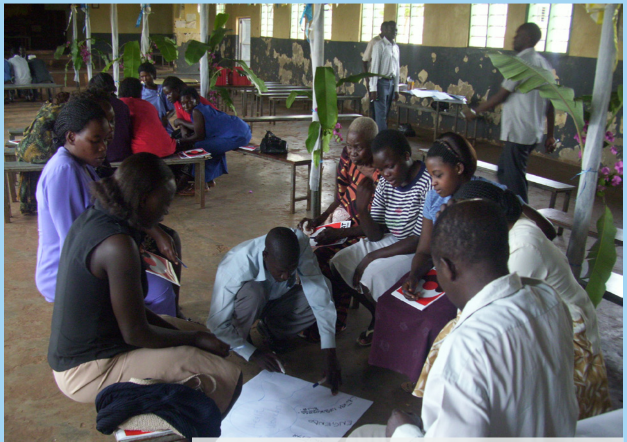

Participatory research meeting in Kiboga, Uganda. Photo: Mulumba Moses

COPASAH was established at the end of a three-day convening meeting held in Johannesburg, South Africa, in July 2011. Thirty-nine practitioners from thirty organizations and twelve countries from three different regions of the world (Africa, Asia and Latin America) actively participated, shared experiences and expressed the urgent need for establishing a community of practice.

COPASAH is currently led by a steering committee made up of at least two representatives from each of the three geographical regions (Africa, Asia and Latin America) represented in the Johannesburg convening meeting. Current members are:

NAME	ORGANIZATION AND COUNTRY
Abhay Shukla	Support for Advocacy and Training to Health Initiatives (SATHI), India.
Abhijit Das	Centre for Health and Social Justice (CHSJ), India.
Ariel Frisancho Arroyo	CARE, Peru
Rene Loewenson and Barbara Kaim	Training and Research Support Centre (TARSC), Zimbabwe.
Renu Khanna	SAHAJ, India.
Robinah Kaitiritimba	National Health Users/Consumers Organization (UNHCO), Uganda
Walter Flores	Centro de Estudios para la Equidad y Gobernanza en los Sistemas de Salud-CEGSS (Center for the Study of Equity and Governance in Health Systems), Guatemala.
Vinay Viswanatha	Accountability and Monitoring in Health Initiative (AMHI), USA.

COPASAH is currently supported by the Accountability and Monitoring Health Initiative (AMHI) of the Open Society Foundation.

What are the key COPASAH activities for 2012?

COPASAH is currently implementing an ambitious action plan that seeks to strengthening the field of community monitoring and the capacity building of its members through the collation, production and dissemination of conceptual, methodological and practical outputs using different media including published materials and the Internet. The key activities to be implemented during the year 2012 are as follows:

- Develop an interactive website to keep members updated and that will collect the resources produced by COPCOM members.
- Writing-up and disseminating eight case studies on community monitoring based on the experience of selected member organization.
- Writing –up and disseminating five issue papers:
    - Theories of change in community monitoring.
    - Theory and methods for participatory knowledge in health systems and community monitoring.
    - Tracking and assessing progress and evaluating impacts in community monitoring.
    - Social accountability of private sector services.
    - Role and ethics of facilitating organizations: putting people center-stage.
- Translate key tools and approaches from South Asian Languages and Spanish into English.
- Organize three regional meetings (one in Latin America, one in Africa and one in Asia) in order to review the case studies, issue papers and work on strategic planning for future work in the region.
- Maintain active communication with all the member organizations through a listserv and a quarterly newsletter.

How to get in contact with COPASAH?

Visit our website www.copasah.net or get in touch with any of our regional coordinators:

Executive Secretariat and Latin America region:
Walter Flores. Centro de Estudios para la Equidad y Gobernanza en los Sistemas de Salud-CEGSS (Center for the Study of Equity and Governance in Health Systems). Guatemala.
Email: waltergflores@gmail.com

Africa region:
Robinah Kaitiritimba. National Health Users/Consumers Organization (UNHCO), Uganda.
Email: rkitungi@unhco.or.ug

Asia region:
Abhijit Das. Centre for Health and Social Justice (CHSJ), India.
Email: abhijitchsj@gmail.com

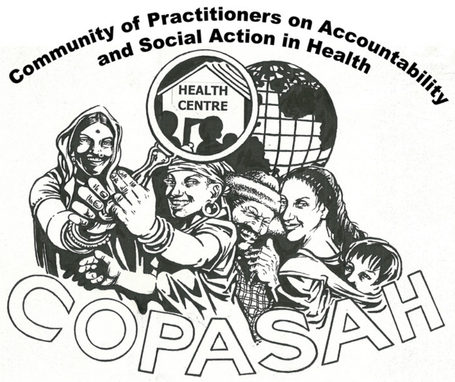

Supplement: Additional file 5 — Community of Practitioners on Accountability and Social Action for Health - COPASAH. [file 1478-4505-12-49-S5.pdf]
